# Conformational heterogeneity of the Pfr chromophore in plant and cyanobacterial phytochromes

**DOI:** 10.3389/fmolb.2015.00037

**Published:** 2015-07-10

**Authors:** Francisco Velazquez Escobar, David von Stetten, Mina Günther-Lütkens, Anke Keidel, Norbert Michael, Tilman Lamparter, Lars-Oliver Essen, Jon Hughes, Wolfgang Gärtner, Yang Yang, Karsten Heyne, Maria A. Mroginski, Peter Hildebrandt

**Affiliations:** ^1^Institut für Chemie, Technische Universität BerlinBerlin, Germany; ^2^Botanisches Institut, Karlsruher Institut für TechnologieKarlsruhe, Germany; ^3^Fachbereich Chemie, Philipps-Universität MarburgMarburg, Germany; ^4^Institut für Pflanzenphysiologie, Justus Liebig UniversityGießen, Germany; ^5^Max-Planck-Institut für Chemische EnergiekonversionMülheim, Germany; ^6^Institut für Experimentalphysik, Freie Universität BerlinBerlin, Germany

**Keywords:** phytochrome, tetrapyrrole, isomerization, structural heterogeneity, hydrogen bonding, resonance Raman spectroscopy, time-resolved IR spectroscopy, quantum chemical calculations

## Abstract

Phytochromes are biological photoreceptors that can be reversibly photoconverted between a dark and photoactivated state. The underlying reaction sequences are initiated by the photoisomerization of the tetrapyrrole cofactor, which in plant and cyanobacterial phytochromes are a phytochromobilin (PΦB) and a phycocyanobilin (PCB), respectively. The transition between the two states represents an on/off-switch of the output module activating or deactivating downstream physiological processes. In addition, the photoactivated state, i.e., Pfr in canonical phytochromes, can be thermally reverted to the dark state (Pr). The present study aimed to improve our understanding of the specific reactivity of various PΦB- and PCB-binding phytochromes in the Pfr state by analysing the cofactor structure by vibrational spectroscopic techniques. Resonance Raman (RR) spectroscopy revealed two Pfr conformers (Pfr-I and Pfr-II) forming a temperature-dependent conformational equilibrium. The two sub-states—found in all phytochromes studied, albeit with different relative contributions—differ in structural details of the *C*-*D* and *A*-*B* methine bridges. In the Pfr-I sub-state the torsion between the rings *C* and *D* is larger by ca. 10° compared to Pfr-II. This structural difference is presumably related to different hydrogen bonding interactions of ring *D* as revealed by time-resolved IR spectroscopic studies of the cyanobacterial phytochrome Cph1. The transitions between the two sub-states are evidently too fast (i.e., nanosecond time scale) to be resolved by NMR spectroscopy which could not detect a structural heterogeneity of the chromophore in Pfr. The implications of the present findings for the dark reversion of the Pfr state are discussed.

## Introduction

**Figure 1:**
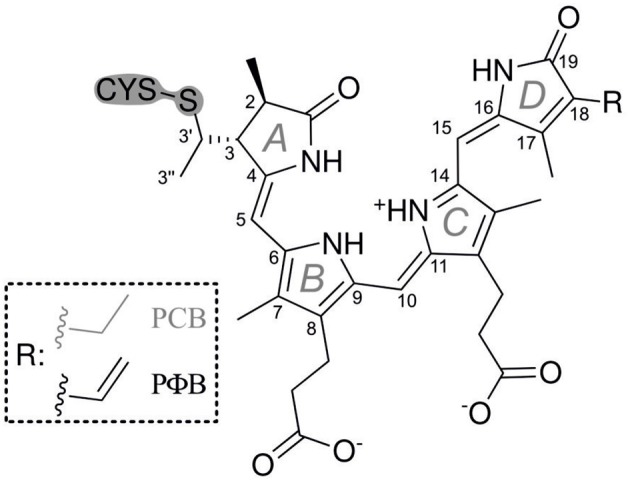
**Structural formulae of the PCB and PΦB chromophores in the *ZZEssa* configuration of Pfr**.

Phytochromes are ubiquitous photoreceptor in plants that utilize light as a source of information for controlling photomorphogenic processes (Quail, 1998; Schäfer and Nagy, 2006). Upon light excitation phytochromes are interconverted between the red-absorbing (Pr) and far-red absorbing state (Pfr), corresponding to a switch between physiologically inactive and active states, respectively. The light-absorbing cofactor is a linear methine-bridged tetrapyrrole, phytochromobilin (PΦB), that is covalently linked to a cysteine residue via a thioether bridge formed with the vinyl substituent of ring *A* (Figure 1) (Gärtner and Braslavsky, 2004; Rockwell et al., 2006; Rockwell and Lagarias, 2010). The primary photochemical step of the Pr → Pfr conversion is a double-bond isomerization (*Z*/*E*) of the *C-D* methine bridge (Rockwell et al., 2006; Rockwell and Lagarias, 2010). Subsequent steps include protein motions which eventually induce the functional relevant structural changes leading to the physiological signal. Phytochrome can therefore be regarded as a bimodal photoswitch which is based on the photoinduced conversion between the *ZZZssa* (Pr) and *ZZEssa* (Pfr) tetrapyrrole configuration. In addition to photoconversion, a unidirectional thermal pathway allowing dark reversion of Pfr to Pr is often apparent. Amongst the prokaryotic bacteriophytochromes, the resting state of the small “bathy”-type group is Pfr rather than Pr. Here dark reversion from Pr to Pfr takes place, corresponding to a thermal *E* → *Z* double bond isomerization that is initiated by a keto/enol tautomerization (Velazquez Escobar et al., 2015). It might well be that an analogous mechanism also holds for Pfr → Pr dark reversion in canonical phytochromes as suggested earlier (Lagarias and Rapoport, 1980).

Most of the knowledge in molecular phytochrome research was obtained from cyanobacterial phytochromes and bacteriophytochromes which employ phycocyanobilin (PCB) and biliverdin (BV) as chromophores, respectively (Rockwell and Lagarias, 2010). These phytochromes are more tractable than plant phytochromes and thus, the first three-dimensional (3D) structures at atomic resolution were obtained from these representatives of the superfamily (Wagner et al., 2005, 2007; Yang et al., 2007, 2008, 2009, 2011; Essen et al., 2008; Malliet et al., 2011; Bellini and Papiz, 2012; Anders et al., 2013, 2014; Takala et al., 2014). Crystallographic studies of a bacteriophytochrome also revealed the protein structural changes implicated in regulating the output module (Takala et al., 2014) that is typically a histidine kinase-like region. Only recently 3D structural data have been obtained for plant phytochromes (Song et al., 2012; Burgie et al., 2014). Otherwise, structural investigations of plant phytochromes were largely restricted to spectroscopic approaches, including nuclear magnetic resonance (NMR), transient absorption, resonance Raman (RR), and infrared (IR) spectroscopy (Fodor et al., 1988, 1990; Mizutani et al., 1994; Matysik et al., 1995; Kneip et al., 1997, 1999; Andel et al., 2000; Gärtner and Braslavsky, 2004; Mroginski et al., 2004, 2011a,b; Murgida et al., 2007; Rohmer et al., 2008; Schwinté et al., 2008; Dasgupta et al., 2009; Song et al., 2012, 2013). These results together with spectroscopic data and molecular modeling studies (Mroginski et al., 2011b) demonstrated extensive similarities in the overall fold and the chromophore structure in the parent states of plant, cyanobacterial, and bacteriophytochromes, although the different chromophores (PΦB vs. BV) attach to different Cys residues (Lamparter et al., 2002; Rockwell et al., 2006).

Although most spectroscopic studies on canonical phytochromes have focused on the thermally stable Pr state (Rockwell et al., 2006; Rockwell and Lagarias, 2010), important structural insight has also been obtained for the Pfr state albeit with partly conflicting conclusions. Based on NMR spectroscopy on the canonical cyanobacterial phytochrome Cph1 as well as plant phytochrome A, Matysik and co-workers demonstrated that the chromophore was held rigidly in the binding pocket of Pfr, whereas in the Pr state the chromophore was much more flexible—indeed showing two distinct substates (Song et al., 2011, 2012, 2013), as implied by fluorescence spectroscopy (Sineshchekov et al., 1998). However, time-resolved optical spectroscopies of plant phytochrome A provided evidence for a conformational heterogeneity in both the Pr and Pfr states, corresponding to two parallel photo-induced reaction pathways (Schmidt et al., 1998; Sineshchekov, 2004). Essentially, the same conclusions were derived from transient absorption spectroscopy of Cph1, covering a wide dynamic range (Kim et al., 2013, 2014a,b). A heterogeneous chromophore structure has also been demonstrated for the Pfr state of algal phytochromes on the basis of circular dichroism (CD) spectroscopy (Rockwell et al., 2014). Furthermore, a recent RR spectroscopic study on BV-binding bacteriophytochromes revealed a homogeneous chromophore structure in the Pfr state only for representatives of the bathy-phytochromes family, whereas a temperature-dependent equilibrium between two Pfr conformers was also observed for prototypical phytochromes (Salewski et al., 2013). This structural heterogeneity was suggested to be associated with the thermal double bond isomerization preceding the Pfr → Pr dark reversion.

In this work, we have extended these studies to the Pfr state of various phytochromes that bind PΦB or PCB. We have employed RR spectroscopy that selectively probes the vibrational spectrum of the cofactor representing a characteristic fingerprint of the structure of the tetrapyrrole and its interactions with the protein environment (Mroginski et al., 2011a). To support the vibrational assignment and thus the structural analysis of the chromophore, we have used phyA adducts including different tetrapyrroles (PΦB vs. PCB) and selectively ^13^C-labeled isotopomers of PCB. These static RR experiments were complemented by time-resolved IR spectroscopy to determine conformational distributions specifically of ring *D*. The main goal of this work is to explore possible structural heterogeneities of the chromophore in the Pfr state that might provide insights into the role of conformational dynamics in the thermal isomerization of the tetrapyrrole.

## Materials and methods

### Protein expression, purification, and reconstitution

PCB (and its isotopomers) and PΦB were assembled in a 5:1 molar ratio with the recombinant His-tagged 65 kDa (residues 1–595) N-terminal photosensory module of oat phyA3 apoprotein as described previously (Mozley et al., 1997; Song et al., 2012). The adduct showed absorption maxima at 650 and 715 nm for Pr and Pfr, respectively. The isotopic labeling affected neither the absorption maxima, the photochemical behavior, nor the thermal stability. Production, purification, and chromophore assembly of Cph1, Cph2, CphA, and Agp1-V249C have been described elsewhere (Landgraf et al., 2001; Essen et al., 2008; Borucki et al., 2009; Schwinté et al., 2009; Anders et al., 2011). In each case, the experiments were carried out with the photosensory module of the proteins, i.e., N-terminal PAS, GAF, and PHY domains. For the sake of simplicity, the deletion of the output module is not specifically indicated here, e.g., the notation Cph1 corresponds to the commonly used abbreviation Cph1Δ2. As long as no further modifications are specified such as Agp1-V249C, these photosensor modules are referred to as wild-type (WT) variants. RR experiments were carried out in 50 mM Tris, 300 mM NaCl, 5 mM EDTA in H_2_O (D_2_O) at pH (pD) of 7.8. Protein samples were concentrated by ultrafiltration to an optical density of ca. 50 at 280 nm. Typical protein concentrations for the RR experiments were between 400 and 600 μ M.

### Syntheses

^13^C(5)-PCB and ^13^C(15)-PCB were synthesized according to Makhynya et al. (2007). The synthesis followed the convergent strategy by generating the right and the left half of PCB separately (Figure 1), followed by condensation of both compounds at the central C(10) position as described previously (Mroginski et al., 2011b). Isotope content at the labeled position of the target PCB was >95% as determined by mass spectrometry.

### Resonance raman spectroscopy

RR spectra of the Pr state of phyA were obtained with 1064-nm excitation (Nd-YAG cw laser, line width <1 cm^−1^) with a Bruker RFS 100/S Fourier-transform Raman spectrometer (4 cm^−1^ spectral resolution). All spectra were measured at −140°C using a liquid-nitrogen cooled cryostat (Linkam). The laser power was ca. 0.4 W at the sample which does not cause any laser-induced damage of the protein samples as checked by comparing the spectra obtained before and after a series of measurements. Data was accumulated for ca. 2 h for each spectrum. In all RR spectra shown in this work, the background as well as contributions from the Pr state were subtracted. For the band fitting analysis of selected spectral regions, the contribution of the apoprotein was also subtracted (see also Salewski et al., 2013; Zienicke et al., 2013).

### Time-resolved VIS pump IR probe spectroscopy

Pump and probe pulses were generated using non-linear optical methods. By difference frequency mixing in various steps, we obtained mid-IR pulses of 200 fs (FWHM) or shorter at a repetition rate of 1.088 kHz. Simultaneously, 200 fs laser pulses at 710 nm were used to photoexcite the sample at the absorption maximum of the Pfr state, thus initiating the photoreaction. Photoselection experiments were performed using focal pump pulse diameters of 300 μm, sample thickness of 50 μm, focal probe pulse diameters of 150 μm, pulse energies of <100 nJ. This results in excitation efficiencies below 8%. The transient absorptions for parallel A_p_ and perpendicular A_s_ polarization were simultaneously probed by two mid-IR pulses with polarizations oriented parallel and perpendicular to the pump pulse polarization at various delay times. The isotropic polarized absorption A_iso_ was calculated by A_iso_ = (A_p_ + 2A_s_)/3 at each delay time. The time-resolved data presented show isotropic polarized absorption. Probe pulses were dispersed with an imaging spectrograph at a resolution of 1.5 cm^−1^ and recorded with a 2 × 32 element MCT array detector, resulting in transient spectra with high spectral resolution (Linke et al., 2013). The high repetition rate requires that the sample be moved across the focused laser beams with a Lissajous sample cell in order to avoid multiple excitation of a specific sample volume. The ^13^C/^15^N labeled Cph1 phytochrome apoprotein, to which non-labeled PCB chromophore was added) was prepared in D_2_O solution at an optical density of 0.15–0.2 OD at 710 nm, as described previously (Hahn et al., 2008; Robben et al., 2010). Background illumination at wavelengths of ~640 nm ensured that the sample remained in the Pfr form.

### Quantum chemical calculations

Vibrational spectra of tetrapyrroles in the *ZZEssa* configuration were calculated by density functional theory (DFT) using the B3LYP functional and the 6–31G^*^ basis set. All spectra refer to protonated (cationic) tetrapyrroles with a chloride ion in the vicinity of the pyrrole N-H groups serving as a counterion. Further details of the computational methods are given elsewhere (Schwinté et al., 2008). Due to the lack of a complete atomic model for a canonical phytochrome in the Pfr state, the calculations in this case refer to the chromophore *in vacuo*, thus ruling out structural interpretation (Mroginski et al., 2009). However, as shown by comparison with previous theoretical analyses of phytochromes with known 3D structures (Mroginski et al., 2011b; Salewski et al., 2013), the calculations can be used to determine the number of normal modes in specific spectral regions and to assess the character of these modes including the expected isotopic shifts. Calculated frequencies, intensities, and normal mode compositions for PΦB and the different PCB isotopomers are given in the Supplementary Material.

## Results

**Figure 2:**
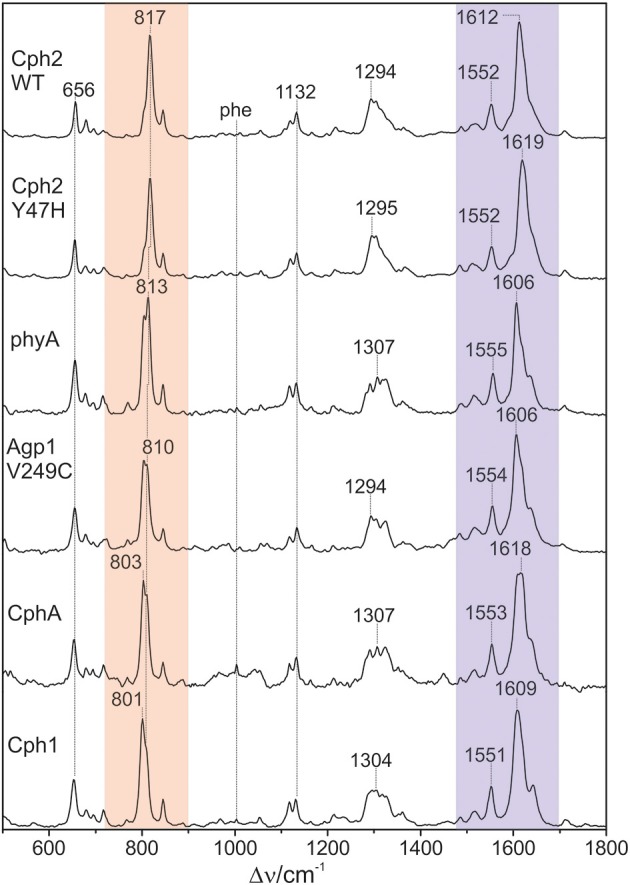
**Overview RR spectra of the Pfr states of various PCB-binding phytochromes**. The HOOP and C = C stretching regions are colored in pale red and violet, respectively. The spectra were measured from buffered H_2_O solutions (pH 7.8) with 1064 nm excitation at -140°C. Further details are given in the text (Sections Resonance Raman Spectroscopy and Results).

The 1064 nm excitation line is ideally suited for selectively probing the vibrational spectrum of the chromophore in the Pfr state of phytochromes. Due to its red-shift compared to the absorption maximum of the chromophore (ca. 700 nm), interference of the Raman spectrum with the chromophore fluorescence as well as unwanted photochemical reactions are avoided, whereas the energy of the excitation line is still sufficient for selective resonance enhancement of the Raman bands of the chromophore (Mroginski et al., 2011a). The only contribution of protein Raman bands refers to the Phe mode at ca. 1004 cm^−1^ which, however, is only of very low intensity (Figure 2). Besides, the RR spectra exclusively display the chromophore bands of the Pfr state.

Figure 2 shows a collection of Pfr spectra obtained from various PCB-binding canonical phytochromes, including those in which PCB is the natural chromophore, i.e., Cph1, CphA, and the WT and the Y47H variant of Cph2, as well as plant phytochrome phyA which in planta binds PΦB. Furthermore, we have studied a variant of the bacterial phytochrome Agp1, Agp1-V249C, in which the natural BV attachment site was by one at position 249 to allow for binding of PCB (or PΦB, *vide infra*) (Borucki et al., 2009). In each case, the characteristic overall band pattern of Pfr is clearly visible, including two regions with prominent bands around 800 and 1600 cm^−1^ originating from modes that are dominated by hydrogen-out-plane (HOOP) (orange rectangle) and C = C stretching coordinates (blue rectangle) of the methine bridges, respectively. The modes in these regions are largely localized in specific parts of the tetrapyrrole and dominated by a single internal coordinate (Mroginski et al., 2011b; Salewski et al., 2013). Thus, spectral changes of these modes in the spectra of the various phytochromes can be more easily related to specific structural changes as compared to variations of the bands in other parts of the spectra, such as between 1200 and 1400 cm^−1^ where the individual modes contain comparable contributions of a large number of coordinates. We therefore restrict a more detailed analysis to the HOOP and C = C stretching regions.

### Vibrational analysis: hoop region

**Figure 3:**
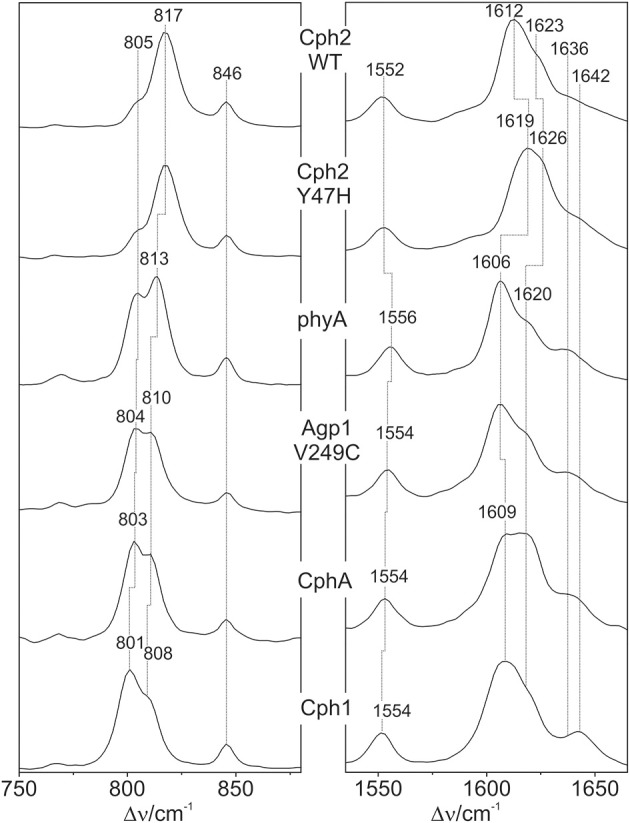
**Expanded view of the HOOP (left) and C = C stretching region (right) of the RR spectra of the Pfr states of various phytochromes including the PCB chromophore (see Figure 2)**. The spectra were measured from buffered H_2_O solutions (pH 7.8) with 1064 nm excitation at -140°C. Further details are given in the text (Sections Resonance Raman Spectroscopy and Results).

**Figure 4:**
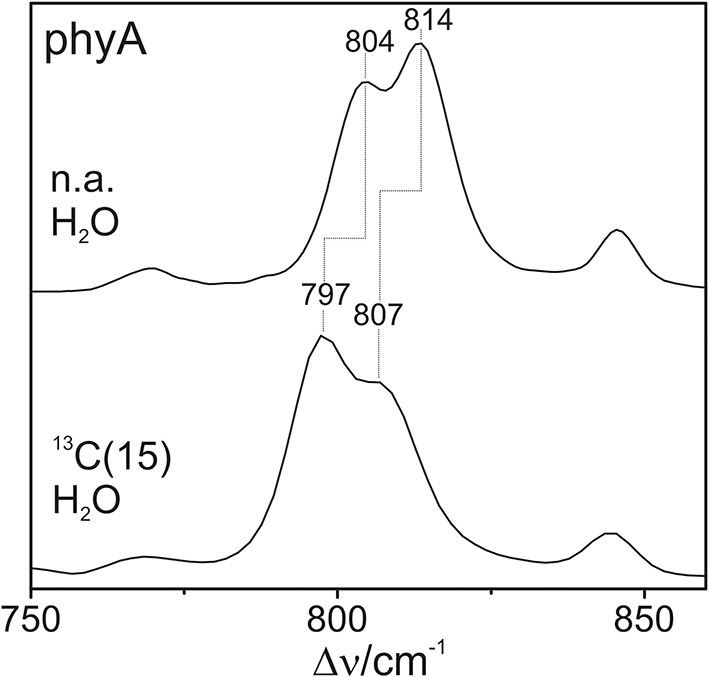
**Expanded view of the HOOP region of the RR spectra of the Pfr state of phyA including the non-labeled (natural abundance, n.a.) PCB and the PCB chromophore ^13^C labeled at C(15)**. The spectra were measured from buffered H_2_O solutions (pH 7.8) with 1064 nm excitation at -140°C. Further details are given in the text (Sections Resonance Raman Spectroscopy and Results).

The Pfr states of all phytochromes display strong RR activity around 800 cm^−1^ attributed to the HOOP mode of the *C-D* methine bridge (Fodor et al., 1988; Mroginski et al., 2011b; Salewski et al., 2013). The high RR intensity was proposed to be related to the torsion of ring *D* with respect to the remainder of the tetrapyrrole (Fodor et al., 1988). However, the present spectra demonstrate two closely-spaced bands with different relative intensities in the various phytochromes (Figure 3). The intensity ratio of the high- to the low-frequency component varies by more than a factor of three among the different species, accompanied by frequency shifts between 5 and 10 cm^−1^. In principle, these bands might originate from two modes of the same chromophore conformer or of the same mode of two conformers. To distinguish between these possibilities we compare the RR spectra of phyA assembled with ^13^C-labeled and non-labeled PCB (Figure 4), which shows the shift of the 804/814 cm^−1^ band pair to 797/807 cm^−1^ when the C(15) (*C-D* methine bridge) position is labeled. Neither labeling at the C(5) (*A-B* methine bridge) position nor D/H exchange at the pyrrole nitrogens has a significant effect on the spectrum (data not shown), ruling out the assignment of one of these bands to a HOOP mode of the *A-B* methine bridge or a N-H out-of-plane deformation mode. Thus, we conclude that both bands are due to HOOP modes of the *C-D* methine bridge but originating from two different conformers. This interpretation is supported by quantum chemical calculations of the free PCB which predict only one mode of strong RR intensity in this region (820 cm^−1^) (Supplementary Material). This mode is dominated by the HOOP mode of the *C-D* methine bridge which is predicted to show a ^13^C/^12^C isotopic shift at position C(15) of −8 cm^−1^, similar to the experimentally determined shifts of −7 cm^−1^.

### Vibrational analysis: C = C stretching region

**Figure 5:**
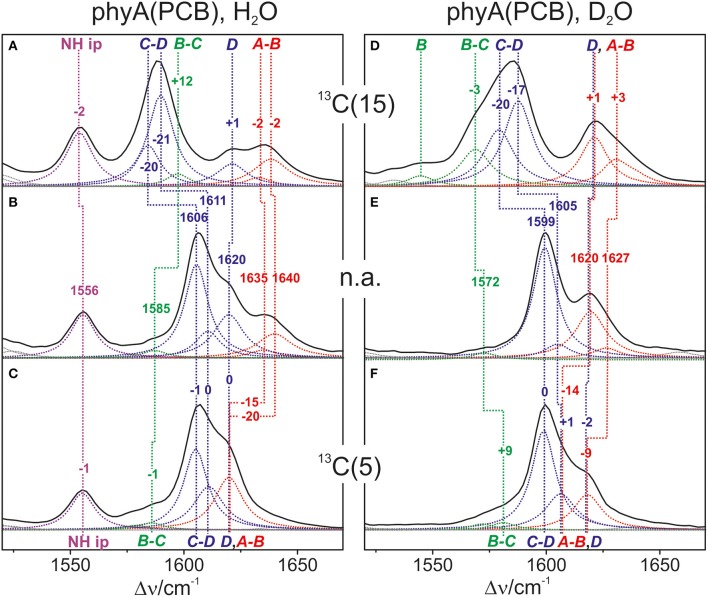
**Expanded view of the C = C stretching region of the RR spectra of the Pfr state of phyA(PCB), including the fitted band components**. The left **(A–C)** and right panel (**D**–**F**) refer to the spectra measured from samples in H_2_O and D_2_O, respectively. The spectra were obtained from phyA-PCB adducts including the non-labeled PCB (**B**,**E**; n.a., natural abundance) and the PCB chromophores ^13^C-labeled at position C(15) **(A,D)** and C(5) **(C,F)**. The main character of the individual modes is indicated by colored Lorentzian functions and abbreviations, i.e., *A*-*B* stretching (red); *C*-*D* and ring *D* C = C stretching (blue); *B*-*C* and ring *B* C = C stretching (green); N-H ip bending of rings *B* and *C* (magenta). The spectra were measured from buffered H_2_O or D_2_O solutions (pH 7.8 or pD 7.8) with 1064 nm excitation at -140°C. Further details are given in the text (Sections Resonance Raman Spectroscopy and Results). For the quality of the fits, see Supplementary Material Figure S1.

Also in the C = C stretching region we note remarkable changes between the spectra of the various PCB-binding phytochromes (Figure 3). However, the C = C stretching region is considerably more complex than the HOOP region as shown exemplarily for phyA (Figure 5). Band fitting analysis of the spectrum of the phyA adduct with non-labeled PCB required a minimum number of 7 bands in the region between 1540 and 1650 cm^−1^ (Figure 5B; Table 1). In contrast, quantum chemical calculations predict only five fundamentals in this region, which are dominated by the C = C stretching coordinates of the *A-B, B-C*, and *C-D* methine bridges, the C = C stretching of ring *D*, and the in-plane N-H bending coordinates of rings *B* and *C* (NH ip) (Supplementary Material). Thus, we expect distinct isotopic shifts upon comparing the RR spectra of non-labeled phyA-PCB with the PCB-adducts including specific ^13^C-labeling at the *A-B* and *C-D* methine bridges [^13^C(5), ^13^C(15), see Figure 1] and deuteration at the pyrrole nitrogens (Figure 5). Accordingly, the band at 1556 cm^−1^ is readily assigned to the NH ip mode (Figure 5B) since it remains nearly unchanged upon ^13^C-labeling at C(5) and C(15) (Figures 5A,C) but disappears upon H/D exchange of the N-H groups (Figures 5D–F). In agreement with previous experimental and theoretical studies (Mroginski et al., 2011b; Salewski et al., 2013), the most intense RR band at 1606 cm^−1^ (Figure 5B) of phyA-PCB is attributed to the C = C stretching of the *C-D* methine bridge. This band is accompanied by a somewhat weaker band on the high frequency side originating from the C = C stretching of ring *D*. This mode is insensitive to ^13^C-labeling at C(15) and C(5) whereas the *C-D* stretching should display a ca. −25 cm^−1^ shift upon ^13^C-labeling at C(15) as predicted by the calculations. Consequently, the invariant band at 1620 cm^−1^ is attributed to the C = C stretching of ring *D* whereas—in view of the ca. −20 cm^−1^ shifts in phyA-PCB-^13^C(15)—both the 1606 and the 1611 cm^−1^ appear to correspond to *C-D* stretching (Figures 5A,B). This assignment implies that these two modes originate from two PCB conformers that differ with respect to the structure of the *C-D* methine bridge, in line with the conclusions drawn from the analysis of the HOOP region (*vide supra*).

**Table 1:** **Band components in the C = C stretching region of the RR spectra of the Pfr state of phyA-PCB obtained by the fitting analysis^a^**.

**Mode^b^**	**n.a. H_2_O**		**^13^C(5) H_2_O**		**^13^C(15) H_2_O**		**n.a. D_2_O**		**^13^C(5) D_2_O**		**^13^C(15) D_2_O**	
	**ν /cm^−1^**	**I_rel_**	**ν /cm^−1^**	**I_rel_**	**ν /cm^−1^**	**I_rel_**	**ν /cm^−1^**	**I_rel_**	**ν /cm^−1^**	**I_rel_**	**ν /cm^−1^**	**I_rel_**
*AB*	1639.9	18			1638.4	21	1630.2^d^	11				
	1635.0	6	1619.8^c^	40	1632.9	7	1626.5^d^	8	1618.1^c^	27		
*D*^c^	1619.8	33			1621.1	17	1619.5	35			1621.1	28
*CD*	1610.5	20	1610.9	33	1584.2	32	1605.0	11	1598.8	76	1568.8	28
	1605.5	71	1605.1	62	1589.8	70	1599.4	84	1606.3	28	1587.7	48
*BC*			1585.7	5	1597.3	10			1581.1	6	1579.1	32
			1578.9	4					1571.9	5		
N-H ip	1555.5	33	1555.3	28	1554.0	41	–	–	–	–	–	–

a *Data refer to the unlabeled (n.a.) and ^13^C-labeled PCB bound to phyA in H_2_O and D_2_O*.

b *The main coordinate of the modes is indicated by AB, CD, BC, and D referring to the C = C stretching of the respective methane bridges and of ring D, and by NH ip, denoting the N-H in-plane bending of the rings B and C*.

c *Not resolved in terms of the two AB and the D band components*.

d *Not resolved in terms of the two AB band components*.

Above 1620 cm^−1^, the band-fitting analysis of the spectrum of non-labeled phyA-PCB (Figure 5B) reveals two further bands at 1640 cm^−1^ and, with rather low intensity, at 1635 cm^−1^. Both are marginally affected by ^13^C-labeling at C (15) but shift down in the PCB-^13^C(5) adduct (Figures 5A,C) such that they coincide with the C = C stretching of ring *D* to give a band envelope centered at 1620 cm^−1^.

H/D exchange at the pyrrole nitrogens affects not only the NH ip mode but also the methine bridge modes due to the admixture of small contributions of the N-H ip coordinates of the neighboring pyrrole rings. These shifts are expected to be <10 cm^−1^ for the *C-D* stretching but 10—15 cm^−1^ for the *A-B* and *B-C* stretching, as predicted by the present QM calculations (Supplementary Material) and observed in previous studies on the Pfr state of BV-binding phytochromes (Salewski et al., 2013). Indeed, our experimental findings accord with this (Figures 5D–F). Since the C = C stretching of ring D is not affected by D/H exchange, this band overlaps with those originating from the downshifted *A-B* stretchings in the spectra of the deuterated sample (Figures 5D,E). The additional downshift of the *A-B* stretchings upon ^13^C-labeling at C(5) then leads to the overlap with the non-shifted *C-D* stretching (Figure 5F). A summary of the assignments of the C = C stretching region is given in Table 1. Note that the correlation between modes of the non-labeled and labeled chromophore is an approximation. Each change of atomic masses (^13^C/^12^C; D/H) affects *all* solutions of the vibrational eigenvalue problem and thus frequencies, intensities and character (i.e., the potential energy distribution—PED) of *all* modes. Although the effects are particularly strong for modes dominated by coordinates of the label site, notable changes may also be observed for other modes that cannot be predicted by intuition. One instructive example refers to the B-C stretching which is known to be IR active but exhibits only low Raman activity (Schwinté et al., 2008) such that it can hardly be identified in the RR spectra. Previous IR studies have assigned this mode to a band between 1580 and 1590 cm^−1^ (Schwinté et al., 2008) and thus is attributed to the weak band at 1585 cm^−1^ in the spectrum of phyA-PCB (Figure 5B). In the spectrum of the deuterated PCB adduct ^13^C-labeled at position C(15) a distinct band at 1569 cm^−1^ is observed (Figure 5D) for which the *B-C* stretching is the only plausible assignment. Most likely, the intensity increase is due to an altered PED, presumably by a stronger contribution of the ring *B* C = C stretching coordinate.

Altogether the analysis of the C = C stretching region (Figure 5; Table 1) indicates a conformational heterogeneity of the PCB chromophore associated with sub-states differing with respect to the *C-D* and *A-B* methine bridges.

### Correlated spectral changes

**Figure 6:**
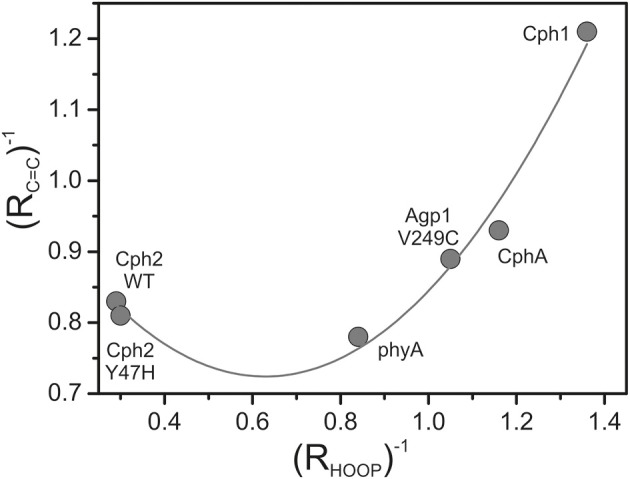
**Plot of the reciprocal intensity ratios of the high- to low-frequency components of the ***A***-***B*** stretching mode (***R_C=C_***) and the HOOP mode (***C***-***D*** methine bridge; ***R_HOOP_***), determined from the RR spectra of the Pfr states of various phytochromes by band fitting analyses**. The solid line represents the fit of a parabolic function to the data points.

The band-fitting analyses of the RR spectra in the C = C stretching region was extended to the Pfr states of all phytochromes studied in this work reflecting different distributions among the sub-states. Due to the strong overlap with the ring *D* C = C stretching, the intensity determination of the two conjugate *C-D* stretching modes is uncertain. Thus, we will restrict the discussion to the *A-B* stretching modes which are somewhat separated from the other modes in this region. As already shown by the spectra in Figure 3 (right panel), the intensity ratio of the high- and low-frequency *A-B* stretching component is different for the Pfr states of the various phytochromes, in analogy to the changes in the HOOP region (Figure 3, left panel). In fact, the intensity ratios *R_i_* of the HOOP and *A-B* stretching mode components as implied by band fitting are correlated (Figure 6), suggesting a coupling of the conformational differences at the *C-D* methine bridge (HOOP mode) and the *A-B* methine bridge (*A-B* stretching) that characterize the two apparent sub-states. Interestingly, a distinct coupling correlation is observed for the HOOP and C = C mode for Cph2 and its Y47H mutant compared to the other phytochromes. This is likely to be a result of the solvent exposure of the A-B ring moiety of the PCB chromophore that is caused by the lack of a shielding PAS domain present in the other studied phytochromes (Anders et al., 2013).

**Figure 7:**
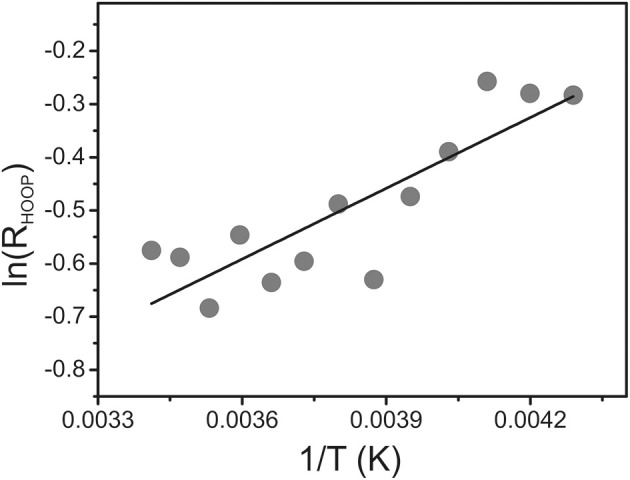
**van't Hoff plot of the intensity ratio of the high- to low-frequency components of the HOOP mode (***R_HOOP_***) of the Pfr state of Agp1-V249C determined from a band fitting of the spectra measured as a function of the temperature**. The solid line represents a linear fit to the experimental data.

To determine the energetic difference between the two sub-states we analyzed the temperature-dependence of the sub-state distribution on the basis of the HOOP mode components. Indeed, temperature-dependent measurements in the range between 293 and 233 K reveal small spectral changes, particularly in the HOOP region. However, in many cases spectral analysis was aggravated by the interference of temperature-dependent contributions of the Pr state. Thus, we have restricted the quantitative analysis to the spectra of Agp1-V249C which included the lowest and largely temperature-independent Pr contributions because the quantum efficiency of the Pfr to Pr photoconversion is extraordinarily low (Lamparter et al., 2002; Schumann et al.,^2008^). The intensity ratio of the HOOP modes of Pfr (*R_HOOP_*, high- to low-frequency), proportional to the equilibrium constant between the two sub-states, can be described by the van't Hoff equation (Figure 7) leading to an enthalpy difference between the two sub-states of 3.6 kJ.M^−1^.

### Time-resolved vibrational analysis: C = O stretching region

**Figure 8:**
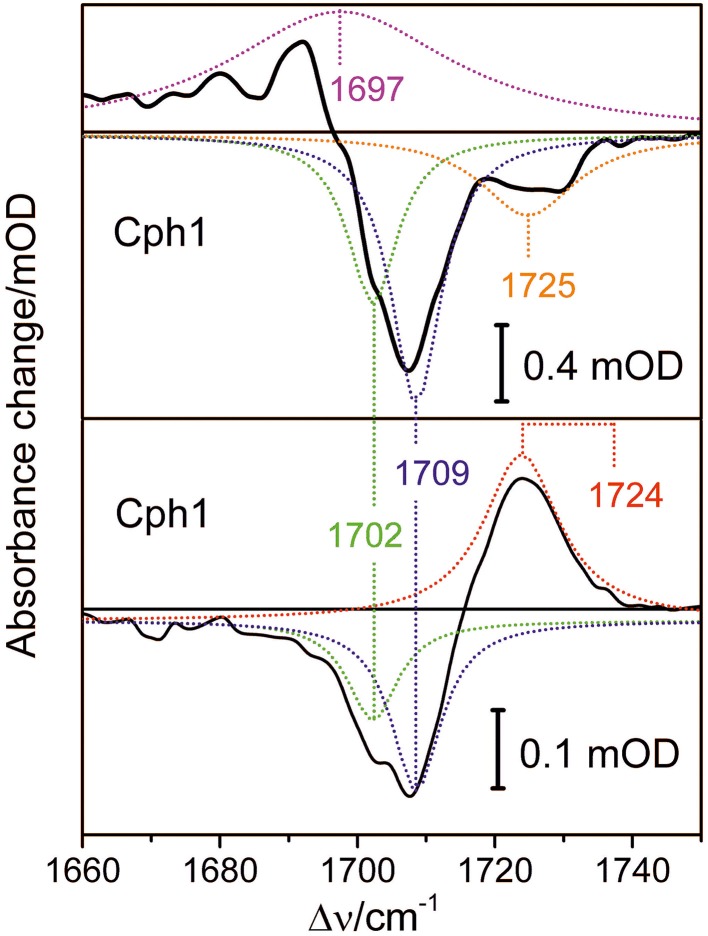
**Transient spectra of femtosecond time-resolved Vis pump IR probe experiments of the Pfr form of Cph1 upon excitation at 710 nm**. **Upper panel:** Decay associated spectrum calculated for delay time zero (black line), and simulated Lorentzian profiles (dotted lines); excited state contribution at 1697 cm^−1^ (magenta), bleaching signals at 1702 cm^−1^ (green), 1709 cm^−1^ (blue), and 1725 cm^−1^ (orange). **Lower panel:** Absorbance spectrum at 100 ps delay time, and simulated Lorentzian profiles (dotted lines); Lumi-F contribution at 1724 cm^−1^ (red), bleaching signals at 1702 cm^−1^ (green), and 1709 cm^−1^ (blue).

Conformational heterogeneity of the PCB chromophore with respect to the *C*-*D* and *A*-*B* methine bridges could be reflected by carbonyl stretching absorptions of ring *D* and ring *A*. We used femtosecond time-resolved IR spectroscopy to study the carbonyl bleaching bands of the unlabeled PCB chromophore upon Pfr photoexcitation within a ^13^C/^15^N-labeled Cph1 apoprotein. Upon excitation at 710 nm ultrafast absorption dynamics are displayed in the spectral range from 1660 cm^−1^ to 1750 cm^−1^ (Figure 8). Positive signals belong to excited state absorption, while negative (bleaching) signals around 1708 cm^−1^, and 1724 cm^−1^ are due to C(19) = O stretching vibrations, and C(1) = O stretching vibrations, respectively (Figure 8, upper panel) (Yang et al., 2012). The C(19) = O stretching vibration bleaching signal around 1708 cm^−1^ consists of two contributions at 1702 cm^−1^ and 1709 cm^−1^ simulated with band integrals of −12, and −20, respectively (Figure 8, upper panel). The C(1) = O stretching vibration bleaching signal exhibits a double peak feature with maxima at 1724 cm^−1^, and 1729 cm^−1^. However, due to low signal strength this feature is simulated with a single bleaching band at 1725 cm^−1^. At 100 ps delay time the initial Pfr photoreaction is finished and the remaining signals only consist of the negative bleaching signals and the positive photoproduct absorption signal of Lumi-F (Figure 8, lower panel). Since ring *A* is not involved in the primary photochemical process, no signals of the C(1) = O stretching vibration remain after photoisomerization. The C(19) = O stretching vibration signals around 1708 cm^−1^ can be assigned to two contributions at 1702 cm^−1^ and at 1709 cm^−1^ with simulated band integrals of −1.8, and −3.1, respectively. The positive Lumi-F signal is at 1724 cm^−1^.

The bleaching bands provide information on the ground state. The transient spectra demonstrate two closely-spaced bleaching bands of the C(19) = O stretching vibration at 1702 cm^−1^ and at 1709 cm^−1^ with relative intensity ratio of I_1702_/I_1709_ = 0.6. This assignment implies two modes originating from two PCB conformers in the Pfr state that differ with respect to the structure of the ring *D* carbonyl mode, in agreement with the conclusions drawn from the analysis of the HOOP and C = C stretching regions (*vide supra*).

### Phytochromobilin-binding phytochromes

**Figure 9:**
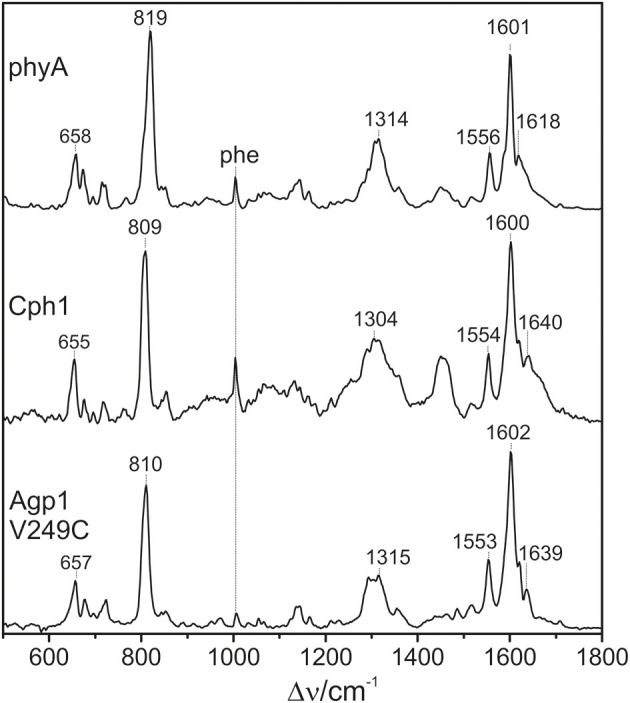
**Overview RR spectra of the Pfr states of phytochromes studied here including the PΦB chromophore**. The spectra were measured from buffered H_2_O solutions (pH 7.8) with 1064 nm excitation at −140°C. Further details are given in the text (Sections Resonance Raman Spectroscopy and Results).

All phytochromes studied in this work are able to attach PΦB at the same site as used for PCB. Previous comparative studies of oat phyA3 already demonstrated that the different ring *D* substituents (Figure 1) are associated with few spectral changes (Kneip et al., 1997; Remberg et al., 1997). It was of interest to determine whether the substituent affects the conformational heterogeneity of the chromophore. Focusing on the PΦB adducts of phyA, Cph1, and Agp1-V249C (Figure 9), spectral differences between the three phytochromes are noted in the entire spectral range including the HOOP and the C = C stretching region, as for PCB adducts.

**Figure 10:**
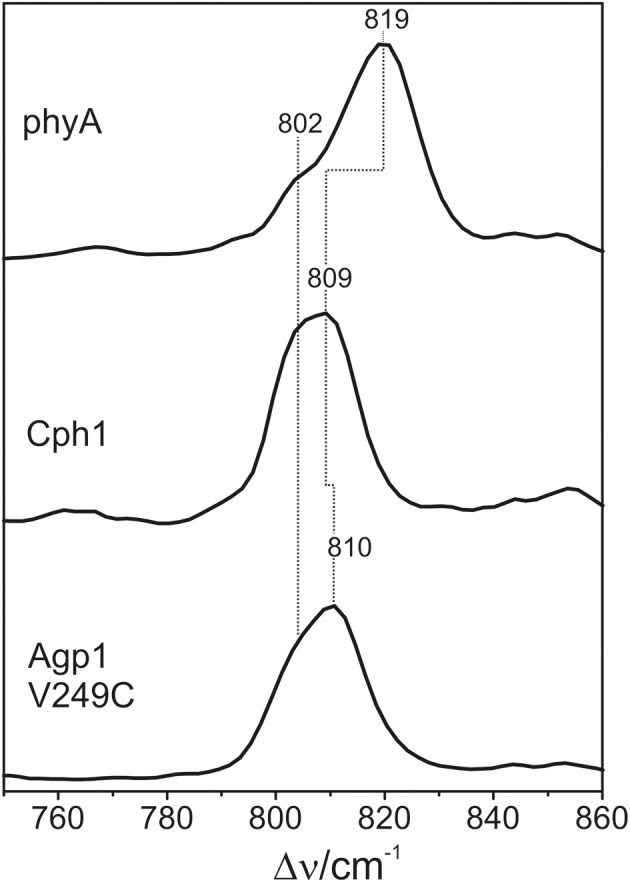
**Expanded view of the HOOP region of the RR spectra of the Pfr states of phytochromes studied here including the PΦB chromophore (see Figure 8)**. The spectra were measured from buffered H_2_O solutions (pH 7.8) with 1064 nm excitation at −140°C. Further details are given in the text (Sections Resonance Raman Spectroscopy and Results).

The HOOP region displays two overlapping bands with different relative intensities in the three spectra (Figure 10). These intensity variations are accompanied by shifts predominantly of the high-frequency component. In phyA(PΦB), the latter band clearly dominates, whereas both components are of similar intensities in the spectra of Cph1(PΦB) and Agp1-V249C(PΦB). As for PCB, quantum chemical calculations predict only one Raman-active mode in this region at 825 cm^−1^ that originates from the HOOP coordinate of the *C-D* methine bridge (Supplementary Material).

**Figure 11:**
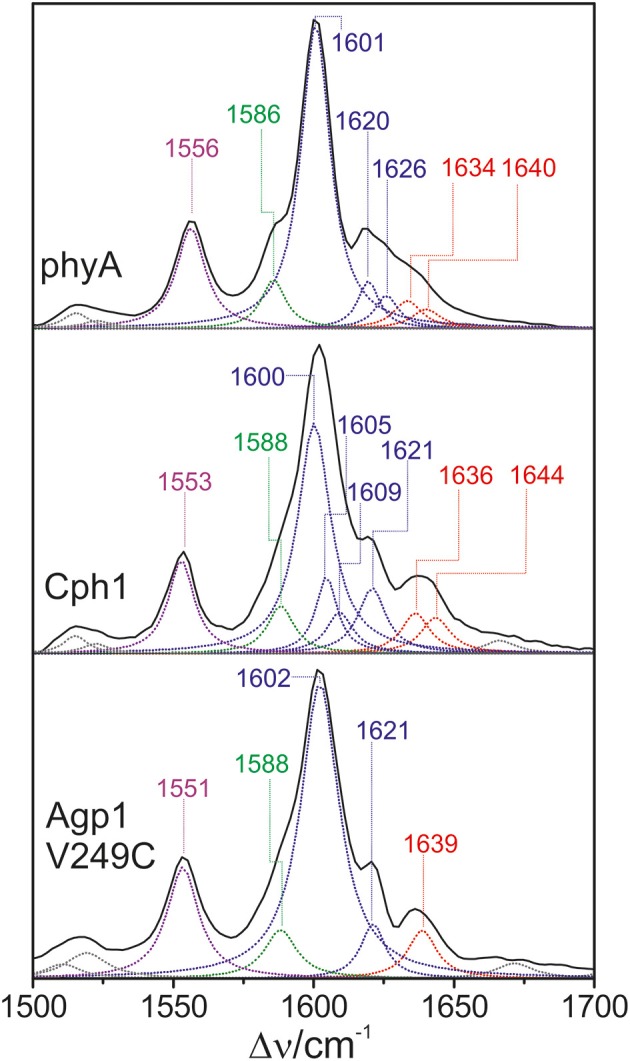
**Expanded view of the C = C stretching region (including fitted band components) of the RR spectra of the Pfr state of phytochromes studied here carrying the PΦB chromophore**. The main character of the individual modes is indicated by colored Lorentzian functions and abbreviations, i.e., *A*-*B* stretching (red); *C*-*D* and ring *D* C = C stretching (blue); *B*-*C* and ring *B* C = C stretching (green); N-H ip bending of rings *B* and *C* (magenta). The spectra were measured from buffered H_2_O solutions (pH 7.8) with 1064 nm excitation at −140°C. Further details are given in the text (Sections Resonance Raman Spectroscopy and Results). For the quality of the fits, see Supplementary Material Figure S1.

In the high-frequency region (Figure 11), the calculations predict again five modes between 1550 and 1650 cm^−1^ with similar mode composition as for PCB, except for the C = C stretching mode of ring *D* (Supplementary Material). For PΦB, this mode up-shifts to higher frequencies compared to the *C-D* stretching mode due to the admixture of the C = C stretching coordinate of the vinyl substituent. The assignment of the individual bands in this region otherwise follows the same scheme as that for the PCB adducts (Table 1). Accordingly, the *C*-*D* stretching corresponds to the strongest band which is found at essentially the same frequency in all three proteins (ca. 1600 cm^−1^; Figure 11). The band at ca. 1555 cm^−1^ is attributed to the N-H ip as it disappears upon H/D exchange (Kneip et al., 1999), whereas the weak band on the low frequency side of the *C*-*D* stretching is due to the *B*-*C* stretching, in line with previous IR spectroscopic data (Schwinté et al., 2008). On the high-frequency side of the *C-D* stretching, the number of bands that are resolved by band fitting differs for the three phytochromes. In phyA and Cph1 the two bands between 1634 and 1644 cm^−1^ are assigned to the A-B stretching in analogy to the spectra analysis of the PCB adducts (Figure 5), pointing to two sub-states with slightly different conformations of the *A-B* methine bridge. Thus, the remaining bands at 1620 and 1626 cm^−1^ in phyA and at 1605, 1609, and 1621 cm^−1^ in Cph1 (Figure 11) can in principle only be assigned to a second *C-D* stretching mode (in addition to the band at ca. 1600 cm^−1^) and to one or two ring *D* modes, pointing to conformational heterogeneity at the *C-D* bridge or ring *D*. In contrast, the spectrum of Agp1-V249C(PΦB) displays a different picture inasmuch as the total number of bands identified by the band-fitting analysis just agrees with the theoretically-predicted number of modes. Thus, the evident structural heterogeneity of the *C-D* methine bridge conformation as mirrored by the HOOP modes (Figure 10) has only marginally affects the C = C stretching region.

## Discussion

The present study has demonstrated that the Pfr states of a number of PCB- and PΦB-binding phytochromes display a structural heterogeneity of the chromophore involving two main sub-states, differing at the *A-B* and *C-D* methine bridges. The sub-states are populated to different extents in the phytochromes studied. The underlying structural differences are probably small since they affect only a few marker bands whereas most of the conjugate modes coincide.

### Structural differences between the sub-states

The structural differences associated with the *C*-*D* methine bridge are reflected by the HOOP, and the C = C stretching, and the C = O stretching mode. Inspection of Figure 3 shows that the intensity ratio of the high- to low-frequency HOOP component decreases in the order Cph2-WT < Cph2-Y47H < phyA < Agp1-V249C < CphA < Cph1. Unfortunately, the overlap of the C = C stretching mode of the ring *D* with those of the *C-D* methine bridge hampers reliable determination of the relative intensities of the latter modes even by band-fitting analyses. This uncertainty is particularly large for the two Cph2 variants in which the PAS domain is missing and the chromophores are partially exposed to the solvent as well as for Cph1. However, the remaining phytochromes display a tendency that can even be seen in Figure 3 inasmuch as the high frequency component of the *C-D* stretching increases in intensity according to phyA < Agp1-V249C < CphA. For these phytochromes, the low-frequency HOOP and the high-frequency *C-D* stretching component can readily be ascribed to one conformer, Pfr-I, whereas the high-frequency HOOP and the low-frequency *C-D* stretching component are attributed to the second conformer, Pfr-II. In view of the strong spectral similarities between phyA(PCB) and phyA(PΦB), this conclusion also holds for plant phytochrome A carrying its natural chromophore.

The coexistence of two conformers differing with respect to the HOOP and methine bridge modes is reminiscent of the results obtained for the Pfr states of BV-binding bacterial phytochromes shown previously (Salewski et al., 2013). In that case, quantum-mechanics/molecular-mechanics (QMMM) hybrid methods could be employed for a more profound analysis of spectra-structure relationships due to the availability of a well-resolved 3D structure of the bathy phytochrome PaBphP from *Pseudomonas aeruginosa* (Yang et al., 2008). The study demonstrated an inverse correlation of the HOOP frequency with the C(14)-C(15)-C(16)-N(*D*) dihedral angle, whereas the C = C stretching frequency was directly correlated with the N(*C*)-C(14)-C(15)-C(16) dihedral angle and inversely correlated with the C(15)-C(*D*) bond length (Salewski et al., 2013). An increase of both dihedral angles, as reflected by a downshift of the HOOP and an upshift of the C = C stretching mode, thus corresponds to an increased torsion of ring *D* with respect to ring *C*. Adopting the approximately linear relationship between the C = C stretching frequency and the dihedral angle with a slope of 0.65 degree/cm^−1^, as previously determined for the Pr state of phyA and Cph1 (Mroginski et al., 2011b), the torsional angle between rings *C* and *D* should be >10° larger in Pfr-I than in Pfr-II in the case of phyA.

An increased torsion of ring *D* with respect to ring *C* by about 10° permits formation of an additional hydrogen bond to ring *D*. Structural investigations on Cph1 demonstrated two possible hydrogen bonds on ring *D* between C(19) = O and Tyr263, and between N(*D*)-H and Asp207 (Song et al., 2013). Structural flexibility of the chromophore and ring *D* makes formation of one hydrogen bond more likely. Hydrogen bonds to carbonyl groups induce a red-shift of the frequency, as well as formation of hydrogen bonds to adjacent N-H groups. Thus, the conformer Pfr-I with a more twisted ring *D* is related with two hydrogen bonds on ring *D*, and conformer Pfr-II with one hydrogen bond on ring *D* in Cph1. This is supported by the intensity ratio Pfr-II / Pfr-I of 0.6 of the C(19) = O stretching vibrations at room temperature. It might be that the different twist angles in Pfr-I and Pfr-II predetermine the heterogeneous excited state dynamics of the Pfr state which in turn leads to two different reaction channels to the Pr state (Kim et al., 2014a). The recent CD spectroscopic analysis of algae phytochromes (Rockwell et al., 2014) is interesting in this context. That study revealed a structural heterogeneity of the Pfr chromophore, presumably associated with the rings *A* and *D*. The authors pointed out that similar CD signatures were also found in previous work on Cph1 (Borucki et al., 2003; Rockwell et al., 2009), implying similar conformational differences.

The structural differences between the two conformers also include the *A*-*B* methine bridge. For phyA, Agp1-V249C, and CphA the low-frequency component of the *A*-*B* stretching can be related to the low-frequency component of the HOOP mode (Figure 6) and thus attributed to Pfr-I. For BV-binding proteins, the frequency of the *A*-*B* stretching was found to be directly correlated with the C(5)-C(6)-N(*B*) and N(*B*)-C(9)-C(10) bond angles (Mroginski et al., 2011b) indicating that a decrease of these bond angles accompanies the increased twist around the *C*-*D* methine bridge.

For Cph2-WT, Cph2-Y47H, and Cph1 carrying the PCB chromophore as well as for the PΦB adducts of Agp1-V249C and Cph1, the correlation of the individual marker band components and their assignment to Pfr-I and Pfr-II is not unambiguous. Thus, it cannot be ruled out that in one conformer changes of the two dihedral angles of the *C*-*D* methine bridge partially compensate each other such that the net effect on the twist between the rings *C* and *D* is small.

### Transition between the conformational sub-states and chromophore dynamics

Temperature-dependent measurements of Agp1-V249C have revealed a reaction enthalpy of 3.6 kJ.M^−1^ for the transition from Pfr-I to Pfr-II and thus nearly a factor of 2 smaller than that determined for the same transition in the BV-binding Agp1-WT (Salewski et al., 2013). Intuitively, the reduction of the *C*-*D* methine bridge twist angle would likely be an exothermic process. However, a putative enthalpy gain due to the slight relaxation at this methine bridge may be overcompensated by concomitant structural changes which, in the case of Agp1-V249C, include a decrease of the bond angles between ring *B* and the neighboring methine bridges as discussed above. In addition, it might be that the decrease of the *C*-*D* methine bridge torsion additional also involves a loss of a hydrogen bond of ring *D* as suggested for Cph1(PCB) on the basis of time-resolved IR measurements (*vide supra*). In fact, hydrogen bond changes in the chromophore pocket might represent the energetically dominant process for the transition between Pfr-I and Pfr-II.

The present cryogenic studies do not provide any information about the kinetics of the transition but one may define a lower limit taking into account previous NMR spectroscopic results on Cph1(PCB) indicating a uniform and rigid chromophore structure (Song et al., 2013). However, conformers that interconvert faster than the magnetic relaxation times are not distinguishable. Thus, we conclude that the conformational heterogeneity detected in the static RR and time-resolved IR experiments for the Pfr state reflects a fast conformational dynamics of the tetrapyrrole which proceeds within nanoseconds or faster. More generally, this interpretation is in line with the conceptual view of correlating structural heterogeneities derived from static experiments with structural dynamics (Ren et al., 2013). Furthermore, the present results accord with previous evidence for chromophore heterogeneity (i.e., dynamics) from time-resolved spectroscopic studies (e.g., Schmidt et al., 1998; Sineshchekov et al., 1998; Sineshchekov, 2004; Kim et al., 2014a,b). However, it contrasts with the heterogeneity of the Pr state, that is slow enough to be resolved by NMR spectroscopy and is probably caused by changes of the chromophore and its environment on a larger scale than in Pfr, perhaps by solvent redistribution within the chromophore binding site.

### Chromophore structural dynamics and thermal back conversion

Among BV-binding bacteriophytochromes, only those with a resting Pr state show Pfr structural heterogeneity of the chromophore, proposed to be related to the capability of the chromophore to undergo a thermal isomerization and reversion to Pr (Salewski et al., 2013). It was suggested many years ago that thermal chromophore isomerization might require the transient formation of an enol form in the case of Pfr → Pr reversion in canonical phytochromes (Lagarias and Rapoport, 1980). In fact, this has recently been proven for the Pr → Pfr reversion in bathy phytochromes (Velazquez Escobar et al., 2015). However, in view of the slow Pfr → Pr dark reversion of the phytochromes studied in this work (with time constants in the order of hours), a detectable contribution of an enolic tetrapyrrole to the RR spectra is neither expected nor apparent.

Vierstra and coworkers have recently analyzed the effect of mutations in plant phytochromes on the dark reversion (Zhang et al., 2013). Although most of these experiments were based on Arabidopsis phyB, the results are likely to be relevant also for phyA and the phytochromes studied in this work since the mutations referred to conserved amino acids. As an example, the substitution of the positively-charged Arg352 (317 in oat phyA3, 254 in Cph1) by Ala slows down Pfr → Pr dark reversion, corresponding to a stabilization of the Pfr state. In view of the present results, this effect can be explained by the conformational dynamics of the *A-B* and *C-D* methine bridges. The salt bridge between Arg352 and the ring *B* propionate might fix that part of the chromophore to allow *D*-ring interactions to twist the *C*-*D* methine bridge, eventually leading to *E* → *Z* isomerization of the chromophore and the subsequent relaxation to the Pr state. Removal of the salt bridge in the Arg352Ala mutant might allow the *A*-*B* moiety to move, preventing the *C-D* torsion and thereby lowering the probability of *E* → *Z* isomerization. Similar explanations are possible for the effect of other substitutions in the chromophore pocket on thermal Pfr → Pr reversion.

In summary, we demonstrate that the chromophore in the Pfr states of canonical (PCB- and PΦB-binding) phytochromes displays a conformational heterogeneity associated with movements at the *A*-*B* and *C*-*D* methine bridges that may be functional for the thermal decay of the photoactivated state of the photosensor.

### Conflict of interest statement

The authors declare that the research was conducted in the absence of any commercial or financial relationships that could be construed as a potential conflict of interest.

## Supplementary material

Supplementary materials include a list of the DFT-based normal mode analyses of PCB and PΦB isotopomers (frequencies, Raman, and IR intensities, normal mode compositions) and a figure illustrating the goodness of the band fitting analyses (residuals).

## Abbreviations

BV: biliverdin
PCB: phycocyanobilin
PΦB: phytochromobilin
Agp1: Cph1, Cph2, CphA, and phyA refer to the photosensor modules of the various phytochromes studied in this work
Pr and Pfr denote the red- and far-red-absorbing states: respectively
*A-B*: *B-C*, and *C-D* denote the methine bridges between the respective pyrrole rings
CD: circular dichroism
RR: resonance Raman
IR: infrared
HOOP: hydrogen out-of-plane
NH ip: N-H in-plane bending.
