# Supplementary material for: Conformational heterogeneity of the Pfr chromophore in plant and cyanobacterial phytochromes
Source: Front Mol Biosci. 2015 Jul 10;2:37. doi: 10.3389/fmolb.2015.00037 (PMC4498102; doi:10.3389/fmolb.2015.00037)
Supplement: Supplementary file 1 [file DataSheet1.PDF]

Supplementary Material

Conformational heterogeneity of the Pfr chromophore in plant and cyanobacterial phytochromes

Francisco Velazquez Escobar[1a], David von Stetten[1,a,b], Mina Günther-Lütkens[1], Anke Keidel[1], Norbert Michael[1], Tilman Lamparter[2], Lars-O. Essen[3], Jon Hughes[4], Wolfgang Gärtner[5], Yang Yang[6], Karsten Heyne[6], Maria Andrea Mroginski[1], and Peter Hildebrandt[1*]

[1] Institut für Chemie, Technische Universität Berlin, Berlin, Germany
[2] Botanisches Institut, Karlsruher Institut für Technologie, Karlsruhe, Germany.
[3] Fachbereich Chemie, Philipps-Universität Marburg, Marburg, Germany
[4] Institut für Pflanzenphysiologie, Justus Liebig University, Gießen, Germany.
[5] Max-Planck-Institut für Chemische Energiekonversion, Mülheim, Germany.
[6] Institut für Experimentalphysik, Freie Universität Berlin, Berlin, Germany

[a] equal contribution to the work
[b] Present address: Structural Biology Group, European Synchrotron Radiation Facility, CS 40220, F-38043 Grenoble Cedex 9, France

[*] **Correspondence:** Professor Peter Hildebrandt, Technische Universität Berlin, Institut für Chemie, Sekr. PC14, Straße des 17. Juni 135, D-10623 Berlin, Germany, Hildebrandt@chem.tu-berlin.de

Content:

1. **Supplementary Figure: band fitting analyses of experimental RR spectra including the residuals of the overall fits.**

2. **Supplementary data: normal mode analyses of PCB and PΦB**

1. Supplementary Figure

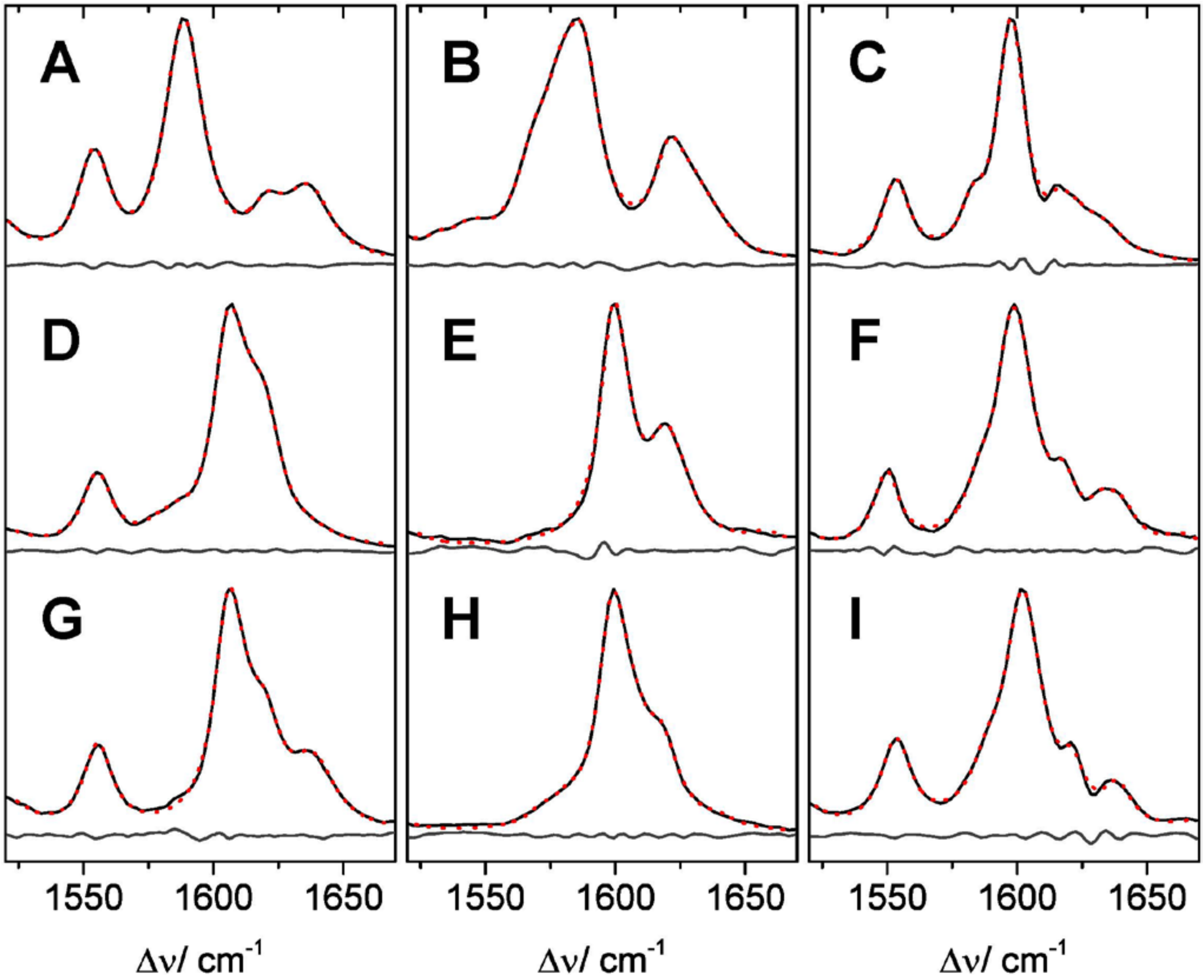

Figure S1. RR spectra of Pfr (solid black) of various phytochromes assembled with PCB (first and second column) and with PΦB (third column). The overall fits (red) and residual (dark gray) are also shown. First column: spectra of phyA in H_2O assembled with (A) PCB-^{13}C(15), (D) PCB n.a., and (G) PCB-^{13}C(5) (G). Second column: corresponding spectra in D_2O (B, E, and H). Third column: spectra of (C) phyA, (F) Cph1, and (I) Agp1V249C assembled with PΦB, all measured in H_2O.

2. Supplementary Data

The following data comprise the complete lists of calculated normal modes of phycocyanobilin (PCB) including various isotopomers, and a selection of calculated normal modes of phytochromobilin (PΦB). The normal modes were calculated on the basis of density functional theory for the free, fully protonated tetrapyrrole *in vacuo* in the *ZZEssa* configuration. The calculations were carried out as described previously (see Mroginski, M. A., von Stetten, D., Kaminski, S., Velazquez Escobar, F., Michael, N., Daminelli-Widany, G., et al. (2011a). Elucidating photoinduced structural changes in phytochromes by the combined application of resonance Raman spectroscopy and theoretical methods. J. Mol. Struct. 993, 15-25., and references therein). Listed are the calculated frequencies, IR and Raman intensities, as well as the potential energy distribution (PED) in terms of contributions of the internal coordinates.

Normal modes of PCB in the ZZEssa configuration

ZZEssa n.a. H_2O

	calc. [cm-1]	exp. [cm-1]	IRint [km/mol]	Rint (rel.)	contribution
1	3522.77	-------	55.00	0.17	(100.07%) (67) ring C-prop: O-H stretch (27,84)
2	3516.59	-------	55.88	0.20	(100.07%) (39) ring B-prop: O-H stretch (85,83)
3	3489.04	-------	55.56	0.14	(99.72%) (69) ring D: N-H stretch (29,28)
4	3122.35	-------	773.95	0.11	(38.09%) (1) ring A: N-H stretch (47,46)
					(33.25%) (20) ring B: N-H stretch (66,65)
					(28.34%) (47) ring C: N-H stretch (10, 9)
5	3067.41	-------	0.99	0.20	(99.72%) (42) bridge BC: C-H stretch (4, 3)
6	3056.79	-------	28.83	0.32	(99.48%) (46) bridge AB: C-H stretch (8, 7)
7	3037.34	-------	525.04	0.51	(58.42%) (1) ring A: N-H stretch (47,46)
					(9.21%) (20) ring B: N-H stretch (66,65)
					(32.03%) (47) ring C: N-H stretch (10, 9)
8	3015.81	-------	170.69	0.11	(55.16%) (20) ring B: N-H stretch (66,65)
					(5.30%) (43) bridge CD: C-H stretch (6, 5)
					(36.24%) (47) ring C: N-H stretch (10, 9)
9	3013.48	-------	16.14	0.10	(48.52%) (56) ring C-methyl: C-H stretch (16,15)
					(45.36%) (57) ring C-methyl: C-H stretch (17,15)
10	3012.89	-------	9.82	0.13	(79.22%) (78) ring D-methyl: C-H stretch (35,34)
					(5.50%) (79) ring D-methyl: C-H stretch (36,34)
					(12.74%) (80) ring D-methyl: C-H stretch (37,34)
11	3011.72	-------	4.80	0.06	(91.38%) (43) bridge CD: C-H stretch (6, 5)
12	3006.08	-------	18.88	0.09	(9.15%) (85) ring D-ethyl: C-H stretch (42,41)
					(11.25%) (86) ring D-ethyl: C-H stretch (43,41)
					(75.62%) (87) ring D-ethyl: C-H stretch (44,41)
13	3003.59	-------	10.08	0.10	(16.39%) (10) ring A-methyl: C-H stretch (55,54)
					(6.14%) (11) ring A-methyl: C-H stretch (56,54)
					(77.33%) (12) ring A-methyl: C-H stretch (57,54)
14	3000.91	-------	15.42	0.04	(6.78%) (60) ring C-prop: C-H stretch (20,19)
					(71.63%) (61) ring C-prop: C-H stretch (21,19)
					(19.66%) (63) ring C-prop: C-H stretch (23,22)
15	2998.99	-------	34.90	0.14	(13.35%) (27) ring B-methyl: C-H stretch (72,71)
					(7.80%) (31) ring B-prop: C-H stretch (76,75)
					(9.98%) (32) ring B-prop: C-H stretch (77,75)
					(60.03%) (34) ring B-prop: C-H stretch (79,78)
					(7.03%) (35) ring B-prop: C-H stretch (80,78)
16	2996.58	-------	11.72	0.12	(73.54%) (27) ring B-methyl: C-H stretch (72,71)
					(5.57%) (29) ring B-methyl: C-H stretch (74,71)
					(13.75%) (34) ring B-prop: C-H stretch (79,78)
17	2987.25	-------	27.14	0.19	(31.88%) (17) ring A-ethyl: C-H stretch (62,60)
					(58.84%) (18) ring A-ethyl: C-H stretch (63,60)
18	2985.50	-------	50.79	0.47	(51.26%) (85) ring D-ethyl: C-H stretch (42,41)
					(45.14%) (86) ring D-ethyl: C-H stretch (43,41)
19	2984.68	-------	0.97	0.07	(18.40%) (61) ring C-prop: C-H stretch (21,19)
					(70.81%) (63) ring C-prop: C-H stretch (23,22)
					(7.85%) (64) ring C-prop: C-H stretch (24,22)
20	2984.25	-------	46.14	0.18	(45.83%) (10) ring A-methyl: C-H stretch (55,54)
					(43.17%) (11) ring A-methyl: C-H stretch (56,54)
					(5.35%) (18) ring A-ethyl: C-H stretch (63,60)
21	2981.34	-------	8.49	0.07	(8.45%) (56) ring C-methyl: C-H stretch (16,15)
					(10.06%) (57) ring C-methyl: C-H stretch (17,15)
					(8.87%) (58) ring C-methyl: C-H stretch (18,15)
					(36.10%) (79) ring D-methyl: C-H stretch (36,34)
					(35.98%) (80) ring D-methyl: C-H stretch (37,34)
22	2979.96	-------	8.81	0.15	(27.75%) (56) ring C-methyl: C-H stretch (16,15)
					(27.06%) (57) ring C-methyl: C-H stretch (17,15)
					(17.96%) (58) ring C-methyl: C-H stretch (18,15)
					(12.94%) (79) ring D-methyl: C-H stretch (36,34)
					(13.89%) (80) ring D-methyl: C-H stretch (37,34)
23	2979.83	-------	34.09	0.09	(5.95%) (14) ring A-ethyl: C-H stretch (59,58)
					(46.58%) (16) ring A-ethyl: C-H stretch (61,60)
					(28.82%) (17) ring A-ethyl: C-H stretch (62,60)
					(11.05%) (18) ring A-ethyl: C-H stretch (63,60)
					(6.24%) (40) ring A-ethyl: C-H stretch (1,58)

24	2977.85	-------	4.04	0.05	(46.43%) (31) ring B-prop: C-H stretch (76,75)
					(35.13%) (32) ring B-prop: C-H stretch (77,75)
					(16.50%) (34) ring B-prop: C-H stretch (79,78)
25	2956.12	-------	10.76	0.10	(56.79%) (82) ring D-ethyl: C-H stretch (39,38)
					(36.28%) (83) ring D-ethyl: C-H stretch (40,38)
26	2954.93	-------	14.59	0.18	(48.16%) (28) ring B-methyl: C-H stretch (73,71)
					(51.99%) (29) ring B-methyl: C-H stretch (74,71)
27	2949.87	-------	6.14	0.10	(52.31%) (14) ring A-ethyl: C-H stretch (59,58)
					(9.42%) (17) ring A-ethyl: C-H stretch (62,60)
					(28.61%) (40) ring A-ethyl: C-H stretch (1,58)
28	2947.12	-------	19.87	0.22	(43.73%) (31) ring B-prop: C-H stretch (76,75)
					(52.09%) (32) ring B-prop: C-H stretch (77,75)
29	2941.13	-------	8.02	0.23	(93.98%) (4) ring A: C-H stretch (50,49)
30	2936.43	-------	17.54	0.34	(75.58%) (60) ring C-prop: C-H stretch (20,19)
					(8.49%) (61) ring C-prop: C-H stretch (21,19)
					(13.79%) (64) ring C-prop: C-H stretch (24,22)
31	2930.97	-------	24.67	0.50	(17.31%) (78) ring D-methyl: C-H stretch (35,34)
					(43.16%) (79) ring D-methyl: C-H stretch (36,34)
					(36.55%) (80) ring D-methyl: C-H stretch (37,34)
32	2928.56	-------	2.64	0.16	(8.79%) (34) ring B-prop: C-H stretch (79,78)
					(87.42%) (35) ring B-prop: C-H stretch (80,78)
33	2925.03	-------	11.13	0.11	(14.88%) (60) ring C-prop: C-H stretch (20,19)
					(7.17%) (63) ring C-prop: C-H stretch (23,22)
					(76.99%) (64) ring C-prop: C-H stretch (24,22)
34	2922.01	-------	46.00	0.30	(38.42%) (85) ring D-ethyl: C-H stretch (42,41)
					(38.29%) (86) ring D-ethyl: C-H stretch (43,41)
					(19.07%) (87) ring D-ethyl: C-H stretch (44,41)
35	2921.78	-------	72.98	0.41	(29.54%) (6) ring A: C-H stretch (52,51)
					(15.85%) (10) ring A-methyl: C-H stretch (55,54)
					(23.80%) (11) ring A-methyl: C-H stretch (56,54)
					(10.89%) (12) ring A-methyl: C-H stretch (57,54)
					(9.76%) (14) ring A-ethyl: C-H stretch (59,58)
36	2919.15	-------	2.49	0.04	(16.34%) (6) ring A: C-H stretch (52,51)
					(15.21%) (10) ring A-methyl: C-H stretch (55,54)
					(21.31%) (11) ring A-methyl: C-H stretch (56,54)
					(8.74%) (12) ring A-methyl: C-H stretch (57,54)
					(15.12%) (14) ring A-ethyl: C-H stretch (59,58)
					(14.43%) (40) ring A-ethyl: C-H stretch (1,58)
37	2918.33	-------	21.76	0.26	(37.09%) (82) ring D-ethyl: C-H stretch (39,38)
					(57.06%) (83) ring D-ethyl: C-H stretch (40,38)
38	2917.20	-------	41.64	0.32	(11.68%) (56) ring C-methyl: C-H stretch (16,15)
					(13.90%) (57) ring C-methyl: C-H stretch (17,15)
					(70.90%) (58) ring C-methyl: C-H stretch (18,15)
39	2915.75	-------	27.18	0.12	(9.86%) (6) ring A: C-H stretch (52,51)
					(41.08%) (16) ring A-ethyl: C-H stretch (61,60)
					(26.18%) (17) ring A-ethyl: C-H stretch (62,60)
					(18.90%) (18) ring A-ethyl: C-H stretch (63,60)
40	2911.28	-------	6.29	0.23	(39.40%) (6) ring A: C-H stretch (52,51)
					(11.35%) (14) ring A-ethyl: C-H stretch (59,58)
					(44.71%) (40) ring A-ethyl: C-H stretch (1,58)
41	2909.71	-------	45.41	0.52	(12.34%) (27) ring B-methyl: C-H stretch (72,71)
					(45.86%) (28) ring B-methyl: C-H stretch (73,71)
					(41.10%) (29) ring B-methyl: C-H stretch (74,71)
42	1783.90	-------	290.69	1.55	(84.17%) (19) ring A: C=O stretch (64,48)
					(5.18%) (88) ring A bending
43	1774.15	-------	191.14	0.05	(81.29%) (66) ring C-prop: C=O stretch (26,25)
					(6.41%) (199) ring C-prop: C-O-H BEND
44	1760.70	-------	253.97	0.02	(81.10%) (37) ring B-prop: C=O stretch (82,81)
					(6.53%) (161) ring B-prop: C-O-H BEND
45	1741.05	-------	907.89	4.36	(80.45%) (74) ring D: C=O stretch (33,45)
					(5.13%) (100) ring D bending
46	1620.32	-------	85.50	100.00	(7.23%) (52) bridge CD: C-C stretch (14, 5)
					(55.22%) (70) bridge CD: C=C stretch (30, 5)
					(5.60%) (71) ring D: C-N stretch (30,28)
					(9.66%) (166) bridge CD: C-H ROCK
47	1618.77	-------	610.98	45.91	(5.14%) (7) ring A: C-N stretch (53,46)
					(15.97%) (41) bridge BC: C-.C stretch (3,70)
					(22.68%) (44) bridge AB: C=C stretch (7,53)
					(12.70%) (48) bridge BC: C-.C stretch (11, 3)

					(7.78%)	(163)	bridge BC: C-H ROCK
48	1613.55	-------	27.58	3.57	(69.22%)	(73)	ring D: C=C stretch (32,31)
					(6.16%)	(77)	ring D-methyl: C-C stretch (34,31)
					(8.28%)	(81)	ring D-ethyl: C-C stretch (38,32)
49	1592.51	-------	1257.69	3.31	(19.52%)	(23)	ring B: C-.C stretch (69,68)
					(7.82%)	(41)	bridge BC: C-.C stretch (3,70)
					(18.27%)	(44)	bridge AB: C=C stretch (7,53)
					(9.09%)	(48)	bridge BC: C-.C stretch (11, 3)
					(8.19%)	(163)	bridge BC: C-H ROCK
50	1557.22	-------	350.25	20.70	(8.16%)	(21)	ring B: C-N stretch (67,65)
					(7.03%)	(45)	bridge AB: C-C stretch (7,67)
					(5.29%)	(48)	bridge BC: C-.C stretch (11, 3)
					(27.36%)	(133)	ring B: N-H ROCK
					(21.89%)	(171)	ring C: N-H ROCK
51	1542.19	-------	1118.64	7.49	(27.42%)	(23)	ring B: C-.C stretch (69,68)
					(5.56%)	(26)	ring B-methyl: C-C stretch (71,68)
					(7.96%)	(41)	bridge BC: C-.C stretch (3,70)
					(8.38%)	(44)	bridge AB: C=C stretch (7,53)
					(5.75%)	(92)	ring B bending
52	1520.66	-------	65.31	8.71	(6.91%)	(7)	ring A: C-N stretch (53,46)
					(7.28%)	(45)	bridge AB: C-C stretch (7,67)
					(6.59%)	(54)	ring C: C-C stretch (14,13)
					(5.26%)	(104)	ring A: N-H ROCK
					(9.47%)	(133)	ring B: N-H ROCK
					(18.01%)	(171)	ring C: N-H ROCK
53	1506.23	-------	207.12	4.29	(37.34%)	(51)	ring C: C-.C stretch (13,12)
					(10.87%)	(53)	ring C: C-N stretch (14, 9)
					(8.07%)	(55)	ring C-methyl: C-C stretch (15,13)
54	1481.71	-------	14.72	0.17	(62.09%)	(182)	ring C-methyl: ADEFa
					(5.59%)	(184)	ring C-methyl: ROCKa
					(5.32%)	(211)	ring D-methyl: ADEFa
55	1477.19	-------	7.13	0.17	(5.85%)	(216)	ring D-ethyl: CH2 SCIS
					(71.41%)	(221)	ring D-ethyl: CH3 ADEFa
					(10.22%)	(222)	ring D-ethyl: CH3 ADEFb
					(6.60%)	(223)	ring D-ethyl: CH3 ROCKa
56	1477.14	-------	58.61	0.12	(9.34%)	(145)	ring B-methyl: ADEFb
					(69.90%)	(149)	ring B-prop: 1-CH2-SCIS
57	1473.57	-------	5.03	0.06	(14.27%)	(124)	ring A-ethyl: CH2 SCIS
					(44.31%)	(129)	ring A-ethyl: CH3 ADEFa
					(25.76%)	(130)	ring A-ethyl: CH3 ADEFb
58	1471.64	-------	5.06	4.20	(10.20%)	(7)	ring A: C-N stretch (53,46)
					(8.25%)	(133)	ring B: N-H ROCK
					(5.64%)	(144)	ring B-methyl: ADEFa
					(24.02%)	(169)	bridge AB: C-H ROCK
					(5.23%)	(182)	ring C-methyl: ADEFa
59	1468.65	-------	4.60	0.13	(20.90%)	(119)	ring A-methyl: ADEFa
					(38.97%)	(129)	ring A-ethyl: CH3 ADEFa
					(25.39%)	(130)	ring A-ethyl: CH3 ADEFb
60	1467.21	-------	11.57	0.21	(30.82%)	(119)	ring A-methyl: ADEFa
					(31.72%)	(120)	ring A-methyl: ADEFb
					(5.09%)	(124)	ring A-ethyl: CH2 SCIS
					(20.58%)	(130)	ring A-ethyl: CH3 ADEFb
61	1465.24	-------	2.81	0.06	(37.65%)	(183)	ring C-methyl: ADEFb
					(12.53%)	(187)	ring C-prop: 1-CH2-SCIS
					(29.53%)	(212)	ring D-methyl: ADEFb
62	1465.11	-------	3.22	0.16	(33.90%)	(119)	ring A-methyl: ADEFa
					(49.02%)	(120)	ring A-methyl: ADEFb
63	1463.92	-------	11.74	0.22	(6.59%)	(221)	ring D-ethyl: CH3 ADEFa
					(77.62%)	(222)	ring D-ethyl: CH3 ADEFb
					(7.16%)	(224)	ring D-ethyl: CH3 ROCKb
64	1459.10	-------	62.81	0.36	(14.77%)	(144)	ring B-methyl: ADEFa
					(25.80%)	(145)	ring B-methyl: ADEFb
					(7.24%)	(149)	ring B-prop: 1-CH2-SCIS
					(11.83%)	(187)	ring C-prop: 1-CH2-SCIS
					(7.15%)	(212)	ring D-methyl: ADEFb
65	1457.01	-------	8.82	0.48	(13.40%)	(144)	ring B-methyl: ADEFa
					(6.11%)	(145)	ring B-methyl: ADEFb
					(23.09%)	(187)	ring C-prop: 1-CH2-SCIS
					(28.11%)	(212)	ring D-methyl: ADEFb
66	1454.71	-------	57.22	1.04	(67.79%)	(211)	ring D-methyl: ADEFa
					(6.33%)	(213)	ring D-methyl: ROCKa
67	1454.20	-------	10.21	0.17	(71.86%)	(124)	ring A-ethyl: CH2 SCIS
					(14.13%)	(130)	ring A-ethyl: CH3 ADEFb

68	1453.20	-------	11.16	0.57	(44.41%)	(144)	ring B-methyl: ADEFa
					(18.83%)	(145)	ring B-methyl: ADEFb
					(13.22%)	(183)	ring C-methyl: ADEFb
69	1452.69	-------	13.74	0.34	(6.57%)	(144)	ring B-methyl: ADEFa
					(17.01%)	(145)	ring B-methyl: ADEFb
					(26.97%)	(183)	ring C-methyl: ADEFb
					(26.51%)	(187)	ring C-prop: 1-CH2-SCIS
					(6.09%)	(212)	ring D-methyl: ADEFb
70	1444.97	-------	2.09	0.33	(75.78%)	(216)	ring D-ethyl: CH2 SCIS
					(9.59%)	(221)	ring D-ethyl: CH3 ADEFa
71	1441.43	-------	12.56	7.27	(9.89%)	(21)	ring B: C-N stretch (67,65)
					(6.04%)	(23)	ring B: C-.C stretch (69,68)
					(5.56%)	(41)	bridge BC: C-.C stretch (3,70)
					(10.72%)	(169)	bridge AB: C-H ROCK
72	1438.80	-------	24.31	0.37	(72.21%)	(154)	ring B-prop: 2-CH2-SCIS
					(6.86%)	(163)	bridge BC: C-H ROCK
73	1434.24	-------	19.50	2.04	(7.19%)	(49)	ring C: C-N stretch (11, 9)
					(6.48%)	(149)	ring B-prop: 1-CH2-SCIS
					(19.20%)	(154)	ring B-prop: 2-CH2-SCIS
					(16.73%)	(163)	bridge BC: C-H ROCK
					(6.04%)	(187)	ring C-prop: 1-CH2-SCIS
74	1424.31	-------	48.00	0.34	(79.73%)	(192)	ring C-prop: 2-CH2-SCIS
75	1414.51	-------	30.53	2.14	(5.21%)	(24)	ring B: C-N stretch (70,65)
					(5.67%)	(25)	ring B: C-C stretch (70,69)
					(7.17%)	(50)	ring C: C-C stretch (12,11)
					(6.81%)	(59)	ring C-prop: C-C stretch (19,12)
					(6.65%)	(97)	ring C bending
					(5.31%)	(182)	ring C-methyl: ADEFa
					(7.01%)	(192)	ring C-prop: 2-CH2-SCIS
76	1407.56	-------	217.75	13.18	(11.18%)	(25)	ring B: C-C stretch (70,69)
					(6.03%)	(30)	ring B-prop: C-C stretch (75,69)
					(5.57%)	(93)	ring B bending
					(7.20%)	(143)	ring B-methyl: SDEF
					(19.01%)	(166)	bridge CD: C-H ROCK
77	1399.56	-------	40.14	0.95	(8.62%)	(65)	ring C-prop: C-C stretch (25,22)
					(8.65%)	(68)	ring C-prop: C-O stretch (27,25)
					(5.57%)	(190)	ring C-prop: 1-CH2-TWIST
					(8.86%)	(192)	ring C-prop: 2-CH2-SCIS
					(28.89%)	(194)	ring C-prop: 2-CH2-WAGG
					(7.99%)	(197)	ring C-prop: C=O ROCK
					(9.40%)	(199)	ring C-prop: C-O-H BEND
78	1395.41	-------	63.98	0.19	(8.66%)	(36)	ring B-prop: C-C stretch (81,78)
					(10.88%)	(38)	ring B-prop: C-O stretch (83,81)
					(20.89%)	(156)	ring B-prop: 2-CH2-WAGG
					(9.41%)	(159)	ring B-prop: C=O ROCK
					(13.41%)	(161)	ring B-prop: C-O-H BEND
79	1394.34	-------	54.60	1.65	(5.76%)	(50)	ring C: C-C stretch (12,11)
					(12.19%)	(143)	ring B-methyl: SDEF
					(5.18%)	(156)	ring B-prop: 2-CH2-WAGG
					(5.28%)	(181)	ring C-methyl: SDEF
80	1389.34	-------	10.22	0.28	(10.70%)	(181)	ring C-methyl: SDEF
					(67.38%)	(210)	ring D-methyl: SDEF
81	1384.75	-------	4.88	0.71	(5.10%)	(128)	ring A-ethyl: CH3 SDEF
					(5.82%)	(143)	ring B-methyl: SDEF
					(67.50%)	(181)	ring C-methyl: SDEF
					(7.40%)	(210)	ring D-methyl: SDEF
82	1384.17	-------	1.95	0.06	(87.01%)	(128)	ring A-ethyl: CH3 SDEF
83	1380.70	-------	33.10	1.89	(11.53%)	(104)	ring A: N-H ROCK
					(12.41%)	(118)	ring A-methyl: SDEF
					(42.06%)	(143)	ring B-methyl: SDEF
84	1376.59	-------	5.71	1.32	(8.27%)	(104)	ring A: N-H ROCK
					(32.91%)	(118)	ring A-methyl: SDEF
					(20.14%)	(143)	ring B-methyl: SDEF
85	1373.32	-------	28.39	1.48	(6.53%)	(104)	ring A: N-H ROCK
					(45.70%)	(118)	ring A-methyl: SDEF
86	1369.85	-------	3.78	0.16	(79.34%)	(220)	ring D-ethyl: CH3 SDEF
87	1367.58	-------	27.00	4.55	(7.85%)	(44)	bridge AB: C=C stretch (7,53)
					(31.60%)	(104)	ring A: N-H ROCK
88	1352.49	-------	5.70	0.55	(17.53%)	(112)	ring A-ethyl: SCIS
					(57.28%)	(126)	ring A-ethyl: CH2 WAGG
89	1348.07	-------	23.00	5.77	(9.12%)	(81)	ring D-ethyl: C-C stretch (38,32)

					(11.31%) (101) ring D bending<br>(6.55%) (210) ring D-methyl: SDEF<br>(23.79%) (218) ring D-ethyl: CH2 WAGG<br>(5.32%) (220) ring D-ethyl: CH3 SDEF
90	1340.57	-------	18.94	0.57	(12.39%) (108) ring A-methyl: SCIS<br>(8.48%) (112) ring A-ethyl: SCIS<br>(6.48%) (114) ring A-ethyl: WAGG<br>(8.57%) (126) ring A-ethyl: CH2 WAGG<br>(8.57%) (127) ring A-ethyl: CH2 TWIST<br>(8.35%) (151) ring B-prop: 1-CH2-WAGG<br>(10.42%) (189) ring C-prop: 1-CH2-WAGG
91	1340.34	-------	20.73	2.02	(50.72%) (189) ring C-prop: 1-CH2-WAGG<br>(7.24%) (195) ring C-prop: 2-CH2-TWIST
92	1337.09	-------	12.22	0.23	(45.72%) (151) ring B-prop: 1-CH2-WAGG<br>(10.18%) (156) ring B-prop: 2-CH2-WAGG<br>(9.52%) (161) ring B-prop: C-O-H BEND
93	1332.69	-------	38.37	12.41	(9.81%) (22) ring B: C-C stretch (68,67)<br>(6.65%) (41) bridge BC: C-.C stretch (3,70)<br>(10.21%) (45) bridge AB: C-C stretch (7,67)<br>(24.36%) (133) ring B: N-H ROCK
94	1328.77	-------	35.90	25.16	(17.15%) (71) ring D: C-N stretch (30,28)<br>(15.51%) (200) ring D: N-H ROCK<br>(12.84%) (218) ring D-ethyl: CH2 WAGG
95	1319.00	-------	13.29	2.75	(10.87%) (76) ring D: C-C stretch (33,32)<br>(6.08%) (101) ring D bending<br>(40.95%) (218) ring D-ethyl: CH2 WAGG<br>(10.51%) (219) ring D-ethyl: CH2 TWIST
96	1314.97	-------	54.41	1.30	(11.47%) (190) ring C-prop: 1-CH2-TWIST<br>(21.66%) (194) ring C-prop: 2-CH2-WAGG<br>(5.28%) (197) ring C-prop: C=O ROCK<br>(35.39%) (199) ring C-prop: C-O-H BEND
97	1307.59	-------	26.40	0.60	(31.53%) (108) ring A-methyl: SCIS<br>(5.65%) (109) ring A-methyl: ROCK<br>(5.75%) (112) ring A-ethyl: SCIS<br>(5.03%) (121) ring A-methyl: ROCKa<br>(30.79%) (127) ring A-ethyl: CH2 TWIST
98	1301.47	-------	333.80	16.94	(6.34%) (25) ring B: C-C stretch (70,69)<br>(9.14%) (152) ring B-prop: 1-CH2-TWIST<br>(5.72%) (163) bridge BC: C-H ROCK<br>(5.10%) (194) ring C-prop: 2-CH2-WAGG<br>(5.69%) (199) ring C-prop: C-O-H BEND
99	1290.35	-------	48.21	0.31	(25.23%) (110) ring A-methyl: WAGG<br>(8.40%) (111) ring A-methyl: TWIST<br>(11.29%) (114) ring A-ethyl: WAGG<br>(5.93%) (115) ring A-ethyl: TWIST<br>(5.36%) (126) ring A-ethyl: CH2 WAGG
100	1288.49	-------	22.95	1.93	(9.76%) (151) ring B-prop: 1-CH2-WAGG<br>(10.94%) (152) ring B-prop: 1-CH2-TWIST<br>(14.98%) (156) ring B-prop: 2-CH2-WAGG<br>(11.31%) (157) ring B-prop: 2-CH2-TWIST<br>(20.60%) (161) ring B-prop: C-O-H BEND
101	1276.29	-------	91.76	11.40	(25.00%) (53) ring C: C-N stretch (14, 9)<br>(5.00%) (75) ring D: C-N stretch (33,28)<br>(15.37%) (171) ring C: N-H ROCK<br>(11.51%) (200) ring D: N-H ROCK
102	1273.29	-------	10.49	0.28	(53.95%) (219) ring D-ethyl: CH2 TWIST<br>(15.13%) (224) ring D-ethyl: CH3 ROCKb
103	1256.23	-------	50.61	0.69	(6.00%) (108) ring A-methyl: SCIS<br>(15.80%) (112) ring A-ethyl: SCIS<br>(6.48%) (125) ring A-ethyl: CH2 ROCK<br>(21.19%) (127) ring A-ethyl: CH2 TWIST<br>(11.95%) (131) ring A-ethyl: CH3 ROCKa<br>(5.07%) (152) ring B-prop: 1-CH2-TWIST
104	1255.32	-------	13.42	0.22	(5.08%) (127) ring A-ethyl: CH2 TWIST<br>(13.11%) (151) ring B-prop: 1-CH2-WAGG<br>(21.77%) (152) ring B-prop: 1-CH2-TWIST<br>(20.53%) (156) ring B-prop: 2-CH2-WAGG<br>(10.83%) (157) ring B-prop: 2-CH2-TWIST
105	1242.34	-------	38.66	0.31	(8.53%) (166) bridge CD: C-H ROCK<br>(37.05%) (190) ring C-prop: 1-CH2-TWIST<br>(8.23%) (194) ring C-prop: 2-CH2-WAGG<br>(7.33%) (195) ring C-prop: 2-CH2-TWIST
106	1239.03	-------	33.75	0.18	(17.34%) (110) ring A-methyl: WAGG<br>(8.18%) (111) ring A-methyl: TWIST<br>(7.97%) (112) ring A-ethyl: SCIS<br>(20.39%) (114) ring A-ethyl: WAGG

					(11.45%) (115) ring A-ethyl: TWIST
107	1238.54	-------	433.41	0.92	(8.39%) (2) ring A: C-N stretch (48,46)
					(6.54%) (75) ring D: C-N stretch (33,28)
					(5.39%) (88) ring A bending
					(5.40%) (106) ring A: C=O ROCK
					(12.63%) (166) bridge CD: C-H ROCK
					(8.80%) (169) bridge AB: C-H ROCK
108	1224.12	-------	525.35	1.65	(5.79%) (53) ring C: C-N stretch (14, 9)
					(11.43%) (166) bridge CD: C-H ROCK
					(19.53%) (195) ring C-prop: 2-CH2-TWIST
109	1204.04	-------	66.44	0.27	(8.08%) (152) ring B-prop: 1-CH2-TWIST
					(10.72%) (157) ring B-prop: 2-CH2-TWIST
					(9.73%) (190) ring C-prop: 1-CH2-TWIST
					(33.60%) (195) ring C-prop: 2-CH2-TWIST
110	1199.33	-------	343.99	1.19	(5.09%) (38) ring B-prop: C-O stretch (83,81)
					(13.43%) (152) ring B-prop: 1-CH2-TWIST
					(16.61%) (157) ring B-prop: 2-CH2-TWIST
					(5.95%) (161) ring B-prop: C-O-H BEND
					(8.50%) (195) ring C-prop: 2-CH2-TWIST
111	1177.03	-------	370.41	0.10	(16.99%) (2) ring A: C-N stretch (48,46)
					(5.42%) (3) ring A: C-C stretch (49,48)
					(16.26%) (7) ring A: C-N stretch (53,46)
					(7.78%) (8) ring A: C-C stretch (53,51)
					(5.09%) (21) ring B: C-N stretch (67,65)
					(9.55%) (169) bridge AB: C-H ROCK
112	1167.33	-------	82.82	2.88	(13.65%) (49) ring C: C-N stretch (11, 9)
					(7.07%) (50) ring C: C-C stretch (12,11)
					(8.08%) (52) bridge CD: C-C stretch (14, 5)
					(11.40%) (53) ring C: C-N stretch (14, 9)
					(13.34%) (55) ring C-methyl: C-C stretch (15,13)
					(7.79%) (97) ring C bending
113	1152.60	-------	182.73	1.99	(5.37%) (26) ring B-methyl: C-C stretch (71,68)
					(24.48%) (38) ring B-prop: C-O stretch (83,81)
					(5.40%) (68) ring C-prop: C-O stretch (27,25)
					(7.45%) (157) ring B-prop: 2-CH2-TWIST
					(11.09%) (161) ring B-prop: C-O-H BEND
114	1148.11	-------	250.16	0.06	(5.03%) (38) ring B-prop: C-O stretch (83,81)
					(24.09%) (68) ring C-prop: C-O stretch (27,25)
					(6.12%) (194) ring C-prop: 2-CH2-WAGG
					(11.23%) (199) ring C-prop: C-O-H BEND
115	1147.46	-------	60.72	0.05	(6.76%) (5) ring A: C-C stretch (51,49)
					(5.78%) (109) ring A-methyl: ROCK
					(7.65%) (113) ring A-ethyl: ROCK
					(10.03%) (121) ring A-methyl: ROCKa
					(6.64%) (122) ring A-methyl: ROCKb
					(15.74%) (125) ring A-ethyl: CH2 ROCK
					(7.55%) (131) ring A-ethyl: CH3 ROCKa
116	1144.63	-------	100.97	0.17	(5.48%) (68) ring C-prop: C-O stretch (27,25)
					(11.47%) (77) ring D-methyl: C-C stretch (34,31)
					(5.20%) (81) ring D-ethyl: C-C stretch (38,32)
					(14.24%) (217) ring D-ethyl: CH2 ROCK
					(11.51%) (224) ring D-ethyl: CH3 ROCKb
117	1126.99	-------	93.84	1.46	(8.89%) (24) ring B: C-N stretch (70,65)
					(5.58%) (30) ring B-prop: C-C stretch (75,69)
					(7.51%) (59) ring C-prop: C-C stretch (19,12)
					(12.41%) (184) ring C-methyl: ROCKa
118	1122.92	-------	81.06	0.85	(10.19%) (21) ring B: C-N stretch (67,65)
					(8.84%) (24) ring B: C-N stretch (70,65)
					(6.18%) (25) ring B: C-C stretch (70,69)
					(7.88%) (26) ring B-methyl: C-C stretch (71,68)
					(6.56%) (45) bridge AB: C-C stretch (7,67)
					(5.42%) (49) ring C: C-N stretch (11, 9)
					(5.77%) (184) ring C-methyl: ROCKa
119	1119.07	-------	75.44	1.42	(8.22%) (81) ring D-ethyl: C-C stretch (38,32)
					(14.69%) (213) ring D-methyl: ROCKa
					(11.68%) (214) ring D-methyl: ROCKb
					(7.38%) (217) ring D-ethyl: CH2 ROCK
					(6.95%) (224) ring D-ethyl: CH3 ROCKb
120	1112.74	-------	166.81	1.16	(9.37%) (24) ring B: C-N stretch (70,65)
					(13.12%) (30) ring B-prop: C-C stretch (75,69)
					(5.57%) (92) ring B bending
					(14.81%) (147) ring B-methyl: ROCKb
					(5.62%) (184) ring C-methyl: ROCKa
121	1097.38	-------	122.91	5.50	(25.72%) (71) ring D: C-N stretch (30,28)
					(21.10%) (75) ring D: C-N stretch (33,28)
					(8.30%) (77) ring D-methyl: C-C stretch (34,31)
					(6.08%) (81) ring D-ethyl: C-C stretch (38,32)
					(11.35%) (200) ring D: N-H ROCK

122	1094.44	-------	102.67	0.71	(10.75%) (5) ring A: C-C stretch (51,49)
					(6.21%) (9) ring A-methyl: C-C stretch (54,49)
					(13.28%) (13) ring A-ethyl: C-C stretch (58,51)
					(5.94%) (115) ring A-ethyl: TWIST
					(6.91%) (123) ring A-ethyl: BEND
					(7.13%) (131) ring A-ethyl: CH3 ROCKa
					(15.36%) (132) ring A-ethyl: CH3 ROCKb
123	1082.97	-------	23.92	0.04	(11.18%) (9) ring A-methyl: C-C stretch (54,49)
					(8.75%) (111) ring A-methyl: TWIST
					(5.17%) (121) ring A-methyl: ROCKa
					(22.66%) (122) ring A-methyl: ROCKb
					(14.23%) (132) ring A-ethyl: CH3 ROCKb
124	1072.64	-------	57.58	1.26	(5.19%) (147) ring B-methyl: ROCKb
					(6.36%) (150) ring B-prop: 1-CH2-ROCK
					(8.86%) (184) ring C-methyl: ROCKa
					(7.90%) (188) ring C-prop: 1-CH2-ROCK
125	1061.68	-------	145.30	2.12	(14.74%) (84) ring D-ethyl: C-C stretch (41,38)
					(12.71%) (213) ring D-methyl: ROCKa
					(8.33%) (215) ring D-ethyl: BEND
					(39.54%) (223) ring D-ethyl: CH3 ROCKa
126	1053.04	-------	12.67	0.87	(6.92%) (178) ring C-methyl: WAGG
					(7.14%) (183) ring C-methyl: ADEFb
					(75.43%) (185) ring C-methyl: ROCKb
127	1047.68	-------	31.63	0.55	(12.58%) (146) ring B-methyl: ROCKa
					(11.97%) (150) ring B-prop: 1-CH2-ROCK
					(8.72%) (155) ring B-prop: 2-CH2-ROCK
					(7.87%) (188) ring C-prop: 1-CH2-ROCK
128	1046.71	-------	2.46	0.07	(8.40%) (138) ring B-methyl: WAGG
					(52.68%) (146) ring B-methyl: ROCKa
					(23.56%) (147) ring B-methyl: ROCKb
129	1040.07	-------	1.49	0.33	(7.26%) (205) ring D-methyl: WAGG
					(21.66%) (213) ring D-methyl: ROCKa
					(51.20%) (214) ring D-methyl: ROCKb
					(7.47%) (223) ring D-ethyl: CH3 ROCKa
130	1027.00	-------	19.81	0.06	(6.50%) (5) ring A: C-C stretch (51,49)
					(6.00%) (13) ring A-ethyl: C-C stretch (58,51)
					(10.88%) (15) ring A-ethyl: C-C stretch (60,58)
					(9.76%) (109) ring A-methyl: ROCK
					(28.57%) (121) ring A-methyl: ROCKa
					(5.20%) (125) ring A-ethyl: CH2 ROCK
					(7.16%) (127) ring A-ethyl: CH2 TWIST
					(6.58%) (132) ring A-ethyl: CH3 ROCKb
131	1025.10	-------	1.64	0.06	(11.01%) (9) ring A-methyl: C-C stretch (54,49)
					(6.96%) (13) ring A-ethyl: C-C stretch (58,51)
					(16.70%) (15) ring A-ethyl: C-C stretch (60,58)
					(6.66%) (112) ring A-ethyl: SCIS
					(17.05%) (122) ring A-methyl: ROCKb
					(10.13%) (131) ring A-ethyl: CH3 ROCKa
132	1020.73	-------	197.05	0.30	(10.56%) (2) ring A: C-N stretch (48,46)
					(5.67%) (7) ring A: C-N stretch (53,46)
					(25.18%) (9) ring A-methyl: C-C stretch (54,49)
					(15.96%) (13) ring A-ethyl: C-C stretch (58,51)
					(16.14%) (15) ring A-ethyl: C-C stretch (60,58)
133	1001.22	-------	50.03	0.91	(5.10%) (51) ring C: C-.C stretch (13,12)
					(44.21%) (62) ring C-prop: C-C stretch (22,19)
					(13.75%) (184) ring C-methyl: ROCKa
					(6.42%) (193) ring C-prop: 2-CH2-ROCK
134	995.56	-------	14.67	0.13	(68.46%) (33) ring B-prop: C-C stretch (78,75)
					(6.03%) (148) ring B-prop: 1-BEND
					(5.39%) (153) ring B-prop: 2-BEND
					(5.13%) (160) ring B-prop: C=O OUT
135	989.08	-------	36.63	2.63	(8.89%) (73) ring D: C=C stretch (32,31)
					(10.04%) (76) ring D: C-C stretch (33,32)
					(7.56%) (77) ring D-methyl: C-C stretch (34,31)
					(11.07%) (84) ring D-ethyl: C-C stretch (41,38)
					(17.72%) (213) ring D-methyl: ROCKa
					(8.07%) (214) ring D-methyl: ROCKb
					(8.16%) (219) ring D-ethyl: CH2 TWIST
					(10.78%) (224) ring D-ethyl: CH3 ROCKb
136	958.27	-------	156.49	0.17	(6.92%) (23) ring B: C-.C stretch (69,68)
					(5.65%) (25) ring B: C-C stretch (70,69)
					(12.88%) (147) ring B-methyl: ROCKb
					(12.27%) (150) ring B-prop: 1-CH2-ROCK
					(13.49%) (155) ring B-prop: 2-CH2-ROCK
137	950.56	-------	41.27	0.20	(5.27%) (62) ring C-prop: C-C stretch (22,19)
					(6.72%) (186) ring C-prop: 1-BEND
					(5.41%) (188) ring C-prop: 1-CH2-ROCK

					(5.16%)	(191)	ring C-prop: 2-BEND
					(28.29%)	(193)	ring C-prop: 2-CH2-ROCK
					(14.19%)	(198)	ring C-prop: C=O OUT
138	946.20	-------	16.93	1.06	(5.63%)	(5)	ring A: C-C stretch (51,49)
					(8.47%)	(7)	ring A: C-N stretch (53,46)
					(10.36%)	(8)	ring A: C-C stretch (53,51)
					(27.62%)	(15)	ring A-ethyl: C-C stretch (60,58)
					(5.55%)	(122)	ring A-methyl: ROCKb
					(5.92%)	(132)	ring A-ethyl: CH3 ROCKb
139	935.57	-------	18.96	1.15	(7.39%)	(76)	ring D: C-C stretch (33,32)
					(52.29%)	(84)	ring D-ethyl: C-C stretch (41,38)
					(12.18%)	(223)	ring D-ethyl: CH3 ROCKa
140	935.26	-------	156.08	0.75	(6.99%)	(50)	ring C: C-C stretch (12,11)
					(6.62%)	(62)	ring C-prop: C-C stretch (22,19)
					(11.84%)	(65)	ring C-prop: C-C stretch (25,22)
					(22.13%)	(188)	ring C-prop: 1-CH2-ROCK
					(5.54%)	(193)	ring C-prop: 2-CH2-ROCK
141	924.21	-------	31.56	0.22	(10.22%)	(8)	ring A: C-C stretch (53,51)
					(5.35%)	(36)	ring B-prop: C-C stretch (81,78)
					(6.23%)	(147)	ring B-methyl: ROCKb
					(7.91%)	(155)	ring B-prop: 2-CH2-ROCK
142	909.73	-------	10.70	0.13	(10.84%)	(5)	ring A: C-C stretch (51,49)
					(12.30%)	(8)	ring A: C-C stretch (53,51)
					(9.59%)	(9)	ring A-methyl: C-C stretch (54,49)
					(6.23%)	(36)	ring B-prop: C-C stretch (81,78)
					(11.46%)	(132)	ring A-ethyl: CH3 ROCKb
143	893.70	-------	20.79	0.10	(14.55%)	(36)	ring B-prop: C-C stretch (81,78)
144	886.91	-------	20.31	2.09	(56.53%)	(164)	bridge BC: C-H OUT
					(9.43%)	(233)	bridge BC: 1-TORS
					(7.08%)	(236)	bridge BC: 2-TORS
145	879.32	-------	61.96	2.12	(11.12%)	(54)	ring C: C-C stretch (14,13)
					(5.14%)	(72)	ring D: C-C stretch (31,30)
					(10.43%)	(164)	bridge BC: C-H OUT
146	861.10	-------	4.49	0.16	(7.76%)	(5)	ring A: C-C stretch (51,49)
					(12.37%)	(13)	ring A-ethyl: C-C stretch (58,51)
					(8.04%)	(122)	ring A-methyl: ROCKb
					(20.29%)	(131)	ring A-ethyl: CH3 ROCKa
147	837.47	-------	85.56	2.06	(13.79%)	(3)	ring A: C-C stretch (49,48)
					(-6.89%)	(91)	ring A torsion
					(42.62%)	(105)	ring A: N-H OUT
					(9.41%)	(134)	ring B: N-H OUT
148	829.26	-------	121.15	0.52	(11.56%)	(72)	ring D: C-C stretch (31,30)
					(5.75%)	(224)	ring D-ethyl: CH3 ROCKb
149	820.22	-------	20.36	55.34	(54.10%)	(167)	bridge CD: C-H OUT
					(9.52%)	(203)	ring D: bridge CD WAGG
					(10.88%)	(243)	bridge C=D: TORS
150	808.60	-------	6.61	0.56	(5.96%)	(65)	ring C-prop: C-C stretch (25,22)
					(9.12%)	(165)	bridge CD: BEND
					(5.45%)	(217)	ring D-ethyl: CH2 ROCK
					(6.63%)	(224)	ring D-ethyl: CH3 ROCKb
151	800.37	-------	52.81	0.48	(9.75%)	(3)	ring A: C-C stretch (49,48)
					(6.74%)	(105)	ring A: N-H OUT
					(6.81%)	(125)	ring A-ethyl: CH2 ROCK
					(6.21%)	(131)	ring A-ethyl: CH3 ROCKa
					(15.19%)	(134)	ring B: N-H OUT
					(10.47%)	(172)	ring C: N-H OUT
152	798.05	-------	44.07	0.08	(6.20%)	(136)	ring B: bridge AB WAGG
					(61.39%)	(170)	bridge AB: C-H OUT
					(13.15%)	(234)	bridge A=B: TORS
					(5.60%)	(235)	bridge A-B: TORS
153	792.19	-------	26.06	1.62	(11.51%)	(36)	ring B-prop: C-C stretch (81,78)
					(13.80%)	(150)	ring B-prop: 1-CH2-ROCK
					(7.29%)	(155)	ring B-prop: 2-CH2-ROCK
154	788.91	-------	18.90	0.45	(26.65%)	(65)	ring C-prop: C-C stretch (25,22)
					(11.10%)	(68)	ring C-prop: C-O stretch (27,25)
					(11.07%)	(188)	ring C-prop: 1-CH2-ROCK
155	773.84	-------	3.05	1.06	(10.32%)	(102)	ring D torsion
					(12.43%)	(207)	ring D-ethyl: WAGG
					(46.87%)	(209)	ring D: C=O OUT
156	771.13	-------	24.07	0.45	(7.29%)	(105)	ring A: N-H OUT
					(5.81%)	(168)	bridge AB: BEND
157	762.67	-------	2.59	0.46	(6.38%)	(105)	ring A: N-H OUT
					(28.05%)	(125)	ring A-ethyl: CH2 ROCK

					(7.18%) (131) ring A-ethyl: CH3 ROCKa
					(6.17%) (132) ring A-ethyl: CH3 ROCKb
					(8.37%) (134) ring B: N-H OUT
					(15.07%) (172) ring C: N-H OUT
158	756.46	-------	7.69	0.24	(6.97%) (76) ring D: C-C stretch (33,32)
					(6.98%) (100) ring D bending
					(8.25%) (209) ring D: C=O OUT
					(26.04%) (217) ring D-ethyl: CH2 ROCK
					(13.56%) (224) ring D-ethyl: CH3 ROCKb
159	749.38	-------	6.07	0.26	(5.36%) (100) ring D bending
					(9.97%) (105) ring A: N-H OUT
					(16.14%) (172) ring C: N-H OUT
160	742.10	-------	8.87	0.57	(13.40%) (105) ring A: N-H OUT
					(6.25%) (125) ring A-ethyl: CH2 ROCK
					(5.83%) (172) ring C: N-H OUT
161	733.20	-------	39.19	5.06	(5.83%) (92) ring B bending
					(6.58%) (94) ring B torsion
					(21.87%) (134) ring B: N-H OUT
162	730.83	-------	7.56	3.98	(6.28%) (94) ring B torsion
					(13.71%) (100) ring D bending
					(6.82%) (134) ring B: N-H OUT
					(7.94%) (140) ring B-prop: 1-WAGG
					(5.04%) (142) ring B: bridge BC WAGG
					(9.82%) (172) ring C: N-H OUT
163	719.90	-------	19.25	0.67	(9.85%) (98) ring C torsion
					(7.53%) (174) ring C: bridge BC WAGG
					(8.95%) (176) ring C-prop: 1-WAGG
164	713.67	-------	13.08	3.48	(5.34%) (96) ring C bending
					(8.64%) (100) ring D bending
					(9.54%) (107) ring A: C=O OUT
					(21.72%) (134) ring B: N-H OUT
					(22.64%) (172) ring C: N-H OUT
165	707.23	-------	2.61	1.01	(21.13%) (95) ring B torsion
					(27.19%) (136) ring B: bridge AB WAGG
					(9.39%) (234) bridge A=B: TORS
166	698.75	-------	103.96	0.22	(9.76%) (155) ring B-prop: 2-CH2-ROCK
					(8.70%) (160) ring B-prop: C=O OUT
					(40.25%) (232) ring B-prop: 4-TORS
167	687.67	-------	71.70	1.31	(9.69%) (92) ring B bending
					(13.63%) (242) ring C-prop: 4-TORS
168	683.62	-------	41.17	2.13	(7.34%) (98) ring C torsion
					(5.22%) (134) ring B: N-H OUT
					(5.27%) (198) ring C-prop: C=O OUT
					(24.30%) (242) ring C-prop: 4-TORS
169	680.49	-------	38.33	0.34	(6.08%) (55) ring C-methyl: C-C stretch (15,13)
					(8.20%) (99) ring C torsion
					(8.86%) (107) ring A: C=O OUT
					(12.33%) (242) ring C-prop: 4-TORS
170	673.99	-------	18.46	0.70	(6.06%) (81) ring D-ethyl: C-C stretch (38,32)
					(7.37%) (95) ring B torsion
					(11.74%) (99) ring C torsion
					(6.81%) (100) ring D bending
					(5.48%) (180) ring C: bridge CD WAGG
					(8.52%) (242) ring C-prop: 4-TORS
171	669.95	-------	13.60	0.72	(12.17%) (55) ring C-methyl: C-C stretch (15,13)
172	660.28	-------	5.13	0.36	(13.40%) (88) ring A bending
					(17.18%) (117) ring A: bridge AB WAGG
173	657.53	-------	12.39	7.61	(6.60%) (99) ring C torsion
					(6.59%) (103) ring D torsion
					(6.52%) (203) ring D: bridge CD WAGG
					(5.07%) (205) ring D-methyl: WAGG
					(5.75%) (208) ring D: C=O ROCK
					(6.40%) (209) ring D: C=O OUT
174	653.87	-------	15.48	27.87	(9.46%) (102) ring D torsion
					(14.77%) (103) ring D torsion
					(24.38%) (203) ring D: bridge CD WAGG
					(8.57%) (205) ring D-methyl: WAGG
175	631.48	-------	16.29	0.65	(10.59%) (26) ring B-methyl: C-C stretch (71,68)
					(9.05%) (93) ring B bending
					(15.00%) (159) ring B-prop: C=O ROCK
					(5.21%) (232) ring B-prop: 4-TORS
176	620.25	-------	48.57	0.26	(13.02%) (159) ring B-prop: C=O ROCK
					(17.57%) (197) ring C-prop: C=O ROCK
					(7.08%) (242) ring C-prop: 4-TORS

177	610.53	-------	30.04	0.24	(6.35%) (13) ring A-ethyl: C-C stretch (58,51)<br>(8.73%) (88) ring A bending<br>(34.86%) (89) ring A bending<br>(18.25%) (117) ring A: bridge AB WAGG
178	605.25	-------	16.80	0.88	(7.56%) (93) ring B bending<br>(7.09%) (159) ring B-prop: C=O ROCK<br>(26.24%) (197) ring C-prop: C=O ROCK
179	594.24	-------	19.31	0.21	(7.54%) (26) ring B-methyl: C-C stretch (71,68)<br>(8.07%) (55) ring C-methyl: C-C stretch (15,13)<br>(5.80%) (59) ring C-prop: C-C stretch (19,12)<br>(12.69%) (93) ring B bending<br>(18.09%) (97) ring C bending
180	572.99	-------	20.68	0.34	(6.13%) (77) ring D-methyl: C-C stretch (34,31)<br>(8.74%) (208) ring D: C=O ROCK
181	560.40	-------	19.01	0.17	(6.52%) (142) ring B: bridge BC WAGG<br>(7.92%) (148) ring B-prop: 1-BEND<br>(6.45%) (158) ring B-prop: 3-BEND<br>(9.62%) (159) ring B-prop: C=O ROCK<br>(16.51%) (160) ring B-prop: C=O OUT<br>(12.81%) (232) ring B-prop: 4-TORS
182	555.72	-------	12.14	1.12	(10.41%) (77) ring D-methyl: C-C stretch (34,31)<br>(30.80%) (101) ring D bending<br>(5.05%) (207) ring D-ethyl: WAGG<br>(5.36%) (209) ring D: C=O OUT<br>(9.64%) (215) ring D-ethyl: BEND
183	534.00	-------	14.21	2.26	(20.81%) (106) ring A: C=O ROCK<br>(5.16%) (116) ring A: bridge AB ROCK<br>(5.05%) (139) ring B-prop: 1-ROCK
184	520.89	-------	9.87	0.33	(8.97%) (196) ring C-prop: 3-BEND<br>(5.49%) (198) ring C-prop: C=O OUT
185	510.62	-------	41.54	1.69	(7.12%) (106) ring A: C=O ROCK<br>(6.94%) (193) ring C-prop: 2-CH2-ROCK<br>(5.28%) (197) ring C-prop: C=O ROCK<br>(11.62%) (198) ring C-prop: C=O OUT<br>(5.08%) (242) ring C-prop: 4-TORS
186	500.11	-------	10.04	4.44	(9.29%) (137) ring B-methyl: ROCK<br>(5.21%) (139) ring B-prop: 1-ROCK<br>(6.43%) (196) ring C-prop: 3-BEND
187	499.14	-------	5.08	4.42	(8.59%) (101) ring D bending<br>(6.25%) (198) ring C-prop: C=O OUT<br>(11.20%) (201) ring D: N-H OUT<br>(6.58%) (205) ring D-methyl: WAGG<br>(9.99%) (207) ring D-ethyl: WAGG<br>(8.69%) (209) ring D: C=O OUT
188	492.26	-------	8.31	0.89	(5.42%) (193) ring C-prop: 2-CH2-ROCK<br>(6.48%) (196) ring C-prop: 3-BEND<br>(14.47%) (198) ring C-prop: C=O OUT
189	482.00	-------	6.97	1.73	(6.42%) (142) ring B: bridge BC WAGG<br>(8.64%) (196) ring C-prop: 3-BEND
190	440.16	-------	0.56	0.46	(6.27%) (5) ring A: C-C stretch (51,49)<br>(5.22%) (9) ring A-methyl: C-C stretch (54,49)<br>(5.59%) (13) ring A-ethyl: C-C stretch (58,51)<br>(9.96%) (109) ring A-methyl: ROCK<br>(5.03%) (112) ring A-ethyl: SCIS<br>(10.24%) (113) ring A-ethyl: ROCK<br>(5.16%) (123) ring A-ethyl: BEND
191	436.75	-------	73.37	4.26	(-9.37%) (103) ring D torsion<br>(69.91%) (201) ring D: N-H OUT<br>(6.72%) (203) ring D: bridge CD WAGG<br>(5.23%) (215) ring D-ethyl: BEND
192	430.77	-------	13.36	1.98	(6.02%) (155) ring B-prop: 2-CH2-ROCK<br>(25.94%) (158) ring B-prop: 3-BEND<br>(5.61%) (196) ring C-prop: 3-BEND<br>(8.91%) (201) ring D: N-H OUT
193	404.93	-------	5.87	1.21	(5.17%) (123) ring A-ethyl: BEND<br>(5.49%) (136) ring B: bridge AB WAGG<br>(7.66%) (138) ring B-methyl: WAGG<br>(12.40%) (158) ring B-prop: 3-BEND<br>(6.38%) (174) ring C: bridge BC WAGG<br>(5.40%) (180) ring C: bridge CD WAGG<br>(5.16%) (196) ring C-prop: 3-BEND
194	401.61	-------	8.82	0.12	(8.24%) (106) ring A: C=O ROCK<br>(17.42%) (123) ring A-ethyl: BEND
195	360.50	-------	0.10	0.88	(9.03%) (178) ring C-methyl: WAGG

					(8.27%)	(179)	ring C: bridge CD ROCK
					(6.57%)	(205)	ring D-methyl: WAGG
196	351.37	-------	2.73	1.20	(7.33%)	(175)	ring C-prop: 1-ROCK
					(23.91%)	(178)	ring C-methyl: WAGG
					(7.47%)	(186)	ring C-prop: 1-BEND
					(13.27%)	(196)	ring C-prop: 3-BEND
					(7.06%)	(205)	ring D-methyl: WAGG
197	343.05	-------	6.38	0.94	(5.65%)	(123)	ring A-ethyl: BEND
					(9.81%)	(137)	ring B-methyl: ROCK
					(9.38%)	(138)	ring B-methyl: WAGG
					(21.64%)	(139)	ring B-prop: 1-ROCK
					(5.61%)	(153)	ring B-prop: 2-BEND
198	327.61	-------	1.08	8.06	(8.37%)	(137)	ring B-methyl: ROCK
					(28.78%)	(138)	ring B-methyl: WAGG
					(6.90%)	(139)	ring B-prop: 1-ROCK
					(5.05%)	(140)	ring B-prop: 1-WAGG
					(10.93%)	(158)	ring B-prop: 3-BEND
199	320.71	-------	2.01	26.21	(13.20%)	(175)	ring C-prop: 1-ROCK
					(5.22%)	(191)	ring C-prop: 2-BEND
					(5.50%)	(203)	ring D: bridge CD WAGG
					(10.65%)	(205)	ring D-methyl: WAGG
					(8.72%)	(215)	ring D-ethyl: BEND
200	317.69	-------	2.56	0.16	(42.22%)	(204)	ring D-methyl: ROCK
					(12.26%)	(205)	ring D-methyl: WAGG
					(8.46%)	(208)	ring D: C=O ROCK
					(-5.53%)	(237)	bridge C-D: TORS
201	305.47	-------	1.93	5.16	(11.18%)	(95)	ring B torsion
					(21.73%)	(177)	ring C-methyl: ROCK
					(12.00%)	(243)	bridge C=D: TORS
					(5.06%)	(246)	ring D-ethyl: 2-TORS
202	295.07	-------	0.57	3.05	(15.51%)	(178)	ring C-methyl: WAGG
					(12.25%)	(206)	ring D-ethyl: ROCK
					(22.78%)	(246)	ring D-ethyl: 2-TORS
203	292.99	-------	2.68	6.91	(5.23%)	(97)	ring C bending
					(6.78%)	(175)	ring C-prop: 1-ROCK
					(9.03%)	(177)	ring C-methyl: ROCK
					(5.99%)	(191)	ring C-prop: 2-BEND
					(7.14%)	(204)	ring D-methyl: ROCK
204	288.39	-------	2.82	12.79	(6.24%)	(204)	ring D-methyl: ROCK
					(10.26%)	(206)	ring D-ethyl: ROCK
					(5.18%)	(208)	ring D: C=O ROCK
					(13.66%)	(246)	ring D-ethyl: 2-TORS
205	280.65	-------	2.31	4.85	(5.36%)	(111)	ring A-methyl: TWIST
					(15.46%)	(123)	ring A-ethyl: BEND
					(5.41%)	(137)	ring B-methyl: ROCK
206	270.02	-------	0.62	0.17	(7.15%)	(177)	ring C-methyl: ROCK
					(23.29%)	(227)	ring A-ethyl: 2-TORS
207	257.21	-------	2.83	0.55	(10.14%)	(109)	ring A-methyl: ROCK
					(6.81%)	(225)	ring A-methyl: TORS
					(22.09%)	(227)	ring A-ethyl: 2-TORS
208	248.61	-------	1.62	0.28	(7.20%)	(110)	ring A-methyl: WAGG
					(7.15%)	(137)	ring B-methyl: ROCK
					(5.86%)	(138)	ring B-methyl: WAGG
					(22.00%)	(225)	ring A-methyl: TORS
209	238.13	-------	0.52	0.26	(10.94%)	(111)	ring A-methyl: TWIST
					(57.69%)	(225)	ring A-methyl: TORS
210	234.45	-------	1.99	0.65	(6.68%)	(202)	ring D: bridge CD ROCK
					(8.95%)	(225)	ring A-methyl: TORS
					(12.90%)	(227)	ring A-ethyl: 2-TORS
					(5.16%)	(234)	bridge A=B: TORS
211	224.92	-------	2.48	0.77	(7.56%)	(135)	ring B: bridge AB ROCK
					(6.16%)	(137)	ring B-methyl: ROCK
					(5.27%)	(202)	ring D: bridge CD ROCK
					(6.25%)	(227)	ring A-ethyl: 2-TORS
					(33.80%)	(238)	ring C-methyl: TORS
212	220.36	-------	0.74	1.28	(37.86%)	(238)	ring C-methyl: TORS
					(9.01%)	(244)	ring D-methyl: TORS
					(8.70%)	(246)	ring D-ethyl: 2-TORS
213	216.56	-------	1.46	0.57	(9.71%)	(135)	ring B: bridge AB ROCK
					(10.72%)	(137)	ring B-methyl: ROCK
					(10.99%)	(153)	ring B-prop: 2-BEND
					(10.64%)	(246)	ring D-ethyl: 2-TORS
214	209.70	-------	2.34	0.71	(6.13%)	(176)	ring C-prop: 1-WAGG
					(9.59%)	(206)	ring D-ethyl: ROCK

					(14.08%) (246) ring D-ethyl: 2-TORS
215	197.96	-------	4.14	2.11	(8.59%) (91) ring A torsion
					(7.39%) (141) ring B: bridge BC ROCK
					(7.05%) (235) bridge A-B: TORS
					(6.63%) (246) ring D-ethyl: 2-TORS
					(11.34%) (248) Cl-H stretch
216	188.80	-------	1.77	3.42	(5.07%) (91) ring A torsion
					(8.10%) (153) ring B-prop: 2-BEND
					(6.07%) (186) ring C-prop: 1-BEND
					(5.68%) (227) ring A-ethyl: 2-TORS
217	185.28	-------	2.67	2.11	(8.40%) (102) ring D torsion
					(7.08%) (205) ring D-methyl: WAGG
					(6.16%) (207) ring D-ethyl: WAGG
					(8.34%) (215) ring D-ethyl: BEND
					(5.76%) (237) bridge C-D: TORS
					(16.39%) (248) Cl-H stretch
218	180.20	-------	3.92	0.27	(10.31%) (91) ring A torsion
					(5.58%) (123) ring A-ethyl: BEND
219	169.11	-------	3.79	0.31	(9.25%) (91) ring A torsion
					(6.42%) (95) ring B torsion
					(5.74%) (109) ring A-methyl: ROCK
					(6.77%) (162) bridge BC: BEND
					(14.25%) (235) bridge A-B: TORS
					(12.37%) (248) Cl-H stretch
220	167.25	-------	5.47	2.63	(5.38%) (91) ring A torsion
					(9.89%) (207) ring D-ethyl: WAGG
					(9.89%) (215) ring D-ethyl: BEND
					(30.37%) (248) Cl-H stretch
221	164.84	-------	0.10	1.13	(6.32%) (98) ring C torsion
					(13.17%) (237) bridge C-D: TORS
					(6.44%) (238) ring C-methyl: TORS
					(7.93%) (243) bridge C=D: TORS
					(7.95%) (244) ring D-methyl: TORS
					(6.66%) (248) Cl-H stretch
222	151.32	-------	1.05	7.51	(7.79%) (103) ring D torsion
					(8.69%) (153) ring B-prop: 2-BEND
					(5.25%) (237) bridge C-D: TORS
					(16.73%) (247) Cl-H stretch
223	147.25	-------	5.26	0.31	(11.93%) (91) ring A torsion
					(5.07%) (114) ring A-ethyl: WAGG
					(6.88%) (115) ring A-ethyl: TWIST
					(-6.79%) (235) bridge A-B: TORS
					(29.54%) (247) Cl-H stretch
					(8.71%) (249) Cl OUT
224	140.87	-------	1.03	1.41	(5.13%) (91) ring A torsion
					(5.36%) (102) ring D torsion
					(12.72%) (103) ring D torsion
					(5.42%) (141) ring B: bridge BC ROCK
					(18.88%) (228) ring B-methyl: TORS
225	137.77	-------	3.68	0.23	(12.33%) (103) ring D torsion
					(19.18%) (228) ring B-methyl: TORS
					(10.27%) (244) ring D-methyl: TORS
					(5.15%) (247) Cl-H stretch
226	131.77	-------	1.00	0.46	(5.26%) (226) ring A-ethyl: 1-TORS
					(49.82%) (228) ring B-methyl: TORS
					(12.85%) (244) ring D-methyl: TORS
227	127.19	-------	0.54	0.33	(5.85%) (116) ring A: bridge AB ROCK
					(5.47%) (168) bridge AB: BEND
					(6.26%) (175) ring C-prop: 1-ROCK
					(16.75%) (226) ring A-ethyl: 1-TORS
					(6.47%) (247) Cl-H stretch
228	122.12	-------	0.84	5.06	(7.36%) (174) ring C: bridge BC WAGG
					(37.35%) (244) ring D-methyl: TORS
229	104.50	-------	1.03	1.55	(5.59%) (102) ring D torsion
					(6.77%) (173) ring C: bridge BC ROCK
					(30.01%) (226) ring A-ethyl: 1-TORS
230	101.75	-------	0.27	7.03	(5.41%) (98) ring C torsion
					(22.19%) (102) ring D torsion
					(9.61%) (207) ring D-ethyl: WAGG
					(14.88%) (226) ring A-ethyl: 1-TORS
					(6.10%) (237) bridge C-D: TORS
231	90.62	-------	0.88	0.80	(16.89%) (94) ring B torsion
					(6.40%) (117) ring A: bridge AB WAGG
					(9.23%) (140) ring B-prop: 1-WAGG
					(6.73%) (148) ring B-prop: 1-BEND
					(7.11%) (229) ring B-prop: 1-TORS

					(5.65%)	(233)	bridge BC: 1-TORS
					(5.34%)	(247)	Cl-H stretch
232	80.29	-------	1.03	1.24	(5.00%)	(103)	ring D torsion
					(13.25%)	(116)	ring A: bridge AB ROCK
					(5.80%)	(135)	ring B: bridge AB ROCK
					(15.10%)	(168)	bridge AB: BEND
					(11.85%)	(226)	ring A-ethyl: 1-TORS
					(5.22%)	(235)	bridge A-B: TORS
					(6.23%)	(239)	ring C-prop: 1-TORS
					(6.28%)	(240)	ring C-prop: 2-TORS
					(13.35%)	(249)	Cl OUT
233	76.29	-------	0.42	1.19	(5.44%)	(103)	ring D torsion
					(6.42%)	(142)	ring B: bridge BC WAGG
					(6.15%)	(233)	bridge BC: 1-TORS
					(22.27%)	(239)	ring C-prop: 1-TORS
234	69.05	-------	2.80	1.49	(12.90%)	(229)	ring B-prop: 1-TORS
					(8.39%)	(234)	bridge A=B: TORS
					(9.32%)	(236)	bridge BC: 2-TORS
					(6.74%)	(237)	bridge C-D: TORS
					(12.24%)	(249)	Cl OUT
235	61.51	-------	0.84	0.33	(5.70%)	(102)	ring D torsion
					(5.97%)	(229)	ring B-prop: 1-TORS
					(5.77%)	(236)	bridge BC: 2-TORS
					(16.44%)	(240)	ring C-prop: 2-TORS
					(10.53%)	(249)	Cl OUT
236	59.02	-------	0.02	3.24	(6.56%)	(162)	bridge BC: BEND
					(10.83%)	(165)	bridge CD: BEND
					(13.49%)	(229)	ring B-prop: 1-TORS
					(11.42%)	(231)	ring B-prop: 3-TORS
					(5.35%)	(245)	ring D-ethyl: 1-TORS
237	45.87	-------	4.04	0.56	(5.09%)	(98)	ring C torsion
					(9.06%)	(229)	ring B-prop: 1-TORS
					(32.84%)	(230)	ring B-prop: 2-TORS
					(11.13%)	(231)	ring B-prop: 3-TORS
					(12.55%)	(241)	ring C-prop: 3-TORS
238	44.34	-------	0.67	0.32	(6.20%)	(180)	ring C: bridge CD WAGG
					(10.23%)	(230)	ring B-prop: 2-TORS
					(36.88%)	(231)	ring B-prop: 3-TORS
					(13.21%)	(241)	ring C-prop: 3-TORS
239	41.47	-------	0.27	2.11	(5.12%)	(241)	ring C-prop: 3-TORS
					(5.56%)	(243)	bridge C=D: TORS
					(56.80%)	(245)	ring D-ethyl: 1-TORS
240	36.21	-------	0.92	0.85	(40.70%)	(90)	ring A torsion
					(7.22%)	(234)	bridge A=B: TORS
					(11.39%)	(235)	bridge A-B: TORS
					(5.19%)	(241)	ring C-prop: 3-TORS
					(9.80%)	(245)	ring D-ethyl: 1-TORS
241	29.93	-------	1.28	4.14	(11.29%)	(90)	ring A torsion
					(5.21%)	(230)	ring B-prop: 2-TORS
					(5.25%)	(233)	bridge BC: 1-TORS
					(10.27%)	(237)	bridge C-D: TORS
					(27.80%)	(241)	ring C-prop: 3-TORS
					(9.82%)	(249)	Cl OUT
242	28.63	-------	0.13	5.00	(8.46%)	(90)	ring A torsion
					(5.72%)	(94)	ring B torsion
					(10.17%)	(136)	ring B: bridge AB WAGG
					(7.56%)	(140)	ring B-prop: 1-WAGG
					(10.87%)	(229)	ring B-prop: 1-TORS
					(13.30%)	(234)	bridge A=B: TORS
					(7.53%)	(239)	ring C-prop: 1-TORS
					(5.06%)	(241)	ring C-prop: 3-TORS
243	26.79	-------	1.20	1.10	(6.23%)	(140)	ring B-prop: 1-WAGG
					(15.84%)	(229)	ring B-prop: 1-TORS
					(18.65%)	(230)	ring B-prop: 2-TORS
					(21.03%)	(239)	ring C-prop: 1-TORS
					(11.83%)	(240)	ring C-prop: 2-TORS
244	24.19	-------	0.25	2.27	(6.33%)	(229)	ring B-prop: 1-TORS
					(19.81%)	(230)	ring B-prop: 2-TORS
					(12.97%)	(231)	ring B-prop: 3-TORS
					(19.27%)	(240)	ring C-prop: 2-TORS
					(5.10%)	(243)	bridge C=D: TORS
					(6.95%)	(245)	ring D-ethyl: 1-TORS
245	22.03	-------	1.30	7.37	(5.51%)	(103)	ring D torsion
					(5.13%)	(167)	bridge CD: C-H OUT
					(6.07%)	(230)	ring B-prop: 2-TORS
					(14.43%)	(237)	bridge C-D: TORS
					(13.02%)	(239)	ring C-prop: 1-TORS
					(9.65%)	(241)	ring C-prop: 3-TORS
					(9.77%)	(249)	Cl OUT

246	19.73	-------	0.11	5.35	(7.95%) (162) bridge BC: BEND<br>(11.34%) (165) bridge CD: BEND<br>(9.65%) (179) ring C: bridge CD ROCK<br>(6.25%) (240) ring C-prop: 2-TORS<br>(9.39%) (245) ring D-ethyl: 1-TORS
247	15.37	-------	0.17	3.11	(21.31%) (90) ring A torsion<br>(26.45%) (235) bridge A-B: TORS<br>(8.47%) (236) bridge BC: 2-TORS
248	12.06	-------	0.93	35.81	(5.99%) (162) bridge BC: BEND<br>(12.01%) (174) ring C: bridge BC WAGG<br>(8.10%) (233) bridge BC: 1-TORS<br>(28.87%) (237) bridge C-D: TORS<br>(7.82%) (249) Cl OUT
249	10.62	-------	0.01	6.34	(5.28%) (90) ring A torsion<br>(5.03%) (136) ring B: bridge AB WAGG<br>(15.13%) (233) bridge BC: 1-TORS<br>(5.24%) (235) bridge A-B: TORS<br>(23.28%) (236) bridge BC: 2-TORS

ZZEssa n.a. D_2O

calc.	exp. [cm-1]	IRint [cm-1]	Rint [km/mol]	contribution (rel.)	
1	3522.77	-------	54.89	0.14	(100.07%) (67) ring C-prop: O-H stretch (27,84)
2	3516.59	-------	56.05	0.17	(100.07%) (39) ring B-prop: O-H stretch (85,83)
3	3067.40	-------	0.44	0.17	(99.76%) (42) bridge BC: C-H stretch (4, 3)
4	3056.72	-------	19.21	0.24	(99.85%) (46) bridge AB: C-H stretch (8, 7)
5	3013.51	-------	6.62	0.09	(50.09%) (56) ring C-methyl: C-H stretch (16,15)
					(46.83%) (57) ring C-methyl: C-H stretch (17,15)
6	3012.89	-------	13.92	0.10	(79.24%) (78) ring D-methyl: C-H stretch (35,34)
					(5.51%) (79) ring D-methyl: C-H stretch (36,34)
					(12.73%) (80) ring D-methyl: C-H stretch (37,34)
7	3012.05	-------	14.02	0.06	(98.37%) (43) bridge CD: C-H stretch (6, 5)
8	3006.08	-------	18.98	0.07	(9.15%) (85) ring D-ethyl: C-H stretch (42,41)
					(11.25%) (86) ring D-ethyl: C-H stretch (43,41)
					(75.62%) (87) ring D-ethyl: C-H stretch (44,41)
9	3003.59	-------	10.90	0.08	(16.39%) (10) ring A-methyl: C-H stretch (55,54)
					(6.14%) (11) ring A-methyl: C-H stretch (56,54)
					(77.33%) (12) ring A-methyl: C-H stretch (57,54)
10	3000.91	-------	15.26	0.03	(6.78%) (60) ring C-prop: C-H stretch (20,19)
					(71.63%) (61) ring C-prop: C-H stretch (21,19)
					(19.66%) (63) ring C-prop: C-H stretch (23,22)
11	2998.99	-------	34.26	0.12	(13.34%) (27) ring B-methyl: C-H stretch (72,71)
					(7.80%) (31) ring B-prop: C-H stretch (76,75)
					(9.99%) (32) ring B-prop: C-H stretch (77,75)
					(60.04%) (34) ring B-prop: C-H stretch (79,78)
					(7.03%) (35) ring B-prop: C-H stretch (80,78)
12	2996.58	-------	11.89	0.10	(73.56%) (27) ring B-methyl: C-H stretch (72,71)
					(5.57%) (29) ring B-methyl: C-H stretch (74,71)
					(13.73%) (34) ring B-prop: C-H stretch (79,78)
13	2987.26	-------	28.02	0.17	(31.92%) (17) ring A-ethyl: C-H stretch (62,60)
					(58.86%) (18) ring A-ethyl: C-H stretch (63,60)
14	2985.50	-------	50.70	0.41	(51.26%) (85) ring D-ethyl: C-H stretch (42,41)
					(45.14%) (86) ring D-ethyl: C-H stretch (43,41)
15	2984.68	-------	1.10	0.06	(18.40%) (61) ring C-prop: C-H stretch (21,19)
					(70.81%) (63) ring C-prop: C-H stretch (23,22)
					(7.85%) (64) ring C-prop: C-H stretch (24,22)
16	2984.25	-------	45.05	0.16	(45.86%) (10) ring A-methyl: C-H stretch (55,54)
					(43.19%) (11) ring A-methyl: C-H stretch (56,54)
					(5.31%) (18) ring A-ethyl: C-H stretch (63,60)
17	2981.34	-------	8.87	0.06	(8.43%) (56) ring C-methyl: C-H stretch (16,15)
					(10.04%) (57) ring C-methyl: C-H stretch (17,15)
					(8.85%) (58) ring C-methyl: C-H stretch (18,15)
					(36.13%) (79) ring D-methyl: C-H stretch (36,34)
					(36.00%) (80) ring D-methyl: C-H stretch (37,34)
18	2979.96	-------	8.82	0.13	(27.76%) (56) ring C-methyl: C-H stretch (16,15)
					(27.08%) (57) ring C-methyl: C-H stretch (17,15)
					(17.98%) (58) ring C-methyl: C-H stretch (18,15)
					(12.91%) (79) ring D-methyl: C-H stretch (36,34)
					(13.86%) (80) ring D-methyl: C-H stretch (37,34)
19	2979.84	-------	36.02	0.07	(5.96%) (14) ring A-ethyl: C-H stretch (59,58)
					(46.59%) (16) ring A-ethyl: C-H stretch (61,60)
					(28.78%) (17) ring A-ethyl: C-H stretch (62,60)
					(11.08%) (18) ring A-ethyl: C-H stretch (63,60)
					(6.25%) (40) ring A-ethyl: C-H stretch (1,58)
20	2977.85	-------	4.42	0.04	(46.44%) (31) ring B-prop: C-H stretch (76,75)
					(35.13%) (32) ring B-prop: C-H stretch (77,75)
					(16.51%) (34) ring B-prop: C-H stretch (79,78)
21	2956.12	-------	10.79	0.09	(56.79%) (82) ring D-ethyl: C-H stretch (39,38)
					(36.28%) (83) ring D-ethyl: C-H stretch (40,38)
22	2954.93	-------	14.72	0.16	(48.15%) (28) ring B-methyl: C-H stretch (73,71)
					(52.00%) (29) ring B-methyl: C-H stretch (74,71)
23	2949.88	-------	6.98	0.08	(52.31%) (14) ring A-ethyl: C-H stretch (59,58)
					(9.43%) (17) ring A-ethyl: C-H stretch (62,60)
					(28.60%) (40) ring A-ethyl: C-H stretch (1,58)
24	2947.13	-------	19.20	0.19	(43.74%) (31) ring B-prop: C-H stretch (76,75)
					(52.11%) (32) ring B-prop: C-H stretch (77,75)

25	2941.13	-------	8.18	0.20	(93.99%) (4) ring A: C-H stretch (50,49)
26	2936.45	-------	15.92	0.29	(75.58%) (60) ring C-prop: C-H stretch (20,19)
					(8.49%) (61) ring C-prop: C-H stretch (21,19)
					(13.81%) (64) ring C-prop: C-H stretch (24,22)
27	2930.97	-------	24.20	0.43	(17.31%) (78) ring D-methyl: C-H stretch (35,34)
					(43.16%) (79) ring D-methyl: C-H stretch (36,34)
					(36.55%) (80) ring D-methyl: C-H stretch (37,34)
28	2928.56	-------	2.42	0.14	(8.79%) (34) ring B-prop: C-H stretch (79,78)
					(87.43%) (35) ring B-prop: C-H stretch (80,78)
29	2925.03	-------	11.07	0.09	(14.89%) (60) ring C-prop: C-H stretch (20,19)
					(7.17%) (63) ring C-prop: C-H stretch (23,22)
					(76.98%) (64) ring C-prop: C-H stretch (24,22)
30	2922.01	-------	45.85	0.26	(38.41%) (85) ring D-ethyl: C-H stretch (42,41)
					(38.28%) (86) ring D-ethyl: C-H stretch (43,41)
					(19.06%) (87) ring D-ethyl: C-H stretch (44,41)
31	2921.79	-------	70.47	0.34	(29.50%) (6) ring A: C-H stretch (52,51)
					(15.90%) (10) ring A-methyl: C-H stretch (55,54)
					(23.86%) (11) ring A-methyl: C-H stretch (56,54)
					(10.92%) (12) ring A-methyl: C-H stretch (57,54)
					(9.71%) (14) ring A-ethyl: C-H stretch (59,58)
32	2919.15	-------	2.77	0.04	(16.39%) (6) ring A: C-H stretch (52,51)
					(15.15%) (10) ring A-methyl: C-H stretch (55,54)
					(21.22%) (11) ring A-methyl: C-H stretch (56,54)
					(8.71%) (12) ring A-methyl: C-H stretch (57,54)
					(15.18%) (14) ring A-ethyl: C-H stretch (59,58)
					(14.50%) (40) ring A-ethyl: C-H stretch (1,58)
33	2918.33	-------	21.75	0.22	(37.09%) (82) ring D-ethyl: C-H stretch (39,38)
					(57.06%) (83) ring D-ethyl: C-H stretch (40,38)
34	2917.21	-------	39.66	0.27	(11.68%) (56) ring C-methyl: C-H stretch (16,15)
					(13.90%) (57) ring C-methyl: C-H stretch (17,15)
					(70.90%) (58) ring C-methyl: C-H stretch (18,15)
35	2915.75	-------	27.24	0.10	(9.85%) (6) ring A: C-H stretch (52,51)
					(41.08%) (16) ring A-ethyl: C-H stretch (61,60)
					(26.18%) (17) ring A-ethyl: C-H stretch (62,60)
					(18.90%) (18) ring A-ethyl: C-H stretch (63,60)
36	2911.29	-------	6.32	0.19	(39.40%) (6) ring A: C-H stretch (52,51)
					(11.35%) (14) ring A-ethyl: C-H stretch (59,58)
					(44.72%) (40) ring A-ethyl: C-H stretch (1,58)
37	2909.72	-------	43.68	0.45	(12.35%) (27) ring B-methyl: C-H stretch (72,71)
					(45.88%) (28) ring B-methyl: C-H stretch (73,71)
					(41.11%) (29) ring B-methyl: C-H stretch (74,71)
38	2561.74	-------	38.38	0.14	(98.25%) (69) ring D: N-H stretch (29,28)
39	2302.24	-------	441.73	0.11	(34.77%) (1) ring A: N-H stretch (47,46)
					(33.03%) (20) ring B: N-H stretch (66,65)
					(29.51%) (47) ring C: N-H stretch (10, 9)
40	2244.29	-------	374.38	0.43	(58.25%) (1) ring A: N-H stretch (47,46)
					(6.21%) (20) ring B: N-H stretch (66,65)
					(33.09%) (47) ring C: N-H stretch (10, 9)
41	2232.11	-------	97.09	0.07	(59.11%) (20) ring B: N-H stretch (66,65)
					(35.51%) (47) ring C: N-H stretch (10, 9)
42	1779.83	-------	274.23	1.53	(85.35%) (19) ring A: C=O stretch (64,48)
43	1774.15	-------	188.98	0.04	(81.27%) (66) ring C-prop: C=O stretch (26,25)
					(6.41%) (199) ring C-prop: C-O-H BEND
44	1760.70	-------	254.70	0.02	(81.10%) (37) ring B-prop: C=O stretch (82,81)
					(6.53%) (161) ring B-prop: C-O-H BEND
45	1736.36	-------	866.43	4.88	(82.29%) (74) ring D: C=O stretch (33,45)
					(5.44%) (100) ring D bending
46	1616.67	-------	156.38	100.00	(7.98%) (52) bridge CD: C-C stretch (14, 5)
					(56.99%) (70) bridge CD: C=C stretch (30, 5)
					(5.44%) (71) ring D: C-N stretch (30,28)
					(10.99%) (166) bridge CD: C-H ROCK
47	1613.46	-------	8.61	2.31	(67.98%) (73) ring D: C=C stretch (32,31)
					(6.02%) (77) ring D-methyl: C-C stretch (34,31)
					(8.22%) (81) ring D-ethyl: C-C stretch (38,32)
48	1610.12	-------	617.66	26.99	(19.53%) (41) bridge BC: C-.C stretch (3,70)
					(22.82%) (44) bridge AB: C=C stretch (7,53)
					(15.94%) (48) bridge BC: C-.C stretch (11, 3)
					(10.84%) (163) bridge BC: C-H ROCK

49	1586.57	-------	1157.86	3.15	(24.46%)	(23)	ring B: C-.C stretch (69,68)
					(6.69%)	(41)	bridge BC: C-.C stretch (3,70)
					(18.35%)	(44)	bridge AB: C=C stretch (7,53)
					(8.01%)	(48)	bridge BC: C-.C stretch (11, 3)
					(7.96%)	(163)	bridge BC: C-H ROCK
					(5.61%)	(169)	bridge AB: C-H ROCK
50	1541.51	-------	1595.62	10.94	(7.16%)	(21)	ring B: C-N stretch (67,65)
					(26.23%)	(23)	ring B: C-.C stretch (69,68)
					(5.01%)	(26)	ring B-methyl: C-C stretch (71,68)
					(5.31%)	(41)	bridge BC: C-.C stretch (3,70)
					(10.93%)	(44)	bridge AB: C=C stretch (7,53)
					(9.67%)	(45)	bridge AB: C-C stretch (7,67)
					(7.11%)	(92)	ring B bending
51	1507.10	-------	154.84	1.95	(38.04%)	(51)	ring C: C-.C stretch (13,12)
					(6.61%)	(54)	ring C: C-C stretch (14,13)
					(10.35%)	(55)	ring C-methyl: C-C stretch (15,13)
52	1490.12	-------	64.50	8.33	(7.42%)	(54)	ring C: C-C stretch (14,13)
					(52.15%)	(182)	ring C-methyl: ADEFa
					(8.90%)	(184)	ring C-methyl: ROCKa
53	1486.87	-------	26.95	5.00	(21.65%)	(7)	ring A: C-N stretch (53,46)
					(10.69%)	(22)	ring B: C-C stretch (68,67)
					(5.17%)	(44)	bridge AB: C=C stretch (7,53)
					(13.13%)	(45)	bridge AB: C-C stretch (7,67)
					(16.81%)	(169)	bridge AB: C-H ROCK
54	1477.20	-------	7.64	0.19	(5.88%)	(216)	ring D-ethyl: CH2 SCIS
					(72.06%)	(221)	ring D-ethyl: CH3 ADEFa
					(10.27%)	(222)	ring D-ethyl: CH3 ADEFb
					(6.66%)	(223)	ring D-ethyl: CH3 ROCKa
55	1476.87	-------	79.09	0.25	(10.08%)	(145)	ring B-methyl: ADEFb
					(65.68%)	(149)	ring B-prop: 1-CH2-SCIS
56	1473.57	-------	5.12	0.05	(14.24%)	(124)	ring A-ethyl: CH2 SCIS
					(44.37%)	(129)	ring A-ethyl: CH3 ADEFa
					(25.76%)	(130)	ring A-ethyl: CH3 ADEFb
57	1468.65	-------	4.53	0.10	(20.84%)	(119)	ring A-methyl: ADEFa
					(38.98%)	(129)	ring A-ethyl: CH3 ADEFa
					(25.45%)	(130)	ring A-ethyl: CH3 ADEFb
58	1467.21	-------	10.94	0.14	(31.24%)	(119)	ring A-methyl: ADEFa
					(31.27%)	(120)	ring A-methyl: ADEFb
					(5.12%)	(124)	ring A-ethyl: CH2 SCIS
					(20.67%)	(130)	ring A-ethyl: CH3 ADEFb
59	1466.19	-------	2.05	2.04	(33.33%)	(183)	ring C-methyl: ADEFb
					(20.50%)	(187)	ring C-prop: 1-CH2-SCIS
					(13.45%)	(212)	ring D-methyl: ADEFb
60	1465.06	-------	4.55	0.12	(34.33%)	(119)	ring A-methyl: ADEFa
					(50.38%)	(120)	ring A-methyl: ADEFb
61	1463.95	-------	17.44	0.25	(6.37%)	(221)	ring D-ethyl: CH3 ADEFa
					(77.28%)	(222)	ring D-ethyl: CH3 ADEFb
					(7.16%)	(224)	ring D-ethyl: CH3 ROCKb
62	1462.76	-------	34.63	3.35	(8.29%)	(144)	ring B-methyl: ADEFa
					(6.47%)	(145)	ring B-methyl: ADEFb
					(7.47%)	(149)	ring B-prop: 1-CH2-SCIS
					(7.41%)	(183)	ring C-methyl: ADEFb
					(8.59%)	(211)	ring D-methyl: ADEFa
					(22.57%)	(212)	ring D-methyl: ADEFb
63	1458.38	-------	1.52	4.14	(29.96%)	(144)	ring B-methyl: ADEFa
					(17.63%)	(145)	ring B-methyl: ADEFb
					(13.94%)	(211)	ring D-methyl: ADEFa
64	1456.59	-------	16.81	0.12	(18.14%)	(187)	ring C-prop: 1-CH2-SCIS
					(35.81%)	(211)	ring D-methyl: ADEFa
					(25.50%)	(212)	ring D-methyl: ADEFb
65	1454.48	-------	1.83	0.18	(71.90%)	(124)	ring A-ethyl: CH2 SCIS
					(14.84%)	(130)	ring A-ethyl: CH3 ADEFb
66	1453.24	-------	20.44	0.91	(30.41%)	(144)	ring B-methyl: ADEFa
					(30.29%)	(145)	ring B-methyl: ADEFb
					(12.13%)	(183)	ring C-methyl: ADEFb
67	1452.44	-------	15.10	0.32	(9.95%)	(144)	ring B-methyl: ADEFa
					(13.41%)	(145)	ring B-methyl: ADEFb
					(29.63%)	(183)	ring C-methyl: ADEFb
					(24.04%)	(187)	ring C-prop: 1-CH2-SCIS
					(6.76%)	(212)	ring D-methyl: ADEFb
68	1450.30	-------	83.28	6.63	(18.57%)	(182)	ring C-methyl: ADEFa
					(10.74%)	(187)	ring C-prop: 1-CH2-SCIS
					(24.72%)	(211)	ring D-methyl: ADEFa

69	1444.88	-------	7.43	0.88	(67.98%)	(216)	ring D-ethyl: CH2 SCIS
					(8.40%)	(221)	ring D-ethyl: CH3 ADEFa
70	1443.60	-------	15.52	5.47	(12.33%)	(52)	bridge CD: C-C stretch (14, 5)
					(9.25%)	(54)	ring C: C-C stretch (14,13)
					(11.53%)	(216)	ring D-ethyl: CH2 SCIS
71	1438.19	-------	15.78	1.21	(80.94%)	(154)	ring B-prop: 2-CH2-SCIS
72	1435.87	-------	13.93	11.33	(20.89%)	(21)	ring B: C-N stretch (67,65)
					(7.02%)	(23)	ring B: C-.C stretch (69,68)
					(7.62%)	(45)	bridge AB: C-C stretch (7,67)
					(9.71%)	(154)	ring B-prop: 2-CH2-SCIS
					(20.62%)	(169)	bridge AB: C-H ROCK
73	1426.77	-------	146.18	1.17	(5.78%)	(48)	bridge BC: C-.C stretch (11, 3)
					(12.51%)	(49)	ring C: C-N stretch (11, 9)
					(6.74%)	(97)	ring C bending
					(14.97%)	(163)	bridge BC: C-H ROCK
					(20.49%)	(192)	ring C-prop: 2-CH2-SCIS
74	1423.14	-------	9.03	0.60	(7.89%)	(163)	bridge BC: C-H ROCK
					(64.20%)	(192)	ring C-prop: 2-CH2-SCIS
75	1410.65	-------	22.03	3.78	(7.48%)	(24)	ring B: C-N stretch (70,65)
					(7.76%)	(93)	ring B bending
					(6.62%)	(97)	ring C bending
					(6.25%)	(163)	bridge BC: C-H ROCK
					(5.52%)	(166)	bridge CD: C-H ROCK
76	1398.94	-------	52.09	4.07	(6.21%)	(65)	ring C-prop: C-C stretch (25,22)
					(6.19%)	(68)	ring C-prop: C-O stretch (27,25)
					(21.09%)	(194)	ring C-prop: 2-CH2-WAGG
					(5.73%)	(197)	ring C-prop: C=O ROCK
					(6.85%)	(199)	ring C-prop: C-O-H BEND
77	1398.67	-------	125.98	8.11	(8.69%)	(143)	ring B-methyl: SDEF
					(8.20%)	(166)	bridge CD: C-H ROCK
					(6.18%)	(192)	ring C-prop: 2-CH2-SCIS
					(10.58%)	(194)	ring C-prop: 2-CH2-WAGG
78	1394.98	-------	106.02	0.41	(10.60%)	(36)	ring B-prop: C-C stretch (81,78)
					(13.35%)	(38)	ring B-prop: C-O stretch (83,81)
					(24.85%)	(156)	ring B-prop: 2-CH2-WAGG
					(11.55%)	(159)	ring B-prop: C=O ROCK
					(16.67%)	(161)	ring B-prop: C-O-H BEND
79	1389.61	-------	9.96	2.28	(5.43%)	(77)	ring D-methyl: C-C stretch (34,31)
					(67.24%)	(210)	ring D-methyl: SDEF
80	1387.33	-------	20.10	3.93	(5.57%)	(166)	bridge CD: C-H ROCK
					(76.65%)	(181)	ring C-methyl: SDEF
81	1384.18	-------	1.19	0.02	(92.05%)	(128)	ring A-ethyl: CH3 SDEF
82	1380.24	-------	21.62	14.02	(66.01%)	(143)	ring B-methyl: SDEF
83	1376.44	-------	1.35	6.09	(48.44%)	(118)	ring A-methyl: SDEF
					(5.35%)	(166)	bridge CD: C-H ROCK
					(5.80%)	(181)	ring C-methyl: SDEF
					(5.53%)	(210)	ring D-methyl: SDEF
84	1374.35	-------	24.66	4.09	(45.24%)	(118)	ring A-methyl: SDEF
					(5.65%)	(210)	ring D-methyl: SDEF
					(9.45%)	(220)	ring D-ethyl: CH3 SDEF
85	1368.72	-------	8.66	8.66	(5.16%)	(166)	bridge CD: C-H ROCK
					(74.13%)	(220)	ring D-ethyl: CH3 SDEF
86	1354.60	-------	12.48	0.41	(6.44%)	(22)	ring B: C-C stretch (68,67)
					(7.84%)	(53)	ring C: C-N stretch (14, 9)
					(9.39%)	(126)	ring A-ethyl: CH2 WAGG
					(9.58%)	(189)	ring C-prop: 1-CH2-WAGG
87	1352.24	-------	10.74	0.64	(16.21%)	(112)	ring A-ethyl: SCIS
					(48.82%)	(126)	ring A-ethyl: CH2 WAGG
88	1342.67	-------	15.25	0.98	(5.14%)	(76)	ring D: C-C stretch (33,32)
					(7.12%)	(81)	ring D-ethyl: C-C stretch (38,32)
					(5.49%)	(101)	ring D bending
					(18.75%)	(189)	ring C-prop: 1-CH2-WAGG
					(25.98%)	(218)	ring D-ethyl: CH2 WAGG
89	1340.63	-------	26.05	1.37	(14.40%)	(108)	ring A-methyl: SCIS
					(9.89%)	(112)	ring A-ethyl: SCIS
					(7.94%)	(114)	ring A-ethyl: WAGG
					(10.28%)	(126)	ring A-ethyl: CH2 WAGG
					(10.13%)	(127)	ring A-ethyl: CH2 TWIST
					(6.02%)	(151)	ring B-prop: 1-CH2-WAGG
90	1337.36	-------	18.13	0.36	(45.21%)	(151)	ring B-prop: 1-CH2-WAGG
					(9.89%)	(156)	ring B-prop: 2-CH2-WAGG
					(9.70%)	(161)	ring B-prop: C-O-H BEND

91	1334.73	-------	29.61	0.64	(9.01%)	(53)	ring C: C-N stretch (14, 9)
					(36.36%)	(189)	ring C-prop: 1-CH2-WAGG
					(7.72%)	(218)	ring D-ethyl: CH2 WAGG
92	1317.85	-------	5.94	2.47	(10.09%)	(76)	ring D: C-C stretch (33,32)
					(7.72%)	(101)	ring D bending
					(41.29%)	(218)	ring D-ethyl: CH2 WAGG
					(10.38%)	(219)	ring D-ethyl: CH2 TWIST
93	1314.28	-------	26.30	0.03	(10.85%)	(190)	ring C-prop: 1-CH2-TWIST
					(23.84%)	(194)	ring C-prop: 2-CH2-WAGG
					(5.51%)	(197)	ring C-prop: C=O ROCK
					(38.08%)	(199)	ring C-prop: C-O-H BEND
94	1307.61	-------	15.51	0.05	(32.25%)	(108)	ring A-methyl: SCIS
					(5.76%)	(109)	ring A-methyl: ROCK
					(5.84%)	(112)	ring A-ethyl: SCIS
					(5.16%)	(121)	ring A-methyl: ROCKa
					(31.57%)	(127)	ring A-ethyl: CH2 TWIST
95	1294.16	-------	421.42	2.46	(18.55%)	(152)	ring B-prop: 1-CH2-TWIST
					(7.00%)	(161)	ring B-prop: C-O-H BEND
96	1290.71	-------	33.64	0.13	(26.41%)	(110)	ring A-methyl: WAGG
					(8.78%)	(111)	ring A-methyl: TWIST
					(10.52%)	(114)	ring A-ethyl: WAGG
					(5.54%)	(115)	ring A-ethyl: TWIST
					(5.42%)	(126)	ring A-ethyl: CH2 WAGG
97	1286.73	-------	203.53	1.65	(6.77%)	(151)	ring B-prop: 1-CH2-WAGG
					(12.89%)	(156)	ring B-prop: 2-CH2-WAGG
					(7.46%)	(157)	ring B-prop: 2-CH2-TWIST
					(14.45%)	(161)	ring B-prop: C-O-H BEND
98	1278.32	-------	0.74	0.08	(5.51%)	(53)	ring C: C-N stretch (14, 9)
					(43.10%)	(219)	ring D-ethyl: CH2 TWIST
					(10.60%)	(224)	ring D-ethyl: CH3 ROCKb
99	1260.28	-------	44.62	2.61	(25.86%)	(71)	ring D: C-N stretch (30,28)
					(6.88%)	(166)	bridge CD: C-H ROCK
					(6.26%)	(208)	ring D: C=O ROCK
					(12.36%)	(219)	ring D-ethyl: CH2 TWIST
100	1256.63	-------	69.79	0.25	(7.27%)	(108)	ring A-methyl: SCIS
					(16.48%)	(112)	ring A-ethyl: SCIS
					(5.22%)	(113)	ring A-ethyl: ROCK
					(7.25%)	(125)	ring A-ethyl: CH2 ROCK
					(24.35%)	(127)	ring A-ethyl: CH2 TWIST
					(13.24%)	(131)	ring A-ethyl: CH3 ROCKa
101	1253.98	-------	26.04	0.56	(13.26%)	(151)	ring B-prop: 1-CH2-WAGG
					(20.16%)	(152)	ring B-prop: 1-CH2-TWIST
					(20.05%)	(156)	ring B-prop: 2-CH2-WAGG
					(12.74%)	(157)	ring B-prop: 2-CH2-TWIST
102	1242.33	-------	314.34	0.07	(11.53%)	(110)	ring A-methyl: WAGG
					(6.02%)	(111)	ring A-methyl: TWIST
					(7.05%)	(112)	ring A-ethyl: SCIS
					(11.23%)	(114)	ring A-ethyl: WAGG
					(7.17%)	(115)	ring A-ethyl: TWIST
					(13.15%)	(190)	ring C-prop: 1-CH2-TWIST
103	1237.21	-------	68.30	0.57	(5.21%)	(114)	ring A-ethyl: WAGG
					(5.03%)	(189)	ring C-prop: 1-CH2-WAGG
					(24.37%)	(190)	ring C-prop: 1-CH2-TWIST
					(6.30%)	(194)	ring C-prop: 2-CH2-WAGG
					(11.27%)	(195)	ring C-prop: 2-CH2-TWIST
104	1226.29	-------	490.73	1.74	(12.56%)	(2)	ring A: C-N stretch (48,46)
					(6.19%)	(3)	ring A: C-C stretch (49,48)
					(7.02%)	(88)	ring A bending
					(5.35%)	(106)	ring A: C=O ROCK
					(6.21%)	(157)	ring B-prop: 2-CH2-TWIST
					(5.38%)	(169)	bridge AB: C-H ROCK
105	1216.82	-------	159.67	0.55	(16.93%)	(2)	ring A: C-N stretch (48,46)
					(14.47%)	(7)	ring A: C-N stretch (53,46)
					(5.08%)	(21)	ring B: C-N stretch (67,65)
					(11.66%)	(44)	bridge AB: C=C stretch (7,53)
					(10.73%)	(104)	ring A: N-H ROCK
					(15.35%)	(169)	bridge AB: C-H ROCK
106	1211.33	-------	102.75	2.58	(6.48%)	(55)	ring C-methyl: C-C stretch (15,13)
					(12.32%)	(75)	ring D: C-N stretch (33,28)
					(7.89%)	(166)	bridge CD: C-H ROCK
					(29.08%)	(195)	ring C-prop: 2-CH2-TWIST
107	1202.64	-------	85.28	0.38	(16.66%)	(152)	ring B-prop: 1-CH2-TWIST
					(21.41%)	(157)	ring B-prop: 2-CH2-TWIST
					(5.48%)	(161)	ring B-prop: C-O-H BEND
					(5.62%)	(190)	ring C-prop: 1-CH2-TWIST
					(13.10%)	(195)	ring C-prop: 2-CH2-TWIST

108	1191.49	-------	685.98	3.99	(5.87%) (75) ring D: C-N stretch (33,28)
					(6.25%) (190) ring C-prop: 1-CH2-TWIST
					(9.05%) (195) ring C-prop: 2-CH2-TWIST
109	1160.00	-------	87.14	0.69	(12.25%) (49) ring C: C-N stretch (11, 9)
					(5.91%) (55) ring C-methyl: C-C stretch (15,13)
					(5.46%) (59) ring C-prop: C-C stretch (19,12)
					(17.95%) (75) ring D: C-N stretch (33,28)
					(6.80%) (97) ring C bending
110	1153.26	-------	182.63	0.07	(7.07%) (38) ring B-prop: C-O stretch (83,81)
					(17.82%) (68) ring C-prop: C-O stretch (27,25)
					(8.57%) (199) ring C-prop: C-O-H BEND
111	1150.82	-------	191.52	1.26	(18.62%) (38) ring B-prop: C-O stretch (83,81)
					(6.78%) (157) ring B-prop: 2-CH2-TWIST
					(8.25%) (161) ring B-prop: C-O-H BEND
112	1147.28	-------	148.83	0.02	(5.45%) (38) ring B-prop: C-O stretch (83,81)
					(11.75%) (68) ring C-prop: C-O stretch (27,25)
					(8.52%) (125) ring A-ethyl: CH2 ROCK
					(5.42%) (199) ring C-prop: C-O-H BEND
113	1139.36	-------	18.31	0.28	(5.41%) (71) ring D: C-N stretch (30,28)
					(14.50%) (77) ring D-methyl: C-C stretch (34,31)
					(11.54%) (217) ring D-ethyl: CH2 ROCK
					(9.32%) (224) ring D-ethyl: CH3 ROCKb
114	1137.89	-------	56.80	1.06	(16.03%) (24) ring B: C-N stretch (70,65)
					(6.51%) (30) ring B-prop: C-C stretch (75,69)
					(6.86%) (133) ring B: N-H ROCK
115	1127.23	-------	82.44	0.28	(5.63%) (3) ring A: C-C stretch (49,48)
					(9.38%) (8) ring A: C-C stretch (53,51)
					(11.46%) (104) ring A: N-H ROCK
					(5.90%) (115) ring A-ethyl: TWIST
116	1126.26	-------	115.78	0.05	(6.37%) (59) ring C-prop: C-C stretch (19,12)
					(5.20%) (96) ring C bending
					(19.62%) (184) ring C-methyl: ROCKa
117	1116.52	-------	83.10	0.22	(13.38%) (81) ring D-ethyl: C-C stretch (38,32)
					(13.20%) (213) ring D-methyl: ROCKa
					(11.34%) (214) ring D-methyl: ROCKb
					(12.25%) (217) ring D-ethyl: CH2 ROCK
					(11.46%) (224) ring D-ethyl: CH3 ROCKb
118	1097.89	-------	56.34	0.28	(5.40%) (26) ring B-methyl: C-C stretch (71,68)
					(5.79%) (92) ring B bending
					(5.60%) (104) ring A: N-H ROCK
					(11.09%) (132) ring A-ethyl: CH3 ROCKb
					(5.61%) (147) ring B-methyl: ROCKb
119	1093.91	-------	38.95	2.42	(6.02%) (5) ring A: C-C stretch (51,49)
					(7.70%) (13) ring A-ethyl: C-C stretch (58,51)
					(5.14%) (104) ring A: N-H ROCK
					(5.25%) (111) ring A-methyl: TWIST
120	1086.61	-------	21.23	0.98	(5.36%) (30) ring B-prop: C-C stretch (75,69)
					(8.37%) (122) ring A-methyl: ROCKb
					(9.07%) (133) ring B: N-H ROCK
					(5.63%) (147) ring B-methyl: ROCKb
					(6.22%) (184) ring C-methyl: ROCKa
121	1076.68	-------	4.60	1.88	(9.78%) (9) ring A-methyl: C-C stretch (54,49)
					(11.85%) (122) ring A-methyl: ROCKb
					(13.54%) (132) ring A-ethyl: CH3 ROCKb
122	1063.79	-------	66.40	1.12	(13.69%) (84) ring D-ethyl: C-C stretch (41,38)
					(12.87%) (213) ring D-methyl: ROCKa
					(9.31%) (215) ring D-ethyl: BEND
					(44.25%) (223) ring D-ethyl: CH3 ROCKa
123	1060.40	-------	0.41	5.93	(21.10%) (133) ring B: N-H ROCK
					(5.92%) (162) bridge BC: BEND
					(13.45%) (171) ring C: N-H ROCK
124	1053.09	-------	4.59	0.72	(7.00%) (178) ring C-methyl: WAGG
					(7.24%) (183) ring C-methyl: ADEFb
					(76.38%) (185) ring C-methyl: ROCKb
125	1046.87	-------	3.90	0.07	(8.35%) (138) ring B-methyl: WAGG
					(5.46%) (144) ring B-methyl: ADEFa
					(63.43%) (146) ring B-methyl: ROCKa
					(11.83%) (147) ring B-methyl: ROCKb
126	1045.62	-------	33.51	0.69	(12.12%) (147) ring B-methyl: ROCKb
					(10.35%) (150) ring B-prop: 1-CH2-ROCK
					(6.87%) (155) ring B-prop: 2-CH2-ROCK
					(10.09%) (188) ring C-prop: 1-CH2-ROCK
					(5.40%) (193) ring C-prop: 2-CH2-ROCK

127	1040.11	-------	0.72	0.42	(7.39%)	(205)	ring D-methyl: WAGG
					(21.97%)	(213)	ring D-methyl: ROCKa
					(51.80%)	(214)	ring D-methyl: ROCKb
					(7.02%)	(223)	ring D-ethyl: CH3 ROCKa
128	1026.41	-------	2.96	0.07	(8.03%)	(13)	ring A-ethyl: C-C stretch (58,51)
					(10.90%)	(15)	ring A-ethyl: C-C stretch (60,58)
					(8.15%)	(109)	ring A-methyl: ROCK
					(27.36%)	(121)	ring A-methyl: ROCKa
					(6.06%)	(125)	ring A-ethyl: CH2 ROCK
					(8.19%)	(127)	ring A-ethyl: CH2 TWIST
129	1024.14	-------	0.44	0.08	(5.37%)	(5)	ring A: C-C stretch (51,49)
					(6.79%)	(9)	ring A-methyl: C-C stretch (54,49)
					(11.49%)	(13)	ring A-ethyl: C-C stretch (58,51)
					(25.20%)	(15)	ring A-ethyl: C-C stretch (60,58)
					(6.20%)	(112)	ring A-ethyl: SCIS
					(15.01%)	(122)	ring A-methyl: ROCKb
					(8.74%)	(131)	ring A-ethyl: CH3 ROCKa
130	1019.40	-------	43.32	0.82	(6.24%)	(150)	ring B-prop: 1-CH2-ROCK
					(5.99%)	(155)	ring B-prop: 2-CH2-ROCK
					(27.18%)	(171)	ring C: N-H ROCK
					(7.81%)	(193)	ring C-prop: 2-CH2-ROCK
131	998.66	-------	112.60	0.14	(5.13%)	(2)	ring A: C-N stretch (48,46)
					(21.88%)	(9)	ring A-methyl: C-C stretch (54,49)
					(15.85%)	(15)	ring A-ethyl: C-C stretch (60,58)
					(8.07%)	(104)	ring A: N-H ROCK
					(6.58%)	(122)	ring A-methyl: ROCKb
132	997.83	-------	76.78	0.34	(5.04%)	(51)	ring C: C-.C stretch (13,12)
					(39.75%)	(62)	ring C-prop: C-C stretch (22,19)
					(13.77%)	(184)	ring C-methyl: ROCKa
133	995.41	-------	14.83	0.17	(67.49%)	(33)	ring B-prop: C-C stretch (78,75)
					(6.08%)	(148)	ring B-prop: 1-BEND
					(5.44%)	(153)	ring B-prop: 2-BEND
					(5.16%)	(160)	ring B-prop: C=O OUT
134	993.52	-------	2.98	2.81	(7.97%)	(71)	ring D: C-N stretch (30,28)
					(17.33%)	(76)	ring D: C-C stretch (33,32)
					(14.15%)	(77)	ring D-methyl: C-C stretch (34,31)
					(14.42%)	(200)	ring D: N-H ROCK
					(7.30%)	(219)	ring D-ethyl: CH2 TWIST
					(8.28%)	(224)	ring D-ethyl: CH3 ROCKb
135	981.81	-------	63.04	0.55	(5.90%)	(72)	ring D: C-C stretch (31,30)
					(5.05%)	(73)	ring D: C=C stretch (32,31)
					(6.68%)	(81)	ring D-ethyl: C-C stretch (38,32)
					(24.16%)	(84)	ring D-ethyl: C-C stretch (41,38)
					(12.18%)	(200)	ring D: N-H ROCK
					(13.52%)	(213)	ring D-methyl: ROCKa
					(6.50%)	(214)	ring D-methyl: ROCKb
136	952.62	-------	143.15	0.06	(5.92%)	(22)	ring B: C-C stretch (68,67)
					(7.61%)	(23)	ring B: C-.C stretch (69,68)
					(15.40%)	(147)	ring B-methyl: ROCKb
					(9.80%)	(150)	ring B-prop: 1-CH2-ROCK
					(8.66%)	(155)	ring B-prop: 2-CH2-ROCK
137	947.75	-------	44.44	0.57	(6.48%)	(186)	ring C-prop: 1-BEND
					(13.14%)	(188)	ring C-prop: 1-CH2-ROCK
					(7.40%)	(191)	ring C-prop: 2-BEND
					(21.12%)	(193)	ring C-prop: 2-CH2-ROCK
					(12.66%)	(198)	ring C-prop: C=O OUT
138	925.78	-------	7.33	1.18	(5.49%)	(5)	ring A: C-C stretch (51,49)
					(8.03%)	(15)	ring A-ethyl: C-C stretch (60,58)
					(5.08%)	(62)	ring C-prop: C-C stretch (22,19)
					(8.00%)	(84)	ring D-ethyl: C-C stretch (41,38)
					(7.85%)	(132)	ring A-ethyl: CH3 ROCKb
					(7.92%)	(193)	ring C-prop: 2-CH2-ROCK
139	923.46	-------	46.29	0.71	(5.75%)	(5)	ring A: C-C stretch (51,49)
					(8.75%)	(15)	ring A-ethyl: C-C stretch (60,58)
					(24.23%)	(84)	ring D-ethyl: C-C stretch (41,38)
					(8.13%)	(132)	ring A-ethyl: CH3 ROCKb
					(6.87%)	(223)	ring D-ethyl: CH3 ROCKa
140	917.44	-------	318.29	0.23	(15.47%)	(84)	ring D-ethyl: C-C stretch (41,38)
					(5.92%)	(188)	ring C-prop: 1-CH2-ROCK
					(5.82%)	(193)	ring C-prop: 2-CH2-ROCK
					(5.39%)	(223)	ring D-ethyl: CH3 ROCKa
141	908.49	-------	44.61	0.43	(21.61%)	(36)	ring B-prop: C-C stretch (81,78)
					(6.76%)	(38)	ring B-prop: C-O stretch (83,81)
					(5.46%)	(148)	ring B-prop: 1-BEND
					(6.68%)	(153)	ring B-prop: 2-BEND
					(12.08%)	(155)	ring B-prop: 2-CH2-ROCK
					(9.86%)	(160)	ring B-prop: C=O OUT
142	886.69	-------	28.38	0.36	(5.45%)	(5)	ring A: C-C stretch (51,49)

					(7.44%) (7) ring A: C-N stretch (53,46)
					(14.41%) (8) ring A: C-C stretch (53,51)
					(6.66%) (13) ring A-ethyl: C-C stretch (58,51)
					(7.01%) (15) ring A-ethyl: C-C stretch (60,58)
					(6.76%) (104) ring A: N-H ROCK
					(10.41%) (164) bridge BC: C-H OUT
143	885.40	-------	11.66	0.73	(58.16%) (164) bridge BC: C-H OUT
					(9.96%) (233) bridge BC: 1-TORS
					(7.39%) (236) bridge BC: 2-TORS
144	853.57	-------	10.01	0.26	(5.51%) (7) ring A: C-N stretch (53,46)
					(5.85%) (36) ring B-prop: C-C stretch (81,78)
					(7.00%) (104) ring A: N-H ROCK
					(11.61%) (133) ring B: N-H ROCK
145	850.15	-------	45.23	0.08	(5.04%) (8) ring A: C-C stretch (53,51)
					(5.05%) (13) ring A-ethyl: C-C stretch (58,51)
					(5.89%) (113) ring A-ethyl: ROCK
					(16.81%) (131) ring A-ethyl: CH3 ROCKa
146	837.94	-------	57.42	3.65	(9.62%) (54) ring C: C-C stretch (14,13)
					(12.97%) (171) ring C: N-H ROCK
					(6.41%) (200) ring D: N-H ROCK
147	818.91	-------	26.17	46.59	(59.01%) (167) bridge CD: C-H OUT
					(9.68%) (203) ring D: bridge CD WAGG
					(11.39%) (243) bridge C=D: TORS
148	814.89	-------	17.60	3.08	(10.66%) (72) ring D: C-C stretch (31,30)
					(5.04%) (206) ring D-ethyl: ROCK
					(5.40%) (213) ring D-methyl: ROCKa
					(11.95%) (217) ring D-ethyl: CH2 ROCK
					(16.58%) (224) ring D-ethyl: CH3 ROCKb
149	801.55	-------	8.96	0.82	(9.16%) (36) ring B-prop: C-C stretch (81,78)
					(7.67%) (65) ring C-prop: C-C stretch (25,22)
					(8.67%) (150) ring B-prop: 1-CH2-ROCK
					(6.79%) (155) ring B-prop: 2-CH2-ROCK
150	798.02	-------	15.00	0.13	(5.40%) (136) ring B: bridge AB WAGG
					(57.72%) (170) bridge AB: C-H OUT
					(11.95%) (234) bridge A=B: TORS
					(7.24%) (235) bridge A-B: TORS
151	795.50	-------	3.15	0.16	(6.64%) (2) ring A: C-N stretch (48,46)
					(22.39%) (3) ring A: C-C stretch (49,48)
					(9.68%) (107) ring A: C=O OUT
					(6.88%) (121) ring A-methyl: ROCKa
					(6.54%) (122) ring A-methyl: ROCKb
					(6.08%) (170) bridge AB: C-H OUT
152	788.10	-------	5.97	0.48	(23.14%) (65) ring C-prop: C-C stretch (25,22)
					(9.88%) (68) ring C-prop: C-O stretch (27,25)
					(5.73%) (150) ring B-prop: 1-CH2-ROCK
					(10.45%) (188) ring C-prop: 1-CH2-ROCK
153	776.31	-------	16.44	0.32	(6.45%) (102) ring D torsion
					(6.40%) (165) bridge CD: BEND
					(6.40%) (207) ring D-ethyl: WAGG
					(24.89%) (209) ring D: C=O OUT
154	769.77	-------	1.90	0.22	(6.23%) (207) ring D-ethyl: WAGG
					(24.39%) (209) ring D: C=O OUT
155	764.61	-------	35.99	1.33	(10.68%) (96) ring C bending
					(9.65%) (125) ring A-ethyl: CH2 ROCK
					(7.36%) (150) ring B-prop: 1-CH2-ROCK
156	756.00	-------	9.42	0.41	(5.31%) (5) ring A: C-C stretch (51,49)
					(26.16%) (125) ring A-ethyl: CH2 ROCK
					(5.24%) (131) ring A-ethyl: CH3 ROCKa
					(6.41%) (132) ring A-ethyl: CH3 ROCKb
157	750.47	-------	11.94	0.56	(6.17%) (76) ring D: C-C stretch (33,32)
					(10.29%) (100) ring D bending
					(5.08%) (209) ring D: C=O OUT
					(23.93%) (217) ring D-ethyl: CH2 ROCK
					(11.37%) (224) ring D-ethyl: CH3 ROCKb
158	735.36	-------	12.01	0.13	(6.91%) (88) ring A bending
					(5.95%) (217) ring D-ethyl: CH2 ROCK
159	730.89	-------	19.06	0.44	(13.80%) (94) ring B torsion
					(12.37%) (140) ring B-prop: 1-WAGG
					(8.18%) (142) ring B: bridge BC WAGG
					(5.51%) (148) ring B-prop: 1-BEND
					(7.29%) (160) ring B-prop: C=O OUT
					(7.26%) (232) ring B-prop: 4-TORS
160	719.16	-------	20.53	0.15	(8.56%) (98) ring C torsion
					(6.99%) (174) ring C: bridge BC WAGG
					(7.96%) (176) ring C-prop: 1-WAGG

161	715.57	-------	23.09	10.78	(5.50%)	(75)	ring D: C-N stretch (33,28)
					(7.75%)	(96)	ring C bending
					(26.63%)	(100)	ring D bending
					(5.81%)	(217)	ring D-ethyl: CH2 ROCK
162	706.83	-------	4.24	0.39	(13.04%)	(95)	ring B torsion
					(26.97%)	(136)	ring B: bridge AB WAGG
					(7.84%)	(232)	ring B-prop: 4-TORS
					(7.10%)	(234)	bridge A=B: TORS
163	698.11	-------	127.42	0.25	(6.41%)	(92)	ring B bending
					(5.90%)	(150)	ring B-prop: 1-CH2-ROCK
					(10.23%)	(155)	ring B-prop: 2-CH2-ROCK
					(7.14%)	(160)	ring B-prop: C=O OUT
					(34.76%)	(232)	ring B-prop: 4-TORS
164	691.98	-------	30.64	0.56	(9.38%)	(2)	ring A: C-N stretch (48,46)
					(6.91%)	(9)	ring A-methyl: C-C stretch (54,49)
					(6.03%)	(92)	ring B bending
					(5.51%)	(105)	ring A: N-H OUT
					(24.82%)	(107)	ring A: C=O OUT
					(5.34%)	(232)	ring B-prop: 4-TORS
165	684.55	-------	43.35	2.44	(9.93%)	(98)	ring C torsion
					(9.59%)	(198)	ring C-prop: C=O OUT
					(40.08%)	(242)	ring C-prop: 4-TORS
166	680.02	-------	30.44	0.56	(5.20%)	(99)	ring C torsion
					(8.37%)	(107)	ring A: C=O OUT
					(10.74%)	(242)	ring C-prop: 4-TORS
167	672.92	-------	49.67	0.21	(5.22%)	(142)	ring B: bridge BC WAGG
					(5.74%)	(232)	ring B-prop: 4-TORS
					(10.01%)	(242)	ring C-prop: 4-TORS
168	668.00	-------	6.21	1.08	(10.30%)	(55)	ring C-methyl: C-C stretch (15,13)
					(14.99%)	(99)	ring C torsion
					(8.70%)	(100)	ring D bending
					(6.80%)	(174)	ring C: bridge BC WAGG
169	662.22	-------	6.21	0.27	(5.32%)	(8)	ring A: C-C stretch (53,51)
					(7.76%)	(88)	ring A bending
					(5.38%)	(105)	ring A: N-H OUT
					(29.62%)	(117)	ring A: bridge AB WAGG
170	652.31	-------	32.80	26.59	(8.14%)	(102)	ring D torsion
					(16.52%)	(103)	ring D torsion
					(26.63%)	(203)	ring D: bridge CD WAGG
					(14.08%)	(205)	ring D-methyl: WAGG
					(9.95%)	(209)	ring D: C=O OUT
					(5.83%)	(243)	bridge C=D: TORS
171	644.51	-------	2.90	2.86	(6.52%)	(72)	ring D: C-C stretch (31,30)
					(6.78%)	(75)	ring D: C-N stretch (33,28)
					(6.20%)	(76)	ring D: C-C stretch (33,32)
					(10.86%)	(81)	ring D-ethyl: C-C stretch (38,32)
					(6.03%)	(203)	ring D: bridge CD WAGG
					(6.64%)	(208)	ring D: C=O ROCK
172	630.11	-------	22.64	0.64	(10.96%)	(26)	ring B-methyl: C-C stretch (71,68)
					(8.78%)	(93)	ring B bending
					(18.99%)	(159)	ring B-prop: C=O ROCK
					(6.98%)	(232)	ring B-prop: 4-TORS
173	618.39	-------	51.89	0.25	(9.30%)	(159)	ring B-prop: C=O ROCK
					(17.04%)	(197)	ring C-prop: C=O ROCK
					(8.10%)	(242)	ring C-prop: 4-TORS
174	612.05	-------	10.33	0.32	(12.99%)	(88)	ring A bending
					(27.17%)	(89)	ring A bending
					(10.17%)	(105)	ring A: N-H OUT
					(8.50%)	(117)	ring A: bridge AB WAGG
					(5.79%)	(197)	ring C-prop: C=O ROCK
175	604.80	-------	15.10	0.87	(7.99%)	(93)	ring B bending
					(7.50%)	(159)	ring B-prop: C=O ROCK
					(22.52%)	(197)	ring C-prop: C=O ROCK
176	593.95	-------	18.16	0.18	(7.27%)	(26)	ring B-methyl: C-C stretch (71,68)
					(7.93%)	(55)	ring C-methyl: C-C stretch (15,13)
					(5.82%)	(59)	ring C-prop: C-C stretch (19,12)
					(12.67%)	(93)	ring B bending
					(17.84%)	(97)	ring C bending
177	584.08	-------	64.52	0.01	(35.96%)	(105)	ring A: N-H OUT
					(21.64%)	(134)	ring B: N-H OUT
					(13.54%)	(172)	ring C: N-H OUT
					(5.51%)	(234)	bridge A=B: TORS
178	567.44	-------	27.18	0.09	(8.94%)	(77)	ring D-methyl: C-C stretch (34,31)
					(8.14%)	(101)	ring D bending

179	559.75	-------	15.38	0.18	(5.59%)	(77)	ring D-methyl: C-C stretch (34,31)
					(8.25%)	(101)	ring D bending
					(5.86%)	(148)	ring B-prop: 1-BEND
					(7.22%)	(159)	ring B-prop: C=O ROCK
					(12.79%)	(160)	ring B-prop: C=O OUT
					(9.79%)	(232)	ring B-prop: 4-TORS
180	550.89	-------	4.14	0.51	(11.71%)	(101)	ring D bending
					(11.04%)	(105)	ring A: N-H OUT
					(6.54%)	(134)	ring B: N-H OUT
					(18.75%)	(172)	ring C: N-H OUT
181	544.47	-------	18.48	1.40	(7.42%)	(101)	ring D bending
					(12.72%)	(105)	ring A: N-H OUT
					(13.74%)	(134)	ring B: N-H OUT
					(20.85%)	(172)	ring C: N-H OUT
					(5.81%)	(208)	ring D: C=O ROCK
182	523.78	-------	7.91	1.00	(50.10%)	(134)	ring B: N-H OUT
					(41.37%)	(172)	ring C: N-H OUT
183	522.46	-------	22.68	1.97	(6.74%)	(172)	ring C: N-H OUT
					(5.02%)	(174)	ring C: bridge BC WAGG
					(6.77%)	(196)	ring C-prop: 3-BEND
					(5.56%)	(233)	bridge BC: 1-TORS
184	515.58	-------	8.43	0.30	(8.37%)	(105)	ring A: N-H OUT
					(11.17%)	(106)	ring A: C=O ROCK
					(8.38%)	(134)	ring B: N-H OUT
					(5.15%)	(193)	ring C-prop: 2-CH2-ROCK
					(10.27%)	(198)	ring C-prop: C=O OUT
185	505.66	-------	29.25	0.32	(8.61%)	(106)	ring A: C=O ROCK
					(7.79%)	(193)	ring C-prop: 2-CH2-ROCK
					(11.80%)	(198)	ring C-prop: C=O OUT
186	498.21	-------	10.91	3.13	(8.72%)	(106)	ring A: C=O ROCK
					(9.05%)	(137)	ring B-methyl: ROCK
					(6.32%)	(196)	ring C-prop: 3-BEND
187	492.30	-------	6.35	8.49	(8.12%)	(101)	ring D bending
					(6.26%)	(203)	ring D: bridge CD WAGG
					(5.85%)	(205)	ring D-methyl: WAGG
					(16.23%)	(207)	ring D-ethyl: WAGG
					(6.03%)	(208)	ring D: C=O ROCK
					(8.77%)	(209)	ring D: C=O OUT
					(7.59%)	(215)	ring D-ethyl: BEND
188	488.79	-------	10.02	1.06	(9.66%)	(196)	ring C-prop: 3-BEND
					(11.15%)	(198)	ring C-prop: C=O OUT
189	480.04	-------	7.16	2.13	(6.05%)	(142)	ring B: bridge BC WAGG
					(5.21%)	(196)	ring C-prop: 3-BEND
190	439.60	-------	2.17	0.17	(6.43%)	(5)	ring A: C-C stretch (51,49)
					(5.27%)	(9)	ring A-methyl: C-C stretch (54,49)
					(5.52%)	(13)	ring A-ethyl: C-C stretch (58,51)
					(9.82%)	(109)	ring A-methyl: ROCK
					(10.11%)	(113)	ring A-ethyl: ROCK
					(5.36%)	(123)	ring A-ethyl: BEND
191	430.99	-------	12.20	0.36	(6.88%)	(155)	ring B-prop: 2-CH2-ROCK
					(29.17%)	(158)	ring B-prop: 3-BEND
					(5.09%)	(174)	ring C: bridge BC WAGG
					(6.12%)	(196)	ring C-prop: 3-BEND
192	404.51	-------	6.93	0.94	(5.62%)	(136)	ring B: bridge AB WAGG
					(7.46%)	(138)	ring B-methyl: WAGG
					(12.69%)	(158)	ring B-prop: 3-BEND
					(6.48%)	(174)	ring C: bridge BC WAGG
					(5.57%)	(180)	ring C: bridge CD WAGG
					(5.40%)	(196)	ring C-prop: 3-BEND
193	399.03	-------	7.13	0.08	(5.38%)	(88)	ring A bending
					(8.50%)	(106)	ring A: C=O ROCK
					(17.75%)	(123)	ring A-ethyl: BEND
194	363.47	-------	6.68	0.61	(8.20%)	(103)	ring D torsion
					(8.56%)	(179)	ring C: bridge CD ROCK
					(11.85%)	(205)	ring D-methyl: WAGG
					(8.30%)	(215)	ring D-ethyl: BEND
195	350.28	-------	1.56	1.07	(6.19%)	(175)	ring C-prop: 1-ROCK
					(28.04%)	(178)	ring C-methyl: WAGG
					(9.46%)	(186)	ring C-prop: 1-BEND
					(14.80%)	(196)	ring C-prop: 3-BEND
					(5.23%)	(205)	ring D-methyl: WAGG
196	343.16	-------	2.47	1.05	(5.74%)	(123)	ring A-ethyl: BEND
					(12.61%)	(137)	ring B-methyl: ROCK
					(6.75%)	(138)	ring B-methyl: WAGG
					(23.31%)	(139)	ring B-prop: 1-ROCK
					(6.29%)	(153)	ring B-prop: 2-BEND

197	326.75	-------	10.10	12.13	(5.28%) (99) ring C torsion
					(22.92%) (138) ring B-methyl: WAGG
					(15.48%) (201) ring D: N-H OUT
					(5.20%) (233) bridge BC: 1-TORS
198	323.96	-------	15.97	1.97	(7.25%) (138) ring B-methyl: WAGG
					(9.81%) (139) ring B-prop: 1-ROCK
					(6.87%) (158) ring B-prop: 3-BEND
					(8.16%) (175) ring C-prop: 1-ROCK
					(17.29%) (201) ring D: N-H OUT
199	317.44	-------	2.14	0.51	(40.88%) (204) ring D-methyl: ROCK
					(14.42%) (205) ring D-methyl: WAGG
					(7.68%) (208) ring D: C=O ROCK
					(-6.93%) (237) bridge C-D: TORS
200	313.28	-------	15.74	12.95	(-15.10%) (103) ring D torsion
					(5.12%) (175) ring C-prop: 1-ROCK
					(5.29%) (177) ring C-methyl: ROCK
					(66.87%) (201) ring D: N-H OUT
					(11.62%) (203) ring D: bridge CD WAGG
201	304.36	-------	3.73	6.13	(10.56%) (95) ring B torsion
					(20.86%) (177) ring C-methyl: ROCK
					(5.04%) (206) ring D-ethyl: ROCK
					(11.84%) (243) bridge C=D: TORS
					(6.29%) (246) ring D-ethyl: 2-TORS
202	294.18	-------	0.42	1.37	(14.55%) (178) ring C-methyl: WAGG
					(13.96%) (206) ring D-ethyl: ROCK
					(25.56%) (246) ring D-ethyl: 2-TORS
203	291.86	-------	3.07	9.43	(5.73%) (175) ring C-prop: 1-ROCK
					(7.54%) (177) ring C-methyl: ROCK
					(5.07%) (191) ring C-prop: 2-BEND
					(9.91%) (204) ring D-methyl: ROCK
204	287.07	-------	3.39	8.42	(5.51%) (99) ring C torsion
					(5.65%) (123) ring A-ethyl: BEND
					(5.43%) (178) ring C-methyl: WAGG
					(7.12%) (206) ring D-ethyl: ROCK
					(8.76%) (246) ring D-ethyl: 2-TORS
205	279.72	-------	2.29	4.25	(5.54%) (111) ring A-methyl: TWIST
					(14.60%) (123) ring A-ethyl: BEND
					(5.29%) (137) ring B-methyl: ROCK
					(5.16%) (227) ring A-ethyl: 2-TORS
206	269.24	-------	0.60	0.16	(5.06%) (173) ring C: bridge BC ROCK
					(7.44%) (177) ring C-methyl: ROCK
					(22.38%) (227) ring A-ethyl: 2-TORS
207	255.96	-------	2.53	0.56	(9.24%) (109) ring A-methyl: ROCK
					(8.82%) (225) ring A-methyl: TORS
					(23.45%) (227) ring A-ethyl: 2-TORS
208	247.80	-------	1.66	0.26	(6.70%) (110) ring A-methyl: WAGG
					(7.26%) (137) ring B-methyl: ROCK
					(6.33%) (138) ring B-methyl: WAGG
					(23.59%) (225) ring A-methyl: TORS
209	237.32	-------	0.36	0.13	(11.45%) (111) ring A-methyl: TWIST
					(60.33%) (225) ring A-methyl: TORS
210	233.27	-------	2.29	0.62	(6.79%) (202) ring D: bridge CD ROCK
					(11.15%) (227) ring A-ethyl: 2-TORS
					(5.96%) (234) bridge A=B: TORS
211	224.50	-------	2.25	0.75	(6.95%) (135) ring B: bridge AB ROCK
					(5.23%) (137) ring B-methyl: ROCK
					(5.12%) (227) ring A-ethyl: 2-TORS
					(39.51%) (238) ring C-methyl: TORS
					(5.04%) (244) ring D-methyl: TORS
212	219.78	-------	1.00	0.86	(33.97%) (238) ring C-methyl: TORS
					(7.89%) (244) ring D-methyl: TORS
					(8.78%) (246) ring D-ethyl: 2-TORS
213	215.77	-------	1.16	0.58	(9.32%) (135) ring B: bridge AB ROCK
					(9.93%) (137) ring B-methyl: ROCK
					(10.45%) (153) ring B-prop: 2-BEND
					(5.64%) (206) ring D-ethyl: ROCK
					(12.41%) (246) ring D-ethyl: 2-TORS
214	209.19	-------	2.47	0.62	(6.37%) (176) ring C-prop: 1-WAGG
					(9.58%) (206) ring D-ethyl: ROCK
					(13.78%) (246) ring D-ethyl: 2-TORS
215	196.54	-------	4.42	1.90	(7.48%) (91) ring A torsion
					(7.18%) (141) ring B: bridge BC ROCK
					(6.43%) (235) bridge A-B: TORS
					(5.55%) (246) ring D-ethyl: 2-TORS

					(11.21%) (248) Cl-H stretch
216	187.92	-------	1.56	2.85	(8.40%) (153) ring B-prop: 2-BEND
					(6.21%) (186) ring C-prop: 1-BEND
					(6.03%) (227) ring A-ethyl: 2-TORS
217	184.23	-------	2.73	1.99	(8.70%) (102) ring D torsion
					(7.40%) (205) ring D-methyl: WAGG
					(6.48%) (207) ring D-ethyl: WAGG
					(8.33%) (215) ring D-ethyl: BEND
					(6.34%) (237) bridge C-D: TORS
					(18.17%) (248) Cl-H stretch
218	179.03	-------	3.66	0.27	(11.77%) (91) ring A torsion
					(5.49%) (123) ring A-ethyl: BEND
					(5.32%) (247) Cl-H stretch
219	168.20	-------	3.91	0.30	(8.48%) (91) ring A torsion
					(6.26%) (95) ring B torsion
					(5.05%) (109) ring A-methyl: ROCK
					(6.96%) (162) bridge BC: BEND
					(13.67%) (235) bridge A-B: TORS
					(17.40%) (248) Cl-H stretch
220	166.52	-------	6.04	1.53	(7.14%) (91) ring A torsion
					(5.21%) (102) ring D torsion
					(5.09%) (205) ring D-methyl: WAGG
					(7.20%) (207) ring D-ethyl: WAGG
					(7.62%) (215) ring D-ethyl: BEND
					(29.54%) (248) Cl-H stretch
221	164.36	-------	0.09	1.37	(6.11%) (98) ring C torsion
					(5.54%) (202) ring D: bridge CD ROCK
					(7.00%) (207) ring D-ethyl: WAGG
					(15.04%) (237) bridge C-D: TORS
					(6.55%) (238) ring C-methyl: TORS
					(7.38%) (243) bridge C=D: TORS
					(9.06%) (244) ring D-methyl: TORS
222	150.99	-------	0.91	6.45	(8.12%) (103) ring D torsion
					(8.95%) (153) ring B-prop: 2-BEND
					(5.42%) (237) bridge C-D: TORS
					(13.52%) (247) Cl-H stretch
223	146.68	-------	5.15	0.41	(12.95%) (91) ring A torsion
					(5.25%) (114) ring A-ethyl: WAGG
					(7.17%) (115) ring A-ethyl: TWIST
					(-6.11%) (235) bridge A-B: TORS
					(30.71%) (247) Cl-H stretch
					(8.16%) (249) Cl OUT
224	140.62	-------	0.98	1.07	(6.24%) (91) ring A torsion
					(5.07%) (102) ring D torsion
					(11.80%) (103) ring D torsion
					(5.73%) (141) ring B: bridge BC ROCK
					(21.67%) (228) ring B-methyl: TORS
225	137.49	-------	3.49	0.19	(12.94%) (103) ring D torsion
					(18.09%) (228) ring B-methyl: TORS
					(10.95%) (244) ring D-methyl: TORS
					(5.56%) (247) Cl-H stretch
226	131.59	-------	0.97	0.41	(5.20%) (91) ring A torsion
					(5.18%) (226) ring A-ethyl: 1-TORS
					(48.74%) (228) ring B-methyl: TORS
					(12.95%) (244) ring D-methyl: TORS
227	126.88	-------	0.53	0.30	(5.92%) (116) ring A: bridge AB ROCK
					(5.45%) (168) bridge AB: BEND
					(6.41%) (175) ring C-prop: 1-ROCK
					(17.09%) (226) ring A-ethyl: 1-TORS
					(6.27%) (247) Cl-H stretch
228	121.84	-------	0.81	4.48	(7.74%) (174) ring C: bridge BC WAGG
					(36.15%) (244) ring D-methyl: TORS
229	104.34	-------	1.02	1.31	(5.44%) (102) ring D torsion
					(6.73%) (173) ring C: bridge BC ROCK
					(30.64%) (226) ring A-ethyl: 1-TORS
230	101.52	-------	0.28	5.98	(5.48%) (98) ring C torsion
					(22.22%) (102) ring D torsion
					(9.72%) (207) ring D-ethyl: WAGG
					(14.45%) (226) ring A-ethyl: 1-TORS
					(6.11%) (237) bridge C-D: TORS
231	90.40	-------	0.89	0.66	(5.03%) (91) ring A torsion
					(16.96%) (94) ring B torsion
					(6.30%) (117) ring A: bridge AB WAGG
					(9.26%) (140) ring B-prop: 1-WAGG
					(6.74%) (148) ring B-prop: 1-BEND
					(7.16%) (229) ring B-prop: 1-TORS
					(5.73%) (233) bridge BC: 1-TORS

					(5.24%) (247) Cl-H stretch
232	80.07	-------	1.05	1.01	(5.16%) (103) ring D torsion
					(13.27%) (116) ring A: bridge AB ROCK
					(5.66%) (135) ring B: bridge AB ROCK
					(15.00%) (168) bridge AB: BEND
					(11.46%) (226) ring A-ethyl: 1-TORS
					(5.20%) (235) bridge A-B: TORS
					(6.63%) (239) ring C-prop: 1-TORS
					(6.57%) (240) ring C-prop: 2-TORS
					(13.34%) (249) Cl OUT
233	76.19	-------	0.42	1.03	(5.35%) (103) ring D torsion
					(6.44%) (142) ring B: bridge BC WAGG
					(6.28%) (233) bridge BC: 1-TORS
					(21.83%) (239) ring C-prop: 1-TORS
234	68.87	-------	2.77	1.30	(13.38%) (229) ring B-prop: 1-TORS
					(8.56%) (234) bridge A=B: TORS
					(9.19%) (236) bridge BC: 2-TORS
					(6.55%) (237) bridge C-D: TORS
					(12.52%) (249) Cl OUT
235	61.28	-------	0.85	0.28	(5.66%) (102) ring D torsion
					(5.48%) (229) ring B-prop: 1-TORS
					(6.13%) (236) bridge BC: 2-TORS
					(16.68%) (240) ring C-prop: 2-TORS
					(10.27%) (249) Cl OUT
236	58.92	-------	0.02	2.76	(6.52%) (162) bridge BC: BEND
					(10.83%) (165) bridge CD: BEND
					(13.49%) (229) ring B-prop: 1-TORS
					(11.50%) (231) ring B-prop: 3-TORS
					(5.41%) (245) ring D-ethyl: 1-TORS
237	45.79	-------	4.07	0.47	(9.07%) (229) ring B-prop: 1-TORS
					(34.22%) (230) ring B-prop: 2-TORS
					(12.64%) (231) ring B-prop: 3-TORS
					(11.82%) (241) ring C-prop: 3-TORS
238	44.30	-------	0.65	0.28	(5.19%) (98) ring C torsion
					(6.50%) (180) ring C: bridge CD WAGG
					(8.90%) (230) ring B-prop: 2-TORS
					(35.35%) (231) ring B-prop: 3-TORS
					(14.19%) (241) ring C-prop: 3-TORS
239	41.32	-------	0.26	1.85	(5.39%) (241) ring C-prop: 3-TORS
					(5.50%) (243) bridge C=D: TORS
					(56.94%) (245) ring D-ethyl: 1-TORS
240	36.09	-------	0.89	0.72	(40.70%) (90) ring A torsion
					(7.24%) (234) bridge A=B: TORS
					(11.40%) (235) bridge A-B: TORS
					(5.17%) (241) ring C-prop: 3-TORS
					(10.08%) (245) ring D-ethyl: 1-TORS
241	29.84	-------	1.27	3.33	(11.96%) (90) ring A torsion
					(5.32%) (230) ring B-prop: 2-TORS
					(5.13%) (231) ring B-prop: 3-TORS
					(5.20%) (233) bridge BC: 1-TORS
					(9.88%) (237) bridge C-D: TORS
					(26.86%) (241) ring C-prop: 3-TORS
					(9.67%) (249) Cl OUT
242	28.60	-------	0.14	4.40	(7.91%) (90) ring A torsion
					(5.58%) (94) ring B torsion
					(10.15%) (136) ring B: bridge AB WAGG
					(7.44%) (140) ring B-prop: 1-WAGG
					(10.82%) (229) ring B-prop: 1-TORS
					(13.39%) (234) bridge A=B: TORS
					(7.76%) (239) ring C-prop: 1-TORS
					(5.63%) (241) ring C-prop: 3-TORS
243	26.78	-------	1.19	0.92	(6.27%) (140) ring B-prop: 1-WAGG
					(16.12%) (229) ring B-prop: 1-TORS
					(19.04%) (230) ring B-prop: 2-TORS
					(20.79%) (239) ring C-prop: 1-TORS
					(11.64%) (240) ring C-prop: 2-TORS
244	24.12	-------	0.25	1.93	(6.16%) (229) ring B-prop: 1-TORS
					(19.55%) (230) ring B-prop: 2-TORS
					(12.82%) (231) ring B-prop: 3-TORS
					(19.96%) (240) ring C-prop: 2-TORS
					(5.11%) (243) bridge C=D: TORS
					(6.47%) (245) ring D-ethyl: 1-TORS
245	22.01	-------	1.29	6.37	(5.56%) (103) ring D torsion
					(5.17%) (167) bridge CD: C-H OUT
					(6.15%) (230) ring B-prop: 2-TORS
					(14.52%) (237) bridge C-D: TORS
					(13.01%) (239) ring C-prop: 1-TORS
					(9.49%) (241) ring C-prop: 3-TORS
					(9.66%) (249) Cl OUT

246	19.65	-------	0.11	4.60	(8.08%)	(162) bridge BC: BEND
					(11.42%)	(165) bridge CD: BEND
					(9.83%)	(179) ring C: bridge CD ROCK
					(5.84%)	(240) ring C-prop: 2-TORS
					(9.36%)	(245) ring D-ethyl: 1-TORS
247	15.36	-------	0.17	2.65	(21.20%)	(90) ring A torsion
					(26.45%)	(235) bridge A-B: TORS
					(8.54%)	(236) bridge BC: 2-TORS
248	12.04	-------	0.93	30.52	(5.95%)	(162) bridge BC: BEND
					(12.07%)	(174) ring C: bridge BC WAGG
					(8.24%)	(233) bridge BC: 1-TORS
					(28.75%)	(237) bridge C-D: TORS
					(7.76%)	(249) Cl OUT
249	10.61	-------	0.01	5.47	(5.26%)	(90) ring A torsion
					(5.05%)	(136) ring B: bridge AB WAGG
					(15.05%)	(233) bridge BC: 1-TORS
					(5.26%)	(235) bridge A-B: TORS
					(23.35%)	(236) bridge BC: 2-TORS

ZZEssa, $^{13}C(5)$, H_2O

	calc. [cm-1]	exp. [cm-1]	IRint [km/mol]	Rint (rel.)	contribution
1	3522.77	-------	55.00	0.15	(100.07%) (67) ring C-prop: O-H stretch (27,84)
2	3516.59	-------	55.88	0.17	(100.07%) (39) ring B-prop: O-H stretch (85,83)
3	3489.04	-------	55.55	0.13	(99.72%) (69) ring D: N-H stretch (29,28)
4	3122.34	-------	774.54	0.10	(38.07%) (1) ring A: N-H stretch (47,46)<br>(33.26%) (20) ring B: N-H stretch (66,65)<br>(28.34%) (47) ring C: N-H stretch (10, 9)
5	3067.41	-------	0.98	0.18	(99.72%) (42) bridge BC: C-H stretch (4, 3)
6	3047.68	-------	37.38	0.31	(98.82%) (46) bridge AB: C-H stretch (8, 7)
7	3037.21	-------	513.67	0.43	(58.04%) (1) ring A: N-H stretch (47,46)<br>(9.10%) (20) ring B: N-H stretch (66,65)<br>(31.86%) (47) ring C: N-H stretch (10, 9)
8	3015.78	-------	170.97	0.09	(55.12%) (20) ring B: N-H stretch (66,65)<br>(5.34%) (43) bridge CD: C-H stretch (6, 5)<br>(36.21%) (47) ring C: N-H stretch (10, 9)
9	3013.48	-------	16.24	0.08	(48.50%) (56) ring C-methyl: C-H stretch (16,15)<br>(45.35%) (57) ring C-methyl: C-H stretch (17,15)
10	3012.89	-------	9.81	0.11	(79.22%) (78) ring D-methyl: C-H stretch (35,34)<br>(5.50%) (79) ring D-methyl: C-H stretch (36,34)<br>(12.73%) (80) ring D-methyl: C-H stretch (37,34)
11	3011.72	-------	4.81	0.05	(91.32%) (43) bridge CD: C-H stretch (6, 5)
12	3006.08	-------	18.88	0.08	(9.15%) (85) ring D-ethyl: C-H stretch (42,41)<br>(11.25%) (86) ring D-ethyl: C-H stretch (43,41)<br>(75.62%) (87) ring D-ethyl: C-H stretch (44,41)
13	3003.59	-------	10.07	0.09	(16.39%) (10) ring A-methyl: C-H stretch (55,54)<br>(6.14%) (11) ring A-methyl: C-H stretch (56,54)<br>(77.33%) (12) ring A-methyl: C-H stretch (57,54)
14	3000.91	-------	15.43	0.03	(6.78%) (60) ring C-prop: C-H stretch (20,19)<br>(71.63%) (61) ring C-prop: C-H stretch (21,19)<br>(19.66%) (63) ring C-prop: C-H stretch (23,22)
15	2998.99	-------	34.92	0.12	(13.32%) (27) ring B-methyl: C-H stretch (72,71)<br>(7.80%) (31) ring B-prop: C-H stretch (76,75)<br>(9.98%) (32) ring B-prop: C-H stretch (77,75)<br>(60.06%) (34) ring B-prop: C-H stretch (79,78)<br>(7.03%) (35) ring B-prop: C-H stretch (80,78)
16	2996.58	-------	11.75	0.11	(73.56%) (27) ring B-methyl: C-H stretch (72,71)<br>(5.57%) (29) ring B-methyl: C-H stretch (74,71)<br>(13.71%) (34) ring B-prop: C-H stretch (79,78)
17	2987.25	-------	27.12	0.17	(31.88%) (17) ring A-ethyl: C-H stretch (62,60)<br>(58.84%) (18) ring A-ethyl: C-H stretch (63,60)
18	2985.50	-------	50.79	0.42	(51.26%) (85) ring D-ethyl: C-H stretch (42,41)<br>(45.14%) (86) ring D-ethyl: C-H stretch (43,41)
19	2984.68	-------	0.97	0.06	(18.40%) (61) ring C-prop: C-H stretch (21,19)<br>(70.81%) (63) ring C-prop: C-H stretch (23,22)<br>(7.85%) (64) ring C-prop: C-H stretch (24,22)
20	2984.25	-------	46.15	0.16	(45.83%) (10) ring A-methyl: C-H stretch (55,54)<br>(43.16%) (11) ring A-methyl: C-H stretch (56,54)<br>(5.36%) (18) ring A-ethyl: C-H stretch (63,60)
21	2981.34	-------	8.49	0.06	(8.45%) (56) ring C-methyl: C-H stretch (16,15)<br>(10.06%) (57) ring C-methyl: C-H stretch (17,15)<br>(8.87%) (58) ring C-methyl: C-H stretch (18,15)<br>(36.10%) (79) ring D-methyl: C-H stretch (36,34)<br>(35.98%) (80) ring D-methyl: C-H stretch (37,34)
22	2979.96	-------	8.80	0.13	(27.75%) (56) ring C-methyl: C-H stretch (16,15)<br>(27.06%) (57) ring C-methyl: C-H stretch (17,15)<br>(17.96%) (58) ring C-methyl: C-H stretch (18,15)<br>(12.94%) (79) ring D-methyl: C-H stretch (36,34)<br>(13.89%) (80) ring D-methyl: C-H stretch (37,34)
23	2979.83	-------	34.07	0.08	(5.95%) (14) ring A-ethyl: C-H stretch (59,58)<br>(46.59%) (16) ring A-ethyl: C-H stretch (61,60)<br>(28.83%) (17) ring A-ethyl: C-H stretch (62,60)<br>(11.05%) (18) ring A-ethyl: C-H stretch (63,60)<br>(6.24%) (40) ring A-ethyl: C-H stretch (1,58)
24	2977.85	-------	4.03	0.05	(46.43%) (31) ring B-prop: C-H stretch (76,75)<br>(35.13%) (32) ring B-prop: C-H stretch (77,75)

					(16.50%) (34) ring B-prop: C-H stretch (79,78)
25	2956.12	-------	10.77	0.09	(56.79%) (82) ring D-ethyl: C-H stretch (39,38)
					(36.28%) (83) ring D-ethyl: C-H stretch (40,38)
26	2954.93	-------	14.59	0.16	(48.16%) (28) ring B-methyl: C-H stretch (73,71)
					(51.99%) (29) ring B-methyl: C-H stretch (74,71)
27	2949.87	-------	6.13	0.09	(52.30%) (14) ring A-ethyl: C-H stretch (59,58)
					(9.42%) (17) ring A-ethyl: C-H stretch (62,60)
					(28.62%) (40) ring A-ethyl: C-H stretch (1,58)
28	2947.12	-------	19.86	0.19	(43.73%) (31) ring B-prop: C-H stretch (76,75)
					(52.09%) (32) ring B-prop: C-H stretch (77,75)
29	2941.13	-------	8.02	0.20	(93.98%) (4) ring A: C-H stretch (50,49)
30	2936.43	-------	17.54	0.30	(75.58%) (60) ring C-prop: C-H stretch (20,19)
					(8.49%) (61) ring C-prop: C-H stretch (21,19)
					(13.79%) (64) ring C-prop: C-H stretch (24,22)
31	2930.97	-------	24.68	0.45	(17.31%) (78) ring D-methyl: C-H stretch (35,34)
					(43.16%) (79) ring D-methyl: C-H stretch (36,34)
					(36.55%) (80) ring D-methyl: C-H stretch (37,34)
32	2928.56	-------	2.64	0.14	(8.79%) (34) ring B-prop: C-H stretch (79,78)
					(87.42%) (35) ring B-prop: C-H stretch (80,78)
33	2925.03	-------	11.13	0.09	(14.88%) (60) ring C-prop: C-H stretch (20,19)
					(7.17%) (63) ring C-prop: C-H stretch (23,22)
					(76.99%) (64) ring C-prop: C-H stretch (24,22)
34	2922.01	-------	46.01	0.26	(38.42%) (85) ring D-ethyl: C-H stretch (42,41)
					(38.29%) (86) ring D-ethyl: C-H stretch (43,41)
					(19.07%) (87) ring D-ethyl: C-H stretch (44,41)
35	2921.78	-------	72.95	0.36	(29.53%) (6) ring A: C-H stretch (52,51)
					(15.85%) (10) ring A-methyl: C-H stretch (55,54)
					(23.80%) (11) ring A-methyl: C-H stretch (56,54)
					(10.89%) (12) ring A-methyl: C-H stretch (57,54)
					(9.76%) (14) ring A-ethyl: C-H stretch (59,58)
36	2919.15	-------	2.49	0.04	(16.34%) (6) ring A: C-H stretch (52,51)
					(15.21%) (10) ring A-methyl: C-H stretch (55,54)
					(21.31%) (11) ring A-methyl: C-H stretch (56,54)
					(8.74%) (12) ring A-methyl: C-H stretch (57,54)
					(15.12%) (14) ring A-ethyl: C-H stretch (59,58)
					(14.43%) (40) ring A-ethyl: C-H stretch (1,58)
37	2918.33	-------	21.76	0.23	(37.09%) (82) ring D-ethyl: C-H stretch (39,38)
					(57.06%) (83) ring D-ethyl: C-H stretch (40,38)
38	2917.20	-------	41.65	0.28	(11.68%) (56) ring C-methyl: C-H stretch (16,15)
					(13.90%) (57) ring C-methyl: C-H stretch (17,15)
					(70.90%) (58) ring C-methyl: C-H stretch (18,15)
39	2915.75	-------	27.22	0.10	(9.85%) (6) ring A: C-H stretch (52,51)
					(41.08%) (16) ring A-ethyl: C-H stretch (61,60)
					(26.18%) (17) ring A-ethyl: C-H stretch (62,60)
					(18.90%) (18) ring A-ethyl: C-H stretch (63,60)
40	2911.28	-------	6.26	0.20	(39.41%) (6) ring A: C-H stretch (52,51)
					(11.35%) (14) ring A-ethyl: C-H stretch (59,58)
					(44.71%) (40) ring A-ethyl: C-H stretch (1,58)
41	2909.71	-------	45.38	0.46	(12.35%) (27) ring B-methyl: C-H stretch (72,71)
					(45.86%) (28) ring B-methyl: C-H stretch (73,71)
					(41.10%) (29) ring B-methyl: C-H stretch (74,71)
42	1783.57	-------	303.72	1.20	(84.42%) (19) ring A: C=O stretch (64,48)
					(5.12%) (88) ring A bending
43	1774.15	-------	191.11	0.04	(81.29%) (66) ring C-prop: C=O stretch (26,25)
					(6.41%) (199) ring C-prop: C-O-H BEND
44	1760.70	-------	252.98	0.02	(81.10%) (37) ring B-prop: C=O stretch (82,81)
					(6.53%) (161) ring B-prop: C-O-H BEND
45	1742.89	-------	919.65	3.45	(79.64%) (74) ring D: C=O stretch (33,45)
46	1621.75	-------	158.22	100.00	(7.32%) (52) bridge CD: C-C stretch (14, 5)
					(58.23%) (70) bridge CD: C=C stretch (30, 5)
					(6.07%) (71) ring D: C-N stretch (30,28)
					(10.04%) (166) bridge CD: C-H ROCK
47	1613.74	-------	22.55	7.77	(63.99%) (73) ring D: C=C stretch (32,31)
					(5.69%) (77) ring D-methyl: C-C stretch (34,31)
					(7.58%) (81) ring D-ethyl: C-C stretch (38,32)
48	1611.39	-------	681.38	15.26	(22.52%) (41) bridge BC: C-.C stretch (3,70)
					(9.40%) (44) bridge AB: C=C stretch (7,53)
					(17.91%) (48) bridge BC: C-.C stretch (11, 3)
					(5.79%) (73) ring D: C=C stretch (32,31)

					(12.71%)	(163)	bridge BC: C-H ROCK
49	1583.86	-------	716.46	5.73	(7.43%)	(7)	ring A: C-N stretch (53,46)
					(24.88%)	(23)	ring B: C-.C stretch (69,68)
					(24.30%)	(44)	bridge AB: C=C stretch (7,53)
					(5.93%)	(104)	ring A: N-H ROCK
50	1555.68	-------	242.14	21.16	(5.82%)	(21)	ring B: C-N stretch (67,65)
					(5.33%)	(45)	bridge AB: C-C stretch (7,67)
					(5.16%)	(48)	bridge BC: C-.C stretch (11, 3)
					(29.27%)	(133)	ring B: N-H ROCK
					(25.47%)	(171)	ring C: N-H ROCK
51	1538.04	-------	1401.63	6.91	(25.33%)	(23)	ring B: C-.C stretch (69,68)
					(5.30%)	(26)	ring B-methyl: C-C stretch (71,68)
					(5.75%)	(41)	bridge BC: C-.C stretch (3,70)
					(14.06%)	(44)	bridge AB: C=C stretch (7,53)
					(6.79%)	(104)	ring A: N-H ROCK
52	1518.78	-------	126.45	8.70	(8.39%)	(45)	bridge AB: C-C stretch (7,67)
					(6.80%)	(54)	ring C: C-C stretch (14,13)
					(11.95%)	(133)	ring B: N-H ROCK
					(17.15%)	(171)	ring C: N-H ROCK
53	1506.42	-------	257.85	4.72	(35.15%)	(51)	ring C: C-.C stretch (13,12)
					(11.54%)	(53)	ring C: C-N stretch (14, 9)
					(7.55%)	(55)	ring C-methyl: C-C stretch (15,13)
54	1481.43	-------	12.43	0.14	(64.55%)	(182)	ring C-methyl: ADEFa
					(5.71%)	(184)	ring C-methyl: ROCKa
					(5.57%)	(211)	ring D-methyl: ADEFa
55	1477.19	-------	5.61	0.14	(5.99%)	(216)	ring D-ethyl: CH2 SCIS
					(72.89%)	(221)	ring D-ethyl: CH3 ADEFa
					(10.39%)	(222)	ring D-ethyl: CH3 ADEFb
					(6.74%)	(223)	ring D-ethyl: CH3 ROCKa
56	1476.95	-------	71.66	0.24	(10.64%)	(145)	ring B-methyl: ADEFb
					(72.14%)	(149)	ring B-prop: 1-CH2-SCIS
57	1473.57	-------	4.97	0.05	(14.26%)	(124)	ring A-ethyl: CH2 SCIS
					(44.32%)	(129)	ring A-ethyl: CH3 ADEFa
					(25.76%)	(130)	ring A-ethyl: CH3 ADEFb
58	1468.69	-------	1.08	0.20	(16.61%)	(119)	ring A-methyl: ADEFa
					(36.37%)	(129)	ring A-ethyl: CH3 ADEFa
					(24.09%)	(130)	ring A-ethyl: CH3 ADEFb
59	1468.29	-------	21.56	3.54	(8.63%)	(7)	ring A: C-N stretch (53,46)
					(6.06%)	(119)	ring A-methyl: ADEFa
					(5.36%)	(133)	ring B: N-H ROCK
					(5.78%)	(144)	ring B-methyl: ADEFa
					(26.18%)	(169)	bridge AB: C-H ROCK
60	1467.18	-------	8.76	0.30	(29.09%)	(119)	ring A-methyl: ADEFa
					(31.10%)	(120)	ring A-methyl: ADEFb
					(20.87%)	(130)	ring A-ethyl: CH3 ADEFb
61	1465.24	-------	2.98	0.05	(37.50%)	(183)	ring C-methyl: ADEFb
					(12.49%)	(187)	ring C-prop: 1-CH2-SCIS
					(29.89%)	(212)	ring D-methyl: ADEFb
62	1465.11	-------	3.31	0.14	(34.16%)	(119)	ring A-methyl: ADEFa
					(49.25%)	(120)	ring A-methyl: ADEFb
63	1463.92	-------	11.47	0.20	(6.60%)	(221)	ring D-ethyl: CH3 ADEFa
					(77.47%)	(222)	ring D-ethyl: CH3 ADEFb
					(7.14%)	(224)	ring D-ethyl: CH3 ROCKb
64	1459.03	-------	58.73	0.28	(16.10%)	(144)	ring B-methyl: ADEFa
					(26.72%)	(145)	ring B-methyl: ADEFb
					(7.12%)	(149)	ring B-prop: 1-CH2-SCIS
					(11.23%)	(187)	ring C-prop: 1-CH2-SCIS
					(6.34%)	(212)	ring D-methyl: ADEFb
65	1457.20	-------	11.22	0.46	(13.16%)	(144)	ring B-methyl: ADEFa
					(5.32%)	(145)	ring B-methyl: ADEFb
					(5.07%)	(183)	ring C-methyl: ADEFb
					(22.79%)	(187)	ring C-prop: 1-CH2-SCIS
					(28.43%)	(212)	ring D-methyl: ADEFb
66	1454.93	-------	54.74	0.93	(67.32%)	(211)	ring D-methyl: ADEFa
					(6.40%)	(213)	ring D-methyl: ROCKa
67	1454.19	-------	13.36	0.17	(69.09%)	(124)	ring A-ethyl: CH2 SCIS
					(13.57%)	(130)	ring A-ethyl: CH3 ADEFb
68	1452.98	-------	13.60	0.76	(43.67%)	(144)	ring B-methyl: ADEFa
					(16.33%)	(145)	ring B-methyl: ADEFb
					(13.93%)	(183)	ring C-methyl: ADEFb
					(5.02%)	(212)	ring D-methyl: ADEFb
69	1452.79	-------	16.96	0.29	(7.32%)	(144)	ring B-methyl: ADEFa

```
                                              ( 19.99%) (145) ring B-methyl: ADEFb
                                              ( 22.26%) (183) ring C-methyl: ADEFb
                                              ( 26.93%) (187) ring C-prop: 1-CH2-SCIS

 70   1445.34   -------     4.08      0.51    ( 65.64%) (216) ring D-ethyl: CH2 SCIS
                                              (  8.42%) (221) ring D-ethyl: CH3 ADEFa

 71   1442.46   -------    12.38      3.49    (  6.74%) ( 21) ring B: C-N stretch (67,65)
                                              (  9.36%) (169) bridge AB: C-H ROCK
                                              ( 14.02%) (216) ring D-ethyl: CH2 SCIS

 72   1438.92   -------    18.14      1.46    ( 68.14%) (154) ring B-prop: 2-CH2-SCIS
                                              (  8.36%) (163) bridge BC: C-H ROCK

 73   1434.72   -------    13.47      3.32    (  5.97%) ( 50) ring C: C-C stretch (12,11)
                                              (  5.89%) (149) ring B-prop: 1-CH2-SCIS
                                              ( 23.51%) (154) ring B-prop: 2-CH2-SCIS
                                              ( 15.47%) (163) bridge BC: C-H ROCK
                                              (  5.89%) (187) ring C-prop: 1-CH2-SCIS

 74   1424.41   -------    58.59      0.44    ( 78.35%) (192) ring C-prop: 2-CH2-SCIS

 75   1414.90   -------    64.43      2.57    (  6.16%) ( 50) ring C: C-C stretch (12,11)
                                              (  5.42%) ( 54) ring C: C-C stretch (14,13)
                                              (  6.72%) ( 59) ring C-prop: C-C stretch (19,12)
                                              (  7.39%) ( 97) ring C bending
                                              (  5.65%) (166) bridge CD: C-H ROCK
                                              (  6.23%) (182) ring C-methyl: ADEFa
                                              (  8.77%) (192) ring C-prop: 2-CH2-SCIS

 76   1410.00   -------   195.36     10.00    ( 14.74%) ( 25) ring B: C-C stretch (70,69)
                                              (  6.52%) ( 30) ring B-prop: C-C stretch (75,69)
                                              (  5.53%) ( 48) bridge BC: C-.C stretch (11, 3)
                                              (  6.40%) ( 93) ring B bending
                                              (  6.98%) (143) ring B-methyl: SDEF
                                              ( 14.09%) (166) bridge CD: C-H ROCK
                                              (  5.99%) (200) ring D: N-H ROCK

 77   1399.42   -------    42.23      0.61    (  8.94%) ( 65) ring C-prop: C-C stretch (25,22)
                                              (  8.98%) ( 68) ring C-prop: C-O stretch (27,25)
                                              (  5.38%) (190) ring C-prop: 1-CH2-TWIST
                                              (  8.90%) (192) ring C-prop: 2-CH2-SCIS
                                              ( 29.92%) (194) ring C-prop: 2-CH2-WAGG
                                              (  8.30%) (197) ring C-prop: C=O ROCK
                                              (  9.79%) (199) ring C-prop: C-O-H BEND

 78   1395.42   -------    69.34      0.11    (  8.97%) ( 36) ring B-prop: C-C stretch (81,78)
                                              ( 11.26%) ( 38) ring B-prop: C-O stretch (83,81)
                                              ( 21.65%) (156) ring B-prop: 2-CH2-WAGG
                                              (  9.74%) (159) ring B-prop: C=O ROCK
                                              ( 13.89%) (161) ring B-prop: C-O-H BEND

 79   1394.11   -------    45.22      0.81    (  5.79%) ( 50) ring C: C-C stretch (12,11)
                                              ( 14.54%) (143) ring B-methyl: SDEF
                                              (  5.26%) (181) ring C-methyl: SDEF

 80   1389.38   -------     9.40      0.23    (  9.76%) (181) ring C-methyl: SDEF
                                              ( 67.84%) (210) ring D-methyl: SDEF

 81   1384.88   -------     4.71      0.55    (  6.11%) (143) ring B-methyl: SDEF
                                              ( 69.88%) (181) ring C-methyl: SDEF
                                              (  6.45%) (210) ring D-methyl: SDEF

 82   1384.16   -------     1.77      0.04    ( 88.67%) (128) ring A-ethyl: CH3 SDEF

 83   1380.11   -------    30.43      1.72    (  6.96%) (104) ring A: N-H ROCK
                                              ( 12.84%) (118) ring A-methyl: SDEF
                                              ( 46.88%) (143) ring B-methyl: SDEF

 84   1376.39   -------     6.02      1.22    (  6.47%) (104) ring A: N-H ROCK
                                              ( 45.23%) (118) ring A-methyl: SDEF
                                              ( 14.54%) (143) ring B-methyl: SDEF

 85   1372.66   -------    28.87      1.78    (  7.15%) (104) ring A: N-H ROCK
                                              ( 33.11%) (118) ring A-methyl: SDEF
                                              (  8.43%) (220) ring D-ethyl: CH3 SDEF

 86   1369.97   -------     2.38      0.16    ( 77.56%) (220) ring D-ethyl: CH3 SDEF

 87   1365.00   -------    40.96      4.28    ( 10.45%) ( 44) bridge AB: C=C stretch ( 7,53)
                                              ( 39.71%) (104) ring A: N-H ROCK

 88   1352.48   -------     7.10      0.56    ( 17.42%) (112) ring A-ethyl: SCIS
                                              ( 57.01%) (126) ring A-ethyl: CH2 WAGG

 89   1351.04   -------    26.55      9.02    (  7.29%) ( 71) ring D: C-N stretch (30,28)
                                              (  7.04%) ( 72) ring D: C-C stretch (31,30)
                                              (  6.21%) ( 81) ring D-ethyl: C-C stretch (38,32)
                                              (  9.83%) (101) ring D bending
                                              ( 11.11%) (200) ring D: N-H ROCK
                                              (  6.50%) (210) ring D-methyl: SDEF
                                              ( 14.61%) (218) ring D-ethyl: CH2 WAGG
                                              (  7.04%) (220) ring D-ethyl: CH3 SDEF
```

90	1340.46	-------	15.50	0.47	(9.69%) (108) ring A-methyl: SCIS
					(6.57%) (112) ring A-ethyl: SCIS
					(6.52%) (126) ring A-ethyl: CH2 WAGG
					(6.49%) (127) ring A-ethyl: CH2 TWIST
					(9.46%) (151) ring B-prop: 1-CH2-WAGG
					(19.71%) (189) ring C-prop: 1-CH2-WAGG
91	1340.32	-------	26.92	1.82	(6.46%) (108) ring A-methyl: SCIS
					(5.04%) (126) ring A-ethyl: CH2 WAGG
					(41.54%) (189) ring C-prop: 1-CH2-WAGG
					(5.99%) (195) ring C-prop: 2-CH2-TWIST
92	1337.32	-------	37.80	2.87	(10.27%) (71) ring D: C-N stretch (30,28)
					(5.78%) (76) ring D: C-C stretch (33,32)
					(8.99%) (151) ring B-prop: 1-CH2-WAGG
					(11.47%) (200) ring D: N-H ROCK
					(16.54%) (218) ring D-ethyl: CH2 WAGG
93	1336.98	-------	23.74	0.27	(36.53%) (151) ring B-prop: 1-CH2-WAGG
					(8.07%) (156) ring B-prop: 2-CH2-WAGG
					(7.63%) (161) ring B-prop: C-O-H BEND
					(6.53%) (189) ring C-prop: 1-CH2-WAGG
94	1330.89	-------	9.10	24.58	(9.74%) (22) ring B: C-C stretch (68,67)
					(5.52%) (24) ring B: C-N stretch (70,65)
					(7.84%) (41) bridge BC: C-.C stretch (3,70)
					(11.95%) (45) bridge AB: C-C stretch (7,67)
					(28.23%) (133) ring B: N-H ROCK
95	1319.19	-------	9.74	1.03	(9.81%) (76) ring D: C-C stretch (33,32)
					(6.59%) (101) ring D bending
					(44.10%) (218) ring D-ethyl: CH2 WAGG
					(9.38%) (219) ring D-ethyl: CH2 TWIST
96	1315.05	-------	46.19	2.33	(11.37%) (190) ring C-prop: 1-CH2-TWIST
					(21.31%) (194) ring C-prop: 2-CH2-WAGG
					(5.23%) (197) ring C-prop: C=O ROCK
					(34.92%) (199) ring C-prop: C-O-H BEND
97	1307.60	-------	30.34	0.92	(30.81%) (108) ring A-methyl: SCIS
					(5.53%) (109) ring A-methyl: ROCK
					(5.72%) (112) ring A-ethyl: SCIS
					(30.52%) (127) ring A-ethyl: CH2 TWIST
98	1302.37	-------	242.99	19.84	(6.22%) (25) ring B: C-C stretch (70,69)
					(8.71%) (152) ring B-prop: 1-CH2-TWIST
					(7.24%) (171) ring C: N-H ROCK
					(5.07%) (194) ring C-prop: 2-CH2-WAGG
					(5.82%) (199) ring C-prop: C-O-H BEND
99	1290.36	-------	47.52	0.28	(25.04%) (110) ring A-methyl: WAGG
					(8.35%) (111) ring A-methyl: TWIST
					(11.13%) (114) ring A-ethyl: WAGG
					(5.85%) (115) ring A-ethyl: TWIST
					(5.31%) (126) ring A-ethyl: CH2 WAGG
100	1288.43	-------	19.16	2.11	(9.79%) (151) ring B-prop: 1-CH2-WAGG
					(10.91%) (152) ring B-prop: 1-CH2-TWIST
					(14.83%) (156) ring B-prop: 2-CH2-WAGG
					(11.25%) (157) ring B-prop: 2-CH2-TWIST
					(20.47%) (161) ring B-prop: C-O-H BEND
101	1280.92	-------	188.14	7.03	(24.29%) (53) ring C: C-N stretch (14, 9)
					(15.51%) (171) ring C: N-H ROCK
					(10.06%) (200) ring D: N-H ROCK
102	1273.83	-------	6.65	2.27	(58.83%) (219) ring D-ethyl: CH2 TWIST
					(16.19%) (224) ring D-ethyl: CH3 ROCKb
103	1256.22	-------	50.85	0.61	(6.02%) (108) ring A-methyl: SCIS
					(15.93%) (112) ring A-ethyl: SCIS
					(6.52%) (125) ring A-ethyl: CH2 ROCK
					(21.30%) (127) ring A-ethyl: CH2 TWIST
					(12.03%) (131) ring A-ethyl: CH3 ROCKa
104	1255.31	-------	13.45	0.20	(13.19%) (151) ring B-prop: 1-CH2-WAGG
					(21.88%) (152) ring B-prop: 1-CH2-TWIST
					(20.64%) (156) ring B-prop: 2-CH2-WAGG
					(10.92%) (157) ring B-prop: 2-CH2-TWIST
105	1242.89	-------	34.97	0.24	(13.14%) (166) bridge CD: C-H ROCK
					(33.09%) (190) ring C-prop: 1-CH2-TWIST
					(7.33%) (194) ring C-prop: 2-CH2-WAGG
					(6.27%) (195) ring C-prop: 2-CH2-TWIST
106	1239.04	-------	418.49	0.52	(8.20%) (2) ring A: C-N stretch (48,46)
					(5.41%) (106) ring A: C=O ROCK
					(10.52%) (166) bridge CD: C-H ROCK
					(8.66%) (169) bridge AB: C-H ROCK
					(6.58%) (190) ring C-prop: 1-CH2-TWIST
107	1238.41	-------	22.13	0.15	(16.69%) (110) ring A-methyl: WAGG

					(7.74%)	(111)	ring A-methyl: TWIST
					(7.62%)	(112)	ring A-ethyl: SCIS
					(20.07%)	(114)	ring A-ethyl: WAGG
					(11.09%)	(115)	ring A-ethyl: TWIST
108	1224.55	-------	563.98	1.36	(6.99%)	(53)	ring C: C-N stretch (14, 9)
					(5.17%)	(157)	ring B-prop: 2-CH2-TWIST
					(10.02%)	(166)	bridge CD: C-H ROCK
					(19.27%)	(195)	ring C-prop: 2-CH2-TWIST
109	1204.11	-------	65.37	0.27	(7.50%)	(152)	ring B-prop: 1-CH2-TWIST
					(9.94%)	(157)	ring B-prop: 2-CH2-TWIST
					(9.87%)	(190)	ring C-prop: 1-CH2-TWIST
					(34.80%)	(195)	ring C-prop: 2-CH2-TWIST
110	1199.40	-------	339.53	0.93	(5.24%)	(38)	ring B-prop: C-O stretch (83,81)
					(14.02%)	(152)	ring B-prop: 1-CH2-TWIST
					(17.32%)	(157)	ring B-prop: 2-CH2-TWIST
					(6.13%)	(161)	ring B-prop: C-O-H BEND
					(7.77%)	(195)	ring C-prop: 2-CH2-TWIST
111	1176.32	-------	341.34	0.06	(17.15%)	(2)	ring A: C-N stretch (48,46)
					(5.46%)	(3)	ring A: C-C stretch (49,48)
					(15.76%)	(7)	ring A: C-N stretch (53,46)
					(8.12%)	(8)	ring A: C-C stretch (53,51)
					(5.02%)	(21)	ring B: C-N stretch (67,65)
					(9.28%)	(169)	bridge AB: C-H ROCK
112	1168.46	-------	95.64	3.00	(14.02%)	(49)	ring C: C-N stretch (11, 9)
					(7.43%)	(50)	ring C: C-C stretch (12,11)
					(8.32%)	(52)	bridge CD: C-C stretch (14, 5)
					(11.88%)	(53)	ring C: C-N stretch (14, 9)
					(13.39%)	(55)	ring C-methyl: C-C stretch (15,13)
					(7.64%)	(97)	ring C bending
113	1152.52	-------	185.89	1.73	(5.31%)	(26)	ring B-methyl: C-C stretch (71,68)
					(24.58%)	(38)	ring B-prop: C-O stretch (83,81)
					(5.47%)	(68)	ring C-prop: C-O stretch (27,25)
					(7.53%)	(157)	ring B-prop: 2-CH2-TWIST
					(11.12%)	(161)	ring B-prop: C-O-H BEND
114	1148.09	-------	254.73	0.05	(5.62%)	(38)	ring B-prop: C-O stretch (83,81)
					(24.10%)	(68)	ring C-prop: C-O stretch (27,25)
					(6.12%)	(194)	ring C-prop: 2-CH2-WAGG
					(11.23%)	(199)	ring C-prop: C-O-H BEND
115	1147.36	-------	50.74	0.04	(6.97%)	(5)	ring A: C-C stretch (51,49)
					(5.92%)	(109)	ring A-methyl: ROCK
					(7.93%)	(113)	ring A-ethyl: ROCK
					(10.28%)	(121)	ring A-methyl: ROCKa
					(6.91%)	(122)	ring A-methyl: ROCKb
					(16.25%)	(125)	ring A-ethyl: CH2 ROCK
					(7.83%)	(131)	ring A-ethyl: CH3 ROCKa
116	1145.25	-------	121.20	0.16	(6.00%)	(68)	ring C-prop: C-O stretch (27,25)
					(10.75%)	(77)	ring D-methyl: C-C stretch (34,31)
					(5.33%)	(81)	ring D-ethyl: C-C stretch (38,32)
					(14.81%)	(217)	ring D-ethyl: CH2 ROCK
					(11.98%)	(224)	ring D-ethyl: CH3 ROCKb
117	1128.11	-------	109.42	0.96	(6.48%)	(24)	ring B: C-N stretch (70,65)
					(8.94%)	(59)	ring C-prop: C-C stretch (19,12)
					(13.03%)	(184)	ring C-methyl: ROCKa
118	1122.48	-------	67.40	1.19	(11.20%)	(21)	ring B: C-N stretch (67,65)
					(12.33%)	(24)	ring B: C-N stretch (70,65)
					(6.86%)	(25)	ring B: C-C stretch (70,69)
					(8.72%)	(26)	ring B-methyl: C-C stretch (71,68)
					(6.30%)	(45)	bridge AB: C-C stretch (7,67)
					(5.27%)	(49)	ring C: C-N stretch (11, 9)
119	1119.20	-------	87.93	1.37	(7.35%)	(81)	ring D-ethyl: C-C stretch (38,32)
					(5.41%)	(184)	ring C-methyl: ROCKa
					(13.76%)	(213)	ring D-methyl: ROCKa
					(10.89%)	(214)	ring D-methyl: ROCKb
					(6.24%)	(217)	ring D-ethyl: CH2 ROCK
					(5.89%)	(224)	ring D-ethyl: CH3 ROCKb
120	1112.34	-------	161.84	1.03	(8.67%)	(24)	ring B: C-N stretch (70,65)
					(12.88%)	(30)	ring B-prop: C-C stretch (75,69)
					(6.17%)	(92)	ring B bending
					(15.33%)	(147)	ring B-methyl: ROCKb
					(5.04%)	(184)	ring C-methyl: ROCKa
121	1100.68	-------	144.54	4.96	(25.23%)	(71)	ring D: C-N stretch (30,28)
					(22.70%)	(75)	ring D: C-N stretch (33,28)
					(9.12%)	(77)	ring D-methyl: C-C stretch (34,31)
					(7.06%)	(81)	ring D-ethyl: C-C stretch (38,32)
					(7.87%)	(200)	ring D: N-H ROCK
122	1094.39	-------	87.27	0.68	(10.98%)	(5)	ring A: C-C stretch (51,49)
					(6.39%)	(9)	ring A-methyl: C-C stretch (54,49)
					(13.58%)	(13)	ring A-ethyl: C-C stretch (58,51)

					(6.08%)	(115)	ring A-ethyl: TWIST
					(7.04%)	(123)	ring A-ethyl: BEND
					(7.29%)	(131)	ring A-ethyl: CH3 ROCKa
					(15.65%)	(132)	ring A-ethyl: CH3 ROCKb
123	1082.95	-------	23.40	0.04	(11.14%)	(9)	ring A-methyl: C-C stretch (54,49)
					(8.69%)	(111)	ring A-methyl: TWIST
					(5.15%)	(121)	ring A-methyl: ROCKa
					(22.57%)	(122)	ring A-methyl: ROCKb
					(14.39%)	(132)	ring A-ethyl: CH3 ROCKb
124	1072.50	-------	56.61	1.09	(5.32%)	(147)	ring B-methyl: ROCKb
					(6.24%)	(150)	ring B-prop: 1-CH2-ROCK
					(8.95%)	(184)	ring C-methyl: ROCKa
					(7.99%)	(188)	ring C-prop: 1-CH2-ROCK
125	1061.84	-------	141.04	1.78	(14.74%)	(84)	ring D-ethyl: C-C stretch (41,38)
					(12.83%)	(213)	ring D-methyl: ROCKa
					(8.45%)	(215)	ring D-ethyl: BEND
					(40.12%)	(223)	ring D-ethyl: CH3 ROCKa
126	1053.01	-------	12.51	0.78	(6.93%)	(178)	ring C-methyl: WAGG
					(7.17%)	(183)	ring C-methyl: ADEFb
					(75.70%)	(185)	ring C-methyl: ROCKb
127	1047.57	-------	31.28	0.49	(14.71%)	(146)	ring B-methyl: ROCKa
					(11.79%)	(150)	ring B-prop: 1-CH2-ROCK
					(8.63%)	(155)	ring B-prop: 2-CH2-ROCK
					(7.62%)	(188)	ring C-prop: 1-CH2-ROCK
128	1046.68	-------	3.13	0.08	(8.21%)	(138)	ring B-methyl: WAGG
					(50.67%)	(146)	ring B-methyl: ROCKa
					(24.08%)	(147)	ring B-methyl: ROCKb
129	1040.10	-------	1.44	0.30	(7.27%)	(205)	ring D-methyl: WAGG
					(21.68%)	(213)	ring D-methyl: ROCKa
					(51.21%)	(214)	ring D-methyl: ROCKb
					(7.47%)	(223)	ring D-ethyl: CH3 ROCKa
130	1026.99	-------	18.06	0.05	(6.44%)	(5)	ring A: C-C stretch (51,49)
					(6.21%)	(13)	ring A-ethyl: C-C stretch (58,51)
					(11.10%)	(15)	ring A-ethyl: C-C stretch (60,58)
					(9.69%)	(109)	ring A-methyl: ROCK
					(28.52%)	(121)	ring A-methyl: ROCKa
					(5.21%)	(125)	ring A-ethyl: CH2 ROCK
					(7.17%)	(127)	ring A-ethyl: CH2 TWIST
					(6.53%)	(132)	ring A-ethyl: CH3 ROCKb
131	1025.03	-------	1.64	0.06	(10.95%)	(9)	ring A-methyl: C-C stretch (54,49)
					(6.99%)	(13)	ring A-ethyl: C-C stretch (58,51)
					(16.84%)	(15)	ring A-ethyl: C-C stretch (60,58)
					(6.67%)	(112)	ring A-ethyl: SCIS
					(17.03%)	(122)	ring A-methyl: ROCKb
					(10.15%)	(131)	ring A-ethyl: CH3 ROCKa
132	1020.36	-------	196.18	0.26	(10.77%)	(2)	ring A: C-N stretch (48,46)
					(5.38%)	(7)	ring A: C-N stretch (53,46)
					(25.33%)	(9)	ring A-methyl: C-C stretch (54,49)
					(15.76%)	(13)	ring A-ethyl: C-C stretch (58,51)
					(16.10%)	(15)	ring A-ethyl: C-C stretch (60,58)
133	1001.19	-------	50.39	0.80	(5.10%)	(51)	ring C: C-.C stretch (13,12)
					(44.23%)	(62)	ring C-prop: C-C stretch (22,19)
					(13.76%)	(184)	ring C-methyl: ROCKa
					(6.40%)	(193)	ring C-prop: 2-CH2-ROCK
134	995.55	-------	14.69	0.11	(68.45%)	(33)	ring B-prop: C-C stretch (78,75)
					(6.04%)	(148)	ring B-prop: 1-BEND
					(5.39%)	(153)	ring B-prop: 2-BEND
					(5.14%)	(160)	ring B-prop: C=O OUT
135	989.12	-------	36.35	2.34	(8.89%)	(73)	ring D: C=C stretch (32,31)
					(10.15%)	(76)	ring D: C-C stretch (33,32)
					(7.64%)	(77)	ring D-methyl: C-C stretch (34,31)
					(10.96%)	(84)	ring D-ethyl: C-C stretch (41,38)
					(17.67%)	(213)	ring D-methyl: ROCKa
					(8.06%)	(214)	ring D-methyl: ROCKb
					(8.20%)	(219)	ring D-ethyl: CH2 TWIST
					(10.82%)	(224)	ring D-ethyl: CH3 ROCKb
136	958.15	-------	154.81	0.15	(6.85%)	(23)	ring B: C-.C stretch (69,68)
					(5.74%)	(25)	ring B: C-C stretch (70,69)
					(12.50%)	(147)	ring B-methyl: ROCKb
					(12.21%)	(150)	ring B-prop: 1-CH2-ROCK
					(13.54%)	(155)	ring B-prop: 2-CH2-ROCK
137	950.48	-------	43.33	0.19	(5.20%)	(62)	ring C-prop: C-C stretch (22,19)
					(6.67%)	(186)	ring C-prop: 1-BEND
					(5.41%)	(188)	ring C-prop: 1-CH2-ROCK
					(5.13%)	(191)	ring C-prop: 2-BEND
					(28.06%)	(193)	ring C-prop: 2-CH2-ROCK
					(14.09%)	(198)	ring C-prop: C=O OUT

138	943.84	-------	18.82	0.96	(6.44%) (5) ring A: C-C stretch (51,49)
					(7.72%) (7) ring A: C-N stretch (53,46)
					(9.99%) (8) ring A: C-C stretch (53,51)
					(28.79%) (15) ring A-ethyl: C-C stretch (60,58)
					(5.82%) (122) ring A-methyl: ROCKb
					(6.95%) (132) ring A-ethyl: CH3 ROCKb
139	935.85	-------	35.38	0.69	(8.20%) (76) ring D: C-C stretch (33,32)
					(56.43%) (84) ring D-ethyl: C-C stretch (41,38)
					(5.17%) (218) ring D-ethyl: CH2 WAGG
					(13.13%) (223) ring D-ethyl: CH3 ROCKa
140	935.23	-------	141.18	0.96	(5.35%) (49) ring C: C-N stretch (11, 9)
					(7.64%) (50) ring C: C-C stretch (12,11)
					(7.07%) (62) ring C-prop: C-C stretch (22,19)
					(12.85%) (65) ring C-prop: C-C stretch (25,22)
					(24.14%) (188) ring C-prop: 1-CH2-ROCK
					(5.91%) (193) ring C-prop: 2-CH2-ROCK
141	922.18	-------	28.61	0.14	(8.13%) (8) ring A: C-C stretch (53,51)
					(7.19%) (36) ring B-prop: C-C stretch (81,78)
					(5.96%) (147) ring B-methyl: ROCKb
					(8.48%) (155) ring B-prop: 2-CH2-ROCK
					(5.56%) (160) ring B-prop: C=O OUT
142	907.49	-------	7.81	0.11	(10.21%) (5) ring A: C-C stretch (51,49)
					(14.68%) (8) ring A: C-C stretch (53,51)
					(9.55%) (9) ring A-methyl: C-C stretch (54,49)
					(7.06%) (36) ring B-prop: C-C stretch (81,78)
					(5.23%) (121) ring A-methyl: ROCKa
					(10.09%) (132) ring A-ethyl: CH3 ROCKb
143	889.60	-------	23.76	0.14	(10.92%) (36) ring B-prop: C-C stretch (81,78)
					(7.27%) (164) bridge BC: C-H OUT
144	886.92	-------	18.45	1.92	(51.88%) (164) bridge BC: C-H OUT
					(8.79%) (233) bridge BC: 1-TORS
					(6.46%) (236) bridge BC: 2-TORS
145	879.60	-------	60.12	1.74	(10.77%) (54) ring C: C-C stretch (14,13)
					(5.14%) (72) ring D: C-C stretch (31,30)
					(11.36%) (164) bridge BC: C-H OUT
146	860.75	-------	3.86	0.13	(7.57%) (5) ring A: C-C stretch (51,49)
					(12.70%) (13) ring A-ethyl: C-C stretch (58,51)
					(8.19%) (122) ring A-methyl: ROCKb
					(20.14%) (131) ring A-ethyl: CH3 ROCKa
147	830.34	-------	103.69	0.52	(11.44%) (72) ring D: C-C stretch (31,30)
					(5.25%) (224) ring D-ethyl: CH3 ROCKb
148	823.38	-------	59.65	16.99	(15.12%) (3) ring A: C-C stretch (49,48)
					(13.85%) (105) ring A: N-H OUT
					(22.73%) (167) bridge CD: C-H OUT
149	818.30	-------	14.19	34.78	(11.20%) (3) ring A: C-C stretch (49,48)
					(7.89%) (105) ring A: N-H OUT
					(37.12%) (167) bridge CD: C-H OUT
					(6.64%) (203) ring D: bridge CD WAGG
					(7.67%) (243) bridge C=D: TORS
150	808.60	-------	7.72	0.73	(6.07%) (65) ring C-prop: C-C stretch (25,22)
					(9.35%) (165) bridge CD: BEND
					(5.51%) (217) ring D-ethyl: CH2 ROCK
					(6.70%) (224) ring D-ethyl: CH3 ROCKb
151	793.58	-------	0.85	0.28	(43.96%) (170) bridge AB: C-H OUT
					(9.23%) (234) bridge A=B: TORS
					(8.48%) (235) bridge A-B: TORS
152	791.63	-------	30.19	0.62	(10.09%) (36) ring B-prop: C-C stretch (81,78)
					(11.15%) (150) ring B-prop: 1-CH2-ROCK
					(5.25%) (155) ring B-prop: 2-CH2-ROCK
					(14.93%) (170) bridge AB: C-H OUT
153	789.55	-------	44.20	0.21	(22.64%) (65) ring C-prop: C-C stretch (25,22)
					(9.25%) (68) ring C-prop: C-O stretch (27,25)
					(6.62%) (96) ring C bending
					(8.95%) (188) ring C-prop: 1-CH2-ROCK
154	781.77	-------	85.72	0.31	(19.47%) (105) ring A: N-H OUT
					(7.07%) (125) ring A-ethyl: CH2 ROCK
					(15.55%) (134) ring B: N-H OUT
					(9.57%) (172) ring C: N-H OUT
155	773.69	-------	2.75	0.88	(10.55%) (102) ring D torsion
					(12.62%) (207) ring D-ethyl: WAGG
					(47.57%) (209) ring D: C=O OUT
156	764.91	-------	33.91	0.53	(11.75%) (105) ring A: N-H OUT
157	758.00	-------	3.88	0.32	(22.58%) (125) ring A-ethyl: CH2 ROCK
					(6.88%) (217) ring D-ethyl: CH2 ROCK

158	755.97	-------	14.62	0.15	(5.37%) (76) ring D: C-C stretch (33,32)
					(6.98%) (125) ring A-ethyl: CH2 ROCK
					(5.84%) (209) ring D: C=O OUT
					(19.56%) (217) ring D-ethyl: CH2 ROCK
					(10.09%) (224) ring D-ethyl: CH3 ROCKb
159	743.31	-------	16.45	1.15	(7.75%) (100) ring D bending
160	734.22	-------	11.94	0.17	(14.10%) (94) ring B torsion
					(10.52%) (140) ring B-prop: 1-WAGG
					(6.27%) (142) ring B: bridge BC WAGG
					(6.32%) (160) ring B-prop: C=O OUT
					(5.61%) (232) ring B-prop: 4-TORS
161	726.06	-------	30.39	8.36	(9.58%) (96) ring C bending
					(19.97%) (100) ring D bending
					(7.75%) (172) ring C: N-H OUT
162	722.10	-------	23.03	2.47	(7.04%) (92) ring B bending
					(23.31%) (105) ring A: N-H OUT
					(17.50%) (134) ring B: N-H OUT
					(12.68%) (172) ring C: N-H OUT
163	718.57	-------	29.06	0.05	(7.59%) (98) ring C torsion
					(6.37%) (174) ring C: bridge BC WAGG
					(6.58%) (176) ring C-prop: 1-WAGG
164	707.56	-------	8.66	0.30	(14.11%) (95) ring B torsion
					(12.53%) (107) ring A: C=O OUT
					(26.19%) (136) ring B: bridge AB WAGG
					(7.49%) (232) ring B-prop: 4-TORS
					(7.24%) (234) bridge A=B: TORS
165	699.74	-------	77.47	0.78	(7.16%) (155) ring B-prop: 2-CH2-ROCK
					(13.22%) (172) ring C: N-H OUT
					(20.03%) (232) ring B-prop: 4-TORS
166	695.95	-------	19.08	0.10	(9.21%) (107) ring A: C=O OUT
					(10.35%) (134) ring B: N-H OUT
					(16.59%) (172) ring C: N-H OUT
					(20.86%) (232) ring B-prop: 4-TORS
167	685.91	-------	57.49	2.27	(8.23%) (98) ring C torsion
					(8.46%) (198) ring C-prop: C=O OUT
					(31.87%) (242) ring C-prop: 4-TORS
168	680.55	-------	40.39	0.17	(6.21%) (95) ring B torsion
					(7.68%) (99) ring C torsion
					(19.70%) (242) ring C-prop: 4-TORS
169	675.98	-------	55.75	1.78	(5.65%) (92) ring B bending
					(6.20%) (95) ring B torsion
					(9.79%) (99) ring C torsion
					(18.36%) (134) ring B: N-H OUT
					(7.28%) (172) ring C: N-H OUT
170	673.07	-------	2.67	0.19	(5.51%) (81) ring D-ethyl: C-C stretch (38,32)
					(6.70%) (100) ring D bending
					(5.71%) (107) ring A: C=O OUT
					(11.13%) (134) ring B: N-H OUT
					(7.59%) (172) ring C: N-H OUT
					(5.70%) (242) ring C-prop: 4-TORS
171	668.06	-------	18.83	0.76	(10.10%) (55) ring C-methyl: C-C stretch (15,13)
172	658.83	-------	6.63	0.24	(9.10%) (88) ring A bending
					(6.43%) (99) ring C torsion
					(8.28%) (117) ring A: bridge AB WAGG
					(6.29%) (208) ring D: C=O ROCK
173	655.91	-------	13.63	7.98	(5.87%) (88) ring A bending
					(8.19%) (103) ring D torsion
					(7.16%) (117) ring A: bridge AB WAGG
					(9.50%) (203) ring D: bridge CD WAGG
					(5.87%) (205) ring D-methyl: WAGG
					(5.46%) (209) ring D: C=O OUT
174	652.80	-------	15.88	22.97	(8.10%) (102) ring D torsion
					(13.15%) (103) ring D torsion
					(6.65%) (117) ring A: bridge AB WAGG
					(20.99%) (203) ring D: bridge CD WAGG
					(7.67%) (205) ring D-methyl: WAGG
175	630.30	-------	20.95	0.43	(9.95%) (26) ring B-methyl: C-C stretch (71,68)
					(8.08%) (93) ring B bending
					(15.35%) (159) ring B-prop: C=O ROCK
					(5.36%) (232) ring B-prop: 4-TORS
176	619.94	-------	47.31	0.22	(11.90%) (159) ring B-prop: C=O ROCK
					(18.84%) (197) ring C-prop: C=O ROCK
					(7.23%) (242) ring C-prop: 4-TORS

177	610.16	-------	30.23	0.20	(6.48%) (13) ring A-ethyl: C-C stretch (58,51)
					(8.24%) (88) ring A bending
					(34.78%) (89) ring A bending
					(18.36%) (117) ring A: bridge AB WAGG
178	604.51	-------	17.51	0.89	(7.34%) (93) ring B bending
					(6.79%) (159) ring B-prop: C=O ROCK
					(24.92%) (197) ring C-prop: C=O ROCK
179	593.89	-------	17.99	0.17	(7.83%) (26) ring B-methyl: C-C stretch (71,68)
					(7.82%) (55) ring C-methyl: C-C stretch (15,13)
					(5.53%) (59) ring C-prop: C-C stretch (19,12)
					(13.38%) (93) ring B bending
					(17.53%) (97) ring C bending
180	572.94	-------	20.47	0.33	(6.08%) (77) ring D-methyl: C-C stretch (34,31)
					(8.70%) (208) ring D: C=O ROCK
181	560.38	-------	19.08	0.15	(6.47%) (142) ring B: bridge BC WAGG
					(7.87%) (148) ring B-prop: 1-BEND
					(6.48%) (158) ring B-prop: 3-BEND
					(9.68%) (159) ring B-prop: C=O ROCK
					(16.44%) (160) ring B-prop: C=O OUT
					(12.76%) (232) ring B-prop: 4-TORS
182	555.99	-------	12.42	1.01	(10.22%) (77) ring D-methyl: C-C stretch (34,31)
					(30.25%) (101) ring D bending
					(5.18%) (207) ring D-ethyl: WAGG
					(5.65%) (209) ring D: C=O OUT
					(9.72%) (215) ring D-ethyl: BEND
183	533.96	-------	12.96	2.07	(19.92%) (106) ring A: C=O ROCK
					(5.01%) (135) ring B: bridge AB ROCK
					(5.13%) (139) ring B-prop: 1-ROCK
184	520.66	-------	10.19	0.25	(5.00%) (193) ring C-prop: 2-CH2-ROCK
					(9.13%) (196) ring C-prop: 3-BEND
					(6.00%) (198) ring C-prop: C=O OUT
185	511.62	-------	52.65	2.04	(5.80%) (106) ring A: C=O ROCK
					(7.72%) (198) ring C-prop: C=O OUT
					(11.24%) (201) ring D: N-H OUT
					(5.15%) (207) ring D-ethyl: WAGG
					(5.08%) (209) ring D: C=O OUT
186	502.73	-------	1.95	3.59	(8.37%) (101) ring D bending
					(5.07%) (193) ring C-prop: 2-CH2-ROCK
					(6.42%) (198) ring C-prop: C=O OUT
					(16.67%) (201) ring D: N-H OUT
					(6.34%) (205) ring D-methyl: WAGG
					(6.01%) (207) ring D-ethyl: WAGG
					(6.78%) (209) ring D: C=O OUT
187	498.89	-------	10.94	1.57	(5.37%) (93) ring B bending
					(6.81%) (106) ring A: C=O ROCK
					(9.77%) (137) ring B-methyl: ROCK
					(5.75%) (139) ring B-prop: 1-ROCK
					(6.23%) (196) ring C-prop: 3-BEND
188	492.02	-------	7.31	0.37	(5.33%) (193) ring C-prop: 2-CH2-ROCK
					(5.07%) (196) ring C-prop: 3-BEND
					(13.34%) (198) ring C-prop: C=O OUT
189	481.69	-------	6.85	1.41	(5.01%) (140) ring B-prop: 1-WAGG
					(6.47%) (142) ring B: bridge BC WAGG
					(9.01%) (196) ring C-prop: 3-BEND
190	452.32	-------	67.14	7.78	(-10.31%) (103) ring D torsion
					(68.36%) (201) ring D: N-H OUT
					(10.66%) (203) ring D: bridge CD WAGG
					(8.64%) (207) ring D-ethyl: WAGG
					(6.87%) (215) ring D-ethyl: BEND
191	439.33	-------	2.88	0.20	(6.53%) (5) ring A: C-C stretch (51,49)
					(5.38%) (9) ring A-methyl: C-C stretch (54,49)
					(5.44%) (13) ring A-ethyl: C-C stretch (58,51)
					(10.04%) (109) ring A-methyl: ROCK
					(5.02%) (112) ring A-ethyl: SCIS
					(10.17%) (113) ring A-ethyl: ROCK
					(5.56%) (123) ring A-ethyl: BEND
192	431.26	-------	10.40	0.72	(6.82%) (155) ring B-prop: 2-CH2-ROCK
					(28.78%) (158) ring B-prop: 3-BEND
					(5.05%) (174) ring C: bridge BC WAGG
					(6.16%) (196) ring C-prop: 3-BEND
193	404.26	-------	4.66	1.11	(5.91%) (136) ring B: bridge AB WAGG
					(7.58%) (138) ring B-methyl: WAGG
					(13.81%) (158) ring B-prop: 3-BEND
					(7.14%) (174) ring C: bridge BC WAGG
					(6.34%) (180) ring C: bridge CD WAGG
					(5.55%) (196) ring C-prop: 3-BEND

194	401.20	-------	9.67	0.06	(5.17%) (88) ring A bending
					(5.18%) (89) ring A bending
					(9.11%) (106) ring A: C=O ROCK
					(19.97%) (123) ring A-ethyl: BEND
195	361.05	-------	0.20	0.76	(8.43%) (178) ring C-methyl: WAGG
					(8.46%) (179) ring C: bridge CD ROCK
					(7.41%) (205) ring D-methyl: WAGG
196	351.45	-------	2.17	1.13	(7.16%) (175) ring C-prop: 1-ROCK
					(24.67%) (178) ring C-methyl: WAGG
					(7.75%) (186) ring C-prop: 1-BEND
					(13.54%) (196) ring C-prop: 3-BEND
					(6.97%) (205) ring D-methyl: WAGG
197	342.99	-------	5.69	0.93	(5.53%) (123) ring A-ethyl: BEND
					(10.24%) (137) ring B-methyl: ROCK
					(9.22%) (138) ring B-methyl: WAGG
					(21.98%) (139) ring B-prop: 1-ROCK
					(5.74%) (153) ring B-prop: 2-BEND
198	327.48	-------	0.93	6.74	(8.13%) (137) ring B-methyl: ROCK
					(29.70%) (138) ring B-methyl: WAGG
					(6.73%) (139) ring B-prop: 1-ROCK
					(5.15%) (140) ring B-prop: 1-WAGG
					(11.01%) (158) ring B-prop: 3-BEND
199	320.77	-------	2.36	23.45	(13.27%) (175) ring C-prop: 1-ROCK
					(5.24%) (191) ring C-prop: 2-BEND
					(5.39%) (203) ring D: bridge CD WAGG
					(11.16%) (205) ring D-methyl: WAGG
					(8.99%) (215) ring D-ethyl: BEND
200	317.61	-------	2.66	0.08	(5.02%) (76) ring D: C-C stretch (33,32)
					(42.97%) (204) ring D-methyl: ROCK
					(11.59%) (205) ring D-methyl: WAGG
					(8.62%) (208) ring D: C=O ROCK
					(-5.07%) (237) bridge C-D: TORS
201	305.15	-------	2.19	5.37	(10.31%) (95) ring B torsion
					(5.28%) (99) ring C torsion
					(21.91%) (177) ring C-methyl: ROCK
					(12.49%) (243) bridge C=D: TORS
					(5.58%) (246) ring D-ethyl: 2-TORS
202	294.81	-------	0.97	1.39	(16.15%) (178) ring C-methyl: WAGG
					(12.15%) (206) ring D-ethyl: ROCK
					(23.28%) (246) ring D-ethyl: 2-TORS
203	292.64	-------	2.24	6.62	(6.28%) (175) ring C-prop: 1-ROCK
					(7.39%) (177) ring C-methyl: ROCK
					(5.35%) (191) ring C-prop: 2-BEND
					(7.41%) (204) ring D-methyl: ROCK
204	288.39	-------	2.69	11.42	(6.26%) (204) ring D-methyl: ROCK
					(10.60%) (206) ring D-ethyl: ROCK
					(5.32%) (208) ring D: C=O ROCK
					(14.17%) (246) ring D-ethyl: 2-TORS
205	280.39	-------	2.23	4.16	(5.61%) (111) ring A-methyl: TWIST
					(15.57%) (123) ring A-ethyl: BEND
					(5.41%) (137) ring B-methyl: ROCK
206	269.89	-------	0.64	0.14	(7.22%) (177) ring C-methyl: ROCK
					(23.19%) (227) ring A-ethyl: 2-TORS
207	257.10	-------	2.91	0.49	(10.12%) (109) ring A-methyl: ROCK
					(7.32%) (225) ring A-methyl: TORS
					(22.33%) (227) ring A-ethyl: 2-TORS
208	247.75	-------	1.62	0.30	(6.63%) (110) ring A-methyl: WAGG
					(7.52%) (137) ring B-methyl: ROCK
					(5.41%) (138) ring B-methyl: WAGG
					(23.48%) (225) ring A-methyl: TORS
209	238.12	-------	0.61	0.27	(11.06%) (111) ring A-methyl: TWIST
					(55.14%) (225) ring A-methyl: TORS
210	234.01	-------	1.81	0.50	(6.63%) (202) ring D: bridge CD ROCK
					(9.50%) (225) ring A-methyl: TORS
					(12.40%) (227) ring A-ethyl: 2-TORS
					(5.57%) (234) bridge A=B: TORS
211	224.87	-------	2.52	0.72	(7.20%) (135) ring B: bridge AB ROCK
					(5.85%) (137) ring B-methyl: ROCK
					(5.07%) (202) ring D: bridge CD ROCK
					(6.39%) (227) ring A-ethyl: 2-TORS
					(34.67%) (238) ring C-methyl: TORS
212	220.29	-------	0.81	1.10	(36.91%) (238) ring C-methyl: TORS
					(8.83%) (244) ring D-methyl: TORS
					(8.89%) (246) ring D-ethyl: 2-TORS

213	216.55	-------	1.46	0.49	(9.69%) (135) ring B: bridge AB ROCK
					(10.82%) (137) ring B-methyl: ROCK
					(11.06%) (153) ring B-prop: 2-BEND
					(10.51%) (246) ring D-ethyl: 2-TORS
214	209.68	-------	2.40	0.65	(6.17%) (176) ring C-prop: 1-WAGG
					(9.63%) (206) ring D-ethyl: ROCK
					(14.14%) (246) ring D-ethyl: 2-TORS
215	197.60	-------	4.16	2.04	(8.42%) (91) ring A torsion
					(7.34%) (141) ring B: bridge BC ROCK
					(5.07%) (206) ring D-ethyl: ROCK
					(6.89%) (235) bridge A-B: TORS
					(6.65%) (246) ring D-ethyl: 2-TORS
					(11.53%) (248) Cl-H stretch
216	188.74	-------	1.88	2.77	(5.24%) (91) ring A torsion
					(8.06%) (153) ring B-prop: 2-BEND
					(5.98%) (186) ring C-prop: 1-BEND
					(5.74%) (227) ring A-ethyl: 2-TORS
217	185.15	-------	2.46	2.01	(8.24%) (102) ring D torsion
					(6.92%) (205) ring D-methyl: WAGG
					(6.01%) (207) ring D-ethyl: WAGG
					(8.22%) (215) ring D-ethyl: BEND
					(6.08%) (237) bridge C-D: TORS
					(15.63%) (248) Cl-H stretch
218	180.23	-------	3.81	0.23	(10.17%) (91) ring A torsion
					(5.55%) (123) ring A-ethyl: BEND
219	169.12	-------	3.79	0.28	(9.03%) (91) ring A torsion
					(6.49%) (95) ring B torsion
					(5.90%) (109) ring A-methyl: ROCK
					(6.79%) (162) bridge BC: BEND
					(14.31%) (235) bridge A-B: TORS
					(13.06%) (248) Cl-H stretch
220	167.19	-------	5.46	2.37	(5.63%) (91) ring A torsion
					(9.51%) (207) ring D-ethyl: WAGG
					(9.58%) (215) ring D-ethyl: BEND
					(30.73%) (248) Cl-H stretch
221	164.87	-------	0.06	1.04	(6.45%) (98) ring C torsion
					(5.02%) (191) ring C-prop: 2-BEND
					(13.45%) (237) bridge C-D: TORS
					(6.56%) (238) ring C-methyl: TORS
					(8.15%) (243) bridge C=D: TORS
					(8.16%) (244) ring D-methyl: TORS
					(5.67%) (248) Cl-H stretch
222	151.23	-------	1.11	6.60	(8.03%) (103) ring D torsion
					(8.80%) (153) ring B-prop: 2-BEND
					(5.28%) (237) bridge C-D: TORS
					(15.56%) (247) Cl-H stretch
223	146.97	-------	5.41	0.35	(12.07%) (91) ring A torsion
					(6.69%) (115) ring A-ethyl: TWIST
					(-6.89%) (235) bridge A-B: TORS
					(31.00%) (247) Cl-H stretch
					(8.61%) (249) Cl OUT
224	140.79	-------	0.96	1.17	(5.60%) (91) ring A torsion
					(5.25%) (102) ring D torsion
					(12.52%) (103) ring D torsion
					(5.65%) (141) ring B: bridge BC ROCK
					(19.69%) (228) ring B-methyl: TORS
225	137.81	-------	3.50	0.20	(12.70%) (103) ring D torsion
					(18.55%) (228) ring B-methyl: TORS
					(10.22%) (244) ring D-methyl: TORS
					(5.15%) (247) Cl-H stretch
226	131.75	-------	0.98	0.42	(5.17%) (226) ring A-ethyl: 1-TORS
					(49.79%) (228) ring B-methyl: TORS
					(12.87%) (244) ring D-methyl: TORS
227	127.18	-------	0.54	0.27	(5.83%) (116) ring A: bridge AB ROCK
					(5.48%) (168) bridge AB: BEND
					(6.28%) (175) ring C-prop: 1-ROCK
					(16.85%) (226) ring A-ethyl: 1-TORS
					(6.32%) (247) Cl-H stretch
228	122.15	-------	0.84	4.48	(7.23%) (174) ring C: bridge BC WAGG
					(37.69%) (244) ring D-methyl: TORS
229	104.50	-------	1.08	1.37	(5.54%) (102) ring D torsion
					(6.75%) (173) ring C: bridge BC ROCK
					(30.19%) (226) ring A-ethyl: 1-TORS
230	101.75	-------	0.27	6.25	(5.44%) (98) ring C torsion
					(22.29%) (102) ring D torsion
					(9.66%) (207) ring D-ethyl: WAGG

					(14.74%) (226) ring A-ethyl: 1-TORS
					(6.10%) (237) bridge C-D: TORS
231	90.53	-------	0.86	0.71	(17.11%) (94) ring B torsion
					(6.27%) (117) ring A: bridge AB WAGG
					(9.27%) (140) ring B-prop: 1-WAGG
					(6.74%) (148) ring B-prop: 1-BEND
					(7.29%) (229) ring B-prop: 1-TORS
					(5.66%) (233) bridge BC: 1-TORS
					(5.25%) (247) Cl-H stretch
232	80.18	-------	1.07	1.06	(5.12%) (103) ring D torsion
					(13.17%) (116) ring A: bridge AB ROCK
					(5.66%) (135) ring B: bridge AB ROCK
					(15.03%) (168) bridge AB: BEND
					(11.70%) (226) ring A-ethyl: 1-TORS
					(5.26%) (235) bridge A-B: TORS
					(6.50%) (239) ring C-prop: 1-TORS
					(6.47%) (240) ring C-prop: 2-TORS
					(13.23%) (249) Cl OUT
233	76.25	-------	0.45	1.06	(5.38%) (103) ring D torsion
					(6.45%) (142) ring B: bridge BC WAGG
					(6.20%) (233) bridge BC: 1-TORS
					(22.03%) (239) ring C-prop: 1-TORS
234	69.00	-------	2.79	1.34	(12.68%) (229) ring B-prop: 1-TORS
					(8.49%) (234) bridge A=B: TORS
					(9.44%) (236) bridge BC: 2-TORS
					(6.81%) (237) bridge C-D: TORS
					(12.14%) (249) Cl OUT
235	61.49	-------	0.81	0.29	(5.76%) (102) ring D torsion
					(5.96%) (229) ring B-prop: 1-TORS
					(5.69%) (236) bridge BC: 2-TORS
					(16.39%) (240) ring C-prop: 2-TORS
					(10.57%) (249) Cl OUT
236	58.96	-------	0.02	2.83	(6.43%) (162) bridge BC: BEND
					(10.75%) (165) bridge CD: BEND
					(13.72%) (229) ring B-prop: 1-TORS
					(11.52%) (231) ring B-prop: 3-TORS
					(5.32%) (245) ring D-ethyl: 1-TORS
237	45.86	-------	4.03	0.49	(5.03%) (98) ring C torsion
					(9.13%) (229) ring B-prop: 1-TORS
					(33.08%) (230) ring B-prop: 2-TORS
					(11.26%) (231) ring B-prop: 3-TORS
					(12.43%) (241) ring C-prop: 3-TORS
238	44.33	-------	0.67	0.28	(6.23%) (180) ring C: bridge CD WAGG
					(9.99%) (230) ring B-prop: 2-TORS
					(36.75%) (231) ring B-prop: 3-TORS
					(13.33%) (241) ring C-prop: 3-TORS
239	41.47	-------	0.28	1.87	(5.10%) (241) ring C-prop: 3-TORS
					(5.56%) (243) bridge C=D: TORS
					(56.79%) (245) ring D-ethyl: 1-TORS
240	36.21	-------	0.93	0.75	(40.68%) (90) ring A torsion
					(7.22%) (234) bridge A=B: TORS
					(11.39%) (235) bridge A-B: TORS
					(5.18%) (241) ring C-prop: 3-TORS
					(9.81%) (245) ring D-ethyl: 1-TORS
241	29.93	-------	1.26	3.68	(11.21%) (90) ring A torsion
					(5.21%) (230) ring B-prop: 2-TORS
					(5.23%) (233) bridge BC: 1-TORS
					(10.29%) (237) bridge C-D: TORS
					(27.90%) (241) ring C-prop: 3-TORS
					(9.81%) (249) Cl OUT
242	28.60	-------	0.13	4.38	(8.60%) (90) ring A torsion
					(5.70%) (94) ring B torsion
					(10.17%) (136) ring B: bridge AB WAGG
					(7.51%) (140) ring B-prop: 1-WAGG
					(10.76%) (229) ring B-prop: 1-TORS
					(13.35%) (234) bridge A=B: TORS
					(7.55%) (239) ring C-prop: 1-TORS
243	26.79	-------	1.20	0.98	(6.24%) (140) ring B-prop: 1-WAGG
					(15.86%) (229) ring B-prop: 1-TORS
					(18.64%) (230) ring B-prop: 2-TORS
					(21.00%) (239) ring C-prop: 1-TORS
					(11.83%) (240) ring C-prop: 2-TORS
244	24.19	-------	0.25	2.01	(6.34%) (229) ring B-prop: 1-TORS
					(19.80%) (230) ring B-prop: 2-TORS
					(12.96%) (231) ring B-prop: 3-TORS
					(19.26%) (240) ring C-prop: 2-TORS
					(5.10%) (243) bridge C=D: TORS
					(6.96%) (245) ring D-ethyl: 1-TORS

245	22.03	-------	1.31	6.53	(5.51%)	(103)	ring D torsion
					(5.13%)	(167)	bridge CD: C-H OUT
					(6.06%)	(230)	ring B-prop: 2-TORS
					(14.45%)	(237)	bridge C-D: TORS
					(13.01%)	(239)	ring C-prop: 1-TORS
					(9.64%)	(241)	ring C-prop: 3-TORS
					(9.78%)	(249)	Cl OUT
246	19.73	-------	0.11	4.74	(7.95%)	(162)	bridge BC: BEND
					(11.34%)	(165)	bridge CD: BEND
					(9.65%)	(179)	ring C: bridge CD ROCK
					(6.27%)	(240)	ring C-prop: 2-TORS
					(9.39%)	(245)	ring D-ethyl: 1-TORS
247	15.36	-------	0.17	2.75	(21.27%)	(90)	ring A torsion
					(26.46%)	(235)	bridge A-B: TORS
					(8.48%)	(236)	bridge BC: 2-TORS
248	12.06	-------	0.92	31.71	(5.99%)	(162)	bridge BC: BEND
					(12.01%)	(174)	ring C: bridge BC WAGG
					(8.09%)	(233)	bridge BC: 1-TORS
					(28.88%)	(237)	bridge C-D: TORS
					(7.82%)	(249)	Cl OUT
249	10.62	-------	0.01	5.61	(5.28%)	(90)	ring A torsion
					(5.03%)	(136)	ring B: bridge AB WAGG
					(15.14%)	(233)	bridge BC: 1-TORS
					(5.25%)	(235)	bridge A-B: TORS
					(23.26%)	(236)	bridge BC: 2-TORS

ZZEssa, $^{13}C(5)$, D_2O

	calc. [cm-1]	exp. [cm-1]	IRint [km/mol]	Rint (rel.)	contribution
1	3522.77	-------	54.89	0.14	(100.07%) (67) ring C-prop: O-H stretch (27,84)
2	3516.59	-------	56.06	0.17	(100.07%) (39) ring B-prop: O-H stretch (85,83)
3	3067.40	-------	0.44	0.17	(99.76%) (42) bridge BC: C-H stretch (4, 3)
4	3047.57	-------	18.55	0.24	(99.87%) (46) bridge AB: C-H stretch (8, 7)
5	3013.51	-------	6.62	0.09	(50.09%) (56) ring C-methyl: C-H stretch (16,15)<br>(46.83%) (57) ring C-methyl: C-H stretch (17,15)
6	3012.89	-------	13.93	0.10	(79.24%) (78) ring D-methyl: C-H stretch (35,34)<br>(5.51%) (79) ring D-methyl: C-H stretch (36,34)<br>(12.73%) (80) ring D-methyl: C-H stretch (37,34)
7	3012.05	-------	14.02	0.06	(98.37%) (43) bridge CD: C-H stretch (6, 5)
8	3006.08	-------	18.98	0.07	(9.15%) (85) ring D-ethyl: C-H stretch (42,41)<br>(11.25%) (86) ring D-ethyl: C-H stretch (43,41)<br>(75.62%) (87) ring D-ethyl: C-H stretch (44,41)
9	3003.59	-------	10.89	0.08	(16.39%) (10) ring A-methyl: C-H stretch (55,54)<br>(6.14%) (11) ring A-methyl: C-H stretch (56,54)<br>(77.33%) (12) ring A-methyl: C-H stretch (57,54)
10	3000.91	-------	15.26	0.03	(6.78%) (60) ring C-prop: C-H stretch (20,19)<br>(71.63%) (61) ring C-prop: C-H stretch (21,19)<br>(19.66%) (63) ring C-prop: C-H stretch (23,22)
11	2998.99	-------	34.29	0.12	(13.30%) (27) ring B-methyl: C-H stretch (72,71)<br>(7.80%) (31) ring B-prop: C-H stretch (76,75)<br>(9.99%) (32) ring B-prop: C-H stretch (77,75)<br>(60.07%) (34) ring B-prop: C-H stretch (79,78)<br>(7.03%) (35) ring B-prop: C-H stretch (80,78)
12	2996.58	-------	11.91	0.10	(73.59%) (27) ring B-methyl: C-H stretch (72,71)<br>(5.57%) (29) ring B-methyl: C-H stretch (74,71)<br>(13.70%) (34) ring B-prop: C-H stretch (79,78)
13	2987.26	-------	28.00	0.16	(31.92%) (17) ring A-ethyl: C-H stretch (62,60)<br>(58.87%) (18) ring A-ethyl: C-H stretch (63,60)
14	2985.50	-------	50.71	0.40	(51.26%) (85) ring D-ethyl: C-H stretch (42,41)<br>(45.14%) (86) ring D-ethyl: C-H stretch (43,41)
15	2984.68	-------	1.10	0.06	(18.40%) (61) ring C-prop: C-H stretch (21,19)<br>(70.81%) (63) ring C-prop: C-H stretch (23,22)<br>(7.85%) (64) ring C-prop: C-H stretch (24,22)
16	2984.25	-------	45.06	0.15	(45.86%) (10) ring A-methyl: C-H stretch (55,54)<br>(43.19%) (11) ring A-methyl: C-H stretch (56,54)<br>(5.31%) (18) ring A-ethyl: C-H stretch (63,60)
17	2981.34	-------	8.87	0.06	(8.43%) (56) ring C-methyl: C-H stretch (16,15)<br>(10.04%) (57) ring C-methyl: C-H stretch (17,15)<br>(8.85%) (58) ring C-methyl: C-H stretch (18,15)<br>(36.13%) (79) ring D-methyl: C-H stretch (36,34)<br>(36.00%) (80) ring D-methyl: C-H stretch (37,34)
18	2979.96	-------	8.82	0.13	(27.76%) (56) ring C-methyl: C-H stretch (16,15)<br>(27.08%) (57) ring C-methyl: C-H stretch (17,15)<br>(17.98%) (58) ring C-methyl: C-H stretch (18,15)<br>(12.91%) (79) ring D-methyl: C-H stretch (36,34)<br>(13.86%) (80) ring D-methyl: C-H stretch (37,34)
19	2979.84	-------	36.00	0.07	(5.96%) (14) ring A-ethyl: C-H stretch (59,58)<br>(46.59%) (16) ring A-ethyl: C-H stretch (61,60)<br>(28.79%) (17) ring A-ethyl: C-H stretch (62,60)<br>(11.07%) (18) ring A-ethyl: C-H stretch (63,60)<br>(6.25%) (40) ring A-ethyl: C-H stretch (1,58)
20	2977.85	-------	4.42	0.04	(46.44%) (31) ring B-prop: C-H stretch (76,75)<br>(35.12%) (32) ring B-prop: C-H stretch (77,75)<br>(16.51%) (34) ring B-prop: C-H stretch (79,78)
21	2956.12	-------	10.79	0.09	(56.79%) (82) ring D-ethyl: C-H stretch (39,38)<br>(36.28%) (83) ring D-ethyl: C-H stretch (40,38)
22	2954.93	-------	14.72	0.15	(48.15%) (28) ring B-methyl: C-H stretch (73,71)<br>(52.00%) (29) ring B-methyl: C-H stretch (74,71)
23	2949.88	-------	6.97	0.08	(52.30%) (14) ring A-ethyl: C-H stretch (59,58)<br>(9.43%) (17) ring A-ethyl: C-H stretch (62,60)<br>(28.60%) (40) ring A-ethyl: C-H stretch (1,58)
24	2947.13	-------	19.19	0.19	(43.74%) (31) ring B-prop: C-H stretch (76,75)<br>(52.11%) (32) ring B-prop: C-H stretch (77,75)

25	2941.13	-------	8.19	0.19	(93.98%) (4) ring A: C-H stretch (50,49)
26	2936.45	-------	15.92	0.29	(75.58%) (60) ring C-prop: C-H stretch (20,19)
					(8.49%) (61) ring C-prop: C-H stretch (21,19)
					(13.81%) (64) ring C-prop: C-H stretch (24,22)
27	2930.97	-------	24.20	0.43	(17.31%) (78) ring D-methyl: C-H stretch (35,34)
					(43.16%) (79) ring D-methyl: C-H stretch (36,34)
					(36.55%) (80) ring D-methyl: C-H stretch (37,34)
28	2928.56	-------	2.42	0.13	(8.79%) (34) ring B-prop: C-H stretch (79,78)
					(87.43%) (35) ring B-prop: C-H stretch (80,78)
29	2925.03	-------	11.07	0.09	(14.89%) (60) ring C-prop: C-H stretch (20,19)
					(7.17%) (63) ring C-prop: C-H stretch (23,22)
					(76.98%) (64) ring C-prop: C-H stretch (24,22)
30	2922.01	-------	45.86	0.25	(38.41%) (85) ring D-ethyl: C-H stretch (42,41)
					(38.28%) (86) ring D-ethyl: C-H stretch (43,41)
					(19.06%) (87) ring D-ethyl: C-H stretch (44,41)
31	2921.79	-------	70.45	0.34	(29.50%) (6) ring A: C-H stretch (52,51)
					(15.90%) (10) ring A-methyl: C-H stretch (55,54)
					(23.86%) (11) ring A-methyl: C-H stretch (56,54)
					(10.92%) (12) ring A-methyl: C-H stretch (57,54)
					(9.71%) (14) ring A-ethyl: C-H stretch (59,58)
32	2919.15	-------	2.76	0.03	(16.39%) (6) ring A: C-H stretch (52,51)
					(15.15%) (10) ring A-methyl: C-H stretch (55,54)
					(21.22%) (11) ring A-methyl: C-H stretch (56,54)
					(8.71%) (12) ring A-methyl: C-H stretch (57,54)
					(15.18%) (14) ring A-ethyl: C-H stretch (59,58)
					(14.50%) (40) ring A-ethyl: C-H stretch (1,58)
33	2918.33	-------	21.75	0.22	(37.09%) (82) ring D-ethyl: C-H stretch (39,38)
					(57.06%) (83) ring D-ethyl: C-H stretch (40,38)
34	2917.21	-------	39.67	0.27	(11.68%) (56) ring C-methyl: C-H stretch (16,15)
					(13.90%) (57) ring C-methyl: C-H stretch (17,15)
					(70.90%) (58) ring C-methyl: C-H stretch (18,15)
35	2915.75	-------	27.28	0.10	(9.85%) (6) ring A: C-H stretch (52,51)
					(41.08%) (16) ring A-ethyl: C-H stretch (61,60)
					(26.18%) (17) ring A-ethyl: C-H stretch (62,60)
					(18.90%) (18) ring A-ethyl: C-H stretch (63,60)
36	2911.29	-------	6.30	0.19	(39.41%) (6) ring A: C-H stretch (52,51)
					(11.35%) (14) ring A-ethyl: C-H stretch (59,58)
					(44.72%) (40) ring A-ethyl: C-H stretch (1,58)
37	2909.72	-------	43.64	0.45	(12.35%) (27) ring B-methyl: C-H stretch (72,71)
					(45.89%) (28) ring B-methyl: C-H stretch (73,71)
					(41.12%) (29) ring B-methyl: C-H stretch (74,71)
38	2561.74	-------	38.36	0.13	(98.25%) (69) ring D: N-H stretch (29,28)
39	2302.25	-------	441.95	0.11	(34.76%) (1) ring A: N-H stretch (47,46)
					(33.04%) (20) ring B: N-H stretch (66,65)
					(29.51%) (47) ring C: N-H stretch (10, 9)
40	2244.20	-------	370.34	0.44	(58.21%) (1) ring A: N-H stretch (47,46)
					(6.13%) (20) ring B: N-H stretch (66,65)
					(33.24%) (47) ring C: N-H stretch (10, 9)
41	2232.11	-------	97.19	0.07	(59.19%) (20) ring B: N-H stretch (66,65)
					(35.36%) (47) ring C: N-H stretch (10, 9)
42	1779.45	-------	286.44	1.32	(85.68%) (19) ring A: C=O stretch (64,48)
43	1774.15	-------	188.68	0.04	(81.27%) (66) ring C-prop: C=O stretch (26,25)
					(6.41%) (199) ring C-prop: C-O-H BEND
44	1760.70	-------	254.34	0.02	(81.10%) (37) ring B-prop: C=O stretch (82,81)
					(6.53%) (161) ring B-prop: C-O-H BEND
45	1737.04	-------	871.28	4.65	(82.12%) (74) ring D: C=O stretch (33,45)
					(5.39%) (100) ring D bending
46	1617.18	-------	169.63	100.00	(8.02%) (52) bridge CD: C-C stretch (14, 5)
					(57.73%) (70) bridge CD: C=C stretch (30, 5)
					(5.54%) (71) ring D: C-N stretch (30,28)
					(10.98%) (166) bridge CD: C-H ROCK
47	1613.47	-------	11.45	2.51	(68.62%) (73) ring D: C=C stretch (32,31)
					(6.08%) (77) ring D-methyl: C-C stretch (34,31)
					(8.27%) (81) ring D-ethyl: C-C stretch (38,32)
48	1603.86	-------	856.58	16.10	(27.06%) (41) bridge BC: C-.C stretch (3,70)
					(8.48%) (44) bridge AB: C=C stretch (7,53)
					(22.76%) (48) bridge BC: C-.C stretch (11, 3)
					(17.14%) (163) bridge BC: C-H ROCK
49	1577.13	-------	483.72	5.57	(5.26%) (7) ring A: C-N stretch (53,46)

					(32.69%) (23) ring B: C-.C stretch (69,68)
					(21.58%) (44) bridge AB: C=C stretch (7,53)
					(5.30%) (169) bridge AB: C-H ROCK
50	1531.99	-------	1875.73	14.37	(17.42%) (23) ring B: C-.C stretch (69,68)
					(22.10%) (44) bridge AB: C=C stretch (7,53)
					(8.81%) (45) bridge AB: C-C stretch (7,67)
					(5.42%) (92) ring B bending
51	1506.71	-------	237.73	2.55	(36.74%) (51) ring C: C-.C stretch (13,12)
					(7.04%) (54) ring C: C-C stretch (14,13)
					(10.38%) (55) ring C-methyl: C-C stretch (15,13)
52	1490.07	-------	68.96	8.80	(6.95%) (54) ring C: C-C stretch (14,13)
					(51.82%) (182) ring C-methyl: ADEFa
					(8.86%) (184) ring C-methyl: ROCKa
53	1483.17	-------	12.42	6.60	(18.17%) (7) ring A: C-N stretch (53,46)
					(11.74%) (22) ring B: C-C stretch (68,67)
					(14.66%) (45) bridge AB: C-C stretch (7,67)
					(19.09%) (169) bridge AB: C-H ROCK
54	1477.20	-------	6.67	0.19	(5.93%) (216) ring D-ethyl: CH2 SCIS
					(72.69%) (221) ring D-ethyl: CH3 ADEFa
					(10.35%) (222) ring D-ethyl: CH3 ADEFb
					(6.72%) (223) ring D-ethyl: CH3 ROCKa
55	1476.70	-------	72.56	0.56	(9.02%) (145) ring B-methyl: ADEFb
					(65.70%) (149) ring B-prop: 1-CH2-SCIS
56	1473.57	-------	5.08	0.05	(14.25%) (124) ring A-ethyl: CH2 SCIS
					(44.37%) (129) ring A-ethyl: CH3 ADEFa
					(25.76%) (130) ring A-ethyl: CH3 ADEFb
57	1468.65	-------	4.73	0.11	(20.86%) (119) ring A-methyl: ADEFa
					(38.96%) (129) ring A-ethyl: CH3 ADEFa
					(25.46%) (130) ring A-ethyl: CH3 ADEFb
58	1467.21	-------	10.87	0.14	(31.22%) (119) ring A-methyl: ADEFa
					(31.29%) (120) ring A-methyl: ADEFb
					(5.12%) (124) ring A-ethyl: CH2 SCIS
					(20.71%) (130) ring A-ethyl: CH3 ADEFb
59	1466.12	-------	2.73	1.79	(34.47%) (183) ring C-methyl: ADEFb
					(20.16%) (187) ring C-prop: 1-CH2-SCIS
					(14.55%) (212) ring D-methyl: ADEFb
60	1465.05	-------	4.46	0.11	(34.26%) (119) ring A-methyl: ADEFa
					(50.27%) (120) ring A-methyl: ADEFb
61	1463.94	-------	16.63	0.25	(6.44%) (221) ring D-ethyl: CH3 ADEFa
					(77.66%) (222) ring D-ethyl: CH3 ADEFb
					(7.19%) (224) ring D-ethyl: CH3 ROCKb
62	1462.55	-------	42.92	3.57	(8.47%) (144) ring B-methyl: ADEFa
					(5.54%) (145) ring B-methyl: ADEFb
					(7.19%) (149) ring B-prop: 1-CH2-SCIS
					(6.06%) (183) ring C-methyl: ADEFb
					(9.11%) (211) ring D-methyl: ADEFa
					(22.19%) (212) ring D-methyl: ADEFb
63	1458.34	-------	0.60	3.88	(32.26%) (144) ring B-methyl: ADEFa
					(17.40%) (145) ring B-methyl: ADEFb
					(13.54%) (211) ring D-methyl: ADEFa
64	1456.60	-------	16.56	0.12	(18.31%) (187) ring C-prop: 1-CH2-SCIS
					(35.18%) (211) ring D-methyl: ADEFa
					(25.76%) (212) ring D-methyl: ADEFb
65	1454.46	-------	2.25	0.19	(73.07%) (124) ring A-ethyl: CH2 SCIS
					(15.05%) (130) ring A-ethyl: CH3 ADEFb
66	1453.15	-------	25.83	0.90	(26.68%) (144) ring B-methyl: ADEFa
					(28.83%) (145) ring B-methyl: ADEFb
					(15.27%) (183) ring C-methyl: ADEFb
					(5.69%) (187) ring C-prop: 1-CH2-SCIS
					(5.51%) (212) ring D-methyl: ADEFb
67	1452.44	-------	10.39	0.35	(12.28%) (144) ring B-methyl: ADEFa
					(16.95%) (145) ring B-methyl: ADEFb
					(26.82%) (183) ring C-methyl: ADEFb
					(21.74%) (187) ring C-prop: 1-CH2-SCIS
					(5.87%) (212) ring D-methyl: ADEFb
68	1450.39	-------	87.40	6.86	(18.69%) (182) ring C-methyl: ADEFa
					(11.66%) (187) ring C-prop: 1-CH2-SCIS
					(24.82%) (211) ring D-methyl: ADEFa
69	1444.97	-------	8.60	1.21	(61.66%) (216) ring D-ethyl: CH2 SCIS
					(7.61%) (221) ring D-ethyl: CH3 ADEFa
70	1443.78	-------	11.23	4.61	(10.88%) (52) bridge CD: C-C stretch (14, 5)
					(8.26%) (54) ring C: C-C stretch (14,13)

					(17.89%) (216) ring D-ethyl: CH2 SCIS
71	1438.15	-------	16.88	0.97	(83.36%) (154) ring B-prop: 2-CH2-SCIS
72	1435.52	-------	12.59	11.41	(22.11%) (21) ring B: C-N stretch (67,65)
					(7.06%) (23) ring B: C-.C stretch (69,68)
					(8.10%) (45) bridge AB: C-C stretch (7,67)
					(7.35%) (154) ring B-prop: 2-CH2-SCIS
					(20.93%) (169) bridge AB: C-H ROCK
73	1426.65	-------	155.13	1.13	(5.63%) (48) bridge BC: C-.C stretch (11, 3)
					(12.28%) (49) ring C: C-N stretch (11, 9)
					(6.66%) (97) ring C bending
					(14.66%) (163) bridge BC: C-H ROCK
					(21.47%) (192) ring C-prop: 2-CH2-SCIS
74	1423.13	-------	9.38	0.63	(8.24%) (163) bridge BC: C-H ROCK
					(63.08%) (192) ring C-prop: 2-CH2-SCIS
75	1410.72	-------	25.02	3.80	(7.42%) (24) ring B: C-N stretch (70,65)
					(7.56%) (93) ring B bending
					(6.71%) (97) ring C bending
					(6.25%) (163) bridge BC: C-H ROCK
					(5.71%) (166) bridge CD: C-H ROCK
76	1398.89	-------	63.04	6.65	(7.12%) (143) ring B-methyl: SDEF
					(5.18%) (166) bridge CD: C-H ROCK
					(15.53%) (194) ring C-prop: 2-CH2-WAGG
77	1398.75	-------	112.29	6.24	(5.06%) (65) ring C-prop: C-C stretch (25,22)
					(5.26%) (68) ring C-prop: C-O stretch (27,25)
					(6.44%) (143) ring B-methyl: SDEF
					(6.35%) (166) bridge CD: C-H ROCK
					(7.82%) (192) ring C-prop: 2-CH2-SCIS
					(16.08%) (194) ring C-prop: 2-CH2-WAGG
					(5.71%) (199) ring C-prop: C-O-H BEND
78	1394.98	-------	105.04	0.42	(10.60%) (36) ring B-prop: C-C stretch (81,78)
					(13.36%) (38) ring B-prop: C-O stretch (83,81)
					(24.86%) (156) ring B-prop: 2-CH2-WAGG
					(11.55%) (159) ring B-prop: C=O ROCK
					(16.67%) (161) ring B-prop: C-O-H BEND
79	1389.61	-------	9.78	2.12	(5.42%) (77) ring D-methyl: C-C stretch (34,31)
					(67.61%) (210) ring D-methyl: SDEF
80	1387.29	-------	20.97	3.67	(5.33%) (166) bridge CD: C-H ROCK
					(76.90%) (181) ring C-methyl: SDEF
81	1384.17	-------	1.44	0.02	(92.04%) (128) ring A-ethyl: CH3 SDEF
82	1380.25	-------	21.88	13.90	(65.93%) (143) ring B-methyl: SDEF
83	1376.36	-------	1.91	5.43	(51.39%) (118) ring A-methyl: SDEF
					(5.27%) (181) ring C-methyl: SDEF
					(5.23%) (210) ring D-methyl: SDEF
84	1374.30	-------	23.23	4.10	(42.37%) (118) ring A-methyl: SDEF
					(5.99%) (210) ring D-methyl: SDEF
					(10.19%) (220) ring D-ethyl: CH3 SDEF
85	1368.66	-------	8.19	8.62	(5.33%) (166) bridge CD: C-H ROCK
					(73.71%) (220) ring D-ethyl: CH3 SDEF
86	1354.30	-------	11.76	0.39	(6.12%) (22) ring B: C-C stretch (68,67)
					(7.22%) (53) ring C: C-N stretch (14, 9)
					(12.14%) (126) ring A-ethyl: CH2 WAGG
					(9.20%) (189) ring C-prop: 1-CH2-WAGG
87	1352.17	-------	12.76	0.67	(15.50%) (112) ring A-ethyl: SCIS
					(46.08%) (126) ring A-ethyl: CH2 WAGG
88	1342.69	-------	14.91	0.95	(5.09%) (76) ring D: C-C stretch (33,32)
					(7.05%) (81) ring D-ethyl: C-C stretch (38,32)
					(5.47%) (101) ring D bending
					(19.30%) (189) ring C-prop: 1-CH2-WAGG
					(25.68%) (218) ring D-ethyl: CH2 WAGG
89	1340.58	-------	28.27	1.40	(14.65%) (108) ring A-methyl: SCIS
					(10.00%) (112) ring A-ethyl: SCIS
					(7.95%) (114) ring A-ethyl: WAGG
					(10.42%) (126) ring A-ethyl: CH2 WAGG
					(10.18%) (127) ring A-ethyl: CH2 TWIST
					(5.83%) (151) ring B-prop: 1-CH2-WAGG
90	1337.32	-------	16.88	0.35	(45.81%) (151) ring B-prop: 1-CH2-WAGG
					(10.05%) (156) ring B-prop: 2-CH2-WAGG
					(9.83%) (161) ring B-prop: C-O-H BEND
91	1334.68	-------	29.95	0.66	(9.19%) (53) ring C: C-N stretch (14, 9)
					(36.24%) (189) ring C-prop: 1-CH2-WAGG
					(7.88%) (218) ring D-ethyl: CH2 WAGG

92	1317.87	-------	5.87	2.43	(10.05%)	(76)	ring D: C-C stretch (33,32)
					(7.73%)	(101)	ring D bending
					(41.36%)	(218)	ring D-ethyl: CH2 WAGG
					(10.33%)	(219)	ring D-ethyl: CH2 TWIST
93	1314.26	-------	25.36	0.03	(10.81%)	(190)	ring C-prop: 1-CH2-TWIST
					(23.92%)	(194)	ring C-prop: 2-CH2-WAGG
					(5.52%)	(197)	ring C-prop: C=O ROCK
					(38.17%)	(199)	ring C-prop: C-O-H BEND
94	1307.51	-------	17.16	0.05	(32.23%)	(108)	ring A-methyl: SCIS
					(5.76%)	(109)	ring A-methyl: ROCK
					(5.96%)	(112)	ring A-ethyl: SCIS
					(5.15%)	(121)	ring A-methyl: ROCKa
					(31.68%)	(127)	ring A-ethyl: CH2 TWIST
95	1294.01	-------	421.63	2.34	(18.94%)	(152)	ring B-prop: 1-CH2-TWIST
					(7.39%)	(161)	ring B-prop: C-O-H BEND
96	1290.66	-------	39.64	0.13	(26.87%)	(110)	ring A-methyl: WAGG
					(8.97%)	(111)	ring A-methyl: TWIST
					(10.53%)	(114)	ring A-ethyl: WAGG
					(5.54%)	(115)	ring A-ethyl: TWIST
					(5.36%)	(126)	ring A-ethyl: CH2 WAGG
97	1286.72	-------	207.37	1.59	(6.56%)	(151)	ring B-prop: 1-CH2-WAGG
					(12.42%)	(156)	ring B-prop: 2-CH2-WAGG
					(7.24%)	(157)	ring B-prop: 2-CH2-TWIST
					(13.93%)	(161)	ring B-prop: C-O-H BEND
98	1279.08	-------	2.54	0.13	(6.75%)	(53)	ring C: C-N stretch (14, 9)
					(5.85%)	(71)	ring D: C-N stretch (30,28)
					(38.62%)	(219)	ring D-ethyl: CH2 TWIST
					(9.29%)	(224)	ring D-ethyl: CH3 ROCKb
99	1261.90	-------	45.18	2.78	(25.76%)	(71)	ring D: C-N stretch (30,28)
					(5.47%)	(166)	bridge CD: C-H ROCK
					(6.46%)	(208)	ring D: C=O ROCK
					(16.28%)	(219)	ring D-ethyl: CH2 TWIST
					(6.21%)	(224)	ring D-ethyl: CH3 ROCKb
100	1256.63	-------	68.14	0.22	(7.32%)	(108)	ring A-methyl: SCIS
					(16.08%)	(112)	ring A-ethyl: SCIS
					(5.11%)	(113)	ring A-ethyl: ROCK
					(7.15%)	(125)	ring A-ethyl: CH2 ROCK
					(24.16%)	(127)	ring A-ethyl: CH2 TWIST
					(13.04%)	(131)	ring A-ethyl: CH3 ROCKa
101	1254.14	-------	20.15	0.38	(13.74%)	(151)	ring B-prop: 1-CH2-WAGG
					(21.16%)	(152)	ring B-prop: 1-CH2-TWIST
					(20.87%)	(156)	ring B-prop: 2-CH2-WAGG
					(13.04%)	(157)	ring B-prop: 2-CH2-TWIST
102	1242.33	-------	316.07	0.08	(11.30%)	(110)	ring A-methyl: WAGG
					(5.91%)	(111)	ring A-methyl: TWIST
					(6.96%)	(112)	ring A-ethyl: SCIS
					(10.93%)	(114)	ring A-ethyl: WAGG
					(7.00%)	(115)	ring A-ethyl: TWIST
					(13.76%)	(190)	ring C-prop: 1-CH2-TWIST
103	1237.34	-------	82.62	0.47	(5.16%)	(114)	ring A-ethyl: WAGG
					(5.01%)	(189)	ring C-prop: 1-CH2-WAGG
					(24.19%)	(190)	ring C-prop: 1-CH2-TWIST
					(6.25%)	(194)	ring C-prop: 2-CH2-WAGG
					(11.08%)	(195)	ring C-prop: 2-CH2-TWIST
104	1226.35	-------	433.64	1.82	(10.98%)	(2)	ring A: C-N stretch (48,46)
					(6.00%)	(3)	ring A: C-C stretch (49,48)
					(7.18%)	(88)	ring A bending
					(5.36%)	(106)	ring A: C=O ROCK
					(5.93%)	(157)	ring B-prop: 2-CH2-TWIST
					(6.53%)	(169)	bridge AB: C-H ROCK
105	1213.39	-------	157.23	0.51	(18.49%)	(2)	ring A: C-N stretch (48,46)
					(12.83%)	(7)	ring A: C-N stretch (53,46)
					(12.34%)	(44)	bridge AB: C=C stretch (7,53)
					(10.14%)	(104)	ring A: N-H ROCK
					(13.05%)	(169)	bridge AB: C-H ROCK
106	1212.72	-------	138.26	3.06	(5.72%)	(55)	ring C-methyl: C-C stretch (15,13)
					(13.39%)	(75)	ring D: C-N stretch (33,28)
					(9.31%)	(166)	bridge CD: C-H ROCK
					(26.18%)	(195)	ring C-prop: 2-CH2-TWIST
107	1202.82	-------	81.23	0.33	(15.90%)	(152)	ring B-prop: 1-CH2-TWIST
					(20.59%)	(157)	ring B-prop: 2-CH2-TWIST
					(5.21%)	(161)	ring B-prop: C-O-H BEND
					(6.28%)	(190)	ring C-prop: 1-CH2-TWIST
					(15.74%)	(195)	ring C-prop: 2-CH2-TWIST
108	1191.82	-------	674.97	3.76	(5.34%)	(75)	ring D: C-N stretch (33,28)
					(5.31%)	(152)	ring B-prop: 1-CH2-TWIST
					(6.22%)	(190)	ring C-prop: 1-CH2-TWIST

					(9.56%) (195) ring C-prop: 2-CH2-TWIST
109	1160.89	-------	76.63	0.63	(12.96%) (49) ring C: C-N stretch (11, 9)
					(6.50%) (55) ring C-methyl: C-C stretch (15,13)
					(5.55%) (59) ring C-prop: C-C stretch (19,12)
					(16.54%) (75) ring D: C-N stretch (33,28)
					(7.28%) (97) ring C bending
110	1152.94	-------	184.88	0.20	(10.16%) (38) ring B-prop: C-O stretch (83,81)
					(16.91%) (68) ring C-prop: C-O stretch (27,25)
					(8.11%) (199) ring C-prop: C-O-H BEND
111	1150.41	-------	200.39	1.03	(16.26%) (38) ring B-prop: C-O stretch (83,81)
					(6.10%) (157) ring B-prop: 2-CH2-TWIST
					(7.18%) (161) ring B-prop: C-O-H BEND
112	1147.23	-------	138.52	0.02	(11.14%) (68) ring C-prop: C-O stretch (27,25)
					(5.16%) (121) ring A-methyl: ROCKa
					(9.16%) (125) ring A-ethyl: CH2 ROCK
					(5.14%) (199) ring C-prop: C-O-H BEND
113	1139.57	-------	25.78	0.27	(14.75%) (77) ring D-methyl: C-C stretch (34,31)
					(12.12%) (217) ring D-ethyl: CH2 ROCK
					(9.80%) (224) ring D-ethyl: CH3 ROCKb
114	1137.16	-------	53.04	1.11	(17.14%) (24) ring B: C-N stretch (70,65)
					(6.74%) (30) ring B-prop: C-C stretch (75,69)
					(7.01%) (133) ring B: N-H ROCK
115	1126.71	-------	68.07	0.28	(6.18%) (8) ring A: C-C stretch (53,51)
					(7.18%) (104) ring A: N-H ROCK
					(6.71%) (184) ring C-methyl: ROCKa
116	1125.82	-------	125.59	0.02	(6.05%) (104) ring A: N-H ROCK
					(13.33%) (184) ring C-methyl: ROCKa
117	1116.77	-------	78.33	0.18	(13.23%) (81) ring D-ethyl: C-C stretch (38,32)
					(13.55%) (213) ring D-methyl: ROCKa
					(11.57%) (214) ring D-methyl: ROCKb
					(12.20%) (217) ring D-ethyl: CH2 ROCK
					(11.41%) (224) ring D-ethyl: CH3 ROCKb
118	1097.62	-------	60.43	0.53	(5.22%) (13) ring A-ethyl: C-C stretch (58,51)
					(5.44%) (26) ring B-methyl: C-C stretch (71,68)
					(5.21%) (115) ring A-ethyl: TWIST
					(5.15%) (123) ring A-ethyl: BEND
					(12.55%) (132) ring A-ethyl: CH3 ROCKb
119	1092.86	-------	39.23	1.78	(6.13%) (5) ring A: C-C stretch (51,49)
					(5.27%) (9) ring A-methyl: C-C stretch (54,49)
					(6.72%) (13) ring A-ethyl: C-C stretch (58,51)
					(6.25%) (104) ring A: N-H ROCK
					(6.55%) (111) ring A-methyl: TWIST
					(5.67%) (122) ring A-methyl: ROCKb
120	1085.81	-------	16.28	1.24	(6.35%) (30) ring B-prop: C-C stretch (75,69)
					(6.52%) (122) ring A-methyl: ROCKb
					(9.89%) (133) ring B: N-H ROCK
					(7.40%) (147) ring B-methyl: ROCKb
					(6.80%) (184) ring C-methyl: ROCKa
121	1076.61	-------	4.59	1.88	(9.95%) (9) ring A-methyl: C-C stretch (54,49)
					(11.88%) (122) ring A-methyl: ROCKb
					(13.47%) (132) ring A-ethyl: CH3 ROCKb
122	1064.02	-------	59.73	1.13	(13.39%) (84) ring D-ethyl: C-C stretch (41,38)
					(12.57%) (213) ring D-methyl: ROCKa
					(9.30%) (215) ring D-ethyl: BEND
					(44.19%) (223) ring D-ethyl: CH3 ROCKa
123	1060.09	-------	0.94	5.78	(21.97%) (133) ring B: N-H ROCK
					(6.08%) (162) bridge BC: BEND
					(13.67%) (171) ring C: N-H ROCK
124	1053.08	-------	4.38	0.67	(7.01%) (178) ring C-methyl: WAGG
					(7.26%) (183) ring C-methyl: ADEFb
					(76.54%) (185) ring C-methyl: ROCKb
125	1046.84	-------	3.26	0.07	(8.51%) (138) ring B-methyl: WAGG
					(5.52%) (144) ring B-methyl: ADEFa
					(64.13%) (146) ring B-methyl: ROCKa
					(12.52%) (147) ring B-methyl: ROCKb
126	1045.46	-------	34.53	0.82	(11.54%) (147) ring B-methyl: ROCKb
					(10.43%) (150) ring B-prop: 1-CH2-ROCK
					(6.93%) (155) ring B-prop: 2-CH2-ROCK
					(10.19%) (188) ring C-prop: 1-CH2-ROCK
					(5.46%) (193) ring C-prop: 2-CH2-ROCK
127	1040.14	-------	0.73	0.45	(7.41%) (205) ring D-methyl: WAGG
					(22.01%) (213) ring D-methyl: ROCKa
					(51.84%) (214) ring D-methyl: ROCKb
					(6.95%) (223) ring D-ethyl: CH3 ROCKa

128	1026.43	-------	2.96	0.07	(7.93%)	(13)	ring A-ethyl: C-C stretch (58,51)
					(10.76%)	(15)	ring A-ethyl: C-C stretch (60,58)
					(8.16%)	(109)	ring A-methyl: ROCK
					(27.40%)	(121)	ring A-methyl: ROCKa
					(6.10%)	(125)	ring A-ethyl: CH2 ROCK
					(8.24%)	(127)	ring A-ethyl: CH2 TWIST
129	1024.13	-------	0.42	0.07	(5.39%)	(5)	ring A: C-C stretch (51,49)
					(6.70%)	(9)	ring A-methyl: C-C stretch (54,49)
					(11.62%)	(13)	ring A-ethyl: C-C stretch (58,51)
					(25.42%)	(15)	ring A-ethyl: C-C stretch (60,58)
					(6.18%)	(112)	ring A-ethyl: SCIS
					(14.98%)	(122)	ring A-methyl: ROCKb
					(8.68%)	(131)	ring A-ethyl: CH3 ROCKa
130	1019.41	-------	46.81	0.70	(6.29%)	(150)	ring B-prop: 1-CH2-ROCK
					(6.04%)	(155)	ring B-prop: 2-CH2-ROCK
					(26.89%)	(171)	ring C: N-H ROCK
					(7.83%)	(193)	ring C-prop: 2-CH2-ROCK
131	999.94	-------	9.24	1.92	(9.45%)	(71)	ring D: C-N stretch (30,28)
					(12.34%)	(76)	ring D: C-C stretch (33,32)
					(10.75%)	(77)	ring D-methyl: C-C stretch (34,31)
					(22.09%)	(200)	ring D: N-H ROCK
132	998.43	-------	90.96	0.14	(18.44%)	(9)	ring A-methyl: C-C stretch (54,49)
					(13.37%)	(15)	ring A-ethyl: C-C stretch (60,58)
					(5.54%)	(62)	ring C-prop: C-C stretch (22,19)
					(7.17%)	(104)	ring A: N-H ROCK
					(5.58%)	(122)	ring A-methyl: ROCKb
133	997.62	-------	106.81	1.02	(36.19%)	(62)	ring C-prop: C-C stretch (22,19)
					(9.61%)	(184)	ring C-methyl: ROCKa
134	995.38	-------	18.31	0.12	(67.20%)	(33)	ring B-prop: C-C stretch (78,75)
					(6.06%)	(148)	ring B-prop: 1-BEND
					(5.43%)	(153)	ring B-prop: 2-BEND
					(5.14%)	(160)	ring B-prop: C=O OUT
135	984.42	-------	67.41	1.17	(5.14%)	(72)	ring D: C-C stretch (31,30)
					(7.43%)	(73)	ring D: C=C stretch (32,31)
					(5.31%)	(81)	ring D-ethyl: C-C stretch (38,32)
					(20.88%)	(84)	ring D-ethyl: C-C stretch (41,38)
					(5.26%)	(200)	ring D: N-H ROCK
					(16.78%)	(213)	ring D-methyl: ROCKa
					(7.80%)	(214)	ring D-methyl: ROCKb
					(6.31%)	(224)	ring D-ethyl: CH3 ROCKb
136	952.35	-------	138.84	0.06	(5.65%)	(22)	ring B: C-C stretch (68,67)
					(7.54%)	(23)	ring B: C-.C stretch (69,68)
					(14.84%)	(147)	ring B-methyl: ROCKb
					(9.63%)	(150)	ring B-prop: 1-CH2-ROCK
					(8.52%)	(155)	ring B-prop: 2-CH2-ROCK
					(5.12%)	(188)	ring C-prop: 1-CH2-ROCK
137	947.74	-------	48.39	0.57	(6.38%)	(186)	ring C-prop: 1-BEND
					(12.81%)	(188)	ring C-prop: 1-CH2-ROCK
					(7.26%)	(191)	ring C-prop: 2-BEND
					(20.91%)	(193)	ring C-prop: 2-CH2-ROCK
					(12.50%)	(198)	ring C-prop: C=O OUT
138	925.82	-------	3.59	1.40	(5.01%)	(62)	ring C-prop: C-C stretch (22,19)
					(18.04%)	(84)	ring D-ethyl: C-C stretch (41,38)
					(7.83%)	(193)	ring C-prop: 2-CH2-ROCK
139	924.16	-------	39.87	0.38	(7.09%)	(5)	ring A: C-C stretch (51,49)
					(10.84%)	(15)	ring A-ethyl: C-C stretch (60,58)
					(22.82%)	(84)	ring D-ethyl: C-C stretch (41,38)
					(10.07%)	(132)	ring A-ethyl: CH3 ROCKb
					(6.55%)	(223)	ring D-ethyl: CH3 ROCKa
140	918.52	-------	334.01	0.27	(5.67%)	(65)	ring C-prop: C-C stretch (25,22)
					(9.17%)	(84)	ring D-ethyl: C-C stretch (41,38)
					(6.56%)	(188)	ring C-prop: 1-CH2-ROCK
					(7.32%)	(193)	ring C-prop: 2-CH2-ROCK
141	908.52	-------	41.36	0.44	(21.76%)	(36)	ring B-prop: C-C stretch (81,78)
					(6.80%)	(38)	ring B-prop: C-O stretch (83,81)
					(5.51%)	(148)	ring B-prop: 1-BEND
					(6.73%)	(153)	ring B-prop: 2-BEND
					(12.08%)	(155)	ring B-prop: 2-CH2-ROCK
					(9.89%)	(160)	ring B-prop: C=O OUT
142	885.84	-------	13.64	0.37	(51.50%)	(164)	bridge BC: C-H OUT
					(9.23%)	(233)	bridge BC: 1-TORS
					(6.11%)	(236)	bridge BC: 2-TORS
143	884.83	-------	24.43	0.65	(5.55%)	(5)	ring A: C-C stretch (51,49)
					(6.07%)	(7)	ring A: C-N stretch (53,46)
					(12.43%)	(8)	ring A: C-C stretch (53,51)
					(5.95%)	(13)	ring A-ethyl: C-C stretch (58,51)
					(6.14%)	(15)	ring A-ethyl: C-C stretch (60,58)

					(5.30%)	(104)	ring A: N-H ROCK
					(17.06%)	(164)	bridge BC: C-H OUT
144	850.81	-------	17.39	0.19	(5.97%)	(36)	ring B-prop: C-C stretch (81,78)
					(6.18%)	(104)	ring A: N-H ROCK
					(9.36%)	(133)	ring B: N-H ROCK
145	849.66	-------	47.70	0.13	(5.14%)	(8)	ring A: C-C stretch (53,51)
					(5.73%)	(113)	ring A-ethyl: ROCK
					(16.15%)	(131)	ring A-ethyl: CH3 ROCKa
146	838.75	-------	37.20	4.29	(7.92%)	(54)	ring C: C-C stretch (14,13)
					(11.89%)	(171)	ring C: N-H ROCK
					(6.21%)	(200)	ring D: N-H ROCK
147	818.62	-------	28.63	43.29	(56.96%)	(167)	bridge CD: C-H OUT
					(9.09%)	(203)	ring D: bridge CD WAGG
					(10.80%)	(243)	bridge C=D: TORS
148	815.07	-------	18.75	5.83	(9.96%)	(72)	ring D: C-C stretch (31,30)
					(10.58%)	(217)	ring D-ethyl: CH2 ROCK
					(14.75%)	(224)	ring D-ethyl: CH3 ROCKb
149	801.20	-------	9.43	0.53	(8.11%)	(36)	ring B-prop: C-C stretch (81,78)
					(8.46%)	(65)	ring C-prop: C-C stretch (25,22)
					(8.11%)	(150)	ring B-prop: 1-CH2-ROCK
					(6.51%)	(155)	ring B-prop: 2-CH2-ROCK
150	795.29	-------	0.05	0.30	(6.55%)	(2)	ring A: C-N stretch (48,46)
					(21.60%)	(3)	ring A: C-C stretch (49,48)
					(8.90%)	(107)	ring A: C=O OUT
					(6.73%)	(121)	ring A-methyl: ROCKa
					(5.90%)	(122)	ring A-methyl: ROCKb
					(5.85%)	(125)	ring A-ethyl: CH2 ROCK
151	792.21	-------	17.07	0.05	(61.56%)	(170)	bridge AB: C-H OUT
					(13.94%)	(234)	bridge A=B: TORS
					(7.76%)	(235)	bridge A-B: TORS
152	787.22	-------	5.56	0.32	(22.76%)	(65)	ring C-prop: C-C stretch (25,22)
					(9.87%)	(68)	ring C-prop: C-O stretch (27,25)
					(6.62%)	(150)	ring B-prop: 1-CH2-ROCK
					(10.69%)	(188)	ring C-prop: 1-CH2-ROCK
153	776.19	-------	22.85	0.16	(5.11%)	(96)	ring C bending
					(6.43%)	(102)	ring D torsion
					(7.25%)	(165)	bridge CD: BEND
					(6.17%)	(207)	ring D-ethyl: WAGG
					(24.24%)	(209)	ring D: C=O OUT
154	769.55	-------	10.22	0.64	(5.04%)	(102)	ring D torsion
					(7.09%)	(207)	ring D-ethyl: WAGG
					(27.00%)	(209)	ring D: C=O OUT
155	762.99	-------	24.76	0.79	(5.56%)	(96)	ring C bending
					(15.87%)	(125)	ring A-ethyl: CH2 ROCK
					(5.54%)	(168)	bridge AB: BEND
156	755.05	-------	9.23	0.40	(21.09%)	(125)	ring A-ethyl: CH2 ROCK
					(5.26%)	(132)	ring A-ethyl: CH3 ROCKb
157	750.91	-------	10.96	0.46	(6.77%)	(76)	ring D: C-C stretch (33,32)
					(9.41%)	(100)	ring D bending
					(5.60%)	(209)	ring D: C=O OUT
					(26.29%)	(217)	ring D-ethyl: CH2 ROCK
					(12.53%)	(224)	ring D-ethyl: CH3 ROCKb
158	733.72	-------	9.90	0.36	(5.07%)	(88)	ring A bending
					(5.89%)	(217)	ring D-ethyl: CH2 ROCK
159	730.40	-------	13.05	0.43	(13.90%)	(94)	ring B torsion
					(13.73%)	(140)	ring B-prop: 1-WAGG
					(9.52%)	(142)	ring B: bridge BC WAGG
					(6.10%)	(148)	ring B-prop: 1-BEND
					(7.31%)	(160)	ring B-prop: C=O OUT
					(7.44%)	(232)	ring B-prop: 4-TORS
160	717.97	-------	16.99	1.99	(8.22%)	(98)	ring C torsion
					(5.04%)	(99)	ring C torsion
					(7.71%)	(174)	ring C: bridge BC WAGG
					(8.50%)	(176)	ring C-prop: 1-WAGG
					(5.22%)	(198)	ring C-prop: C=O OUT
161	715.13	-------	26.78	8.94	(5.21%)	(75)	ring D: C-N stretch (33,28)
					(9.72%)	(96)	ring C bending
					(24.86%)	(100)	ring D bending
162	705.54	-------	5.77	0.27	(13.29%)	(95)	ring B torsion
					(6.41%)	(107)	ring A: C=O OUT
					(25.53%)	(136)	ring B: bridge AB WAGG
					(10.02%)	(232)	ring B-prop: 4-TORS
					(6.31%)	(234)	bridge A=B: TORS

163	697.31	-------	115.68	0.22	(5.23%) (92) ring B bending
					(5.87%) (150) ring B-prop: 1-CH2-ROCK
					(9.84%) (155) ring B-prop: 2-CH2-ROCK
					(6.92%) (160) ring B-prop: C=O OUT
					(35.46%) (232) ring B-prop: 4-TORS
164	689.57	-------	56.44	0.73	(8.41%) (2) ring A: C-N stretch (48,46)
					(5.91%) (9) ring A-methyl: C-C stretch (54,49)
					(9.90%) (92) ring B bending
					(19.75%) (107) ring A: C=O OUT
165	684.35	-------	43.64	2.14	(9.21%) (98) ring C torsion
					(9.62%) (198) ring C-prop: C=O OUT
					(41.14%) (242) ring C-prop: 4-TORS
166	678.25	-------	30.57	0.75	(6.20%) (55) ring C-methyl: C-C stretch (15,13)
					(6.96%) (99) ring C torsion
					(8.17%) (107) ring A: C=O OUT
					(5.64%) (180) ring C: bridge CD WAGG
					(10.03%) (242) ring C-prop: 4-TORS
167	671.42	-------	33.25	0.20	(7.34%) (95) ring B torsion
					(5.29%) (107) ring A: C=O OUT
					(5.69%) (142) ring B: bridge BC WAGG
					(5.64%) (232) ring B-prop: 4-TORS
					(8.18%) (242) ring C-prop: 4-TORS
168	667.77	-------	8.38	1.27	(11.77%) (55) ring C-methyl: C-C stretch (15,13)
					(14.70%) (99) ring C torsion
					(8.19%) (100) ring D bending
					(6.54%) (174) ring C: bridge BC WAGG
169	659.30	-------	6.52	0.27	(5.27%) (8) ring A: C-C stretch (53,51)
					(10.13%) (88) ring A bending
					(29.74%) (117) ring A: bridge AB WAGG
170	651.54	-------	33.15	24.20	(7.07%) (102) ring D torsion
					(15.16%) (103) ring D torsion
					(24.36%) (203) ring D: bridge CD WAGG
					(13.56%) (205) ring D-methyl: WAGG
					(10.83%) (209) ring D: C=O OUT
					(5.58%) (243) bridge C=D: TORS
171	644.87	-------	2.84	4.73	(5.98%) (72) ring D: C-C stretch (31,30)
					(6.08%) (75) ring D: C-N stretch (33,28)
					(5.19%) (76) ring D: C-C stretch (33,32)
					(9.50%) (81) ring D-ethyl: C-C stretch (38,32)
					(5.13%) (99) ring C torsion
					(8.19%) (203) ring D: bridge CD WAGG
					(7.10%) (208) ring D: C=O ROCK
172	629.41	-------	24.09	0.50	(10.67%) (26) ring B-methyl: C-C stretch (71,68)
					(8.32%) (93) ring B bending
					(18.38%) (159) ring B-prop: C=O ROCK
					(6.54%) (232) ring B-prop: 4-TORS
173	618.06	-------	48.09	0.26	(9.41%) (159) ring B-prop: C=O ROCK
					(19.68%) (197) ring C-prop: C=O ROCK
					(8.48%) (242) ring C-prop: 4-TORS
174	609.78	-------	17.74	0.19	(10.31%) (88) ring A bending
					(31.74%) (89) ring A bending
					(11.68%) (117) ring A: bridge AB WAGG
175	604.16	-------	12.69	0.95	(7.40%) (93) ring B bending
					(6.72%) (159) ring B-prop: C=O ROCK
					(21.10%) (197) ring C-prop: C=O ROCK
176	593.51	-------	17.26	0.16	(7.84%) (26) ring B-methyl: C-C stretch (71,68)
					(7.80%) (55) ring C-methyl: C-C stretch (15,13)
					(5.56%) (59) ring C-prop: C-C stretch (19,12)
					(13.64%) (93) ring B bending
					(17.45%) (97) ring C bending
177	567.67	-------	5.45	0.09	(7.56%) (77) ring D-methyl: C-C stretch (34,31)
					(6.51%) (101) ring D bending
					(5.24%) (105) ring A: N-H OUT
178	565.56	-------	88.44	0.04	(45.59%) (105) ring A: N-H OUT
					(15.02%) (134) ring B: N-H OUT
					(9.08%) (172) ring C: N-H OUT
179	559.75	-------	15.90	0.21	(5.29%) (77) ring D-methyl: C-C stretch (34,31)
					(7.79%) (101) ring D bending
					(6.01%) (148) ring B-prop: 1-BEND
					(5.03%) (158) ring B-prop: 3-BEND
					(7.54%) (159) ring B-prop: C=O ROCK
					(13.14%) (160) ring B-prop: C=O OUT
					(10.07%) (232) ring B-prop: 4-TORS
180	549.29	-------	7.71	1.08	(18.93%) (101) ring D bending
					(5.93%) (179) ring C: bridge CD ROCK
					(9.52%) (208) ring D: C=O ROCK

181	531.16	-------	23.43	1.51	(6.85%)	(105) ring A: N-H OUT
					(11.31%)	(106) ring A: C=O ROCK
					(18.19%)	(134) ring B: N-H OUT
					(25.56%)	(172) ring C: N-H OUT
182	520.86	-------	17.02	0.36	(5.20%)	(134) ring B: N-H OUT
					(5.24%)	(174) ring C: bridge BC WAGG
					(8.06%)	(196) ring C-prop: 3-BEND
183	512.97	-------	9.96	1.66	(14.84%)	(105) ring A: N-H OUT
					(18.04%)	(172) ring C: N-H OUT
					(5.43%)	(193) ring C-prop: 2-CH2-ROCK
					(11.63%)	(198) ring C-prop: C=O OUT
					(5.03%)	(242) ring C-prop: 4-TORS
184	504.10	-------	25.28	0.02	(8.52%)	(105) ring A: N-H OUT
					(7.60%)	(106) ring A: C=O ROCK
					(7.83%)	(172) ring C: N-H OUT
					(5.58%)	(193) ring C-prop: 2-CH2-ROCK
					(7.17%)	(198) ring C-prop: C=O OUT
185	501.11	-------	6.97	1.01	(59.33%)	(134) ring B: N-H OUT
					(35.49%)	(172) ring C: N-H OUT
186	497.06	-------	12.28	4.04	(9.90%)	(106) ring A: C=O ROCK
					(6.41%)	(137) ring B-methyl: ROCK
					(7.05%)	(196) ring C-prop: 3-BEND
187	492.60	-------	6.20	7.36	(7.53%)	(101) ring D bending
					(5.73%)	(203) ring D: bridge CD WAGG
					(5.23%)	(205) ring D-methyl: WAGG
					(15.13%)	(207) ring D-ethyl: WAGG
					(6.51%)	(208) ring D: C=O ROCK
					(8.16%)	(209) ring D: C=O OUT
					(7.18%)	(215) ring D-ethyl: BEND
188	488.32	-------	12.16	1.01	(5.82%)	(137) ring B-methyl: ROCK
					(8.06%)	(196) ring C-prop: 3-BEND
					(9.28%)	(198) ring C-prop: C=O OUT
189	479.42	-------	7.12	1.90	(5.90%)	(142) ring B: bridge BC WAGG
					(5.45%)	(196) ring C-prop: 3-BEND
190	438.83	-------	2.33	0.18	(6.63%)	(5) ring A: C-C stretch (51,49)
					(5.37%)	(9) ring A-methyl: C-C stretch (54,49)
					(5.34%)	(13) ring A-ethyl: C-C stretch (58,51)
					(9.80%)	(109) ring A-methyl: ROCK
					(9.98%)	(113) ring A-ethyl: ROCK
					(5.70%)	(123) ring A-ethyl: BEND
191	431.04	-------	11.85	0.34	(6.83%)	(155) ring B-prop: 2-CH2-ROCK
					(28.94%)	(158) ring B-prop: 3-BEND
					(5.08%)	(174) ring C: bridge BC WAGG
					(6.12%)	(196) ring C-prop: 3-BEND
192	403.80	-------	6.08	0.96	(5.89%)	(136) ring B: bridge AB WAGG
					(7.36%)	(138) ring B-methyl: WAGG
					(13.57%)	(158) ring B-prop: 3-BEND
					(6.88%)	(174) ring C: bridge BC WAGG
					(6.10%)	(180) ring C: bridge CD WAGG
					(5.65%)	(196) ring C-prop: 3-BEND
193	398.56	-------	7.78	0.06	(5.57%)	(88) ring A bending
					(5.02%)	(89) ring A bending
					(8.99%)	(106) ring A: C=O ROCK
					(19.11%)	(123) ring A-ethyl: BEND
194	363.81	-------	9.58	0.68	(8.64%)	(103) ring D torsion
					(8.33%)	(179) ring C: bridge CD ROCK
					(10.90%)	(205) ring D-methyl: WAGG
					(8.48%)	(215) ring D-ethyl: BEND
195	350.20	-------	1.40	1.07	(6.33%)	(175) ring C-prop: 1-ROCK
					(27.95%)	(178) ring C-methyl: WAGG
					(9.27%)	(186) ring C-prop: 1-BEND
					(14.70%)	(196) ring C-prop: 3-BEND
					(5.35%)	(205) ring D-methyl: WAGG
196	343.65	-------	2.53	1.16	(5.16%)	(123) ring A-ethyl: BEND
					(12.39%)	(137) ring B-methyl: ROCK
					(5.33%)	(138) ring B-methyl: WAGG
					(20.71%)	(139) ring B-prop: 1-ROCK
					(5.79%)	(153) ring B-prop: 2-BEND
					(9.79%)	(201) ring D: N-H OUT
197	334.90	-------	35.98	0.62	(-16.35%)	(103) ring D torsion
					(75.17%)	(201) ring D: N-H OUT
198	325.27	-------	2.54	3.93	(8.02%)	(137) ring B-methyl: ROCK
					(28.31%)	(138) ring B-methyl: WAGG
					(7.15%)	(139) ring B-prop: 1-ROCK
					(5.20%)	(140) ring B-prop: 1-WAGG

					(11.08%) (158) ring B-prop: 3-BEND
199	319.06	-------	0.24	22.09	(10.36%) (175) ring C-prop: 1-ROCK
					(12.22%) (201) ring D: N-H OUT
					(9.18%) (203) ring D: bridge CD WAGG
					(18.81%) (205) ring D-methyl: WAGG
					(9.58%) (215) ring D-ethyl: BEND
200	317.12	-------	3.38	1.47	(5.11%) (76) ring D: C-C stretch (33,32)
					(43.91%) (204) ring D-methyl: ROCK
					(9.19%) (208) ring D: C=O ROCK
201	304.16	-------	3.13	6.01	(9.75%) (95) ring B torsion
					(21.93%) (177) ring C-methyl: ROCK
					(5.10%) (206) ring D-ethyl: ROCK
					(12.20%) (243) bridge C=D: TORS
					(6.40%) (246) ring D-ethyl: 2-TORS
202	293.94	-------	0.69	0.48	(14.56%) (178) ring C-methyl: WAGG
					(14.47%) (206) ring D-ethyl: ROCK
					(27.04%) (246) ring D-ethyl: 2-TORS
203	291.54	-------	2.37	9.03	(5.33%) (175) ring C-prop: 1-ROCK
					(6.13%) (177) ring C-methyl: ROCK
					(9.74%) (204) ring D-methyl: ROCK
204	287.14	-------	3.22	8.65	(5.58%) (99) ring C torsion
					(5.26%) (123) ring A-ethyl: BEND
					(5.22%) (178) ring C-methyl: WAGG
					(7.57%) (206) ring D-ethyl: ROCK
					(9.41%) (246) ring D-ethyl: 2-TORS
205	279.52	-------	2.08	4.10	(5.03%) (110) ring A-methyl: WAGG
					(5.79%) (111) ring A-methyl: TWIST
					(14.78%) (123) ring A-ethyl: BEND
					(5.30%) (137) ring B-methyl: ROCK
					(5.42%) (227) ring A-ethyl: 2-TORS
206	269.13	-------	0.61	0.15	(5.07%) (173) ring C: bridge BC ROCK
					(7.49%) (177) ring C-methyl: ROCK
					(22.31%) (227) ring A-ethyl: 2-TORS
207	255.84	-------	2.58	0.55	(9.16%) (109) ring A-methyl: ROCK
					(9.57%) (225) ring A-methyl: TORS
					(23.64%) (227) ring A-ethyl: 2-TORS
208	246.93	-------	1.65	0.32	(6.08%) (110) ring A-methyl: WAGG
					(7.64%) (137) ring B-methyl: ROCK
					(5.81%) (138) ring B-methyl: WAGG
					(24.95%) (225) ring A-methyl: TORS
209	237.27	-------	0.47	0.16	(11.85%) (111) ring A-methyl: TWIST
					(57.43%) (225) ring A-methyl: TORS
210	232.83	-------	2.07	0.54	(6.81%) (202) ring D: bridge CD ROCK
					(10.92%) (227) ring A-ethyl: 2-TORS
					(6.50%) (234) bridge A=B: TORS
211	224.47	-------	2.29	0.77	(6.63%) (135) ring B: bridge AB ROCK
					(5.28%) (227) ring A-ethyl: 2-TORS
					(40.06%) (238) ring C-methyl: TORS
					(5.18%) (244) ring D-methyl: TORS
212	219.72	-------	1.04	0.83	(33.29%) (238) ring C-methyl: TORS
					(7.76%) (244) ring D-methyl: TORS
					(8.93%) (246) ring D-ethyl: 2-TORS
213	215.79	-------	1.19	0.55	(9.39%) (135) ring B: bridge AB ROCK
					(10.14%) (137) ring B-methyl: ROCK
					(10.62%) (153) ring B-prop: 2-BEND
					(5.44%) (206) ring D-ethyl: ROCK
					(12.04%) (246) ring D-ethyl: 2-TORS
214	209.16	-------	2.55	0.64	(6.36%) (176) ring C-prop: 1-WAGG
					(9.75%) (206) ring D-ethyl: ROCK
					(13.98%) (246) ring D-ethyl: 2-TORS
215	196.27	-------	4.41	2.02	(7.47%) (91) ring A torsion
					(7.16%) (141) ring B: bridge BC ROCK
					(6.35%) (235) bridge A-B: TORS
					(5.62%) (246) ring D-ethyl: 2-TORS
					(11.37%) (248) Cl-H stretch
216	187.90	-------	1.69	2.59	(5.03%) (113) ring A-ethyl: ROCK
					(8.37%) (153) ring B-prop: 2-BEND
					(6.11%) (186) ring C-prop: 1-BEND
					(6.08%) (227) ring A-ethyl: 2-TORS
217	184.11	-------	2.53	2.10	(8.59%) (102) ring D torsion
					(7.26%) (205) ring D-methyl: WAGG
					(6.28%) (207) ring D-ethyl: WAGG
					(8.17%) (215) ring D-ethyl: BEND
					(6.67%) (237) bridge C-D: TORS

					(17.48%)	(248)	Cl-H stretch
218	179.18	-------	3.58	0.25	(11.60%)	(91)	ring A torsion
					(5.48%)	(123)	ring A-ethyl: BEND
					(5.20%)	(247)	Cl-H stretch
219	168.25	-------	3.92	0.29	(8.38%)	(91)	ring A torsion
					(6.45%)	(95)	ring B torsion
					(5.29%)	(109)	ring A-methyl: ROCK
					(6.98%)	(162)	bridge BC: BEND
					(13.83%)	(235)	bridge A-B: TORS
					(5.01%)	(247)	Cl-H stretch
					(17.49%)	(248)	Cl-H stretch
220	166.53	-------	5.89	1.55	(7.24%)	(91)	ring A torsion
					(5.37%)	(102)	ring D torsion
					(5.12%)	(205)	ring D-methyl: WAGG
					(6.79%)	(207)	ring D-ethyl: WAGG
					(7.30%)	(215)	ring D-ethyl: BEND
					(30.12%)	(248)	Cl-H stretch
221	164.33	-------	0.11	1.42	(6.22%)	(98)	ring C torsion
					(5.86%)	(202)	ring D: bridge CD ROCK
					(7.52%)	(207)	ring D-ethyl: WAGG
					(5.00%)	(215)	ring D-ethyl: BEND
					(15.27%)	(237)	bridge C-D: TORS
					(6.60%)	(238)	ring C-methyl: TORS
					(7.52%)	(243)	bridge C=D: TORS
					(9.27%)	(244)	ring D-methyl: TORS
222	150.93	-------	1.01	6.30	(8.44%)	(103)	ring D torsion
					(9.02%)	(153)	ring B-prop: 2-BEND
					(5.37%)	(237)	bridge C-D: TORS
					(12.88%)	(247)	Cl-H stretch
223	146.45	-------	5.27	0.48	(12.86%)	(91)	ring A torsion
					(5.09%)	(114)	ring A-ethyl: WAGG
					(6.98%)	(115)	ring A-ethyl: TWIST
					(-6.20%)	(235)	bridge A-B: TORS
					(31.77%)	(247)	Cl-H stretch
					(8.03%)	(249)	Cl OUT
224	140.53	-------	0.95	1.00	(6.66%)	(91)	ring A torsion
					(11.61%)	(103)	ring D torsion
					(5.94%)	(141)	ring B: bridge BC ROCK
					(22.63%)	(228)	ring B-methyl: TORS
225	137.52	-------	3.29	0.19	(13.55%)	(103)	ring D torsion
					(17.23%)	(228)	ring B-methyl: TORS
					(10.94%)	(244)	ring D-methyl: TORS
					(5.58%)	(247)	Cl-H stretch
226	131.58	-------	0.95	0.41	(5.36%)	(91)	ring A torsion
					(5.08%)	(226)	ring A-ethyl: 1-TORS
					(48.79%)	(228)	ring B-methyl: TORS
					(12.93%)	(244)	ring D-methyl: TORS
227	126.89	-------	0.54	0.27	(5.89%)	(116)	ring A: bridge AB ROCK
					(5.45%)	(168)	bridge AB: BEND
					(6.43%)	(175)	ring C-prop: 1-ROCK
					(17.19%)	(226)	ring A-ethyl: 1-TORS
					(6.11%)	(247)	Cl-H stretch
228	121.87	-------	0.81	4.42	(7.61%)	(174)	ring C: bridge BC WAGG
					(36.52%)	(244)	ring D-methyl: TORS
229	104.34	-------	1.06	1.29	(5.36%)	(102)	ring D torsion
					(6.70%)	(173)	ring C: bridge BC ROCK
					(30.89%)	(226)	ring A-ethyl: 1-TORS
230	101.51	-------	0.29	5.95	(5.51%)	(98)	ring C torsion
					(22.37%)	(102)	ring D torsion
					(9.79%)	(207)	ring D-ethyl: WAGG
					(14.25%)	(226)	ring A-ethyl: 1-TORS
					(6.11%)	(237)	bridge C-D: TORS
231	90.31	-------	0.87	0.66	(17.17%)	(94)	ring B torsion
					(6.18%)	(117)	ring A: bridge AB WAGG
					(9.29%)	(140)	ring B-prop: 1-WAGG
					(6.75%)	(148)	ring B-prop: 1-BEND
					(7.34%)	(229)	ring B-prop: 1-TORS
					(5.74%)	(233)	bridge BC: 1-TORS
					(5.16%)	(247)	Cl-H stretch
					(5.02%)	(249)	Cl OUT
232	79.96	-------	1.09	0.96	(5.29%)	(103)	ring D torsion
					(13.18%)	(116)	ring A: bridge AB ROCK
					(5.51%)	(135)	ring B: bridge AB ROCK
					(14.91%)	(168)	bridge AB: BEND
					(11.32%)	(226)	ring A-ethyl: 1-TORS
					(5.23%)	(235)	bridge A-B: TORS
					(6.91%)	(239)	ring C-prop: 1-TORS
					(6.77%)	(240)	ring C-prop: 2-TORS

					(13.20%)	(249)	Cl OUT
233	76.15	-------	0.44	1.03	(5.30%)	(103)	ring D torsion
					(6.47%)	(142)	ring B: bridge BC WAGG
					(6.33%)	(233)	bridge BC: 1-TORS
					(21.58%)	(239)	ring C-prop: 1-TORS
234	68.82	-------	2.76	1.31	(13.16%)	(229)	ring B-prop: 1-TORS
					(8.66%)	(234)	bridge A=B: TORS
					(9.31%)	(236)	bridge BC: 2-TORS
					(6.62%)	(237)	bridge C-D: TORS
					(12.41%)	(249)	Cl OUT
235	61.26	-------	0.82	0.27	(5.71%)	(102)	ring D torsion
					(5.50%)	(229)	ring B-prop: 1-TORS
					(6.04%)	(236)	bridge BC: 2-TORS
					(16.61%)	(240)	ring C-prop: 2-TORS
					(10.30%)	(249)	Cl OUT
236	58.86	-------	0.02	2.69	(6.40%)	(162)	bridge BC: BEND
					(10.75%)	(165)	bridge CD: BEND
					(13.69%)	(229)	ring B-prop: 1-TORS
					(11.58%)	(231)	ring B-prop: 3-TORS
					(5.39%)	(245)	ring D-ethyl: 1-TORS
237	45.78	-------	4.07	0.46	(9.14%)	(229)	ring B-prop: 1-TORS
					(34.46%)	(230)	ring B-prop: 2-TORS
					(12.78%)	(231)	ring B-prop: 3-TORS
					(11.70%)	(241)	ring C-prop: 3-TORS
238	44.29	-------	0.64	0.28	(5.23%)	(98)	ring C torsion
					(6.54%)	(180)	ring C: bridge CD WAGG
					(8.67%)	(230)	ring B-prop: 2-TORS
					(35.20%)	(231)	ring B-prop: 3-TORS
					(14.33%)	(241)	ring C-prop: 3-TORS
239	41.32	-------	0.26	1.83	(5.37%)	(241)	ring C-prop: 3-TORS
					(5.50%)	(243)	bridge C=D: TORS
					(56.91%)	(245)	ring D-ethyl: 1-TORS
240	36.10	-------	0.91	0.71	(40.69%)	(90)	ring A torsion
					(7.24%)	(234)	bridge A=B: TORS
					(11.41%)	(235)	bridge A-B: TORS
					(5.15%)	(241)	ring C-prop: 3-TORS
					(10.09%)	(245)	ring D-ethyl: 1-TORS
241	29.83	-------	1.26	3.32	(11.87%)	(90)	ring A torsion
					(5.33%)	(230)	ring B-prop: 2-TORS
					(5.12%)	(231)	ring B-prop: 3-TORS
					(5.18%)	(233)	bridge BC: 1-TORS
					(9.90%)	(237)	bridge C-D: TORS
					(26.97%)	(241)	ring C-prop: 3-TORS
					(9.66%)	(249)	Cl OUT
242	28.58	-------	0.14	4.31	(8.05%)	(90)	ring A torsion
					(5.56%)	(94)	ring B torsion
					(10.15%)	(136)	ring B: bridge AB WAGG
					(7.40%)	(140)	ring B-prop: 1-WAGG
					(10.72%)	(229)	ring B-prop: 1-TORS
					(13.45%)	(234)	bridge A=B: TORS
					(7.78%)	(239)	ring C-prop: 1-TORS
					(5.55%)	(241)	ring C-prop: 3-TORS
243	26.78	-------	1.18	0.91	(6.28%)	(140)	ring B-prop: 1-WAGG
					(16.13%)	(229)	ring B-prop: 1-TORS
					(19.02%)	(230)	ring B-prop: 2-TORS
					(20.77%)	(239)	ring C-prop: 1-TORS
					(11.64%)	(240)	ring C-prop: 2-TORS
244	24.12	-------	0.26	1.90	(6.17%)	(229)	ring B-prop: 1-TORS
					(19.53%)	(230)	ring B-prop: 2-TORS
					(12.81%)	(231)	ring B-prop: 3-TORS
					(19.94%)	(240)	ring C-prop: 2-TORS
					(5.12%)	(243)	bridge C=D: TORS
					(6.49%)	(245)	ring D-ethyl: 1-TORS
245	22.00	-------	1.30	6.30	(5.55%)	(103)	ring D torsion
					(5.17%)	(167)	bridge CD: C-H OUT
					(6.15%)	(230)	ring B-prop: 2-TORS
					(14.56%)	(237)	bridge C-D: TORS
					(13.01%)	(239)	ring C-prop: 1-TORS
					(9.47%)	(241)	ring C-prop: 3-TORS
					(9.67%)	(249)	Cl OUT
246	19.65	-------	0.11	4.54	(8.09%)	(162)	bridge BC: BEND
					(11.42%)	(165)	bridge CD: BEND
					(9.83%)	(179)	ring C: bridge CD ROCK
					(5.85%)	(240)	ring C-prop: 2-TORS
					(9.37%)	(245)	ring D-ethyl: 1-TORS
247	15.35	-------	0.17	2.62	(21.15%)	(90)	ring A torsion
					(26.45%)	(235)	bridge A-B: TORS
					(8.55%)	(236)	bridge BC: 2-TORS

248	12.04	-------	0.92	30.17	(5.95%) (162) bridge BC: BEND<br>(12.07%) (174) ring C: bridge BC WAGG<br>(8.23%) (233) bridge BC: 1-TORS<br>(28.76%) (237) bridge C-D: TORS<br>(7.77%) (249) Cl OUT
249	10.61	-------	0.01	5.41	(5.26%) (90) ring A torsion<br>(5.05%) (136) ring B: bridge AB WAGG<br>(15.05%) (233) bridge BC: 1-TORS<br>(5.27%) (235) bridge A-B: TORS<br>(23.34%) (236) bridge BC: 2-TORS

ZZEssa, $^{13}C(15)$, H_2O

	calc. [cm-1]	exp. [cm-1]	IRint [km/mol]	Rint (rel.)	contribution
1	3522.77	-------	55.00	0.18	(100.07%) (67) ring C-prop: O-H stretch (27,84)
2	3516.59	-------	55.88	0.21	(100.07%) (39) ring B-prop: O-H stretch (85,83)
3	3489.04	-------	55.55	0.15	(99.72%) (69) ring D: N-H stretch (29,28)
4	3122.34	-------	774.59	0.12	(38.08%) (1) ring A: N-H stretch (47,46)<br>(33.26%) (20) ring B: N-H stretch (66,65)<br>(28.34%) (47) ring C: N-H stretch (10, 9)
5	3067.41	-------	0.99	0.21	(99.72%) (42) bridge BC: C-H stretch (4, 3)
6	3056.79	-------	28.77	0.35	(99.49%) (46) bridge AB: C-H stretch (8, 7)
7	3037.26	-------	524.94	0.55	(58.50%) (1) ring A: N-H stretch (47,46)<br>(9.27%) (20) ring B: N-H stretch (66,65)<br>(32.00%) (47) ring C: N-H stretch (10, 9)
8	3015.64	-------	160.94	0.11	(56.94%) (20) ring B: N-H stretch (66,65)<br>(38.68%) (47) ring C: N-H stretch (10, 9)
9	3013.43	-------	18.26	0.10	(49.29%) (56) ring C-methyl: C-H stretch (16,15)<br>(46.00%) (57) ring C-methyl: C-H stretch (17,15)
10	3012.89	-------	10.20	0.14	(79.60%) (78) ring D-methyl: C-H stretch (35,34)<br>(5.54%) (79) ring D-methyl: C-H stretch (36,34)<br>(12.78%) (80) ring D-methyl: C-H stretch (37,34)
11	3006.08	-------	19.05	0.09	(9.15%) (85) ring D-ethyl: C-H stretch (42,41)<br>(11.25%) (86) ring D-ethyl: C-H stretch (43,41)<br>(75.61%) (87) ring D-ethyl: C-H stretch (44,41)
12	3003.59	-------	10.42	0.11	(16.38%) (10) ring A-methyl: C-H stretch (55,54)<br>(6.13%) (11) ring A-methyl: C-H stretch (56,54)<br>(77.31%) (12) ring A-methyl: C-H stretch (57,54)
13	3002.73	-------	10.93	0.07	(99.05%) (43) bridge CD: C-H stretch (6, 5)
14	3000.91	-------	15.26	0.04	(6.78%) (60) ring C-prop: C-H stretch (20,19)<br>(71.56%) (61) ring C-prop: C-H stretch (21,19)<br>(19.65%) (63) ring C-prop: C-H stretch (23,22)
15	2998.99	-------	35.23	0.14	(13.35%) (27) ring B-methyl: C-H stretch (72,71)<br>(7.80%) (31) ring B-prop: C-H stretch (76,75)<br>(9.98%) (32) ring B-prop: C-H stretch (77,75)<br>(60.02%) (34) ring B-prop: C-H stretch (79,78)<br>(7.03%) (35) ring B-prop: C-H stretch (80,78)
16	2996.58	-------	11.74	0.13	(73.54%) (27) ring B-methyl: C-H stretch (72,71)<br>(5.57%) (29) ring B-methyl: C-H stretch (74,71)<br>(13.74%) (34) ring B-prop: C-H stretch (79,78)
17	2987.25	-------	27.14	0.21	(31.88%) (17) ring A-ethyl: C-H stretch (62,60)<br>(58.84%) (18) ring A-ethyl: C-H stretch (63,60)
18	2985.50	-------	50.80	0.51	(51.26%) (85) ring D-ethyl: C-H stretch (42,41)<br>(45.14%) (86) ring D-ethyl: C-H stretch (43,41)
19	2984.68	-------	0.97	0.07	(18.40%) (61) ring C-prop: C-H stretch (21,19)<br>(70.81%) (63) ring C-prop: C-H stretch (23,22)<br>(7.85%) (64) ring C-prop: C-H stretch (24,22)
20	2984.25	-------	46.15	0.20	(45.83%) (10) ring A-methyl: C-H stretch (55,54)<br>(43.17%) (11) ring A-methyl: C-H stretch (56,54)<br>(5.35%) (18) ring A-ethyl: C-H stretch (63,60)
21	2981.34	-------	8.46	0.07	(8.45%) (56) ring C-methyl: C-H stretch (16,15)<br>(10.06%) (57) ring C-methyl: C-H stretch (17,15)

					(8.87%) (58) ring C-methyl: C-H stretch (18,15)
					(36.10%) (79) ring D-methyl: C-H stretch (36,34)
					(35.98%) (80) ring D-methyl: C-H stretch (37,34)
22	2979.96	-------	8.80	0.16	(27.75%) (56) ring C-methyl: C-H stretch (16,15)
					(27.06%) (57) ring C-methyl: C-H stretch (17,15)
					(17.96%) (58) ring C-methyl: C-H stretch (18,15)
					(12.94%) (79) ring D-methyl: C-H stretch (36,34)
					(13.89%) (80) ring D-methyl: C-H stretch (37,34)
23	2979.83	-------	34.08	0.09	(5.95%) (14) ring A-ethyl: C-H stretch (59,58)
					(46.58%) (16) ring A-ethyl: C-H stretch (61,60)
					(28.82%) (17) ring A-ethyl: C-H stretch (62,60)
					(11.05%) (18) ring A-ethyl: C-H stretch (63,60)
					(6.24%) (40) ring A-ethyl: C-H stretch (1,58)
24	2977.85	-------	4.04	0.06	(46.43%) (31) ring B-prop: C-H stretch (76,75)
					(35.13%) (32) ring B-prop: C-H stretch (77,75)
					(16.50%) (34) ring B-prop: C-H stretch (79,78)
25	2956.12	-------	10.76	0.11	(56.79%) (82) ring D-ethyl: C-H stretch (39,38)
					(36.28%) (83) ring D-ethyl: C-H stretch (40,38)
26	2954.93	-------	14.59	0.19	(48.16%) (28) ring B-methyl: C-H stretch (73,71)
					(51.99%) (29) ring B-methyl: C-H stretch (74,71)
27	2949.87	-------	6.14	0.10	(52.31%) (14) ring A-ethyl: C-H stretch (59,58)
					(9.42%) (17) ring A-ethyl: C-H stretch (62,60)
					(28.61%) (40) ring A-ethyl: C-H stretch (1,58)
28	2947.12	-------	19.87	0.23	(43.73%) (31) ring B-prop: C-H stretch (76,75)
					(52.09%) (32) ring B-prop: C-H stretch (77,75)
29	2941.13	-------	8.02	0.25	(93.98%) (4) ring A: C-H stretch (50,49)
30	2936.43	-------	17.54	0.37	(75.58%) (60) ring C-prop: C-H stretch (20,19)
					(8.49%) (61) ring C-prop: C-H stretch (21,19)
					(13.79%) (64) ring C-prop: C-H stretch (24,22)
31	2930.97	-------	24.67	0.54	(17.31%) (78) ring D-methyl: C-H stretch (35,34)
					(43.16%) (79) ring D-methyl: C-H stretch (36,34)
					(36.55%) (80) ring D-methyl: C-H stretch (37,34)
32	2928.56	-------	2.64	0.17	(8.79%) (34) ring B-prop: C-H stretch (79,78)
					(87.42%) (35) ring B-prop: C-H stretch (80,78)
33	2925.03	-------	11.13	0.11	(14.88%) (60) ring C-prop: C-H stretch (20,19)
					(7.17%) (63) ring C-prop: C-H stretch (23,22)
					(76.99%) (64) ring C-prop: C-H stretch (24,22)
34	2922.01	-------	46.00	0.32	(38.42%) (85) ring D-ethyl: C-H stretch (42,41)
					(38.29%) (86) ring D-ethyl: C-H stretch (43,41)
					(19.07%) (87) ring D-ethyl: C-H stretch (44,41)
35	2921.78	-------	72.98	0.44	(29.54%) (6) ring A: C-H stretch (52,51)
					(15.85%) (10) ring A-methyl: C-H stretch (55,54)
					(23.80%) (11) ring A-methyl: C-H stretch (56,54)
					(10.89%) (12) ring A-methyl: C-H stretch (57,54)
					(9.76%) (14) ring A-ethyl: C-H stretch (59,58)
36	2919.15	-------	2.49	0.04	(16.34%) (6) ring A: C-H stretch (52,51)
					(15.21%) (10) ring A-methyl: C-H stretch (55,54)
					(21.31%) (11) ring A-methyl: C-H stretch (56,54)
					(8.74%) (12) ring A-methyl: C-H stretch (57,54)
					(15.12%) (14) ring A-ethyl: C-H stretch (59,58)
					(14.43%) (40) ring A-ethyl: C-H stretch (1,58)
37	2918.33	-------	21.76	0.28	(37.09%) (82) ring D-ethyl: C-H stretch (39,38)
					(57.06%) (83) ring D-ethyl: C-H stretch (40,38)
38	2917.20	-------	41.63	0.34	(11.68%) (56) ring C-methyl: C-H stretch (16,15)
					(13.90%) (57) ring C-methyl: C-H stretch (17,15)
					(70.90%) (58) ring C-methyl: C-H stretch (18,15)

39	2915.75	-------	27.18	0.13	(9.86%)	(6)	ring A: C-H stretch (52,51)
					(41.08%)	(16)	ring A-ethyl: C-H stretch (61,60)
					(26.18%)	(17)	ring A-ethyl: C-H stretch (62,60)
					(18.90%)	(18)	ring A-ethyl: C-H stretch (63,60)
40	2911.28	-------	6.29	0.24	(39.40%)	(6)	ring A: C-H stretch (52,51)
					(11.35%)	(14)	ring A-ethyl: C-H stretch (59,58)
					(44.71%)	(40)	ring A-ethyl: C-H stretch (1,58)
41	2909.71	-------	45.42	0.56	(12.34%)	(27)	ring B-methyl: C-H stretch (72,71)
					(45.86%)	(28)	ring B-methyl: C-H stretch (73,71)
					(41.10%)	(29)	ring B-methyl: C-H stretch (74,71)
42	1783.90	-------	291.90	1.67	(84.17%)	(19)	ring A: C=O stretch (64,48)
					(5.18%)	(88)	ring A bending
43	1774.15	-------	191.15	0.05	(81.29%)	(66)	ring C-prop: C=O stretch (26,25)
					(6.41%)	(199)	ring C-prop: C-O-H BEND
44	1760.70	-------	253.13	0.02	(81.10%)	(37)	ring B-prop: C=O stretch (82,81)
					(6.53%)	(161)	ring B-prop: C-O-H BEND
45	1742.86	-------	928.76	3.78	(79.67%)	(74)	ring D: C=O stretch (33,45)
46	1618.85	-------	498.63	31.18	(5.33%)	(7)	ring A: C-N stretch (53,46)
					(17.62%)	(41)	bridge BC: C-.C stretch (3,70)
					(23.73%)	(44)	bridge AB: C=C stretch (7,53)
					(13.07%)	(48)	bridge BC: C-.C stretch (11, 3)
					(8.74%)	(163)	bridge BC: C-H ROCK
47	1613.81	-------	8.52	19.84	(68.52%)	(73)	ring D: C=C stretch (32,31)
					(6.16%)	(77)	ring D-methyl: C-C stretch (34,31)
					(7.86%)	(81)	ring D-ethyl: C-C stretch (38,32)
48	1596.74	-------	154.47	100.00	(5.46%)	(52)	bridge CD: C-C stretch (14, 5)
					(56.32%)	(70)	bridge CD: C=C stretch (30, 5)
					(6.90%)	(71)	ring D: C-N stretch (30,28)
					(8.18%)	(166)	bridge CD: C-H ROCK
49	1592.42	-------	1294.67	2.41	(19.29%)	(23)	ring B: C-.C stretch (69,68)
					(7.44%)	(41)	bridge BC: C-.C stretch (3,70)
					(17.63%)	(44)	bridge AB: C=C stretch (7,53)
					(9.20%)	(48)	bridge BC: C-.C stretch (11, 3)
					(7.81%)	(163)	bridge BC: C-H ROCK
50	1556.90	-------	363.76	25.14	(8.40%)	(21)	ring B: C-N stretch (67,65)
					(7.29%)	(45)	bridge AB: C-C stretch (7,67)
					(5.31%)	(48)	bridge BC: C-.C stretch (11, 3)
					(27.50%)	(133)	ring B: N-H ROCK
					(21.11%)	(171)	ring C: N-H ROCK
51	1542.03	-------	1104.95	8.72	(27.33%)	(23)	ring B: C-.C stretch (69,68)
					(5.57%)	(26)	ring B-methyl: C-C stretch (71,68)
					(7.90%)	(41)	bridge BC: C-.C stretch (3,70)
					(8.41%)	(44)	bridge AB: C=C stretch (7,53)
					(5.62%)	(92)	ring B bending
52	1519.98	-------	71.67	11.08	(6.95%)	(7)	ring A: C-N stretch (53,46)
					(7.33%)	(45)	bridge AB: C-C stretch (7,67)
					(6.47%)	(54)	ring C: C-C stretch (14,13)
					(5.21%)	(104)	ring A: N-H ROCK
					(8.86%)	(133)	ring B: N-H ROCK
					(18.70%)	(171)	ring C: N-H ROCK
53	1506.49	-------	216.74	5.50	(36.86%)	(51)	ring C: C-.C stretch (13,12)
					(11.16%)	(53)	ring C: C-N stretch (14, 9)
					(8.00%)	(55)	ring C-methyl: C-C stretch (15,13)
54	1481.69	-------	15.12	0.20	(62.73%)	(182)	ring C-methyl: ADEFa
					(5.66%)	(184)	ring C-methyl: ROCKa
					(5.32%)	(211)	ring D-methyl: ADEFa
55	1477.18	-------	11.24	0.17	(6.22%)	(149)	ring B-prop: 1-CH2-SCIS
					(5.44%)	(216)	ring D-ethyl: CH2 SCIS

					(66.51%) (221) ring D-ethyl: CH3 ADEFa
					(9.60%) (222) ring D-ethyl: CH3 ADEFb
					(6.14%) (223) ring D-ethyl: CH3 ROCKa
56	1477.14	-------	54.80	0.16	(8.71%) (145) ring B-methyl: ADEFb
					(65.12%) (149) ring B-prop: 1-CH2-SCIS
					(6.53%) (221) ring D-ethyl: CH3 ADEFa
57	1473.57	-------	5.03	0.06	(14.27%) (124) ring A-ethyl: CH2 SCIS
					(44.32%) (129) ring A-ethyl: CH3 ADEFa
					(25.76%) (130) ring A-ethyl: CH3 ADEFb
58	1471.19	-------	4.11	5.50	(10.14%) (7) ring A: C-N stretch (53,46)
					(8.37%) (133) ring B: N-H ROCK
					(6.03%) (144) ring B-methyl: ADEFa
					(24.17%) (169) bridge AB: C-H ROCK
59	1468.65	-------	4.60	0.14	(20.91%) (119) ring A-methyl: ADEFa
					(38.96%) (129) ring A-ethyl: CH3 ADEFa
					(25.38%) (130) ring A-ethyl: CH3 ADEFb
60	1467.21	-------	11.58	0.23	(30.79%) (119) ring A-methyl: ADEFa
					(31.72%) (120) ring A-methyl: ADEFb
					(5.09%) (124) ring A-ethyl: CH2 SCIS
					(20.57%) (130) ring A-ethyl: CH3 ADEFb
61	1465.19	-------	3.09	0.07	(36.72%) (183) ring C-methyl: ADEFb
					(11.98%) (187) ring C-prop: 1-CH2-SCIS
					(28.25%) (212) ring D-methyl: ADEFb
62	1465.11	-------	2.80	0.17	(32.60%) (119) ring A-methyl: ADEFa
					(47.61%) (120) ring A-methyl: ADEFb
63	1463.92	-------	11.50	0.24	(6.62%) (221) ring D-ethyl: CH3 ADEFa
					(77.50%) (222) ring D-ethyl: CH3 ADEFb
					(7.15%) (224) ring D-ethyl: CH3 ROCKb
64	1459.06	-------	63.61	0.43	(5.11%) (21) ring B: C-N stretch (67,65)
					(14.27%) (144) ring B-methyl: ADEFa
					(25.65%) (145) ring B-methyl: ADEFb
					(7.33%) (149) ring B-prop: 1-CH2-SCIS
					(12.00%) (187) ring C-prop: 1-CH2-SCIS
					(7.08%) (212) ring D-methyl: ADEFb
65	1457.06	-------	8.62	0.39	(13.19%) (144) ring B-methyl: ADEFa
					(6.30%) (145) ring B-methyl: ADEFb
					(22.72%) (187) ring C-prop: 1-CH2-SCIS
					(28.46%) (212) ring D-methyl: ADEFb
66	1454.63	-------	58.02	1.38	(69.00%) (211) ring D-methyl: ADEFa
					(6.35%) (213) ring D-methyl: ROCKa
67	1454.19	-------	8.46	0.16	(72.78%) (124) ring A-ethyl: CH2 SCIS
					(14.31%) (130) ring A-ethyl: CH3 ADEFb
68	1453.21	-------	11.18	0.54	(44.51%) (144) ring B-methyl: ADEFa
					(18.50%) (145) ring B-methyl: ADEFb
					(13.25%) (183) ring C-methyl: ADEFb
69	1452.64	-------	13.14	0.35	(7.13%) (144) ring B-methyl: ADEFa
					(17.26%) (145) ring B-methyl: ADEFb
					(26.76%) (183) ring C-methyl: ADEFb
					(26.38%) (187) ring C-prop: 1-CH2-SCIS
					(6.02%) (212) ring D-methyl: ADEFb
70	1445.04	-------	2.00	0.41	(75.50%) (216) ring D-ethyl: CH2 SCIS
					(9.60%) (221) ring D-ethyl: CH3 ADEFa
71	1441.04	-------	13.05	9.53	(9.56%) (21) ring B: C-N stretch (67,65)
					(5.84%) (23) ring B: C-.C stretch (69,68)
					(5.44%) (41) bridge BC: C-.C stretch (3,70)
					(5.05%) (52) bridge CD: C-C stretch (14, 5)
					(10.09%) (169) bridge AB: C-H ROCK

72	1438.89	-------	23.69	0.34	(69.66%)	(154)	ring B-prop: 2-CH2-SCIS
					(7.52%)	(163)	bridge BC: C-H ROCK
73	1434.49	-------	14.72	1.76	(6.52%)	(49)	ring C: C-N stretch (11, 9)
					(6.46%)	(149)	ring B-prop: 1-CH2-SCIS
					(21.79%)	(154)	ring B-prop: 2-CH2-SCIS
					(15.96%)	(163)	bridge BC: C-H ROCK
					(5.51%)	(187)	ring C-prop: 1-CH2-SCIS
74	1424.41	-------	56.40	0.41	(78.71%)	(192)	ring C-prop: 2-CH2-SCIS
75	1414.80	-------	49.11	2.51	(6.73%)	(50)	ring C: C-C stretch (12,11)
					(6.94%)	(59)	ring C-prop: C-C stretch (19,12)
					(7.19%)	(97)	ring C bending
					(5.93%)	(182)	ring C-methyl: ADEFa
					(8.34%)	(192)	ring C-prop: 2-CH2-SCIS
76	1409.08	-------	197.15	10.89	(13.32%)	(25)	ring B: C-C stretch (70,69)
					(6.27%)	(30)	ring B-prop: C-C stretch (75,69)
					(6.11%)	(93)	ring B bending
					(7.08%)	(143)	ring B-methyl: SDEF
					(16.83%)	(166)	bridge CD: C-H ROCK
					(6.56%)	(200)	ring D: N-H ROCK
77	1399.61	-------	38.02	0.86	(8.58%)	(65)	ring C-prop: C-C stretch (25,22)
					(8.59%)	(68)	ring C-prop: C-O stretch (27,25)
					(5.53%)	(190)	ring C-prop: 1-CH2-TWIST
					(8.57%)	(192)	ring C-prop: 2-CH2-SCIS
					(28.83%)	(194)	ring C-prop: 2-CH2-WAGG
					(7.94%)	(197)	ring C-prop: C=O ROCK
					(9.34%)	(199)	ring C-prop: C-O-H BEND
78	1395.45	-------	57.63	0.18	(7.91%)	(36)	ring B-prop: C-C stretch (81,78)
					(9.94%)	(38)	ring B-prop: C-O stretch (83,81)
					(19.20%)	(156)	ring B-prop: 2-CH2-WAGG
					(8.60%)	(159)	ring B-prop: C=O ROCK
					(12.23%)	(161)	ring B-prop: C-O-H BEND
79	1394.48	-------	62.90	1.09	(5.43%)	(50)	ring C: C-C stretch (12,11)
					(11.98%)	(143)	ring B-methyl: SDEF
					(6.87%)	(156)	ring B-prop: 2-CH2-WAGG
					(5.01%)	(161)	ring B-prop: C-O-H BEND
80	1389.34	-------	8.75	0.28	(9.59%)	(181)	ring C-methyl: SDEF
					(68.20%)	(210)	ring D-methyl: SDEF
81	1384.75	-------	4.97	0.61	(5.46%)	(128)	ring A-ethyl: CH3 SDEF
					(5.69%)	(143)	ring B-methyl: SDEF
					(68.42%)	(181)	ring C-methyl: SDEF
					(6.44%)	(210)	ring D-methyl: SDEF
82	1384.17	-------	2.04	0.06	(86.67%)	(128)	ring A-ethyl: CH3 SDEF
83	1380.68	-------	33.17	2.00	(11.40%)	(104)	ring A: N-H ROCK
					(12.33%)	(118)	ring A-methyl: SDEF
					(42.97%)	(143)	ring B-methyl: SDEF
84	1376.45	-------	6.33	1.35	(8.40%)	(104)	ring A: N-H ROCK
					(38.03%)	(118)	ring A-methyl: SDEF
					(19.01%)	(143)	ring B-methyl: SDEF
85	1372.99	-------	27.72	1.43	(9.76%)	(104)	ring A: N-H ROCK
					(41.08%)	(118)	ring A-methyl: SDEF
					(6.82%)	(220)	ring D-ethyl: CH3 SDEF
86	1369.98	-------	3.91	0.08	(6.01%)	(104)	ring A: N-H ROCK
					(76.60%)	(220)	ring D-ethyl: CH3 SDEF
87	1367.01	-------	25.58	5.65	(7.14%)	(44)	bridge AB: C=C stretch (7,53)
					(5.23%)	(54)	ring C: C-C stretch (14,13)
					(27.48%)	(104)	ring A: N-H ROCK
88	1352.49	-------	6.12	0.65	(17.51%)	(112)	ring A-ethyl: SCIS
					(57.17%)	(126)	ring A-ethyl: CH2 WAGG

89	1350.01	-------	22.95	10.92	(5.56%) (71) ring D: C-N stretch (30,28)
					(6.51%) (72) ring D: C-C stretch (31,30)
					(6.93%) (81) ring D-ethyl: C-C stretch (38,32)
					(10.14%) (101) ring D bending
					(9.74%) (200) ring D: N-H ROCK
					(6.53%) (210) ring D-methyl: SDEF
					(16.78%) (218) ring D-ethyl: CH2 WAGG
					(6.37%) (220) ring D-ethyl: CH3 SDEF
90	1340.56	-------	18.62	0.61	(12.25%) (108) ring A-methyl: SCIS
					(8.38%) (112) ring A-ethyl: SCIS
					(6.40%) (114) ring A-ethyl: WAGG
					(8.47%) (126) ring A-ethyl: CH2 WAGG
					(8.47%) (127) ring A-ethyl: CH2 TWIST
					(8.37%) (151) ring B-prop: 1-CH2-WAGG
					(11.01%) (189) ring C-prop: 1-CH2-WAGG
91	1340.30	-------	20.85	2.19	(49.96%) (189) ring C-prop: 1-CH2-WAGG
					(7.12%) (195) ring C-prop: 2-CH2-TWIST
92	1337.10	-------	12.20	0.54	(44.35%) (151) ring B-prop: 1-CH2-WAGG
					(9.90%) (156) ring B-prop: 2-CH2-WAGG
					(9.24%) (161) ring B-prop: C-O-H BEND
93	1336.23	-------	51.01	3.72	(12.63%) (71) ring D: C-N stretch (30,28)
					(5.71%) (76) ring D: C-C stretch (33,32)
					(15.23%) (200) ring D: N-H ROCK
					(5.27%) (208) ring D: C=O ROCK
					(16.30%) (218) ring D-ethyl: CH2 WAGG
94	1331.63	-------	4.49	30.94	(9.34%) (22) ring B: C-C stretch (68,67)
					(5.62%) (24) ring B: C-N stretch (70,65)
					(7.62%) (41) bridge BC: C-.C stretch (3,70)
					(10.72%) (45) bridge AB: C-C stretch (7,67)
					(25.86%) (133) ring B: N-H ROCK
95	1319.09	-------	9.63	1.66	(10.05%) (76) ring D: C-C stretch (33,32)
					(6.57%) (101) ring D bending
					(43.59%) (218) ring D-ethyl: CH2 WAGG
					(9.61%) (219) ring D-ethyl: CH2 TWIST
96	1315.03	-------	46.33	2.48	(11.44%) (190) ring C-prop: 1-CH2-TWIST
					(21.48%) (194) ring C-prop: 2-CH2-WAGG
					(5.26%) (197) ring C-prop: C=O ROCK
					(35.19%) (199) ring C-prop: C-O-H BEND
97	1307.61	-------	26.46	1.02	(31.27%) (108) ring A-methyl: SCIS
					(5.61%) (109) ring A-methyl: ROCK
					(5.69%) (112) ring A-ethyl: SCIS
					(30.56%) (127) ring A-ethyl: CH2 TWIST
98	1302.06	-------	253.75	23.77	(6.22%) (25) ring B: C-C stretch (70,69)
					(8.86%) (152) ring B-prop: 1-CH2-TWIST
					(7.31%) (171) ring C: N-H ROCK
					(5.01%) (194) ring C-prop: 2-CH2-WAGG
					(5.71%) (199) ring C-prop: C-O-H BEND
99	1290.38	-------	46.84	0.36	(25.21%) (110) ring A-methyl: WAGG
					(8.39%) (111) ring A-methyl: TWIST
					(11.31%) (114) ring A-ethyl: WAGG
					(5.95%) (115) ring A-ethyl: TWIST
					(5.41%) (126) ring A-ethyl: CH2 WAGG
100	1288.46	-------	19.31	2.48	(9.85%) (151) ring B-prop: 1-CH2-WAGG
					(11.03%) (152) ring B-prop: 1-CH2-TWIST
					(15.00%) (156) ring B-prop: 2-CH2-WAGG
					(11.35%) (157) ring B-prop: 2-CH2-TWIST
					(20.70%) (161) ring B-prop: C-O-H BEND
101	1280.89	-------	186.10	8.42	(24.33%) (53) ring C: C-N stretch (14, 9)
					(15.54%) (171) ring C: N-H ROCK
					(10.35%) (200) ring D: N-H ROCK

102	1273.81	-------	6.67	2.77	(58.81%) (219) ring D-ethyl: CH2 TWIST
					(16.19%) (224) ring D-ethyl: CH3 ROCKb
103	1256.23	-------	50.44	0.72	(6.05%) (108) ring A-methyl: SCIS
					(15.87%) (112) ring A-ethyl: SCIS
					(6.52%) (125) ring A-ethyl: CH2 ROCK
					(21.34%) (127) ring A-ethyl: CH2 TWIST
					(12.03%) (131) ring A-ethyl: CH3 ROCKa
104	1255.30	-------	13.43	0.24	(13.20%) (151) ring B-prop: 1-CH2-WAGG
					(21.87%) (152) ring B-prop: 1-CH2-TWIST
					(20.64%) (156) ring B-prop: 2-CH2-WAGG
					(10.91%) (157) ring B-prop: 2-CH2-TWIST
105	1242.81	-------	35.19	0.24	(12.65%) (166) bridge CD: C-H ROCK
					(33.52%) (190) ring C-prop: 1-CH2-TWIST
					(7.42%) (194) ring C-prop: 2-CH2-WAGG
					(6.40%) (195) ring C-prop: 2-CH2-TWIST
106	1239.10	-------	372.22	0.21	(9.32%) (110) ring A-methyl: WAGG
					(5.59%) (111) ring A-methyl: TWIST
					(8.77%) (114) ring A-ethyl: WAGG
					(6.29%) (115) ring A-ethyl: TWIST
					(7.13%) (166) bridge CD: C-H ROCK
					(5.83%) (169) bridge AB: C-H ROCK
107	1238.88	-------	63.74	0.67	(7.38%) (88) ring A bending
					(8.38%) (110) ring A-methyl: WAGG
					(11.66%) (114) ring A-ethyl: WAGG
					(5.44%) (115) ring A-ethyl: TWIST
108	1224.57	-------	559.21	1.64	(6.91%) (53) ring C: C-N stretch (14, 9)
					(5.16%) (157) ring B-prop: 2-CH2-TWIST
					(10.04%) (166) bridge CD: C-H ROCK
					(19.41%) (195) ring C-prop: 2-CH2-TWIST
109	1204.04	-------	65.91	0.30	(7.99%) (152) ring B-prop: 1-CH2-TWIST
					(10.60%) (157) ring B-prop: 2-CH2-TWIST
					(9.79%) (190) ring C-prop: 1-CH2-TWIST
					(33.96%) (195) ring C-prop: 2-CH2-TWIST
110	1199.24	-------	348.23	1.21	(5.14%) (38) ring B-prop: C-O stretch (83,81)
					(13.52%) (152) ring B-prop: 1-CH2-TWIST
					(16.65%) (157) ring B-prop: 2-CH2-TWIST
					(5.98%) (161) ring B-prop: C-O-H BEND
					(8.51%) (195) ring C-prop: 2-CH2-TWIST
111	1176.99	-------	367.85	0.11	(16.96%) (2) ring A: C-N stretch (48,46)
					(5.41%) (3) ring A: C-C stretch (49,48)
					(16.28%) (7) ring A: C-N stretch (53,46)
					(7.81%) (8) ring A: C-C stretch (53,51)
					(5.10%) (21) ring B: C-N stretch (67,65)
					(9.57%) (169) bridge AB: C-H ROCK
112	1166.72	-------	80.23	3.25	(14.07%) (49) ring C: C-N stretch (11, 9)
					(7.02%) (50) ring C: C-C stretch (12,11)
					(8.11%) (52) bridge CD: C-C stretch (14, 5)
					(12.13%) (53) ring C: C-N stretch (14, 9)
					(13.72%) (55) ring C-methyl: C-C stretch (15,13)
					(8.10%) (97) ring C bending
113	1152.54	-------	183.38	2.13	(5.42%) (26) ring B-methyl: C-C stretch (71,68)
					(24.63%) (38) ring B-prop: C-O stretch (83,81)
					(5.14%) (68) ring C-prop: C-O stretch (27,25)
					(7.53%) (157) ring B-prop: 2-CH2-TWIST
					(11.14%) (161) ring B-prop: C-O-H BEND
114	1147.96	-------	247.40	0.07	(23.90%) (68) ring C-prop: C-O stretch (27,25)
					(6.08%) (194) ring C-prop: 2-CH2-WAGG
					(11.11%) (199) ring C-prop: C-O-H BEND
115	1147.46	-------	63.64	0.05	(6.61%) (5) ring A: C-C stretch (51,49)
					(5.62%) (109) ring A-methyl: ROCK
					(7.48%) (113) ring A-ethyl: ROCK

					(9.77%) (121) ring A-methyl: ROCKa
					(6.51%) (122) ring A-methyl: ROCKb
					(15.38%) (125) ring A-ethyl: CH2 ROCK
					(7.39%) (131) ring A-ethyl: CH3 ROCKa
116	1145.03	-------	113.18	0.24	(5.39%) (68) ring C-prop: C-O stretch (27,25)
					(10.77%) (77) ring D-methyl: C-C stretch (34,31)
					(5.23%) (81) ring D-ethyl: C-C stretch (38,32)
					(15.42%) (217) ring D-ethyl: CH2 ROCK
					(12.49%) (224) ring D-ethyl: CH3 ROCKb
117	1127.26	-------	93.83	1.40	(8.51%) (24) ring B: C-N stretch (70,65)
					(5.42%) (30) ring B-prop: C-C stretch (75,69)
					(7.35%) (59) ring C-prop: C-C stretch (19,12)
					(11.59%) (184) ring C-methyl: ROCKa
118	1122.88	-------	74.27	1.12	(10.34%) (21) ring B: C-N stretch (67,65)
					(9.28%) (24) ring B: C-N stretch (70,65)
					(6.28%) (25) ring B: C-C stretch (70,69)
					(7.94%) (26) ring B-methyl: C-C stretch (71,68)
					(6.51%) (45) bridge AB: C-C stretch (7,67)
					(5.28%) (49) ring C: C-N stretch (11, 9)
					(5.01%) (184) ring C-methyl: ROCKa
119	1117.96	-------	68.62	1.38	(7.95%) (81) ring D-ethyl: C-C stretch (38,32)
					(13.50%) (213) ring D-methyl: ROCKa
					(10.85%) (214) ring D-methyl: ROCKb
					(6.10%) (217) ring D-ethyl: CH2 ROCK
					(5.80%) (224) ring D-ethyl: CH3 ROCKb
120	1112.33	-------	197.00	1.17	(8.64%) (24) ring B: C-N stretch (70,65)
					(12.02%) (30) ring B-prop: C-C stretch (75,69)
					(13.80%) (147) ring B-methyl: ROCKb
					(6.82%) (184) ring C-methyl: ROCKa
121	1100.12	-------	129.36	5.98	(25.87%) (71) ring D: C-N stretch (30,28)
					(22.99%) (75) ring D: C-N stretch (33,28)
					(9.29%) (77) ring D-methyl: C-C stretch (34,31)
					(6.22%) (81) ring D-ethyl: C-C stretch (38,32)
					(7.89%) (200) ring D: N-H ROCK
122	1094.43	-------	88.51	0.80	(10.94%) (5) ring A: C-C stretch (51,49)
					(6.34%) (9) ring A-methyl: C-C stretch (54,49)
					(13.55%) (13) ring A-ethyl: C-C stretch (58,51)
					(6.07%) (115) ring A-ethyl: TWIST
					(7.03%) (123) ring A-ethyl: BEND
					(7.27%) (131) ring A-ethyl: CH3 ROCKa
					(15.62%) (132) ring A-ethyl: CH3 ROCKb
123	1083.02	-------	24.24	0.04	(11.16%) (9) ring A-methyl: C-C stretch (54,49)
					(8.74%) (111) ring A-methyl: TWIST
					(5.18%) (121) ring A-methyl: ROCKa
					(22.64%) (122) ring A-methyl: ROCKb
					(14.25%) (132) ring A-ethyl: CH3 ROCKb
124	1072.49	-------	57.03	1.40	(5.26%) (147) ring B-methyl: ROCKb
					(6.45%) (150) ring B-prop: 1-CH2-ROCK
					(9.21%) (184) ring C-methyl: ROCKa
					(7.77%) (188) ring C-prop: 1-CH2-ROCK
125	1061.83	-------	141.01	2.17	(14.74%) (84) ring D-ethyl: C-C stretch (41,38)
					(12.88%) (213) ring D-methyl: ROCKa
					(8.45%) (215) ring D-ethyl: BEND
					(40.12%) (223) ring D-ethyl: CH3 ROCKa
126	1052.99	-------	12.26	0.93	(6.94%) (178) ring C-methyl: WAGG
					(7.16%) (183) ring C-methyl: ADEFb
					(75.67%) (185) ring C-methyl: ROCKb
127	1047.60	-------	31.17	0.62	(14.30%) (146) ring B-methyl: ROCKa
					(11.68%) (150) ring B-prop: 1-CH2-ROCK
					(8.56%) (155) ring B-prop: 2-CH2-ROCK
					(7.75%) (188) ring C-prop: 1-CH2-ROCK

128	1046.70	-------	2.98	0.10	(8.23%) (138) ring B-methyl: WAGG
					(50.96%) (146) ring B-methyl: ROCKa
					(23.89%) (147) ring B-methyl: ROCKb
129	1040.10	-------	1.43	0.36	(7.27%) (205) ring D-methyl: WAGG
					(21.72%) (213) ring D-methyl: ROCKa
					(51.18%) (214) ring D-methyl: ROCKb
					(7.47%) (223) ring D-ethyl: CH3 ROCKa
130	1027.02	-------	19.87	0.06	(6.46%) (5) ring A: C-C stretch (51,49)
					(5.91%) (13) ring A-ethyl: C-C stretch (58,51)
					(10.74%) (15) ring A-ethyl: C-C stretch (60,58)
					(9.77%) (109) ring A-methyl: ROCK
					(28.63%) (121) ring A-methyl: ROCKa
					(5.25%) (125) ring A-ethyl: CH2 ROCK
					(7.22%) (127) ring A-ethyl: CH2 TWIST
					(6.59%) (132) ring A-ethyl: CH3 ROCKb
131	1025.04	-------	1.43	0.07	(10.70%) (9) ring A-methyl: C-C stretch (54,49)
					(7.19%) (13) ring A-ethyl: C-C stretch (58,51)
					(17.17%) (15) ring A-ethyl: C-C stretch (60,58)
					(6.67%) (112) ring A-ethyl: SCIS
					(16.96%) (122) ring A-methyl: ROCKb
					(10.11%) (131) ring A-ethyl: CH3 ROCKa
132	1020.71	-------	197.30	0.33	(10.62%) (2) ring A: C-N stretch (48,46)
					(5.66%) (7) ring A: C-N stretch (53,46)
					(25.48%) (9) ring A-methyl: C-C stretch (54,49)
					(15.81%) (13) ring A-ethyl: C-C stretch (58,51)
					(15.83%) (15) ring A-ethyl: C-C stretch (60,58)
133	1001.20	-------	49.92	0.97	(5.10%) (51) ring C: C-.C stretch (13,12)
					(44.25%) (62) ring C-prop: C-C stretch (22,19)
					(13.74%) (184) ring C-methyl: ROCKa
					(6.40%) (193) ring C-prop: 2-CH2-ROCK
134	995.55	-------	14.76	0.14	(68.45%) (33) ring B-prop: C-C stretch (78,75)
					(6.04%) (148) ring B-prop: 1-BEND
					(5.39%) (153) ring B-prop: 2-BEND
					(5.14%) (160) ring B-prop: C=O OUT
135	989.06	-------	36.38	2.78	(8.93%) (73) ring D: C=C stretch (32,31)
					(10.22%) (76) ring D: C-C stretch (33,32)
					(7.69%) (77) ring D-methyl: C-C stretch (34,31)
					(11.00%) (84) ring D-ethyl: C-C stretch (41,38)
					(17.51%) (213) ring D-methyl: ROCKa
					(7.99%) (214) ring D-methyl: ROCKb
					(8.22%) (219) ring D-ethyl: CH2 TWIST
					(10.85%) (224) ring D-ethyl: CH3 ROCKb
136	958.16	-------	158.70	0.19	(6.95%) (23) ring B: C-.C stretch (69,68)
					(5.65%) (25) ring B: C-C stretch (70,69)
					(12.96%) (147) ring B-methyl: ROCKb
					(12.29%) (150) ring B-prop: 1-CH2-ROCK
					(13.51%) (155) ring B-prop: 2-CH2-ROCK
137	950.36	-------	39.99	0.20	(5.37%) (62) ring C-prop: C-C stretch (22,19)
					(6.75%) (186) ring C-prop: 1-BEND
					(5.45%) (188) ring C-prop: 1-CH2-ROCK
					(5.20%) (191) ring C-prop: 2-BEND
					(28.51%) (193) ring C-prop: 2-CH2-ROCK
					(14.32%) (198) ring C-prop: C=O OUT
138	946.09	-------	16.93	1.12	(5.75%) (5) ring A: C-C stretch (51,49)
					(8.45%) (7) ring A: C-N stretch (53,46)
					(10.16%) (8) ring A: C-C stretch (53,51)
					(27.64%) (15) ring A-ethyl: C-C stretch (60,58)
					(5.64%) (122) ring A-methyl: ROCKb
					(6.05%) (132) ring A-ethyl: CH3 ROCKb
139	935.82	-------	30.35	0.94	(8.07%) (76) ring D: C-C stretch (33,32)
					(56.02%) (84) ring D-ethyl: C-C stretch (41,38)
					(5.13%) (218) ring D-ethyl: CH2 WAGG
					(13.02%) (223) ring D-ethyl: CH3 ROCKa

140	935.15	-------	142.43	1.14	(5.25%) (49) ring C: C-N stretch (11, 9)
					(7.65%) (50) ring C: C-C stretch (12,11)
					(6.90%) (62) ring C-prop: C-C stretch (22,19)
					(12.82%) (65) ring C-prop: C-C stretch (25,22)
					(24.22%) (188) ring C-prop: 1-CH2-ROCK
					(5.70%) (193) ring C-prop: 2-CH2-ROCK
141	924.10	-------	32.38	0.23	(10.06%) (8) ring A: C-C stretch (53,51)
					(5.42%) (36) ring B-prop: C-C stretch (81,78)
					(6.18%) (147) ring B-methyl: ROCKb
					(7.98%) (155) ring B-prop: 2-CH2-ROCK
142	909.36	-------	9.00	0.13	(10.70%) (5) ring A: C-C stretch (51,49)
					(12.58%) (8) ring A: C-C stretch (53,51)
					(9.82%) (9) ring A-methyl: C-C stretch (54,49)
					(6.42%) (36) ring B-prop: C-C stretch (81,78)
					(11.28%) (132) ring A-ethyl: CH3 ROCKb
143	893.50	-------	22.90	0.14	(14.25%) (36) ring B-prop: C-C stretch (81,78)
144	886.35	-------	13.60	1.52	(61.88%) (164) bridge BC: C-H OUT
					(10.56%) (233) bridge BC: 1-TORS
					(7.64%) (236) bridge BC: 2-TORS
145	876.05	-------	69.55	2.38	(5.33%) (52) bridge CD: C-C stretch (14, 5)
					(12.35%) (54) ring C: C-C stretch (14,13)
					(5.98%) (72) ring D: C-C stretch (31,30)
146	861.00	-------	3.16	0.18	(7.74%) (5) ring A: C-C stretch (51,49)
					(12.57%) (13) ring A-ethyl: C-C stretch (58,51)
					(8.26%) (122) ring A-methyl: ROCKb
					(20.17%) (131) ring A-ethyl: CH3 ROCKa
147	830.03	-------	98.67	1.04	(11.41%) (72) ring D: C-C stretch (31,30)
					(5.27%) (224) ring D-ethyl: CH3 ROCKb
148	822.25	-------	51.07	1.99	(23.59%) (3) ring A: C-C stretch (49,48)
					(20.34%) (105) ring A: N-H OUT
149	811.58	-------	28.96	57.73	(55.16%) (167) bridge CD: C-H OUT
					(8.91%) (203) ring D: bridge CD WAGG
					(11.40%) (243) bridge C=D: TORS
150	804.67	-------	2.95	4.05	(5.22%) (36) ring B-prop: C-C stretch (81,78)
					(8.55%) (65) ring C-prop: C-C stretch (25,22)
					(6.15%) (165) bridge CD: BEND
					(5.01%) (217) ring D-ethyl: CH2 ROCK
					(5.68%) (224) ring D-ethyl: CH3 ROCKb
151	798.28	-------	15.26	0.15	(61.53%) (170) bridge AB: C-H OUT
					(12.47%) (234) bridge A=B: TORS
					(8.52%) (235) bridge A-B: TORS
152	793.48	-------	27.18	1.43	(10.06%) (36) ring B-prop: C-C stretch (81,78)
					(9.99%) (65) ring C-prop: C-C stretch (25,22)
					(9.07%) (150) ring B-prop: 1-CH2-ROCK
					(5.81%) (168) bridge AB: BEND
153	787.59	-------	44.74	0.29	(13.79%) (65) ring C-prop: C-C stretch (25,22)
					(5.97%) (68) ring C-prop: C-O stretch (27,25)
					(7.95%) (96) ring C bending
					(7.16%) (165) bridge CD: BEND
					(6.14%) (188) ring C-prop: 1-CH2-ROCK
					(6.33%) (217) ring D-ethyl: CH2 ROCK
					(5.20%) (224) ring D-ethyl: CH3 ROCKb
154	782.88	-------	68.33	0.46	(17.00%) (105) ring A: N-H OUT
					(6.53%) (125) ring A-ethyl: CH2 ROCK
					(13.66%) (134) ring B: N-H OUT
					(8.81%) (172) ring C: N-H OUT
155	773.10	-------	2.59	1.00	(11.05%) (102) ring D torsion
					(12.83%) (207) ring D-ethyl: WAGG

					(49.35%) (209) ring D: C=O OUT
156	767.76	-------	41.14	0.92	(14.53%) (105) ring A: N-H OUT
157	757.85	-------	5.81	0.36	(5.92%) (5) ring A: C-C stretch (51,49)
					(5.45%) (121) ring A-methyl: ROCKa
					(28.66%) (125) ring A-ethyl: CH2 ROCK
					(6.36%) (131) ring A-ethyl: CH3 ROCKa
					(6.08%) (132) ring A-ethyl: CH3 ROCKb
					(5.38%) (172) ring C: N-H OUT
158	754.22	-------	6.88	0.15	(7.42%) (76) ring D: C-C stretch (33,32)
					(5.03%) (100) ring D bending
					(5.26%) (180) ring C: bridge CD WAGG
					(5.83%) (209) ring D: C=O OUT
					(25.09%) (217) ring D-ethyl: CH2 ROCK
					(12.56%) (224) ring D-ethyl: CH3 ROCKb
159	744.19	-------	24.38	0.65	(5.58%) (88) ring A bending
					(5.80%) (100) ring D bending
					(5.56%) (172) ring C: N-H OUT
160	734.27	-------	16.83	0.39	(14.01%) (94) ring B torsion
					(10.08%) (140) ring B-prop: 1-WAGG
					(5.86%) (142) ring B: bridge BC WAGG
					(6.22%) (160) ring B-prop: C=O OUT
					(5.49%) (232) ring B-prop: 4-TORS
161	724.05	-------	33.12	11.80	(9.39%) (96) ring C bending
					(22.71%) (100) ring D bending
					(8.54%) (172) ring C: N-H OUT
162	723.29	-------	28.20	2.58	(8.67%) (92) ring B bending
					(19.91%) (105) ring A: N-H OUT
					(18.41%) (134) ring B: N-H OUT
					(12.57%) (172) ring C: N-H OUT
163	719.51	-------	20.21	0.28	(9.03%) (98) ring C torsion
					(5.71%) (99) ring C torsion
					(7.03%) (174) ring C: bridge BC WAGG
					(7.74%) (176) ring C-prop: 1-WAGG
164	707.81	-------	9.46	0.35	(13.69%) (95) ring B torsion
					(13.29%) (107) ring A: C=O OUT
					(25.39%) (136) ring B: bridge AB WAGG
					(7.52%) (232) ring B-prop: 4-TORS
					(7.58%) (234) bridge A=B: TORS
165	699.57	-------	77.31	1.46	(5.47%) (100) ring D bending
					(7.52%) (155) ring B-prop: 2-CH2-ROCK
					(11.74%) (172) ring C: N-H OUT
					(21.14%) (232) ring B-prop: 4-TORS
166	696.22	-------	17.84	0.17	(10.28%) (107) ring A: C=O OUT
					(9.66%) (134) ring B: N-H OUT
					(17.40%) (172) ring C: N-H OUT
					(19.08%) (232) ring B-prop: 4-TORS
167	685.98	-------	55.00	2.77	(8.28%) (98) ring C torsion
					(7.99%) (198) ring C-prop: C=O OUT
					(29.85%) (242) ring C-prop: 4-TORS
168	680.83	-------	40.52	0.16	(5.44%) (92) ring B bending
					(7.70%) (95) ring B torsion
					(5.20%) (99) ring C torsion
					(20.11%) (242) ring C-prop: 4-TORS
169	675.84	-------	55.14	2.26	(9.85%) (99) ring C torsion
					(21.71%) (134) ring B: N-H OUT
					(8.74%) (172) ring C: N-H OUT
170	673.65	-------	5.51	0.31	(5.20%) (81) ring D-ethyl: C-C stretch (38,32)
					(6.39%) (100) ring D bending
					(5.78%) (107) ring A: C=O OUT

					(10.39%) (134) ring B: N-H OUT
					(5.43%) (172) ring C: N-H OUT
					(8.44%) (242) ring C-prop: 4-TORS
171	668.90	-------	15.48	0.71	(11.35%) (55) ring C-methyl: C-C stretch (15,13)
172	659.60	-------	2.93	0.67	(16.12%) (88) ring A bending
					(19.65%) (117) ring A: bridge AB WAGG
173	656.05	-------	17.33	7.12	(6.77%) (99) ring C torsion
					(7.70%) (208) ring D: C=O ROCK
					(6.35%) (209) ring D: C=O OUT
174	653.02	-------	16.22	29.70	(9.68%) (102) ring D torsion
					(15.61%) (103) ring D torsion
					(25.37%) (203) ring D: bridge CD WAGG
					(8.89%) (205) ring D-methyl: WAGG
175	630.95	-------	18.20	0.74	(10.81%) (26) ring B-methyl: C-C stretch (71,68)
					(8.87%) (93) ring B bending
					(15.81%) (159) ring B-prop: C=O ROCK
					(5.57%) (232) ring B-prop: 4-TORS
176	619.91	-------	46.55	0.34	(12.07%) (159) ring B-prop: C=O ROCK
					(18.54%) (197) ring C-prop: C=O ROCK
					(7.21%) (242) ring C-prop: 4-TORS
177	610.75	-------	29.74	0.28	(6.31%) (13) ring A-ethyl: C-C stretch (58,51)
					(8.87%) (88) ring A bending
					(34.92%) (89) ring A bending
					(18.07%) (117) ring A: bridge AB WAGG
178	605.08	-------	17.13	1.01	(8.01%) (93) ring B bending
					(7.31%) (159) ring B-prop: C=O ROCK
					(25.31%) (197) ring C-prop: C=O ROCK
179	593.95	-------	19.30	0.30	(7.29%) (26) ring B-methyl: C-C stretch (71,68)
					(8.06%) (55) ring C-methyl: C-C stretch (15,13)
					(5.97%) (59) ring C-prop: C-C stretch (19,12)
					(12.16%) (93) ring B bending
					(18.39%) (97) ring C bending
180	572.31	-------	21.76	0.46	(6.12%) (77) ring D-methyl: C-C stretch (34,31)
					(7.84%) (208) ring D: C=O ROCK
181	560.21	-------	17.82	0.15	(6.25%) (142) ring B: bridge BC WAGG
					(7.69%) (148) ring B-prop: 1-BEND
					(6.22%) (158) ring B-prop: 3-BEND
					(9.33%) (159) ring B-prop: C=O ROCK
					(16.15%) (160) ring B-prop: C=O OUT
					(12.49%) (232) ring B-prop: 4-TORS
182	555.66	-------	11.93	1.22	(9.67%) (77) ring D-methyl: C-C stretch (34,31)
					(29.29%) (101) ring D bending
					(5.15%) (207) ring D-ethyl: WAGG
					(5.62%) (209) ring D: C=O OUT
					(9.65%) (215) ring D-ethyl: BEND
183	533.90	-------	13.35	2.33	(20.77%) (106) ring A: C=O ROCK
					(5.14%) (116) ring A: bridge AB ROCK
					(5.05%) (139) ring B-prop: 1-ROCK
184	520.58	-------	9.16	0.26	(8.65%) (196) ring C-prop: 3-BEND
					(5.80%) (198) ring C-prop: C=O OUT
185	511.66	-------	53.87	2.55	(6.20%) (106) ring A: C=O ROCK
					(5.36%) (193) ring C-prop: 2-CH2-ROCK
					(9.56%) (198) ring C-prop: C=O OUT
					(10.63%) (201) ring D: N-H OUT
186	502.51	-------	2.12	4.14	(8.65%) (101) ring D bending
					(5.18%) (193) ring C-prop: 2-CH2-ROCK
					(7.10%) (198) ring C-prop: C=O OUT
					(17.67%) (201) ring D: N-H OUT

					(6.36%) (205) ring D-methyl: WAGG
					(6.17%) (207) ring D-ethyl: WAGG
					(6.96%) (209) ring D: C=O OUT
187	499.74	-------	10.60	1.91	(5.36%) (93) ring B bending
					(6.69%) (106) ring A: C=O ROCK
					(11.74%) (137) ring B-methyl: ROCK
					(6.88%) (139) ring B-prop: 1-ROCK
					(5.09%) (196) ring C-prop: 3-BEND
188	490.38	-------	6.83	0.68	(5.63%) (193) ring C-prop: 2-CH2-ROCK
					(9.54%) (196) ring C-prop: 3-BEND
					(15.53%) (198) ring C-prop: C=O OUT
					(5.30%) (208) ring D: C=O ROCK
189	481.68	-------	6.95	1.65	(5.02%) (140) ring B-prop: 1-WAGG
					(6.59%) (142) ring B: bridge BC WAGG
					(7.04%) (196) ring C-prop: 3-BEND
190	452.18	-------	67.09	9.35	(-10.35%) (103) ring D torsion
					(68.07%) (201) ring D: N-H OUT
					(10.69%) (203) ring D: bridge CD WAGG
					(8.69%) (207) ring D-ethyl: WAGG
					(6.85%) (215) ring D-ethyl: BEND
191	440.04	-------	2.90	0.21	(6.31%) (5) ring A: C-C stretch (51,49)
					(5.25%) (9) ring A-methyl: C-C stretch (54,49)
					(5.59%) (13) ring A-ethyl: C-C stretch (58,51)
					(10.00%) (109) ring A-methyl: ROCK
					(5.04%) (112) ring A-ethyl: SCIS
					(10.27%) (113) ring A-ethyl: ROCK
					(5.21%) (123) ring A-ethyl: BEND
192	430.77	-------	10.38	0.82	(6.85%) (155) ring B-prop: 2-CH2-ROCK
					(29.31%) (158) ring B-prop: 3-BEND
					(5.73%) (196) ring C-prop: 3-BEND
193	404.31	-------	6.56	1.07	(7.23%) (123) ring A-ethyl: BEND
					(5.15%) (136) ring B: bridge AB WAGG
					(7.58%) (138) ring B-methyl: WAGG
					(10.70%) (158) ring B-prop: 3-BEND
					(6.06%) (174) ring C: bridge BC WAGG
194	401.42	-------	7.96	0.15	(7.39%) (106) ring A: C=O ROCK
					(15.32%) (123) ring A-ethyl: BEND
195	360.57	-------	0.29	0.71	(9.53%) (178) ring C-methyl: WAGG
					(8.10%) (179) ring C: bridge CD ROCK
					(6.98%) (205) ring D-methyl: WAGG
196	351.05	-------	2.00	1.71	(7.27%) (175) ring C-prop: 1-ROCK
					(23.69%) (178) ring C-methyl: WAGG
					(7.13%) (186) ring C-prop: 1-BEND
					(12.82%) (196) ring C-prop: 3-BEND
					(7.93%) (205) ring D-methyl: WAGG
197	343.06	-------	5.72	1.20	(5.62%) (123) ring A-ethyl: BEND
					(9.73%) (137) ring B-methyl: ROCK
					(9.26%) (138) ring B-methyl: WAGG
					(21.49%) (139) ring B-prop: 1-ROCK
					(5.58%) (153) ring B-prop: 2-BEND
198	327.54	-------	0.97	6.28	(8.70%) (137) ring B-methyl: ROCK
					(28.93%) (138) ring B-methyl: WAGG
					(7.40%) (139) ring B-prop: 1-ROCK
					(5.33%) (140) ring B-prop: 1-WAGG
					(11.45%) (158) ring B-prop: 3-BEND
199	319.78	-------	2.66	24.64	(14.56%) (175) ring C-prop: 1-ROCK
					(5.46%) (177) ring C-methyl: ROCK
					(6.01%) (191) ring C-prop: 2-BEND
					(8.65%) (205) ring D-methyl: WAGG
					(7.66%) (215) ring D-ethyl: BEND

200	317.50	-------	2.56	0.32	(41.70%) (204) ring D-methyl: ROCK
					(13.20%) (205) ring D-methyl: WAGG
					(8.15%) (208) ring D: C=O ROCK
					(-5.91%) (237) bridge C-D: TORS
201	304.58	-------	2.22	4.95	(12.16%) (95) ring B torsion
					(19.77%) (177) ring C-methyl: ROCK
					(12.02%) (243) bridge C=D: TORS
					(5.69%) (246) ring D-ethyl: 2-TORS
202	294.90	-------	0.77	1.93	(14.50%) (178) ring C-methyl: WAGG
					(14.12%) (206) ring D-ethyl: ROCK
					(26.21%) (246) ring D-ethyl: 2-TORS
203	292.58	-------	2.65	6.77	(5.54%) (97) ring C bending
					(6.47%) (175) ring C-prop: 1-ROCK
					(8.49%) (177) ring C-methyl: ROCK
					(5.68%) (191) ring C-prop: 2-BEND
					(5.76%) (204) ring D-methyl: ROCK
204	287.59	-------	2.30	16.49	(6.63%) (123) ring A-ethyl: BEND
					(6.77%) (204) ring D-methyl: ROCK
					(8.07%) (206) ring D-ethyl: ROCK
					(10.88%) (246) ring D-ethyl: 2-TORS
205	280.30	-------	2.19	7.43	(5.10%) (111) ring A-methyl: TWIST
					(13.97%) (123) ring A-ethyl: BEND
					(5.25%) (137) ring B-methyl: ROCK
206	270.03	-------	0.63	0.14	(7.39%) (177) ring C-methyl: ROCK
					(23.13%) (227) ring A-ethyl: 2-TORS
207	257.24	-------	2.86	0.59	(10.19%) (109) ring A-methyl: ROCK
					(6.82%) (225) ring A-methyl: TORS
					(22.10%) (227) ring A-ethyl: 2-TORS
208	248.59	-------	1.62	0.30	(7.17%) (110) ring A-methyl: WAGG
					(7.17%) (137) ring B-methyl: ROCK
					(5.86%) (138) ring B-methyl: WAGG
					(21.90%) (225) ring A-methyl: TORS
209	238.15	-------	0.53	0.29	(10.90%) (111) ring A-methyl: TWIST
					(57.64%) (225) ring A-methyl: TORS
210	234.44	-------	1.99	0.70	(6.61%) (202) ring D: bridge CD ROCK
					(9.10%) (225) ring A-methyl: TORS
					(13.05%) (227) ring A-ethyl: 2-TORS
					(5.18%) (234) bridge A=B: TORS
211	224.79	-------	2.56	0.87	(7.67%) (135) ring B: bridge AB ROCK
					(6.30%) (137) ring B-methyl: ROCK
					(5.33%) (202) ring D: bridge CD ROCK
					(6.18%) (227) ring A-ethyl: 2-TORS
					(34.01%) (238) ring C-methyl: TORS
212	220.35	-------	0.76	1.34	(37.79%) (238) ring C-methyl: TORS
					(9.00%) (244) ring D-methyl: TORS
					(8.74%) (246) ring D-ethyl: 2-TORS
213	216.52	-------	1.41	0.60	(9.55%) (135) ring B: bridge AB ROCK
					(10.52%) (137) ring B-methyl: ROCK
					(10.79%) (153) ring B-prop: 2-BEND
					(11.04%) (246) ring D-ethyl: 2-TORS
214	209.36	-------	2.41	0.83	(6.19%) (176) ring C-prop: 1-WAGG
					(9.41%) (206) ring D-ethyl: ROCK
					(13.76%) (246) ring D-ethyl: 2-TORS
215	197.90	-------	4.06	2.24	(8.91%) (91) ring A torsion
					(7.47%) (141) ring B: bridge BC ROCK
					(7.20%) (235) bridge A-B: TORS
					(6.47%) (246) ring D-ethyl: 2-TORS
					(11.06%) (248) Cl-H stretch

216	188.85	-------	1.86	3.65	(7.92%) (153) ring B-prop: 2-BEND
					(6.04%) (186) ring C-prop: 1-BEND
					(5.53%) (227) ring A-ethyl: 2-TORS
217	185.18	-------	2.55	2.38	(8.28%) (102) ring D torsion
					(6.94%) (205) ring D-methyl: WAGG
					(6.05%) (207) ring D-ethyl: WAGG
					(8.27%) (215) ring D-ethyl: BEND
					(5.98%) (237) bridge C-D: TORS
					(16.14%) (248) Cl-H stretch
218	180.22	-------	3.81	0.28	(10.32%) (91) ring A torsion
					(5.51%) (123) ring A-ethyl: BEND
219	169.20	-------	3.87	0.33	(9.19%) (91) ring A torsion
					(6.59%) (95) ring B torsion
					(5.92%) (109) ring A-methyl: ROCK
					(6.78%) (162) bridge BC: BEND
					(14.34%) (235) bridge A-B: TORS
					(12.10%) (248) Cl-H stretch
220	167.17	-------	5.41	2.81	(5.30%) (91) ring A torsion
					(9.19%) (207) ring D-ethyl: WAGG
					(9.34%) (215) ring D-ethyl: BEND
					(32.71%) (248) Cl-H stretch
221	164.84	-------	0.05	1.33	(6.47%) (98) ring C torsion
					(13.82%) (237) bridge C-D: TORS
					(6.63%) (238) ring C-methyl: TORS
					(8.09%) (243) bridge C=D: TORS
					(8.37%) (244) ring D-methyl: TORS
					(5.00%) (248) Cl-H stretch
222	151.21	-------	1.10	8.04	(8.00%) (103) ring D torsion
					(8.63%) (153) ring B-prop: 2-BEND
					(5.25%) (237) bridge C-D: TORS
					(16.83%) (247) Cl-H stretch
223	147.29	-------	5.34	0.32	(11.83%) (91) ring A torsion
					(5.06%) (114) ring A-ethyl: WAGG
					(6.88%) (115) ring A-ethyl: TWIST
					(-6.80%) (235) bridge A-B: TORS
					(29.35%) (247) Cl-H stretch
					(8.76%) (249) Cl OUT
224	140.87	-------	1.04	1.49	(5.06%) (91) ring A torsion
					(5.28%) (102) ring D torsion
					(12.58%) (103) ring D torsion
					(5.46%) (141) ring B: bridge BC ROCK
					(19.21%) (228) ring B-methyl: TORS
225	137.81	-------	3.52	0.24	(12.76%) (103) ring D torsion
					(18.39%) (228) ring B-methyl: TORS
					(10.16%) (244) ring D-methyl: TORS
					(5.28%) (247) Cl-H stretch
226	131.78	-------	1.00	0.50	(5.26%) (226) ring A-ethyl: 1-TORS
					(50.18%) (228) ring B-methyl: TORS
					(12.62%) (244) ring D-methyl: TORS
227	127.20	-------	0.53	0.33	(5.82%) (116) ring A: bridge AB ROCK
					(5.44%) (168) bridge AB: BEND
					(6.27%) (175) ring C-prop: 1-ROCK
					(16.66%) (226) ring A-ethyl: 1-TORS
					(6.35%) (247) Cl-H stretch
228	122.15	-------	0.83	5.43	(7.23%) (174) ring C: bridge BC WAGG
					(37.78%) (244) ring D-methyl: TORS
229	104.39	-------	1.03	1.47	(6.55%) (173) ring C: bridge BC ROCK
					(32.36%) (226) ring A-ethyl: 1-TORS
230	101.53	-------	0.30	7.88	(5.74%) (98) ring C torsion
					(23.50%) (102) ring D torsion

					(10.11%) (207) ring D-ethyl: WAGG
					(12.51%) (226) ring A-ethyl: 1-TORS
					(6.19%) (237) bridge C-D: TORS
231	90.64	-------	0.87	0.87	(16.89%) (94) ring B torsion
					(6.40%) (117) ring A: bridge AB WAGG
					(9.21%) (140) ring B-prop: 1-WAGG
					(6.72%) (148) ring B-prop: 1-BEND
					(7.08%) (229) ring B-prop: 1-TORS
					(5.66%) (233) bridge BC: 1-TORS
					(5.32%) (247) Cl-H stretch
232	80.26	-------	1.05	1.31	(13.33%) (116) ring A: bridge AB ROCK
					(5.88%) (135) ring B: bridge AB ROCK
					(15.26%) (168) bridge AB: BEND
					(11.91%) (226) ring A-ethyl: 1-TORS
					(5.23%) (235) bridge A-B: TORS
					(6.00%) (239) ring C-prop: 1-TORS
					(6.13%) (240) ring C-prop: 2-TORS
					(13.44%) (249) Cl OUT
233	76.25	-------	0.46	1.27	(5.52%) (103) ring D torsion
					(6.43%) (142) ring B: bridge BC WAGG
					(6.10%) (233) bridge BC: 1-TORS
					(22.52%) (239) ring C-prop: 1-TORS
234	69.01	-------	2.82	1.54	(12.91%) (229) ring B-prop: 1-TORS
					(8.39%) (234) bridge A=B: TORS
					(9.33%) (236) bridge BC: 2-TORS
					(6.71%) (237) bridge C-D: TORS
					(12.27%) (249) Cl OUT
235	61.49	-------	0.81	0.35	(5.68%) (102) ring D torsion
					(6.11%) (229) ring B-prop: 1-TORS
					(5.74%) (236) bridge BC: 2-TORS
					(16.40%) (240) ring C-prop: 2-TORS
					(10.44%) (249) Cl OUT
236	58.97	-------	0.02	3.45	(6.64%) (162) bridge BC: BEND
					(10.92%) (165) bridge CD: BEND
					(13.34%) (229) ring B-prop: 1-TORS
					(11.38%) (231) ring B-prop: 3-TORS
					(5.40%) (245) ring D-ethyl: 1-TORS
237	45.86	-------	4.04	0.60	(5.05%) (98) ring C torsion
					(9.03%) (229) ring B-prop: 1-TORS
					(32.98%) (230) ring B-prop: 2-TORS
					(11.41%) (231) ring B-prop: 3-TORS
					(12.46%) (241) ring C-prop: 3-TORS
238	44.33	-------	0.67	0.34	(6.25%) (180) ring C: bridge CD WAGG
					(10.06%) (230) ring B-prop: 2-TORS
					(36.60%) (231) ring B-prop: 3-TORS
					(13.33%) (241) ring C-prop: 3-TORS
239	41.47	-------	0.28	2.26	(5.12%) (241) ring C-prop: 3-TORS
					(5.56%) (243) bridge C=D: TORS
					(56.77%) (245) ring D-ethyl: 1-TORS
240	36.20	-------	0.94	0.91	(40.80%) (90) ring A torsion
					(7.23%) (234) bridge A=B: TORS
					(11.39%) (235) bridge A-B: TORS
					(5.12%) (241) ring C-prop: 3-TORS
					(9.79%) (245) ring D-ethyl: 1-TORS
241	29.89	-------	1.25	4.34	(11.28%) (90) ring A torsion
					(5.35%) (230) ring B-prop: 2-TORS
					(5.02%) (231) ring B-prop: 3-TORS
					(5.25%) (233) bridge BC: 1-TORS
					(10.11%) (237) bridge C-D: TORS
					(27.71%) (241) ring C-prop: 3-TORS
					(9.71%) (249) Cl OUT
242	28.63	-------	0.13	5.36	(8.36%) (90) ring A torsion

					(5.71%) (94) ring B torsion
					(10.17%) (136) ring B: bridge AB WAGG
					(7.57%) (140) ring B-prop: 1-WAGG
					(10.92%) (229) ring B-prop: 1-TORS
					(13.30%) (234) bridge A=B: TORS
					(7.53%) (239) ring C-prop: 1-TORS
					(5.17%) (241) ring C-prop: 3-TORS
243	26.79	-------	1.20	1.18	(6.22%) (140) ring B-prop: 1-WAGG
					(15.77%) (229) ring B-prop: 1-TORS
					(18.52%) (230) ring B-prop: 2-TORS
					(21.13%) (239) ring C-prop: 1-TORS
					(11.86%) (240) ring C-prop: 2-TORS
244	24.18	-------	0.26	2.42	(6.36%) (229) ring B-prop: 1-TORS
					(19.76%) (230) ring B-prop: 2-TORS
					(12.92%) (231) ring B-prop: 3-TORS
					(19.30%) (240) ring C-prop: 2-TORS
					(5.09%) (243) bridge C=D: TORS
					(6.91%) (245) ring D-ethyl: 1-TORS
245	22.01	-------	1.30	7.89	(5.55%) (103) ring D torsion
					(5.16%) (167) bridge CD: C-H OUT
					(6.26%) (230) ring B-prop: 2-TORS
					(14.48%) (237) bridge C-D: TORS
					(13.07%) (239) ring C-prop: 1-TORS
					(9.42%) (241) ring C-prop: 3-TORS
					(9.73%) (249) Cl OUT
246	19.72	-------	0.11	5.75	(8.03%) (162) bridge BC: BEND
					(11.35%) (165) bridge CD: BEND
					(9.64%) (179) ring C: bridge CD ROCK
					(6.21%) (240) ring C-prop: 2-TORS
					(9.37%) (245) ring D-ethyl: 1-TORS
247	15.37	-------	0.17	3.33	(21.31%) (90) ring A torsion
					(26.44%) (235) bridge A-B: TORS
					(8.47%) (236) bridge BC: 2-TORS
248	12.06	-------	0.92	38.32	(5.96%) (162) bridge BC: BEND
					(12.02%) (174) ring C: bridge BC WAGG
					(8.12%) (233) bridge BC: 1-TORS
					(28.93%) (237) bridge C-D: TORS
					(7.79%) (249) Cl OUT
249	10.62	-------	0.01	6.80	(5.28%) (90) ring A torsion
					(5.04%) (136) ring B: bridge AB WAGG
					(15.10%) (233) bridge BC: 1-TORS
					(5.24%) (235) bridge A-B: TORS
					(23.30%) (236) bridge BC: 2-TORS

ZZEssa, $^{13}C(15)$, D_2O

	calc. [cm-1]	exp. [cm-1]	IRint [km/mol]	Rint (rel.)	contribution
1	3522.77	-------	54.89	0.16	(100.07%) (67) ring C-prop: O-H stretch (27,84)
2	3516.59	-------	56.05	0.19	(100.07%) (39) ring B-prop: O-H stretch (85,83)
3	3067.40	-------	0.44	0.20	(99.76%) (42) bridge BC: C-H stretch (4, 3)
4	3056.72	-------	19.22	0.28	(99.85%) (46) bridge AB: C-H stretch (8, 7)
5	3013.49	-------	4.87	0.11	(50.73%) (56) ring C-methyl: C-H stretch (16,15)<br>(47.38%) (57) ring C-methyl: C-H stretch (17,15)
6	3012.89	-------	12.38	0.12	(79.95%) (78) ring D-methyl: C-H stretch (35,34)<br>(5.58%) (79) ring D-methyl: C-H stretch (36,34)<br>(12.82%) (80) ring D-methyl: C-H stretch (37,34)
7	3006.08	-------	18.93	0.08	(9.15%) (85) ring D-ethyl: C-H stretch (42,41)<br>(11.25%) (86) ring D-ethyl: C-H stretch (43,41)<br>(75.60%) (87) ring D-ethyl: C-H stretch (44,41)
8	3003.59	-------	11.25	0.10	(16.39%) (10) ring A-methyl: C-H stretch (55,54)<br>(6.14%) (11) ring A-methyl: C-H stretch (56,54)<br>(77.31%) (12) ring A-methyl: C-H stretch (57,54)
9	3002.86	-------	16.76	0.06	(99.77%) (43) bridge CD: C-H stretch (6, 5)
10	3000.91	-------	14.83	0.03	(6.78%) (60) ring C-prop: C-H stretch (20,19)<br>(71.57%) (61) ring C-prop: C-H stretch (21,19)<br>(19.65%) (63) ring C-prop: C-H stretch (23,22)
11	2998.99	-------	34.52	0.13	(13.33%) (27) ring B-methyl: C-H stretch (72,71)<br>(7.80%) (31) ring B-prop: C-H stretch (76,75)<br>(9.99%) (32) ring B-prop: C-H stretch (77,75)<br>(60.04%) (34) ring B-prop: C-H stretch (79,78)<br>(7.03%) (35) ring B-prop: C-H stretch (80,78)
12	2996.58	-------	11.92	0.12	(73.56%) (27) ring B-methyl: C-H stretch (72,71)<br>(5.57%) (29) ring B-methyl: C-H stretch (74,71)<br>(13.73%) (34) ring B-prop: C-H stretch (79,78)
13	2987.26	-------	28.02	0.19	(31.92%) (17) ring A-ethyl: C-H stretch (62,60)<br>(58.86%) (18) ring A-ethyl: C-H stretch (63,60)
14	2985.50	-------	50.72	0.46	(51.26%) (85) ring D-ethyl: C-H stretch (42,41)<br>(45.14%) (86) ring D-ethyl: C-H stretch (43,41)
15	2984.68	-------	1.10	0.07	(18.40%) (61) ring C-prop: C-H stretch (21,19)<br>(70.81%) (63) ring C-prop: C-H stretch (23,22)<br>(7.85%) (64) ring C-prop: C-H stretch (24,22)
16	2984.25	-------	45.06	0.18	(45.86%) (10) ring A-methyl: C-H stretch (55,54)<br>(43.19%) (11) ring A-methyl: C-H stretch (56,54)<br>(5.31%) (18) ring A-ethyl: C-H stretch (63,60)
17	2981.34	-------	8.86	0.07	(8.43%) (56) ring C-methyl: C-H stretch (16,15)<br>(10.04%) (57) ring C-methyl: C-H stretch (17,15)<br>(8.85%) (58) ring C-methyl: C-H stretch (18,15)<br>(36.13%) (79) ring D-methyl: C-H stretch (36,34)<br>(36.01%) (80) ring D-methyl: C-H stretch (37,34)
18	2979.96	-------	8.81	0.15	(27.76%) (56) ring C-methyl: C-H stretch (16,15)<br>(27.08%) (57) ring C-methyl: C-H stretch (17,15)<br>(17.98%) (58) ring C-methyl: C-H stretch (18,15)<br>(12.91%) (79) ring D-methyl: C-H stretch (36,34)<br>(13.86%) (80) ring D-methyl: C-H stretch (37,34)
19	2979.84	-------	36.02	0.08	(5.96%) (14) ring A-ethyl: C-H stretch (59,58)<br>(46.59%) (16) ring A-ethyl: C-H stretch (61,60)<br>(28.78%) (17) ring A-ethyl: C-H stretch (62,60)<br>(11.08%) (18) ring A-ethyl: C-H stretch (63,60)

					(6.25%) (40) ring A-ethyl: C-H stretch (1,58)
20	2977.85	-------	4.43	0.05	(46.44%) (31) ring B-prop: C-H stretch (76,75)
					(35.13%) (32) ring B-prop: C-H stretch (77,75)
					(16.51%) (34) ring B-prop: C-H stretch (79,78)
21	2956.12	-------	10.79	0.10	(56.79%) (82) ring D-ethyl: C-H stretch (39,38)
					(36.28%) (83) ring D-ethyl: C-H stretch (40,38)
22	2954.93	-------	14.72	0.18	(48.15%) (28) ring B-methyl: C-H stretch (73,71)
					(52.00%) (29) ring B-methyl: C-H stretch (74,71)
23	2949.88	-------	6.98	0.09	(52.31%) (14) ring A-ethyl: C-H stretch (59,58)
					(9.43%) (17) ring A-ethyl: C-H stretch (62,60)
					(28.60%) (40) ring A-ethyl: C-H stretch (1,58)
24	2947.13	-------	19.20	0.22	(43.74%) (31) ring B-prop: C-H stretch (76,75)
					(52.11%) (32) ring B-prop: C-H stretch (77,75)
25	2941.13	-------	8.18	0.22	(93.99%) (4) ring A: C-H stretch (50,49)
26	2936.45	-------	15.92	0.33	(75.58%) (60) ring C-prop: C-H stretch (20,19)
					(8.49%) (61) ring C-prop: C-H stretch (21,19)
					(13.81%) (64) ring C-prop: C-H stretch (24,22)
27	2930.97	-------	24.19	0.49	(17.31%) (78) ring D-methyl: C-H stretch (35,34)
					(43.16%) (79) ring D-methyl: C-H stretch (36,34)
					(36.55%) (80) ring D-methyl: C-H stretch (37,34)
28	2928.56	-------	2.42	0.15	(8.79%) (34) ring B-prop: C-H stretch (79,78)
					(87.43%) (35) ring B-prop: C-H stretch (80,78)
29	2925.03	-------	11.07	0.10	(14.89%) (60) ring C-prop: C-H stretch (20,19)
					(7.17%) (63) ring C-prop: C-H stretch (23,22)
					(76.98%) (64) ring C-prop: C-H stretch (24,22)
30	2922.01	-------	45.85	0.29	(38.41%) (85) ring D-ethyl: C-H stretch (42,41)
					(38.28%) (86) ring D-ethyl: C-H stretch (43,41)
					(19.06%) (87) ring D-ethyl: C-H stretch (44,41)
31	2921.79	-------	70.47	0.39	(29.50%) (6) ring A: C-H stretch (52,51)
					(15.90%) (10) ring A-methyl: C-H stretch (55,54)
					(23.86%) (11) ring A-methyl: C-H stretch (56,54)
					(10.92%) (12) ring A-methyl: C-H stretch (57,54)
					(9.71%) (14) ring A-ethyl: C-H stretch (59,58)
32	2919.15	-------	2.77	0.04	(16.39%) (6) ring A: C-H stretch (52,51)
					(15.15%) (10) ring A-methyl: C-H stretch (55,54)
					(21.22%) (11) ring A-methyl: C-H stretch (56,54)
					(8.71%) (12) ring A-methyl: C-H stretch (57,54)
					(15.18%) (14) ring A-ethyl: C-H stretch (59,58)
					(14.50%) (40) ring A-ethyl: C-H stretch (1,58)
33	2918.33	-------	21.76	0.25	(37.09%) (82) ring D-ethyl: C-H stretch (39,38)
					(57.06%) (83) ring D-ethyl: C-H stretch (40,38)
34	2917.21	-------	39.65	0.31	(11.68%) (56) ring C-methyl: C-H stretch (16,15)
					(13.90%) (57) ring C-methyl: C-H stretch (17,15)
					(70.91%) (58) ring C-methyl: C-H stretch (18,15)
35	2915.75	-------	27.24	0.12	(9.85%) (6) ring A: C-H stretch (52,51)
					(41.08%) (16) ring A-ethyl: C-H stretch (61,60)
					(26.18%) (17) ring A-ethyl: C-H stretch (62,60)
					(18.90%) (18) ring A-ethyl: C-H stretch (63,60)
36	2911.29	-------	6.32	0.21	(39.40%) (6) ring A: C-H stretch (52,51)
					(11.35%) (14) ring A-ethyl: C-H stretch (59,58)
					(44.72%) (40) ring A-ethyl: C-H stretch (1,58)
37	2909.72	-------	43.68	0.52	(12.35%) (27) ring B-methyl: C-H stretch (72,71)
					(45.88%) (28) ring B-methyl: C-H stretch (73,71)
					(41.11%) (29) ring B-methyl: C-H stretch (74,71)
38	2561.74	-------	38.36	0.15	(98.25%) (69) ring D: N-H stretch (29,28)

39	2302.25	-------	441.93	0.13	(34.77%) (1) ring A: N-H stretch (47,46)
					(33.04%) (20) ring B: N-H stretch (66,65)
					(29.51%) (47) ring C: N-H stretch (10, 9)
40	2244.28	-------	374.32	0.49	(58.27%) (1) ring A: N-H stretch (47,46)
					(6.23%) (20) ring B: N-H stretch (66,65)
					(33.06%) (47) ring C: N-H stretch (10, 9)
41	2232.12	-------	97.12	0.07	(59.08%) (20) ring B: N-H stretch (66,65)
					(35.55%) (47) ring C: N-H stretch (10, 9)
42	1779.83	-------	274.54	1.74	(85.35%) (19) ring A: C=O stretch (64,48)
43	1774.15	-------	188.97	0.05	(81.27%) (66) ring C-prop: C=O stretch (26,25)
					(6.41%) (199) ring C-prop: C-O-H BEND
44	1760.70	-------	254.49	0.02	(81.10%) (37) ring B-prop: C=O stretch (82,81)
					(6.53%) (161) ring B-prop: C-O-H BEND
45	1736.95	-------	884.37	4.71	(82.25%) (74) ring D: C=O stretch (33,45)
					(5.33%) (100) ring D bending
46	1613.62	-------	2.45	16.98	(69.45%) (73) ring D: C=C stretch (32,31)
					(6.25%) (77) ring D-methyl: C-C stretch (34,31)
					(8.03%) (81) ring D-ethyl: C-C stretch (38,32)
47	1610.21	-------	527.27	24.70	(20.21%) (41) bridge BC: C-.C stretch (3,70)
					(22.68%) (44) bridge AB: C=C stretch (7,53)
					(16.02%) (48) bridge BC: C-.C stretch (11, 3)
					(11.34%) (163) bridge BC: C-H ROCK
48	1590.70	-------	191.39	100.00	(6.47%) (52) bridge CD: C-C stretch (14, 5)
					(56.87%) (70) bridge CD: C=C stretch (30, 5)
					(6.24%) (71) ring D: C-N stretch (30,28)
					(9.36%) (166) bridge CD: C-H ROCK
49	1586.38	-------	1198.65	4.12	(23.77%) (23) ring B: C-.C stretch (69,68)
					(6.19%) (41) bridge BC: C-.C stretch (3,70)
					(16.93%) (44) bridge AB: C=C stretch (7,53)
					(8.04%) (48) bridge BC: C-.C stretch (11, 3)
					(7.31%) (163) bridge BC: C-H ROCK
					(5.24%) (169) bridge AB: C-H ROCK
50	1541.46	-------	1606.42	12.34	(7.17%) (21) ring B: C-N stretch (67,65)
					(26.18%) (23) ring B: C-.C stretch (69,68)
					(5.01%) (26) ring B-methyl: C-C stretch (71,68)
					(5.29%) (41) bridge BC: C-.C stretch (3,70)
					(10.92%) (44) bridge AB: C=C stretch (7,53)
					(9.68%) (45) bridge AB: C-C stretch (7,67)
					(7.09%) (92) ring B bending
51	1507.04	-------	153.62	2.25	(38.09%) (51) ring C: C-.C stretch (13,12)
					(6.28%) (54) ring C: C-C stretch (14,13)
					(10.23%) (55) ring C-methyl: C-C stretch (15,13)
52	1489.65	-------	65.77	9.69	(6.99%) (54) ring C: C-C stretch (14,13)
					(53.73%) (182) ring C-methyl: ADEFa
					(8.91%) (184) ring C-methyl: ROCKa
53	1486.85	-------	27.01	5.76	(21.66%) (7) ring A: C-N stretch (53,46)
					(10.69%) (22) ring B: C-C stretch (68,67)
					(5.18%) (44) bridge AB: C=C stretch (7,53)
					(13.13%) (45) bridge AB: C-C stretch (7,67)
					(16.81%) (169) bridge AB: C-H ROCK
54	1477.19	-------	7.99	0.25	(5.85%) (216) ring D-ethyl: CH2 SCIS
					(71.94%) (221) ring D-ethyl: CH3 ADEFa
					(10.28%) (222) ring D-ethyl: CH3 ADEFb
					(6.64%) (223) ring D-ethyl: CH3 ROCKa
55	1476.86	-------	79.12	0.29	(10.04%) (145) ring B-methyl: ADEFb
					(65.42%) (149) ring B-prop: 1-CH2-SCIS

56	1473.57	-------	5.12	0.05	(14.24%) (124) ring A-ethyl: CH2 SCIS
					(44.37%) (129) ring A-ethyl: CH3 ADEFa
					(25.76%) (130) ring A-ethyl: CH3 ADEFb
57	1468.65	-------	4.54	0.12	(20.84%) (119) ring A-methyl: ADEFa
					(38.98%) (129) ring A-ethyl: CH3 ADEFa
					(25.45%) (130) ring A-ethyl: CH3 ADEFb
58	1467.21	-------	10.96	0.16	(31.26%) (119) ring A-methyl: ADEFa
					(31.27%) (120) ring A-methyl: ADEFb
					(5.12%) (124) ring A-ethyl: CH2 SCIS
					(20.67%) (130) ring A-ethyl: CH3 ADEFb
59	1466.06	-------	2.20	2.15	(34.26%) (183) ring C-methyl: ADEFb
					(20.07%) (187) ring C-prop: 1-CH2-SCIS
					(14.22%) (212) ring D-methyl: ADEFb
60	1465.05	-------	4.57	0.13	(34.26%) (119) ring A-methyl: ADEFa
					(50.31%) (120) ring A-methyl: ADEFb
61	1463.95	-------	17.08	0.27	(6.40%) (221) ring D-ethyl: CH3 ADEFa
					(77.35%) (222) ring D-ethyl: CH3 ADEFb
					(7.17%) (224) ring D-ethyl: CH3 ROCKb
62	1462.73	-------	35.40	3.69	(8.82%) (144) ring B-methyl: ADEFa
					(6.89%) (145) ring B-methyl: ADEFb
					(7.83%) (149) ring B-prop: 1-CH2-SCIS
					(6.79%) (183) ring C-methyl: ADEFb
					(8.29%) (211) ring D-methyl: ADEFa
					(21.83%) (212) ring D-methyl: ADEFb
63	1458.18	-------	2.28	4.41	(29.97%) (144) ring B-methyl: ADEFa
					(16.99%) (145) ring B-methyl: ADEFb
					(5.94%) (187) ring C-prop: 1-CH2-SCIS
					(13.81%) (211) ring D-methyl: ADEFa
					(5.42%) (212) ring D-methyl: ADEFb
64	1456.57	-------	17.16	0.14	(17.26%) (187) ring C-prop: 1-CH2-SCIS
					(38.30%) (211) ring D-methyl: ADEFa
					(24.59%) (212) ring D-methyl: ADEFb
65	1454.48	-------	1.88	0.21	(71.89%) (124) ring A-ethyl: CH2 SCIS
					(14.83%) (130) ring A-ethyl: CH3 ADEFb
66	1453.24	-------	20.36	1.09	(31.49%) (144) ring B-methyl: ADEFa
					(31.11%) (145) ring B-methyl: ADEFb
					(11.06%) (183) ring C-methyl: ADEFb
67	1452.36	-------	21.60	0.34	(8.81%) (144) ring B-methyl: ADEFa
					(12.65%) (145) ring B-methyl: ADEFb
					(28.98%) (183) ring C-methyl: ADEFb
					(27.80%) (187) ring C-prop: 1-CH2-SCIS
					(7.23%) (212) ring D-methyl: ADEFb
68	1449.83	-------	80.65	6.94	(5.56%) (48) bridge BC: C-.C stretch (11, 3)
					(16.56%) (182) ring C-methyl: ADEFa
					(7.01%) (187) ring C-prop: 1-CH2-SCIS
					(23.47%) (211) ring D-methyl: ADEFa
69	1444.70	-------	1.97	0.19	(78.78%) (216) ring D-ethyl: CH2 SCIS
					(9.83%) (221) ring D-ethyl: CH3 ADEFa
70	1441.11	-------	17.20	10.66	(16.15%) (52) bridge CD: C-C stretch (14, 5)
					(6.86%) (53) ring C: C-N stretch (14, 9)
					(12.09%) (54) ring C: C-C stretch (14,13)
					(5.46%) (96) ring C bending
					(5.99%) (169) bridge AB: C-H ROCK
					(5.11%) (182) ring C-methyl: ADEFa
71	1438.19	-------	15.30	1.42	(81.84%) (154) ring B-prop: 2-CH2-SCIS
72	1435.63	-------	14.59	16.05	(19.16%) (21) ring B: C-N stretch (67,65)
					(7.07%) (23) ring B: C-.C stretch (69,68)
					(7.28%) (45) bridge AB: C-C stretch (7,67)

					(8.72%) (154) ring B-prop: 2-CH2-SCIS
					(18.56%) (169) bridge AB: C-H ROCK
73	1426.75	-------	148.25	1.15	(5.92%) (48) bridge BC: C-.C stretch (11, 3)
					(12.46%) (49) ring C: C-N stretch (11, 9)
					(6.70%) (97) ring C bending
					(14.66%) (163) bridge BC: C-H ROCK
					(20.60%) (192) ring C-prop: 2-CH2-SCIS
74	1423.14	-------	9.03	0.62	(7.87%) (163) bridge BC: C-H ROCK
					(64.07%) (192) ring C-prop: 2-CH2-SCIS
75	1410.53	-------	19.66	4.50	(7.34%) (24) ring B: C-N stretch (70,65)
					(5.06%) (25) ring B: C-C stretch (70,69)
					(7.86%) (93) ring B bending
					(6.67%) (97) ring C bending
					(5.02%) (143) ring B-methyl: SDEF
					(6.14%) (163) bridge BC: C-H ROCK
					(5.64%) (166) bridge CD: C-H ROCK
76	1398.91	-------	43.80	2.53	(7.51%) (65) ring C-prop: C-C stretch (25,22)
					(7.52%) (68) ring C-prop: C-O stretch (27,25)
					(5.57%) (192) ring C-prop: 2-CH2-SCIS
					(25.29%) (194) ring C-prop: 2-CH2-WAGG
					(6.96%) (197) ring C-prop: C=O ROCK
					(8.31%) (199) ring C-prop: C-O-H BEND
77	1398.62	-------	133.01	9.96	(10.39%) (143) ring B-methyl: SDEF
					(9.84%) (166) bridge CD: C-H ROCK
					(6.39%) (194) ring C-prop: 2-CH2-WAGG
78	1394.97	-------	106.39	0.44	(10.58%) (36) ring B-prop: C-C stretch (81,78)
					(13.33%) (38) ring B-prop: C-O stretch (83,81)
					(24.80%) (156) ring B-prop: 2-CH2-WAGG
					(11.53%) (159) ring B-prop: C=O ROCK
					(16.64%) (161) ring B-prop: C-O-H BEND
79	1389.58	-------	9.83	2.72	(5.43%) (77) ring D-methyl: C-C stretch (34,31)
					(67.24%) (210) ring D-methyl: SDEF
80	1387.33	-------	19.91	4.22	(5.61%) (166) bridge CD: C-H ROCK
					(76.68%) (181) ring C-methyl: SDEF
81	1384.18	-------	1.20	0.03	(92.05%) (128) ring A-ethyl: CH3 SDEF
82	1380.22	-------	21.97	15.36	(66.32%) (143) ring B-methyl: SDEF
83	1376.40	-------	1.53	6.59	(50.19%) (118) ring A-methyl: SDEF
					(5.34%) (166) bridge CD: C-H ROCK
					(5.66%) (181) ring C-methyl: SDEF
					(5.34%) (210) ring D-methyl: SDEF
84	1374.31	-------	24.30	4.75	(43.46%) (118) ring A-methyl: SDEF
					(5.89%) (210) ring D-methyl: SDEF
					(9.99%) (220) ring D-ethyl: CH3 SDEF
85	1368.71	-------	8.72	9.59	(5.30%) (166) bridge CD: C-H ROCK
					(73.71%) (220) ring D-ethyl: CH3 SDEF
86	1354.29	-------	10.71	0.43	(6.07%) (22) ring B: C-C stretch (68,67)
					(6.89%) (53) ring C: C-N stretch (14, 9)
					(11.75%) (126) ring A-ethyl: CH2 WAGG
					(9.21%) (189) ring C-prop: 1-CH2-WAGG
87	1352.20	-------	11.54	0.75	(15.70%) (112) ring A-ethyl: SCIS
					(46.47%) (126) ring A-ethyl: CH2 WAGG
88	1342.56	-------	14.76	1.01	(5.01%) (76) ring D: C-C stretch (33,32)
					(6.86%) (81) ring D-ethyl: C-C stretch (38,32)
					(5.23%) (101) ring D bending
					(19.69%) (189) ring C-prop: 1-CH2-WAGG
					(25.20%) (218) ring D-ethyl: CH2 WAGG
89	1340.62	-------	25.93	1.55	(14.24%) (108) ring A-methyl: SCIS

					(9.77%) (112) ring A-ethyl: SCIS
					(7.85%) (114) ring A-ethyl: WAGG
					(10.18%) (126) ring A-ethyl: CH2 WAGG
					(10.02%) (127) ring A-ethyl: CH2 TWIST
					(6.12%) (151) ring B-prop: 1-CH2-WAGG
					(5.32%) (189) ring C-prop: 1-CH2-WAGG
90	1337.34	-------	17.94	0.38	(45.66%) (151) ring B-prop: 1-CH2-WAGG
					(10.00%) (156) ring B-prop: 2-CH2-WAGG
					(9.79%) (161) ring B-prop: C-O-H BEND
91	1334.57	-------	30.86	0.74	(8.97%) (53) ring C: C-N stretch (14, 9)
					(35.18%) (189) ring C-prop: 1-CH2-WAGG
					(8.34%) (218) ring D-ethyl: CH2 WAGG
92	1317.83	-------	6.31	2.82	(10.07%) (76) ring D: C-C stretch (33,32)
					(7.73%) (101) ring D bending
					(41.12%) (218) ring D-ethyl: CH2 WAGG
					(10.33%) (219) ring D-ethyl: CH2 TWIST
93	1314.25	-------	25.45	0.04	(10.80%) (190) ring C-prop: 1-CH2-TWIST
					(23.88%) (194) ring C-prop: 2-CH2-WAGG
					(5.51%) (197) ring C-prop: C=O ROCK
					(38.11%) (199) ring C-prop: C-O-H BEND
94	1307.60	-------	15.23	0.06	(32.29%) (108) ring A-methyl: SCIS
					(5.76%) (109) ring A-methyl: ROCK
					(5.85%) (112) ring A-ethyl: SCIS
					(5.16%) (121) ring A-methyl: ROCKa
					(31.57%) (127) ring A-ethyl: CH2 TWIST
95	1293.98	-------	415.57	2.64	(18.56%) (152) ring B-prop: 1-CH2-TWIST
					(7.24%) (161) ring B-prop: C-O-H BEND
96	1290.74	-------	32.69	0.14	(26.24%) (110) ring A-methyl: WAGG
					(8.73%) (111) ring A-methyl: TWIST
					(10.47%) (114) ring A-ethyl: WAGG
					(5.52%) (115) ring A-ethyl: TWIST
					(5.41%) (126) ring A-ethyl: CH2 WAGG
97	1286.69	-------	208.59	1.74	(6.57%) (151) ring B-prop: 1-CH2-WAGG
					(12.60%) (156) ring B-prop: 2-CH2-WAGG
					(7.26%) (157) ring B-prop: 2-CH2-TWIST
					(14.05%) (161) ring B-prop: C-O-H BEND
98	1278.26	-------	2.61	0.15	(5.59%) (53) ring C: C-N stretch (14, 9)
					(42.07%) (219) ring D-ethyl: CH2 TWIST
					(10.36%) (224) ring D-ethyl: CH3 ROCKb
99	1260.57	-------	40.77	3.76	(24.97%) (71) ring D: C-N stretch (30,28)
					(6.95%) (166) bridge CD: C-H ROCK
					(6.32%) (208) ring D: C=O ROCK
					(12.93%) (219) ring D-ethyl: CH2 TWIST
					(5.08%) (224) ring D-ethyl: CH3 ROCKb
100	1256.64	-------	70.11	0.28	(7.28%) (108) ring A-methyl: SCIS
					(16.35%) (112) ring A-ethyl: SCIS
					(5.19%) (113) ring A-ethyl: ROCK
					(7.21%) (125) ring A-ethyl: CH2 ROCK
					(24.26%) (127) ring A-ethyl: CH2 TWIST
					(13.16%) (131) ring A-ethyl: CH3 ROCKa
101	1254.02	-------	23.91	0.70	(13.34%) (151) ring B-prop: 1-CH2-WAGG
					(20.29%) (152) ring B-prop: 1-CH2-TWIST
					(20.17%) (156) ring B-prop: 2-CH2-WAGG
					(12.77%) (157) ring B-prop: 2-CH2-TWIST
102	1242.34	-------	312.66	0.08	(11.45%) (110) ring A-methyl: WAGG
					(5.98%) (111) ring A-methyl: TWIST
					(7.01%) (112) ring A-ethyl: SCIS
					(11.14%) (114) ring A-ethyl: WAGG
					(7.12%) (115) ring A-ethyl: TWIST
					(13.40%) (190) ring C-prop: 1-CH2-TWIST

103	1237.07	-------	67.93	0.79	(5.38%)	(114)	ring A-ethyl: WAGG
					(23.92%)	(190)	ring C-prop: 1-CH2-TWIST
					(6.21%)	(194)	ring C-prop: 2-CH2-WAGG
					(11.29%)	(195)	ring C-prop: 2-CH2-TWIST
104	1226.08	-------	503.27	1.89	(12.51%)	(2)	ring A: C-N stretch (48,46)
					(6.14%)	(3)	ring A: C-C stretch (49,48)
					(6.89%)	(88)	ring A bending
					(5.29%)	(106)	ring A: C=O ROCK
					(6.36%)	(157)	ring B-prop: 2-CH2-TWIST
					(5.29%)	(169)	bridge AB: C-H ROCK
105	1216.82	-------	158.89	0.62	(16.88%)	(2)	ring A: C-N stretch (48,46)
					(14.49%)	(7)	ring A: C-N stretch (53,46)
					(5.09%)	(21)	ring B: C-N stretch (67,65)
					(11.66%)	(44)	bridge AB: C=C stretch (7,53)
					(10.73%)	(104)	ring A: N-H ROCK
					(15.38%)	(169)	bridge AB: C-H ROCK
106	1211.42	-------	100.18	2.61	(6.08%)	(55)	ring C-methyl: C-C stretch (15,13)
					(14.35%)	(75)	ring D: C-N stretch (33,28)
					(8.12%)	(166)	bridge CD: C-H ROCK
					(27.21%)	(195)	ring C-prop: 2-CH2-TWIST
107	1202.72	-------	82.42	0.38	(16.24%)	(152)	ring B-prop: 1-CH2-TWIST
					(20.92%)	(157)	ring B-prop: 2-CH2-TWIST
					(5.33%)	(161)	ring B-prop: C-O-H BEND
					(5.89%)	(190)	ring C-prop: 1-CH2-TWIST
					(14.19%)	(195)	ring C-prop: 2-CH2-TWIST
108	1191.68	-------	683.68	4.15	(5.70%)	(75)	ring D: C-N stretch (33,28)
					(5.11%)	(152)	ring B-prop: 1-CH2-TWIST
					(6.21%)	(190)	ring C-prop: 1-CH2-TWIST
					(9.32%)	(195)	ring C-prop: 2-CH2-TWIST
109	1160.45	-------	76.61	0.72	(13.47%)	(49)	ring C: C-N stretch (11, 9)
					(6.01%)	(55)	ring C-methyl: C-C stretch (15,13)
					(5.94%)	(59)	ring C-prop: C-C stretch (19,12)
					(15.54%)	(75)	ring D: C-N stretch (33,28)
					(7.17%)	(97)	ring C bending
110	1153.17	-------	184.65	0.07	(6.68%)	(38)	ring B-prop: C-O stretch (83,81)
					(18.36%)	(68)	ring C-prop: C-O stretch (27,25)
					(8.83%)	(199)	ring C-prop: C-O-H BEND
111	1150.81	-------	193.03	1.42	(19.06%)	(38)	ring B-prop: C-O stretch (83,81)
					(6.95%)	(157)	ring B-prop: 2-CH2-TWIST
					(8.44%)	(161)	ring B-prop: C-O-H BEND
112	1147.22	-------	150.21	0.03	(5.41%)	(38)	ring B-prop: C-O stretch (83,81)
					(11.88%)	(68)	ring C-prop: C-O stretch (27,25)
					(8.62%)	(125)	ring A-ethyl: CH2 ROCK
					(5.47%)	(199)	ring C-prop: C-O-H BEND
113	1138.87	-------	9.51	0.21	(5.10%)	(71)	ring D: C-N stretch (30,28)
					(14.19%)	(77)	ring D-methyl: C-C stretch (34,31)
					(12.64%)	(217)	ring D-ethyl: CH2 ROCK
					(10.25%)	(224)	ring D-ethyl: CH3 ROCKb
114	1137.64	-------	61.60	1.28	(16.15%)	(24)	ring B: C-N stretch (70,65)
					(6.84%)	(30)	ring B-prop: C-C stretch (75,69)
					(6.46%)	(133)	ring B: N-H ROCK
115	1127.22	-------	93.86	0.25	(6.21%)	(3)	ring A: C-C stretch (49,48)
					(9.78%)	(8)	ring A: C-C stretch (53,51)
					(12.68%)	(104)	ring A: N-H ROCK
					(5.44%)	(106)	ring A: C=O ROCK
					(6.32%)	(115)	ring A-ethyl: TWIST
116	1124.74	-------	96.12	0.11	(5.65%)	(59)	ring C-prop: C-C stretch (19,12)
					(5.63%)	(96)	ring C bending
					(20.44%)	(184)	ring C-methyl: ROCKa
117	1116.43	-------	79.04	0.24	(13.56%)	(81)	ring D-ethyl: C-C stretch (38,32)

					(14.08%) (213) ring D-methyl: ROCKa
					(12.04%) (214) ring D-methyl: ROCKb
					(11.72%) (217) ring D-ethyl: CH2 ROCK
					(11.01%) (224) ring D-ethyl: CH3 ROCKb
118	1097.88	-------	54.31	0.32	(5.39%) (26) ring B-methyl: C-C stretch (71,68)
					(5.83%) (92) ring B bending
					(5.57%) (104) ring A: N-H ROCK
					(11.14%) (132) ring A-ethyl: CH3 ROCKb
					(5.65%) (147) ring B-methyl: ROCKb
119	1093.90	-------	39.59	2.73	(6.01%) (5) ring A: C-C stretch (51,49)
					(7.67%) (13) ring A-ethyl: C-C stretch (58,51)
					(5.13%) (104) ring A: N-H ROCK
					(5.24%) (111) ring A-methyl: TWIST
120	1086.52	-------	21.68	1.15	(5.33%) (30) ring B-prop: C-C stretch (75,69)
					(8.58%) (122) ring A-methyl: ROCKb
					(9.27%) (133) ring B: N-H ROCK
					(5.51%) (147) ring B-methyl: ROCKb
					(6.33%) (184) ring C-methyl: ROCKa
121	1076.60	-------	4.32	2.02	(9.75%) (9) ring A-methyl: C-C stretch (54,49)
					(11.62%) (122) ring A-methyl: ROCKb
					(13.60%) (132) ring A-ethyl: CH3 ROCKb
122	1063.98	-------	60.13	1.33	(13.41%) (84) ring D-ethyl: C-C stretch (41,38)
					(12.72%) (213) ring D-methyl: ROCKa
					(9.27%) (215) ring D-ethyl: BEND
					(44.09%) (223) ring D-ethyl: CH3 ROCKa
123	1060.10	-------	0.71	6.54	(20.95%) (133) ring B: N-H ROCK
					(5.86%) (162) bridge BC: BEND
					(13.21%) (171) ring C: N-H ROCK
124	1053.07	-------	4.32	0.78	(7.01%) (178) ring C-methyl: WAGG
					(7.24%) (183) ring C-methyl: ADEFb
					(76.40%) (185) ring C-methyl: ROCKb
125	1046.87	-------	3.69	0.08	(8.42%) (138) ring B-methyl: WAGG
					(5.47%) (144) ring B-methyl: ADEFa
					(63.61%) (146) ring B-methyl: ROCKa
					(12.18%) (147) ring B-methyl: ROCKb
126	1045.57	-------	33.97	0.87	(11.64%) (147) ring B-methyl: ROCKb
					(10.21%) (150) ring B-prop: 1-CH2-ROCK
					(6.78%) (155) ring B-prop: 2-CH2-ROCK
					(10.27%) (188) ring C-prop: 1-CH2-ROCK
					(5.50%) (193) ring C-prop: 2-CH2-ROCK
127	1040.14	-------	0.72	0.52	(7.41%) (205) ring D-methyl: WAGG
					(22.04%) (213) ring D-methyl: ROCKa
					(51.82%) (214) ring D-methyl: ROCKb
					(6.95%) (223) ring D-ethyl: CH3 ROCKa
128	1026.43	-------	2.96	0.08	(7.92%) (13) ring A-ethyl: C-C stretch (58,51)
					(10.73%) (15) ring A-ethyl: C-C stretch (60,58)
					(8.15%) (109) ring A-methyl: ROCK
					(27.40%) (121) ring A-methyl: ROCKa
					(6.11%) (125) ring A-ethyl: CH2 ROCK
					(8.25%) (127) ring A-ethyl: CH2 TWIST
129	1024.13	-------	0.42	0.09	(5.42%) (5) ring A: C-C stretch (51,49)
					(6.70%) (9) ring A-methyl: C-C stretch (54,49)
					(11.65%) (13) ring A-ethyl: C-C stretch (58,51)
					(25.47%) (15) ring A-ethyl: C-C stretch (60,58)
					(6.16%) (112) ring A-ethyl: SCIS
					(14.97%) (122) ring A-methyl: ROCKb
					(8.65%) (131) ring A-ethyl: CH3 ROCKa
130	1019.20	-------	46.99	0.92	(6.33%) (150) ring B-prop: 1-CH2-ROCK
					(6.08%) (155) ring B-prop: 2-CH2-ROCK
					(27.36%) (171) ring C: N-H ROCK
					(7.75%) (193) ring C-prop: 2-CH2-ROCK

131	999.58	-------	17.78	2.35	(8.83%)	(71)	ring D: C-N stretch (30,28)
					(11.45%)	(76)	ring D: C-C stretch (33,32)
					(9.94%)	(77)	ring D-methyl: C-C stretch (34,31)
					(20.53%)	(200)	ring D: N-H ROCK
132	998.51	-------	87.97	0.24	(17.14%)	(9)	ring A-methyl: C-C stretch (54,49)
					(12.38%)	(15)	ring A-ethyl: C-C stretch (60,58)
					(6.42%)	(104)	ring A: N-H ROCK
					(5.13%)	(122)	ring A-methyl: ROCKb
133	997.66	-------	99.55	1.10	(37.78%)	(62)	ring C-prop: C-C stretch (22,19)
					(10.21%)	(184)	ring C-methyl: ROCKa
134	995.38	-------	18.03	0.14	(67.34%)	(33)	ring B-prop: C-C stretch (78,75)
					(6.06%)	(148)	ring B-prop: 1-BEND
					(5.43%)	(153)	ring B-prop: 2-BEND
					(5.14%)	(160)	ring B-prop: C=O OUT
135	984.33	-------	67.31	1.25	(5.11%)	(72)	ring D: C-C stretch (31,30)
					(7.44%)	(73)	ring D: C=C stretch (32,31)
					(5.38%)	(81)	ring D-ethyl: C-C stretch (38,32)
					(21.10%)	(84)	ring D-ethyl: C-C stretch (41,38)
					(5.43%)	(200)	ring D: N-H ROCK
					(16.67%)	(213)	ring D-methyl: ROCKa
					(7.76%)	(214)	ring D-methyl: ROCKb
					(6.27%)	(224)	ring D-ethyl: CH3 ROCKb
136	952.60	-------	143.36	0.07	(5.92%)	(22)	ring B: C-C stretch (68,67)
					(7.61%)	(23)	ring B: C-.C stretch (69,68)
					(15.40%)	(147)	ring B-methyl: ROCKb
					(9.80%)	(150)	ring B-prop: 1-CH2-ROCK
					(8.65%)	(155)	ring B-prop: 2-CH2-ROCK
137	947.72	-------	44.64	0.63	(6.52%)	(186)	ring C-prop: 1-BEND
					(13.17%)	(188)	ring C-prop: 1-CH2-ROCK
					(7.44%)	(191)	ring C-prop: 2-BEND
					(21.30%)	(193)	ring C-prop: 2-CH2-ROCK
					(12.75%)	(198)	ring C-prop: C=O OUT
138	925.65	-------	2.60	1.35	(5.04%)	(5)	ring A: C-C stretch (51,49)
					(7.34%)	(15)	ring A-ethyl: C-C stretch (60,58)
					(12.18%)	(84)	ring D-ethyl: C-C stretch (41,38)
					(7.19%)	(132)	ring A-ethyl: CH3 ROCKb
					(7.16%)	(193)	ring C-prop: 2-CH2-ROCK
139	924.19	-------	39.32	0.53	(5.44%)	(5)	ring A: C-C stretch (51,49)
					(8.27%)	(15)	ring A-ethyl: C-C stretch (60,58)
					(29.16%)	(84)	ring D-ethyl: C-C stretch (41,38)
					(7.74%)	(132)	ring A-ethyl: CH3 ROCKb
					(8.36%)	(223)	ring D-ethyl: CH3 ROCKa
140	918.46	-------	332.31	0.31	(5.91%)	(65)	ring C-prop: C-C stretch (25,22)
					(8.55%)	(84)	ring D-ethyl: C-C stretch (41,38)
					(6.86%)	(188)	ring C-prop: 1-CH2-ROCK
					(7.58%)	(193)	ring C-prop: 2-CH2-ROCK
141	908.52	-------	41.52	0.50	(21.74%)	(36)	ring B-prop: C-C stretch (81,78)
					(6.79%)	(38)	ring B-prop: C-O stretch (83,81)
					(5.50%)	(148)	ring B-prop: 1-BEND
					(6.72%)	(153)	ring B-prop: 2-BEND
					(12.08%)	(155)	ring B-prop: 2-CH2-ROCK
					(9.89%)	(160)	ring B-prop: C=O OUT
142	886.66	-------	28.24	0.41	(5.43%)	(5)	ring A: C-C stretch (51,49)
					(7.43%)	(7)	ring A: C-N stretch (53,46)
					(14.37%)	(8)	ring A: C-C stretch (53,51)
					(6.64%)	(13)	ring A-ethyl: C-C stretch (58,51)
					(7.00%)	(15)	ring A-ethyl: C-C stretch (60,58)
					(6.74%)	(104)	ring A: N-H ROCK
					(10.57%)	(164)	bridge BC: C-H OUT
143	885.39	-------	11.79	0.83	(58.00%)	(164)	bridge BC: C-H OUT
					(9.95%)	(233)	bridge BC: 1-TORS

					(7.37%) (236) bridge BC: 2-TORS
144	853.44	-------	9.73	0.20	(5.81%) (7) ring A: C-N stretch (53,46)
					(5.60%) (36) ring B-prop: C-C stretch (81,78)
					(7.45%) (104) ring A: N-H ROCK
					(11.24%) (133) ring B: N-H ROCK
145	850.14	-------	44.45	0.12	(5.75%) (113) ring A-ethyl: ROCK
					(16.43%) (131) ring A-ethyl: CH3 ROCKa
146	836.83	-------	49.11	2.83	(9.56%) (54) ring C: C-C stretch (14,13)
					(12.55%) (171) ring C: N-H ROCK
					(5.62%) (200) ring D: N-H ROCK
147	813.60	-------	31.57	0.16	(10.28%) (72) ring D: C-C stretch (31,30)
					(5.11%) (206) ring D-ethyl: ROCK
					(5.12%) (213) ring D-methyl: ROCKa
					(12.74%) (217) ring D-ethyl: CH2 ROCK
					(17.23%) (224) ring D-ethyl: CH3 ROCKb
148	810.69	-------	25.89	56.50	(57.42%) (167) bridge CD: C-H OUT
					(9.67%) (203) ring D: bridge CD WAGG
					(12.30%) (243) bridge C=D: TORS
149	800.57	-------	6.28	3.03	(9.02%) (36) ring B-prop: C-C stretch (81,78)
					(8.90%) (65) ring C-prop: C-C stretch (25,22)
					(8.80%) (150) ring B-prop: 1-CH2-ROCK
					(6.67%) (155) ring B-prop: 2-CH2-ROCK
150	798.00	-------	14.93	0.15	(5.38%) (136) ring B: bridge AB WAGG
					(58.39%) (170) bridge AB: C-H OUT
					(12.04%) (234) bridge A=B: TORS
					(7.28%) (235) bridge A-B: TORS
151	794.89	-------	3.04	0.26	(6.80%) (2) ring A: C-N stretch (48,46)
					(22.75%) (3) ring A: C-C stretch (49,48)
					(9.34%) (107) ring A: C=O OUT
					(6.83%) (121) ring A-methyl: ROCKa
					(6.55%) (122) ring A-methyl: ROCKb
					(5.01%) (170) bridge AB: C-H OUT
152	787.98	-------	6.09	0.64	(5.03%) (36) ring B-prop: C-C stretch (81,78)
					(21.98%) (65) ring C-prop: C-C stretch (25,22)
					(9.43%) (68) ring C-prop: C-O stretch (27,25)
					(6.31%) (150) ring B-prop: 1-CH2-ROCK
					(10.04%) (188) ring C-prop: 1-CH2-ROCK
153	774.26	-------	12.73	0.35	(8.22%) (102) ring D torsion
					(5.01%) (165) bridge CD: BEND
					(8.10%) (207) ring D-ethyl: WAGG
					(32.41%) (209) ring D: C=O OUT
154	769.34	-------	2.40	0.21	(5.35%) (162) bridge BC: BEND
					(18.64%) (209) ring D: C=O OUT
155	763.47	-------	34.30	1.62	(11.05%) (96) ring C bending
					(10.07%) (125) ring A-ethyl: CH2 ROCK
					(7.03%) (150) ring B-prop: 1-CH2-ROCK
156	755.71	-------	11.21	0.63	(5.03%) (5) ring A: C-C stretch (51,49)
					(25.48%) (125) ring A-ethyl: CH2 ROCK
					(5.05%) (131) ring A-ethyl: CH3 ROCKa
					(6.27%) (132) ring A-ethyl: CH3 ROCKb
157	748.59	-------	8.93	0.29	(7.29%) (76) ring D: C-C stretch (33,32)
					(8.04%) (100) ring D bending
					(5.10%) (180) ring C: bridge CD WAGG
					(25.87%) (217) ring D-ethyl: CH2 ROCK
					(11.87%) (224) ring D-ethyl: CH3 ROCKb
158	736.25	-------	11.32	0.27	(7.09%) (88) ring A bending
					(5.55%) (217) ring D-ethyl: CH2 ROCK
159	730.86	-------	17.05	0.47	(14.38%) (94) ring B torsion

					(12.98%) (140) ring B-prop: 1-WAGG
					(8.48%) (142) ring B: bridge BC WAGG
					(5.78%) (148) ring B-prop: 1-BEND
					(7.47%) (160) ring B-prop: C=O OUT
					(7.46%) (232) ring B-prop: 4-TORS
160	718.96	-------	18.81	0.36	(8.71%) (98) ring C torsion
					(7.53%) (174) ring C: bridge BC WAGG
					(8.48%) (176) ring C-prop: 1-WAGG
161	713.65	-------	30.63	13.19	(5.10%) (75) ring D: C-N stretch (33,28)
					(6.01%) (92) ring B bending
					(9.14%) (96) ring C bending
					(27.17%) (100) ring D bending
162	706.48	-------	4.55	0.39	(14.38%) (95) ring B torsion
					(27.19%) (136) ring B: bridge AB WAGG
					(8.21%) (232) ring B-prop: 4-TORS
					(7.44%) (234) bridge A=B: TORS
163	697.86	-------	119.78	0.42	(5.35%) (92) ring B bending
					(5.88%) (150) ring B-prop: 1-CH2-ROCK
					(10.21%) (155) ring B-prop: 2-CH2-ROCK
					(7.18%) (160) ring B-prop: C=O OUT
					(35.40%) (232) ring B-prop: 4-TORS
164	690.62	-------	39.35	0.71	(8.98%) (2) ring A: C-N stretch (48,46)
					(6.85%) (9) ring A-methyl: C-C stretch (54,49)
					(7.44%) (92) ring B bending
					(24.18%) (107) ring A: C=O OUT
165	684.33	-------	46.20	2.77	(9.28%) (98) ring C torsion
					(5.04%) (193) ring C-prop: 2-CH2-ROCK
					(9.78%) (198) ring C-prop: C=O OUT
					(41.77%) (242) ring C-prop: 4-TORS
166	678.58	-------	28.30	0.56	(5.76%) (55) ring C-methyl: C-C stretch (15,13)
					(8.83%) (107) ring A: C=O OUT
					(9.31%) (242) ring C-prop: 4-TORS
167	672.11	-------	43.75	0.28	(5.59%) (95) ring B torsion
					(5.23%) (142) ring B: bridge BC WAGG
					(5.47%) (232) ring B-prop: 4-TORS
					(9.69%) (242) ring C-prop: 4-TORS
168	667.92	-------	5.95	1.62	(10.01%) (55) ring C-methyl: C-C stretch (15,13)
					(5.92%) (81) ring D-ethyl: C-C stretch (38,32)
					(15.82%) (99) ring C torsion
					(9.64%) (100) ring D bending
					(6.60%) (174) ring C: bridge BC WAGG
169	661.07	-------	5.46	0.26	(5.69%) (8) ring A: C-C stretch (53,51)
					(9.93%) (88) ring A bending
					(31.50%) (117) ring A: bridge AB WAGG
170	651.42	-------	32.95	29.79	(7.84%) (102) ring D torsion
					(15.93%) (103) ring D torsion
					(26.22%) (203) ring D: bridge CD WAGG
					(14.08%) (205) ring D-methyl: WAGG
					(10.42%) (209) ring D: C=O OUT
					(5.66%) (243) bridge C=D: TORS
171	643.44	-------	2.62	3.15	(6.14%) (72) ring D: C-C stretch (31,30)
					(6.72%) (75) ring D: C-N stretch (33,28)
					(5.24%) (76) ring D: C-C stretch (33,32)
					(9.34%) (81) ring D-ethyl: C-C stretch (38,32)
					(5.29%) (99) ring C torsion
					(6.52%) (203) ring D: bridge CD WAGG
					(7.47%) (208) ring D: C=O ROCK
172	629.84	-------	22.25	0.81	(11.16%) (26) ring B-methyl: C-C stretch (71,68)
					(8.76%) (93) ring B bending
					(18.94%) (159) ring B-prop: C=O ROCK
					(6.83%) (232) ring B-prop: 4-TORS

173	617.75	-------	46.33	0.40	(9.16%) (159) ring B-prop: C=O ROCK
					(19.48%) (197) ring C-prop: C=O ROCK
					(8.42%) (242) ring C-prop: 4-TORS
174	610.22	-------	17.11	0.26	(11.18%) (88) ring A bending
					(32.14%) (89) ring A bending
					(11.73%) (117) ring A: bridge AB WAGG
175	604.75	-------	12.94	1.05	(8.21%) (93) ring B bending
					(7.42%) (159) ring B-prop: C=O ROCK
					(21.72%) (197) ring C-prop: C=O ROCK
176	593.58	-------	18.49	0.27	(7.30%) (26) ring B-methyl: C-C stretch (71,68)
					(8.05%) (55) ring C-methyl: C-C stretch (15,13)
					(6.02%) (59) ring C-prop: C-C stretch (19,12)
					(12.32%) (93) ring B bending
					(18.38%) (97) ring C bending
177	567.67	-------	4.40	0.12	(6.56%) (77) ring D-methyl: C-C stretch (34,31)
					(5.67%) (101) ring D bending
					(8.97%) (105) ring A: N-H OUT
					(6.81%) (134) ring B: N-H OUT
178	565.69	-------	90.32	0.05	(42.25%) (105) ring A: N-H OUT
					(12.97%) (134) ring B: N-H OUT
					(7.74%) (172) ring C: N-H OUT
179	559.54	-------	14.72	0.19	(5.71%) (77) ring D-methyl: C-C stretch (34,31)
					(8.52%) (101) ring D bending
					(5.77%) (148) ring B-prop: 1-BEND
					(7.17%) (159) ring B-prop: C=O ROCK
					(12.72%) (160) ring B-prop: C=O OUT
					(9.70%) (232) ring B-prop: 4-TORS
180	548.83	-------	7.29	1.33	(18.15%) (101) ring D bending
					(6.07%) (179) ring C: bridge CD ROCK
					(9.40%) (208) ring D: C=O ROCK
181	531.16	-------	24.09	1.63	(6.75%) (105) ring A: N-H OUT
					(11.63%) (106) ring A: C=O ROCK
					(18.53%) (134) ring B: N-H OUT
					(25.89%) (172) ring C: N-H OUT
182	520.61	-------	16.64	0.30	(5.10%) (134) ring B: N-H OUT
					(5.10%) (174) ring C: bridge BC WAGG
					(7.78%) (196) ring C-prop: 3-BEND
183	512.99	-------	9.82	1.91	(14.69%) (105) ring A: N-H OUT
					(17.93%) (172) ring C: N-H OUT
					(5.39%) (193) ring C-prop: 2-CH2-ROCK
					(11.74%) (198) ring C-prop: C=O OUT
					(5.07%) (242) ring C-prop: 4-TORS
184	504.17	-------	26.68	0.02	(9.51%) (105) ring A: N-H OUT
					(8.45%) (106) ring A: C=O ROCK
					(6.83%) (172) ring C: N-H OUT
					(6.00%) (193) ring C-prop: 2-CH2-ROCK
					(8.21%) (198) ring C-prop: C=O OUT
185	501.18	-------	6.30	1.00	(58.75%) (134) ring B: N-H OUT
					(36.60%) (172) ring C: N-H OUT
186	497.78	-------	12.22	4.10	(10.64%) (106) ring A: C=O ROCK
					(8.24%) (137) ring B-methyl: ROCK
					(5.61%) (196) ring C-prop: 3-BEND
187	492.11	-------	8.20	9.42	(9.02%) (101) ring D bending
					(6.42%) (203) ring D: bridge CD WAGG
					(6.14%) (205) ring D-methyl: WAGG
					(16.37%) (207) ring D-ethyl: WAGG
					(8.87%) (209) ring D: C=O OUT
					(7.25%) (215) ring D-ethyl: BEND

188	487.40	-------	8.57	0.88	(12.82%) (196) ring C-prop: 3-BEND
					(12.79%) (198) ring C-prop: C=O OUT
189	479.00	-------	8.07	2.27	(5.67%) (142) ring B: bridge BC WAGG
190	439.53	-------	2.25	0.18	(6.42%) (5) ring A: C-C stretch (51,49)
					(5.25%) (9) ring A-methyl: C-C stretch (54,49)
					(5.49%) (13) ring A-ethyl: C-C stretch (58,51)
					(9.76%) (109) ring A-methyl: ROCK
					(10.08%) (113) ring A-ethyl: ROCK
					(5.38%) (123) ring A-ethyl: BEND
191	430.55	-------	11.77	0.36	(6.85%) (155) ring B-prop: 2-CH2-ROCK
					(29.42%) (158) ring B-prop: 3-BEND
					(5.69%) (196) ring C-prop: 3-BEND
192	403.83	-------	7.63	0.90	(5.42%) (123) ring A-ethyl: BEND
					(5.44%) (136) ring B: bridge AB WAGG
					(7.38%) (138) ring B-methyl: WAGG
					(11.61%) (158) ring B-prop: 3-BEND
					(6.44%) (174) ring C: bridge BC WAGG
					(5.32%) (180) ring C: bridge CD WAGG
					(5.29%) (196) ring C-prop: 3-BEND
193	398.85	-------	6.47	0.09	(5.29%) (88) ring A bending
					(8.11%) (106) ring A: C=O ROCK
					(16.66%) (123) ring A-ethyl: BEND
194	363.15	-------	10.07	0.56	(8.19%) (103) ring D torsion
					(5.11%) (178) ring C-methyl: WAGG
					(8.11%) (179) ring C: bridge CD ROCK
					(10.73%) (205) ring D-methyl: WAGG
					(8.52%) (215) ring D-ethyl: BEND
195	349.91	-------	1.15	1.54	(6.35%) (175) ring C-prop: 1-ROCK
					(27.51%) (178) ring C-methyl: WAGG
					(8.90%) (186) ring C-prop: 1-BEND
					(14.25%) (196) ring C-prop: 3-BEND
					(6.17%) (205) ring D-methyl: WAGG
196	343.71	-------	2.40	1.14	(5.33%) (123) ring A-ethyl: BEND
					(12.21%) (137) ring B-methyl: ROCK
					(5.58%) (138) ring B-methyl: WAGG
					(20.84%) (139) ring B-prop: 1-ROCK
					(5.79%) (153) ring B-prop: 2-BEND
					(9.24%) (201) ring D: N-H OUT
197	334.70	-------	36.22	0.33	(-16.84%) (103) ring D torsion
					(77.17%) (201) ring D: N-H OUT
198	325.42	-------	2.53	3.64	(8.45%) (137) ring B-methyl: ROCK
					(27.55%) (138) ring B-methyl: WAGG
					(7.58%) (139) ring B-prop: 1-ROCK
					(5.29%) (140) ring B-prop: 1-WAGG
					(11.33%) (158) ring B-prop: 3-BEND
199	318.15	-------	0.11	21.59	(11.38%) (175) ring C-prop: 1-ROCK
					(10.41%) (201) ring D: N-H OUT
					(8.23%) (203) ring D: bridge CD WAGG
					(18.40%) (205) ring D-methyl: WAGG
					(9.21%) (215) ring D-ethyl: BEND
200	317.04	-------	3.43	1.96	(5.06%) (76) ring D: C-C stretch (33,32)
					(43.11%) (204) ring D-methyl: ROCK
					(9.14%) (208) ring D: C=O ROCK
201	303.51	-------	3.09	5.43	(11.58%) (95) ring B torsion
					(19.70%) (177) ring C-methyl: ROCK
					(5.51%) (206) ring D-ethyl: ROCK
					(11.74%) (243) bridge C=D: TORS
					(6.74%) (246) ring D-ethyl: 2-TORS
202	294.08	-------	0.53	0.84	(13.48%) (178) ring C-methyl: WAGG
					(15.74%) (206) ring D-ethyl: ROCK

					(28.81%) (246) ring D-ethyl: 2-TORS
203	291.35	-------	2.90	9.05	(5.10%) (97) ring C bending
					(5.76%) (175) ring C-prop: 1-ROCK
					(7.46%) (177) ring C-methyl: ROCK
					(5.11%) (191) ring C-prop: 2-BEND
					(7.88%) (204) ring D-methyl: ROCK
204	286.45	-------	2.70	12.56	(5.52%) (99) ring C torsion
					(7.34%) (123) ring A-ethyl: BEND
					(6.05%) (206) ring D-ethyl: ROCK
					(7.62%) (246) ring D-ethyl: 2-TORS
205	279.39	-------	2.06	6.93	(5.22%) (111) ring A-methyl: TWIST
					(13.13%) (123) ring A-ethyl: BEND
					(5.10%) (137) ring B-methyl: ROCK
					(5.05%) (227) ring A-ethyl: 2-TORS
206	269.27	-------	0.60	0.13	(5.00%) (173) ring C: bridge BC ROCK
					(7.67%) (177) ring C-methyl: ROCK
					(22.24%) (227) ring A-ethyl: 2-TORS
207	255.98	-------	2.52	0.64	(9.28%) (109) ring A-methyl: ROCK
					(8.91%) (225) ring A-methyl: TORS
					(23.47%) (227) ring A-ethyl: 2-TORS
208	247.80	-------	1.65	0.30	(6.67%) (110) ring A-methyl: WAGG
					(7.30%) (137) ring B-methyl: ROCK
					(6.34%) (138) ring B-methyl: WAGG
					(23.18%) (225) ring A-methyl: TORS
209	237.36	-------	0.36	0.15	(11.40%) (111) ring A-methyl: TWIST
					(60.54%) (225) ring A-methyl: TORS
210	233.28	-------	2.29	0.71	(6.76%) (202) ring D: bridge CD ROCK
					(11.31%) (227) ring A-ethyl: 2-TORS
					(5.99%) (234) bridge A=B: TORS
211	224.38	-------	2.32	0.90	(7.04%) (135) ring B: bridge AB ROCK
					(5.34%) (137) ring B-methyl: ROCK
					(5.01%) (202) ring D: bridge CD ROCK
					(5.07%) (227) ring A-ethyl: 2-TORS
					(39.64%) (238) ring C-methyl: TORS
212	219.79	-------	1.00	0.95	(33.97%) (238) ring C-methyl: TORS
					(7.91%) (244) ring D-methyl: TORS
					(8.82%) (246) ring D-ethyl: 2-TORS
213	215.77	-------	1.16	0.63	(9.25%) (135) ring B: bridge AB ROCK
					(9.87%) (137) ring B-methyl: ROCK
					(10.37%) (153) ring B-prop: 2-BEND
					(5.76%) (206) ring D-ethyl: ROCK
					(12.57%) (246) ring D-ethyl: 2-TORS
214	208.84	-------	2.56	0.77	(6.39%) (176) ring C-prop: 1-WAGG
					(9.54%) (206) ring D-ethyl: ROCK
					(13.62%) (246) ring D-ethyl: 2-TORS
215	196.54	-------	4.30	2.13	(7.93%) (91) ring A torsion
					(7.30%) (141) ring B: bridge BC ROCK
					(6.64%) (235) bridge A-B: TORS
					(5.43%) (246) ring D-ethyl: 2-TORS
					(10.91%) (248) Cl-H stretch
216	188.01	-------	1.68	3.23	(8.18%) (153) ring B-prop: 2-BEND
					(6.18%) (186) ring C-prop: 1-BEND
					(5.86%) (227) ring A-ethyl: 2-TORS
217	184.13	-------	2.61	2.38	(8.63%) (102) ring D torsion
					(7.29%) (205) ring D-methyl: WAGG
					(6.34%) (207) ring D-ethyl: WAGG
					(8.24%) (215) ring D-ethyl: BEND
					(6.60%) (237) bridge C-D: TORS
					(17.92%) (248) Cl-H stretch

```
218    179.17   -------     3.58     0.29   ( 11.76%) ( 91) ring A torsion
                                            (  5.45%) (123) ring A-ethyl: BEND
                                            (  5.13%) (247) Cl-H stretch

219    168.32   -------     4.01     0.32   (  8.68%) ( 91) ring A torsion
                                            (  6.56%) ( 95) ring B torsion
                                            (  5.32%) (109) ring A-methyl: ROCK
                                            (  7.02%) (162) bridge BC: BEND
                                            ( 14.01%) (235) bridge A-B: TORS
                                            (  5.29%) (247) Cl-H stretch
                                            ( 16.20%) (248) Cl-H stretch

220    166.53   -------     5.78     1.74   (  6.76%) ( 91) ring A torsion
                                            (  5.48%) (102) ring D torsion
                                            (  5.16%) (205) ring D-methyl: WAGG
                                            (  6.52%) (207) ring D-ethyl: WAGG
                                            (  7.09%) (215) ring D-ethyl: BEND
                                            ( 32.14%) (248) Cl-H stretch

221    164.28   -------     0.15     1.69   (  6.18%) ( 98) ring C torsion
                                            (  5.94%) (202) ring D: bridge CD ROCK
                                            (  7.81%) (207) ring D-ethyl: WAGG
                                            (  5.24%) (215) ring D-ethyl: BEND
                                            ( 15.49%) (237) bridge C-D: TORS
                                            (  6.61%) (238) ring C-methyl: TORS
                                            (  7.39%) (243) bridge C=D: TORS
                                            (  9.40%) (244) ring D-methyl: TORS

222    150.90   -------     0.99     7.30   (  8.48%) (103) ring D torsion
                                            (  8.90%) (153) ring B-prop: 2-BEND
                                            (  5.39%) (237) bridge C-D: TORS
                                            ( 13.79%) (247) Cl-H stretch

223    146.75   -------     5.22     0.45   ( 12.82%) ( 91) ring A torsion
                                            (  5.22%) (114) ring A-ethyl: WAGG
                                            (  7.13%) (115) ring A-ethyl: TWIST
                                            ( -6.18%) (235) bridge A-B: TORS
                                            ( 30.61%) (247) Cl-H stretch
                                            (  8.30%) (249) Cl OUT

224    140.62   -------     1.01     1.22   (  6.02%) ( 91) ring A torsion
                                            ( 11.76%) (103) ring D torsion
                                            (  5.77%) (141) ring B: bridge BC ROCK
                                            ( 21.99%) (228) ring B-methyl: TORS

225    137.51   -------     3.31     0.22   ( 13.56%) (103) ring D torsion
                                            ( 17.17%) (228) ring B-methyl: TORS
                                            ( 10.89%) (244) ring D-methyl: TORS
                                            (  5.68%) (247) Cl-H stretch

226    131.62   -------     0.97     0.47   (  5.10%) ( 91) ring A torsion
                                            (  5.17%) (226) ring A-ethyl: 1-TORS
                                            ( 49.23%) (228) ring B-methyl: TORS
                                            ( 12.68%) (244) ring D-methyl: TORS

227    126.90   -------     0.53     0.32   (  5.88%) (116) ring A: bridge AB ROCK
                                            (  5.42%) (168) bridge AB: BEND
                                            (  6.41%) (175) ring C-prop: 1-ROCK
                                            ( 17.00%) (226) ring A-ethyl: 1-TORS
                                            (  6.15%) (247) Cl-H stretch

228    121.87   -------     0.80     5.08   (  7.60%) (174) ring C: bridge BC WAGG
                                            ( 36.61%) (244) ring D-methyl: TORS

229    104.24   -------     1.01     1.32   (  6.51%) (173) ring C: bridge BC ROCK
                                            ( 32.93%) (226) ring A-ethyl: 1-TORS

230    101.30   -------     0.31     7.10   (  5.80%) ( 98) ring C torsion
                                            ( 23.51%) (102) ring D torsion
                                            ( 10.21%) (207) ring D-ethyl: WAGG
                                            ( 12.15%) (226) ring A-ethyl: 1-TORS
                                            (  6.20%) (237) bridge C-D: TORS
```

231	90.42	-------	0.88	0.76	(16.96%) (94) ring B torsion
					(6.31%) (117) ring A: bridge AB WAGG
					(9.24%) (140) ring B-prop: 1-WAGG
					(6.73%) (148) ring B-prop: 1-BEND
					(7.13%) (229) ring B-prop: 1-TORS
					(5.74%) (233) bridge BC: 1-TORS
					(5.23%) (247) Cl-H stretch
232	80.04	-------	1.07	1.14	(5.06%) (103) ring D torsion
					(13.36%) (116) ring A: bridge AB ROCK
					(5.74%) (135) ring B: bridge AB ROCK
					(15.17%) (168) bridge AB: BEND
					(11.53%) (226) ring A-ethyl: 1-TORS
					(5.21%) (235) bridge A-B: TORS
					(6.38%) (239) ring C-prop: 1-TORS
					(6.42%) (240) ring C-prop: 2-TORS
					(13.44%) (249) Cl OUT
233	76.16	-------	0.45	1.17	(5.46%) (103) ring D torsion
					(6.45%) (142) ring B: bridge BC WAGG
					(6.23%) (233) bridge BC: 1-TORS
					(22.11%) (239) ring C-prop: 1-TORS
234	68.83	-------	2.79	1.43	(13.39%) (229) ring B-prop: 1-TORS
					(8.56%) (234) bridge A=B: TORS
					(9.20%) (236) bridge BC: 2-TORS
					(6.52%) (237) bridge C-D: TORS
					(12.54%) (249) Cl OUT
235	61.27	-------	0.82	0.31	(5.63%) (102) ring D torsion
					(5.65%) (229) ring B-prop: 1-TORS
					(6.09%) (236) bridge BC: 2-TORS
					(16.62%) (240) ring C-prop: 2-TORS
					(10.18%) (249) Cl OUT
236	58.87	-------	0.02	3.12	(6.61%) (162) bridge BC: BEND
					(10.92%) (165) bridge CD: BEND
					(13.32%) (229) ring B-prop: 1-TORS
					(11.44%) (231) ring B-prop: 3-TORS
					(5.47%) (245) ring D-ethyl: 1-TORS
237	45.78	-------	4.07	0.53	(9.04%) (229) ring B-prop: 1-TORS
					(34.36%) (230) ring B-prop: 2-TORS
					(12.93%) (231) ring B-prop: 3-TORS
					(11.72%) (241) ring C-prop: 3-TORS
238	44.29	-------	0.64	0.32	(5.24%) (98) ring C torsion
					(6.56%) (180) ring C: bridge CD WAGG
					(8.74%) (230) ring B-prop: 2-TORS
					(35.05%) (231) ring B-prop: 3-TORS
					(14.33%) (241) ring C-prop: 3-TORS
239	41.32	-------	0.26	2.10	(5.38%) (241) ring C-prop: 3-TORS
					(5.50%) (243) bridge C=D: TORS
					(56.89%) (245) ring D-ethyl: 1-TORS
240	36.09	-------	0.91	0.82	(40.80%) (90) ring A torsion
					(7.24%) (234) bridge A=B: TORS
					(11.41%) (235) bridge A-B: TORS
					(5.10%) (241) ring C-prop: 3-TORS
					(10.07%) (245) ring D-ethyl: 1-TORS
241	29.80	-------	1.24	3.69	(11.96%) (90) ring A torsion
					(5.46%) (230) ring B-prop: 2-TORS
					(5.21%) (231) ring B-prop: 3-TORS
					(5.20%) (233) bridge BC: 1-TORS
					(9.70%) (237) bridge C-D: TORS
					(26.74%) (241) ring C-prop: 3-TORS
					(9.56%) (249) Cl OUT
242	28.60	-------	0.14	5.01	(7.80%) (90) ring A torsion
					(5.56%) (94) ring B torsion
					(10.14%) (136) ring B: bridge AB WAGG
					(7.45%) (140) ring B-prop: 1-WAGG

					(10.88%) (229) ring B-prop: 1-TORS
					(13.39%) (234) bridge A=B: TORS
					(7.77%) (239) ring C-prop: 1-TORS
					(5.78%) (241) ring C-prop: 3-TORS
243	26.78	-------	1.19	1.05	(6.26%) (140) ring B-prop: 1-WAGG
					(16.04%) (229) ring B-prop: 1-TORS
					(18.91%) (230) ring B-prop: 2-TORS
					(20.89%) (239) ring C-prop: 1-TORS
					(11.67%) (240) ring C-prop: 2-TORS
244	24.11	-------	0.26	2.18	(6.18%) (229) ring B-prop: 1-TORS
					(19.48%) (230) ring B-prop: 2-TORS
					(12.76%) (231) ring B-prop: 3-TORS
					(19.98%) (240) ring C-prop: 2-TORS
					(5.11%) (243) bridge C=D: TORS
					(6.44%) (245) ring D-ethyl: 1-TORS
245	21.99	-------	1.29	7.22	(5.60%) (103) ring D torsion
					(5.20%) (167) bridge CD: C-H OUT
					(6.34%) (230) ring B-prop: 2-TORS
					(14.58%) (237) bridge C-D: TORS
					(13.06%) (239) ring C-prop: 1-TORS
					(9.26%) (241) ring C-prop: 3-TORS
					(9.62%) (249) Cl OUT
246	19.64	-------	0.11	5.23	(8.16%) (162) bridge BC: BEND
					(11.43%) (165) bridge CD: BEND
					(9.82%) (179) ring C: bridge CD ROCK
					(5.80%) (240) ring C-prop: 2-TORS
					(9.34%) (245) ring D-ethyl: 1-TORS
247	15.36	-------	0.17	3.01	(21.20%) (90) ring A torsion
					(26.44%) (235) bridge A-B: TORS
					(8.55%) (236) bridge BC: 2-TORS
248	12.04	-------	0.92	34.60	(5.92%) (162) bridge BC: BEND
					(12.08%) (174) ring C: bridge BC WAGG
					(8.26%) (233) bridge BC: 1-TORS
					(28.80%) (237) bridge C-D: TORS
					(7.73%) (249) Cl OUT
249	10.61	-------	0.01	6.23	(5.26%) (90) ring A torsion
					(5.05%) (136) ring B: bridge AB WAGG
					(15.02%) (233) bridge BC: 1-TORS
					(5.26%) (235) bridge A-B: TORS
					(23.37%) (236) bridge BC: 2-TORS

Selection of calculated normal modes for PΦB in the ZZEssa configuration in H2O

```
Mode  frequ./cm-1          IR Int.  Raman Int  composition
48   1635.13   -------     2.94     1.09   (  7.66%) ( 70) C  =  C  STRE (30, 5)
                                             (  6.92%) ( 73) C  =  C  STRE (32,31)
                                             (  9.67%) ( 81) C  -  C  STRE (38,32)
                                             ( 45.99%) ( 83) C  -  C  STRE (40,38)
                                             ( 10.49%) (214)ring D, vinyl CH rock
                                             (  9.85%) (216)ring D, vinyl CH2 sym def

  49   1618.42   -------   479.92    14.49   (  6.29%) (  7) C  -  N  STRE (51,44)
                                             ( 17.09%) ( 41) C -.  C  STRE ( 3,68)
                                             ( 24.87%) ( 44) C  =  C  STRE ( 7,51)
                                             ( 10.96%) ( 48) C -.  C  STRE (11, 3)
                                             (  5.20%) (102)ring A   N-H  ROCK
                                             (  7.33%) (161)Methin BC C-H ROCK

  50   1610.62   -------   189.34   100.00   (  6.07%) ( 52) C  -  C  STRE (14, 5)
                                             ( 48.82%) ( 70) C  =  C  STRE (30, 5)
                                             (  5.93%) ( 71) C  -  N  STRE (30,28)
                                             (  5.67%) ( 83) C  -  C  STRE (40,38)
                                             ( 10.72%) (164)Methin CD C-H ROCK

  51   1594.44   -------  1285.18     0.56   ( 19.23%) ( 23) C -.  C  STRE (67,66)
                                             ( 10.09%) ( 41) C -.  C  STRE ( 3,68)
                                             ( 14.83%) ( 44) C  =  C  STRE ( 7,51)
                                             (  9.58%) ( 48) C -.  C  STRE (11, 3)
                                             (  5.62%) (102)ring A   N-H  ROCK
                                             (  8.28%) (161)Methin BC C-H ROCK

  52   1561.15   -------   207.84     3.24   ( 21.05%) ( 73) C  =  C  STRE (32,31)
                                             ( 15.92%) (131)ring B N-H ROCK
                                             ( 15.90%) (169)ring C N-H ROCK

  53   1556.08   -------   169.18    17.26   ( 37.25%) ( 73) C  =  C  STRE (32,31)
                                             (  6.76%) ( 83) C  -  C  STRE (40,38)
                                             ( 11.04%) (131)ring B N-H ROCK
                                             (  7.40%) (169)ring C N-H ROCK

  54   1545.71   -------  1215.07     3.54   ( 30.69%) ( 23) C -.  C  STRE (67,66)
                                             (  6.05%) ( 26) C  -  C  STRE (69,66)
                                             (  7.65%) ( 41) C -.  C  STRE ( 3,68)
                                             (  7.30%) ( 44) C  =  C  STRE ( 7,51)
                                             (  5.70%) ( 90) 5-membered ring B BEND
                                             (  7.12%) (102)ring A   N-H  ROCK

  55   1525.57   -------   143.43     8.61   (  6.30%) (  7) C  -  N  STRE (51,44)
                                             (  9.50%) ( 45) C  -  C  STRE ( 7,65)
                                             (  6.43%) ( 54) C  -  C  STRE (14,13)
                                             (  6.95%) (102)ring A   N-H  ROCK
                                             (  6.69%) (131)ring B N-H ROCK
                                             (  6.94%) (167)Methin AB C-H ROCK
                                             ( 18.38%) (169)ring C N-H ROCK

  56   1500.27   -------   178.62     3.05   ( 34.76%) ( 51) C -.  C  STRE (13,12)
                                             ( 10.83%) ( 53) C  -  N  STRE (14, 9)
                                             (  8.25%) ( 55) C  -  C  STRE (15,13)

 157    833.86   -------    82.05     1.85   ( 14.66%) ( 72) C  -  C  STRE (31,30)
                                             (  6.42%) ( 76) C  -  C  STRE (33,32)

 158    825.95   -------    35.30    41.22   ( 58.36%) (165)Methin CD C-H OUT
                                             (  9.58%) (201)ring D-Methin DC WAGG
                                             ( 10.45%) (237)torsion ring D-methin CD TORS

 159    807.98   -------    21.13     0.43   (  8.34%) ( 38) O  -  C  STRE (81,79)
                                             (  5.82%) (157)ring B, Prop C-O ROCK

 160    805.28   -------    13.25     0.07   ( 10.37%) (163)skel, Methin CD BEND
                                             (  5.98%) (191)ring C, Prop bCH2 ROCK

 161    797.60   -------    36.44     0.05   (  7.24%) (134)ring B-Methin BA WAGG
                                             ( 62.84%) (168)Methin AB C-H OUT
                                             ( 13.15%) (228)torsion ring A-methin AB TORS
```
